# Potassium Poly(Heptazine Imide): Transition Metal‐Free Solid‐State Triplet Sensitizer in Cascade Energy Transfer and [3+2]‐cycloadditions

**DOI:** 10.1002/anie.202004747

**Published:** 2020-06-09

**Authors:** Aleksandr Savateev, Nadezda V. Tarakina, Volker Strauss, Tanveer Hussain, Katharina ten Brummelhuis, José Manuel Sánchez Vadillo, Yevheniia Markushyna, Stefano Mazzanti, Alexander P. Tyutyunnik, Ralf Walczak, Martin Oschatz, Dirk M. Guldi, Amir Karton, Markus Antonietti

**Affiliations:** ^1^ Department of Colloid Chemistry Max Planck Institute of Colloids and Interfaces Am Mühlenberg 1 14476 Potsdam Germany; ^2^ School of Molecular Sciences The University of Western Australia 35 Stirling Highway 6009 Perth Western Australia Australia; ^3^ Institute of Secondary Education Maimónides Calle Alfonso XIII, 4 14001 Córdoba Spain; ^4^ Institute of Solid State Chemistry Ural Branch of the Russian Academy of Sciences 91 Pervomayskaya str. 620990 Ekaterinburg Russia; ^5^ Department of Chemistry and Pharmacy Interdisciplinary Center for Molecular Materials (ICMM) Friedrich-Alexander University of Erlangen-Nürnberg Egerlandstrasse 3 91058 Erlangen Germany

**Keywords:** carbon nitrides, organic photocatalysis, potassium poly(heptazine imide), singlet oxygen, solid-state sensitizer

## Abstract

Polymeric carbon nitride materials have been used in numerous light‐to‐energy conversion applications ranging from photocatalysis to optoelectronics. For a new application and modelling, we first refined the crystal structure of potassium poly(heptazine imide) (K‐PHI)—a benchmark carbon nitride material in photocatalysis—by means of X‐ray powder diffraction and transmission electron microscopy. Using the crystal structure of K‐PHI, periodic DFT calculations were performed to calculate the density‐of‐states (DOS) and localize intra band states (IBS). IBS were found to be responsible for the enhanced K‐PHI absorption in the near IR region, to serve as electron traps, and to be useful in energy transfer reactions. Once excited with visible light, carbon nitrides, in addition to the direct recombination, can also undergo singlet–triplet intersystem crossing. We utilized the K‐PHI centered triplet excited states to trigger a cascade of energy transfer reactions and, in turn, to sensitize, for example, singlet oxygen (^1^O_2_) as a starting point to synthesis up to 25 different N‐rich heterocycles.

## Graphical abstract

**The triplet excited states** of the new heterogeneous triplet sensitizer, potassium poly(heptazine imide),can be used to trigger a cascade of energy‐transfer reactions and, in turn, to sensitize, for example, singlet oxygen (^1^O_2_), as a starting point to synthesize N‐rich heterocycles. In this way aldoximes can be converted into 1,2,4‐oxadiazoles under visible light irradiation.

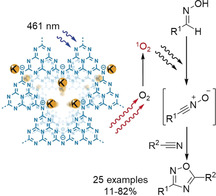

## Introduction

Artificial photosynthesis has been the primary area of inorganic semiconductors application in chemistry for many years.^1^, ^2^, ^3^ Similarly, carbon nitrides are traditionally associated with water splitting and CO_2_ conversion.^4^, ^5^, ^6^ In fact, the evolution of hydrogen from water in the case of polymeric carbon nitride impacted work in related areas, such as photoelectrochemical cells,^7^ metal‐free electrodes,^8^, ^9^ and electroluminescence devices.^10^ Applications beyond the aforementioned are autonomous actuators,^11^ photodetectors based on photon driven ion transport in asymmetric carbon nitride membranes,^12^ and light‐driven ion pump.^13^ Polymeric carbon nitride materials have also been recognized as versatile and reliable heterogeneous photocatalysts for preparing value‐added organic compounds.^14^, ^15^ Ghosh, König et al. showed, for example, that mesoporous graphitic carbon nitride (mpg‐CN) enables various kinds of reactions and also enables one‐pot C−H bifunctionalization of organic molecules.^16^ Extending this first generation catalysts, potassium poly(heptazine imide) (K‐PHI), a crystalline carbon nitride material, facilitates a remarkable number of unique reactions. Leading examples are oxidative thiolation of toluene at room temperature^17^ and multiple tandem reactions.^18^, ^19^ It is also capable to store electrons.^20^, ^21^

In the context of carbon nitride photocatalysts, the overwhelming majority of reactions is based on electron transfer. Energy transfer, on the other hand, generates excited‐state molecules rather than charged radicals. Therefore, new reaction paths can be designed.^22^ In terms of dioxygen, one of the greenest oxidants in organic synthesis and simplest bimodal reactant, one‐electron reduction leads to the superoxide radical (O_2_^.−^), while energy transfer from a triplet excited state sensitizer affords singlet oxygen (^1^O_2_). The chemistry of O_2_^.−^ and ^1^O_2_ could not be more different.^23^ O_2_^.−^ participates in proton abstraction, disproportionation, or nucleophilic substitution reactions,^24^ while the most prominent reactions of ^1^O_2_ are Diels–Alder cycloaddition and formation of dioxetanes.^25^, ^26^, ^27^

Numerous small organic compounds have been reported to sensitize ^1^O_2_: Ir(ppy)_3_,^28^ Ru(bpy)_3_Cl_2_,^29^ [Mes‐Acr]^+^ClO_4_^−^,^23^ riboflavin tetraacetate (RFT),^30^ just to name a few. Ease of separation, higher thermo‐chemical stability, and possible application on large scale make solid‐state sensitizers more attractive than homogeneous analogues. π‐conjugated triplet sensitizers have been a topic of research in past decades and have been quite successfully used in areas such as light energy conversion, LEDs fabrication,^31^, ^32^ photon upconversion,^33^, ^34^, ^35^ cells imaging.^36^ However, only few examples are known using such materials in photocatalysis.^37^ Notable is that their use has been restricted to “model” reactions.^38^ Most of these solid‐state sensitizers are made out of soft polymer matrices with encapsulated platinum group metal complexes.^39^

To date the modus operandi of carbon nitrides has always been linked to electron‐transfer reactions, while energy‐transfer reactions in most cases have not been even considered. Replacing hazardous and toxic oxidants by simpler and more sustainable chemicals, such as ^1^O_2_, is a central topic of contemporary research. By virtue of ^1^O_2_ mediated reactions, which are surprisingly restricted to the synthesis of model compounds only, many questions and challenges evolve around the function of carbon nitrides. Finally, we envisioned bimodal photocatalysis, featuring a redox mediator, which is also a photosensitizer, integrated into the same material, as a tool to intensify research applied in the synthesis of organic molecules.

**Figure 1:**
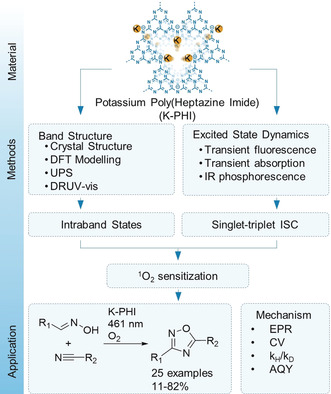
Topics covered in the current work and interconnections between them. Abbreviations are explained in the text.

Herein, we address a number of questions using K‐PHI (Figure 1). We refine the crystal structure of nanocrystalline K‐PHI, giving special attention to understanding the defects structure of this compound, and provided unambiguous evidence that light‐excited K‐PHI indeed undergoes singlet–triplet intersystem crossing (ISC).

As such, we employ a cascade of energy‐transfer reactions, starting with the K‐PHI triplet excited states to O_2_ and a subsequent quenching with aldoximes. Interaction of ^1^O_2_ with aldoximes gives nitrile oxides, which triggered our interest in exploring the synthesis of various oxadiazoles‐1,2,4 and isoxazoles via dipolar [3+2]‐cycloaddition.

## Results and Discussion

**Figure 2:**
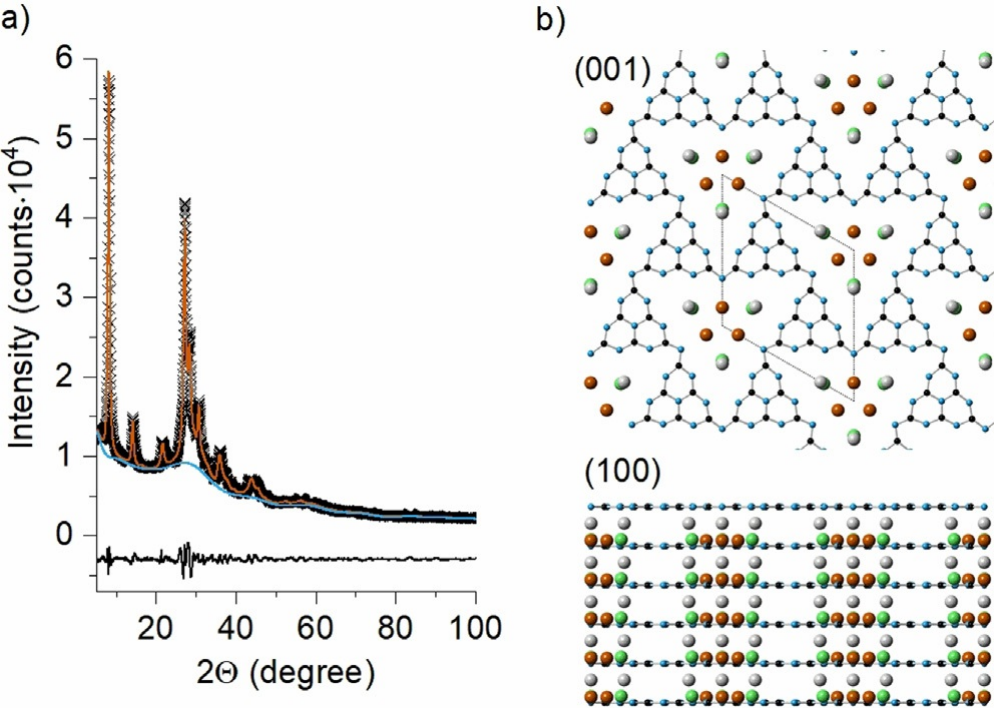
a) Experimental (crosses), calculated (solid line), and difference (bottom line) XRD patterns of K‐PHI, b) crystal structure of K‐PHI after refinement of XRD data. Blue spheres N sites, black spheres C sites; brown K(1), grey K(2), and green K(3).

K‐PHI was prepared from 5‐aminotetrazole in LiCl/KCl eutectics using mechanochemical pre‐treatment of the respective precursors,^40^ while details regarding its characterization are given in Figure S1 in the Supporting Information. At first glance, the collected X‐ray powder diffraction patterns of K‐PHI revealed both anisotropy in the shape of the Bragg peaks (in the 2*θ* range 20–42°) and a clearly pronounced diffuse halo, thus pointing towards the presence of defects/disorder (Figure 2 a). Having a better understanding of the real structure of K‐PHI and the degree of structural disorder is considered essential for explaining and tuning K‐PHI properties.

**Figure 3:**
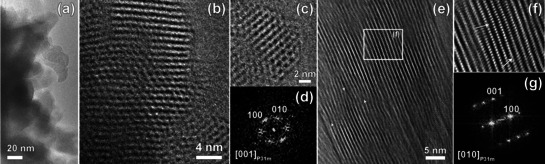
HRTEM images of K‐PHI: a) agglomerates of lamellar crystallites, b),c) view along (001) direction and d) corresponding FFT, e),f) view along (010) direction and g) corresponding FFT. White arrows indicate dislocations and rippling of CN‐layers.

To start building a structural model, a high‐resolution transmission electron microscopy (HRTEM) study was performed. K‐PHI powder consists of lamellar nanocrystallites, which form large agglomerates (Figure 3 a). Fast Fourier Transforms (FFTs) obtained from the HRTEM images can be indexed in a hexagonal lattice with unit cell parameters *a=*11.4(8) Å and *c=*3.7(2) Å. HRTEM images reveal a layered structure with nanometer‐sized domains that display unit cell distortions, faults in the sequences of CN‐layer stacking, edge and screw dislocations as well as rippling of CN‐layers (Figure 3).

The XRD pattern of K‐PHI can be correspondingly indexed in a hexagonal lattice with unit cell parameters: *a=*12.637(3) Å, *c=*3.2998(3) Å, space group *P*31*m* (157), which is in good agreement with TEM data. Based on HRTEM, XRD data, and general assumptions about the structure from earlier work,^41^ the starting model for refinement was proposed to consist of heptazine units, which are placed on top of each other in a so‐called AAA stacking forming continuous channels along the *c* direction. Models with different CN‐layers stacking, in particular ccp (ABCABC), hcp (ABAB) and mixed stacking (AABB, etc.) allowed the positions and the broadening of the peaks to be partially described, but did not give a better description of the XRD pattern, suggesting the presence of stacking faults rather than a second structural modification in the sample. Potassium atoms were located closer to the center of the channels and in between layers; their positions were corrected further during refinement based on difference Fourier maps. The details of the refinement, refined atomic coordinates, and temperature factors can be found in Tables S1 and S2.

Our results are in good agreement with the model presented by Lotsch et al.^42^ However, we believe that describing the nanocrystalline sample based on the model obtained for larger crystals^42^ would not be totally correct in our case. We think that a high symmetry description with defects and disorder included is closer to the real structure of the nanocrystalline sample and assists in explaining the high catalytic activity of K‐PHI.

From Table S2 we derive that the highest probability of finding any K atoms corresponds to the K(1) positions (brown spheres on Figure 2), suggesting that K atoms tend to sit closer to the center of the channels so that they can form bonds with bridging nitrogen atoms N(1).

Note that the structural model obtained is still idealized; in the real compound, there is a high degree of disorder, which is associated with 1) the stacking of PHI layers, and 2) a disordered distribution of K atoms (the low probabilities to find K(2) and K(3) at particular position suggests that atoms can be randomly distributed in between layers). Taking earlier reports into account, an optimum size of K‐PHI crystallites exists in terms of highest‐performing photocatalysis, which is based on electron transfer, such as dehydrogenation of alcohols.^40^

**Figure 4:**
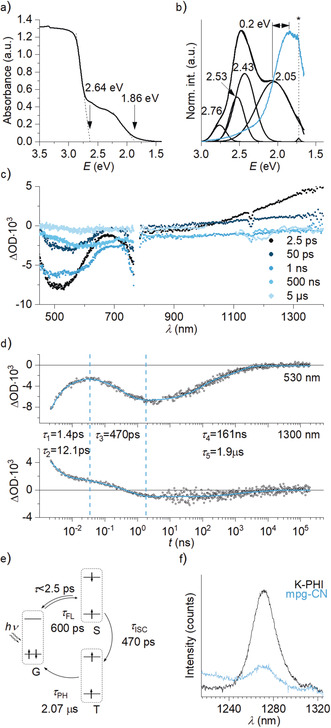
Spectroscopic characterization of K‐PHI. a) Solid‐state DRUV‐vis spectrum of K‐PHI. b) Room temperature steady‐state fluorescence spectrum (black curve, λ_ex_=360 nm) and deconvoluted fluorescence spectrum. Phosphorescence spectrum (light blue, λ_ex_=360 nm, delay 10 ms). The asterisk at 1.72 eV denotes 2nd order excitation light diffraction. c) Transient absorption spectra of K‐PHI suspension in MeCN (1.7 mg mL^−1^, λ_ex_=387 nm). d) Time‐absorption profiles of the TAS monitored at 530 nm and 1300 nm. Lifetime components obtained by fitting the data with exponential functions are shown. e) Summary of the excited state dynamics in K‐PHI. G ‐ground state, S singlet excited state, T  triplet excited state. f) ^1^O_2_ fluorescence (λ_ex_=320 nm, OD_320nm_=0.35). Suspension of carbon nitride in MeCN purged with O_2_.

Diffuse reflectance UV‐vis (DRUV‐vis) spectrum suggests that in the K‐PHI structure at least two band gaps exist (Figure 4 a). Of great relevance is the onset of absorption with an energy of 2.64 eV; it relates to the intrinsic optical band gap seen typically for graphitic carbon nitrides.^4^ A smooth onset at around 1.86 eV stands for low‐energy transitions, which involve intraband states (IBS).^43^

Room‐temperature steady‐state photoluminescence (PL) spectrum of K‐PHI is more complex compared to, for example, g‐C_3_N_4_,^43^ and involves at least 4 major transitions. The peaks at 2.76 eV, 2.53 eV, and 2.43 eV correspond to CBM‐to‐VBM transitions whereas that at 2.05 eV is assigned to CBM‐to‐IBS transitions (Figure 4 b).

To correlate the 600 ps *τ*_FL_,^17^ which is significantly shorter than typically observed for carbon nitride materials, (normally over 1 ns), with the photocatalytic activity of K‐PHI (shown below), we performed further spectroscopic characterization. The fluorescence internal quantum efficiency (IQE) of K‐PHI is 0.072 %. Relatively low IQE values speak for non‐radiative deactivations in carbon nitride once in the singlet excited state. Taking the extremely short fluorescence lifetime of 600 ps into account, we conclude that only 1 out of 1000 exciton separation events leads to recombination and that the vast majority of excitons reaches non‐radiative trap states in a matter of sub‐nanosecond time frame. However, it still remains unclear what is the quantum state of those.

By applying a 10 ms delay, the phosphorescence spectrum was recorded (Figure 4 b). The phosphorescence features are red‐shifted compared to the fluorescence. Given that fluorescence spectrum of K‐PHI comprises at least four transitions, with 47.7 % contribution of the peak at 2.05 eV to the total fluorescence (Table S3), ISC is more favorable when additional states are associated with this transition. In this view, the singlet–triplet energy gap in K‐PHI was calculated to be 0.2 eV (Figure 4 b). Earlier reported singlet–triplet energy gaps for graphitic carbon nitride range from 0.156 to 0.248 eV.^38^

To address the question on the quantum state of excitons in non‐radiative traps and to obtain insights into the excited state dynamics in K‐PHI, we conducted transient absorption measurements (Figure 4 c and Figure S6). Upon excitation at 387 nm, a broad minimum centered at 525 nm and a maximum at *λ*>1100 nm evolve. The minimum is assigned to ground state bleaching of the singlet excited state, while the maximum is assigned to excited‐state absorptions. Within a few hundred picoseconds, all of the aforementioned features transform into a broad negative transient stretching over the entire spectrum and minimizing at around 600 nm. This signal decays to zero after 10 μs. The steady deactivation of the short‐lived transient together with the evolution of the long‐lived transient points to a population of a triplet from the former singlet excited state.

Multi‐wavelength analysis of the time‐absorption profiles yields five lifetimes (Figure 4 d). The ground‐state recovery from the singlet excited state occurs with two lifetime components of *τ*_1_=1.4 ps and *τ*_2_=12.1 ps. A triplet excited state is populated via intersystem crossing within a lifetime of *τ*_3_=470 ps. The triplet finally deactivates with two lifetime components *τ*_4_=161 ns and *τ*_5_=1.9 μs. The presence of two lifetime components indicates the presence IBS.

Such a complex excited‐state dynamics of K‐PHI is very different from a carbon nitride reported by Wu et al. and Durrant et al., who described the excited state deactivation by a single exponential fitting function.^43^, ^44^ On the other hand, Xie et al. also observed by TAS formation of triplet states in carbon nitride, albeit intersystem crossing occurs within a few picoseconds.^45^ Figure 4 e summarizes the excited state dynamics in K‐PHI.

To explore the potential of the non‐radiative triplet excited states in K‐PHI to sensitize, for example, ^1^O_2_, we monitored the emission of K‐PHI suspension in MeCN saturated with O_2_ at 1271 nm. Indeed, we recorded the distinct ^1^O_2_ fluorescence pattern (Figure 4 f). mpg‐CN was also able to produce ^1^O_2_, albeit with significantly lower efficiency. Sensitization of ^1^O_2_ thereby evidences that triplet states in carbon nitride materials may be used for energy transfer in photocatalytic reactions as will be shown below.

To characterize the non‐radiative trap states, we invoked theoretical modelling of the K‐PHI structure. To date, the density functional theory (DFT) modeling has been carried out for diverse carbon nitrides.^43^, ^46^ Considering, however, the unique chemical structure of K‐PHI, we want to obtain a cohesive picture of its electronic structure.

**Figure 5:**
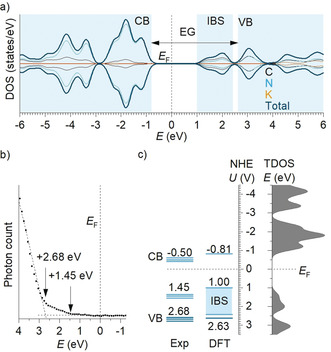
Band structure of K‐PHI. a) Partial (C, N, K) and total DOS in K‐PHI ground state. Fermi energy is located at 0 eV. b) UPS of K‐PHI. c) Experimental band structure of K‐PHI and that derived from DFT calculations. TDOS on the right is given as a guide for CBM, IBS, and VBM. Experimental data set: CB potential determined by Mott‐Schottky analysis.^47^ Uncertainty of IBS onset and VBM determination due to smooth signal onset in UPS and presence of several emissive states near CBM (Figure 4 b) are denoted by closely lying levels.

Using the refined crystal structure, we performed periodic DFT calculations to obtain the total and partial density of states (DOS) of K‐PHI. For our calculations, we took the K‐PHI structure bearing three K(1) potassium atoms (those with a fraction 0.7032 in Table S2, brown spheres on Figure 2 b). The DOS profile (Figure 5 a) reveal that the conduction band of K‐PHI is formed by s‐ and p‐orbitals of nitrogen (55 %) and carbon (26 %) followed by potassium s, p, and d‐orbitals (19 %) with the edge located at −0.81 eV. The experimentally determined flat‐band potential is −0.50 V versus the normalized hydrogen electrode (NHE).^47^

By means of ultraviolet photoelectron spectroscopy (UPS), the valence band maximum is determined at +2.68 eV (Figure 5 b). However, signal starts to develop at around +1.45 eV. This is indicative for the existence of IBS. Taking UPS and DRUV‐vis data as well as theoretical DOS modeling of K‐PHI (Figure 5 a) into account, we conclude that the IBS are located at approximately +1.45 eV. In the DOS of K‐PHI, IBS are observed as an “island” emerging at +1.00 eV and stretching up to +2.42 eV (Figure 5 a, c). Presence of a “valley” between +2.42 eV and +2.63 eV underlines that the states are well‐separated from the VB. The IBS are not caused by the introduction of potassium itself, as their corresponding contributions in the range of energies from +0.93 to +2.5 eV are negligibly small (0.48 %). Instead, we ascribe them to n‐π* transitions.^48^ In K‐PHI, the heptazine units are arranged in large macrocycles and each of them is formed by 6 heptazine units (Figure 2 b). Therefore, the K‐PHI structure is more flexible compared to the conventional graphitic carbon nitride, in which heptazine units either form a rigid conjugated 2D structure or are best described by chains of heptazine units bound by hydrogen bonds.^49^ Therefore, ensembles of heptazine units in K‐PHI can adopt out‐of‐plane conformations, which, in turn, facilitates the otherwise restricted n‐π* transitions.^50^

Figure 5 c summarizes the experimentally determined CBM and VBM in K‐PHI with that obtained from the DFT modelling. The difference between CBM (−0.50 eV calculated taking into account Mott‐Schottky plots) and VBM (+2.68 eV, determined from UPS) is 3.18 eV—the energy gap or transport gap, is larger than the optical band of 2.64 eV.^51^

The preliminary results of ^1^O_2_ addition to 9,10‐diphenylanthracene to undergo endoperoxide formation point to higher selectivity of the heterogeneous photocatalysis versus homogeneous system (Table S4). To test the K‐PHI‐assisted ^1^O_2_ sensitization in the synthesis of N‐rich heterocycles, we examine the synthesis of oxadiazoles‐1,2,4 in greater detail owing to the importance of this class of organic compounds for medicinal chemistry and material science.^52^, ^53^ We expect formation of nitrile oxides from the corresponding aldoximes upon ^1^O_2_ quenching followed by [3+2] cycloaddition to the C≡N‐group of a nitrile.

**Figure 6:**
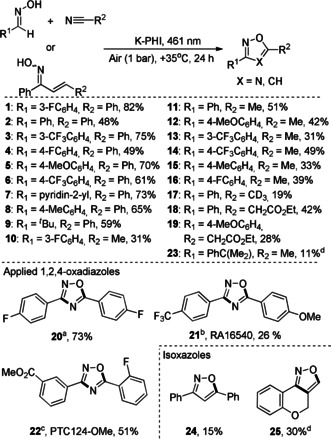
Photocatalytic synthesis of oxadiazoles‐1,2,4 and isoxazoles. Conditions: oxime 50 μmol, K‐PHI 5 mg, nitrile 3 mL (acetonitrile 3 mL was used in isoxazoles synthesis), air balloon 1 bar, 461 nm (88 mW cm^−2^), *T*=35 °C, 24 h. [a] Blue phosphorescent OLEDs precursor.^53^ [b] A potential drug for treatment of Alzheimer's disease.^54^ [c] A pharmaceutical drug (PTC124) precursor to target genetic disorders.^55^ [d] NMR yield.

Thorough reaction‐condition screening is given in Tables S5–S12. A series of oxadiazoles‐1,2,4 (**1**–**23**, Figure 6) was prepared by coupling different oximes with nitriles. Generally, the yields of oxadiazoles were higher combining aromatic nitriles with electron deficient oximes (Figure S10). For ethylcyanoacetate, cyclization toward oxadiazoles **18** and **19** competes with the Knoevenagel condensation. Using the developed method, we prepared several applied oxadiazoles derivatives.^53^, ^54^ An attempt to couple oxime of 3‐formylbenzoic acid with 2‐fluorobenzonitrile in order to prepare PTC124‐a pharmaceutical drug to target genetic disorders—was not successful.^55^ Instead, we succeeded in synthesizing the corresponding methyl ester **22**. The ester group of the oxadiazole is subjected to hydrolysis under alkaline conditions.^56^ Furthermore, we found that the intramolecular cyclization in chalcone oximes is triggered by K‐PHI under blue light irradiation. The tentative intermediate, namely 4,5‐dihydroisoxasole, is further oxidized to isoxazole **24**.

**Figure 7:**
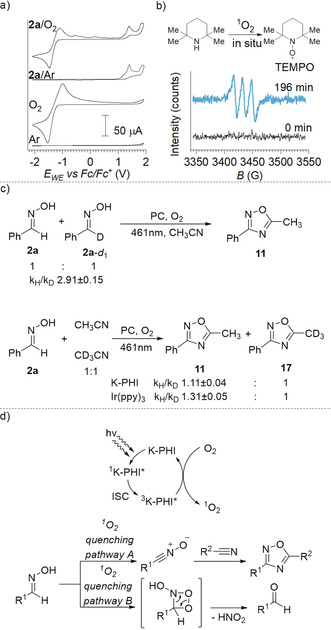
Mechanism investigation. a) Cyclic voltammetry study. From bottom to top: electrolyte purged with argon; electrolyte saturated with O_2_; a solution of benzaldehyde oxime **2 a** (2 mm) in electrolyte purged with Ar; a solution of benzaldehyde oxime **2 a** (2 mm) in electrolyte saturated with O_2_. b) EPR spectrum of in situ generated TEMPO. c) Primary and secondary KIE (mean ± SD., *n*=3) of oxadiazoles‐1,2,4 synthesis. d) Proposed mechanism of cascade energy transfer and ^1^O_2_ quenching via pathways A and B.

The reaction between benzaldehyde oxime and acetonitrile has been chosen to investigate the mechanism. The apparent quantum yield (AQY) of oxadiazole **11** reached (5.8±0.4)×10^−5^ % after 6 h of irradiation (Figure S11). The redox potentials of the reagents were determined by cyclic voltammetry (CV) in MeCN (Figure 7 a). Acetonitrile is stable against oxidation and reduction in the range from −2.1 V to +1.9 V versus Fc/Fc^+^ (Fc=[(η‐C_5_H_5_)_2_Fe]. Relative to the aforementioned, the standard redox potential of the O_2_/O_2_^.−^ couple is centered at −1.27 V.

Given that the potential of the conduction band of K‐PHI is located at −0.9 V vs. Fc/Fc^+^ (−0.5 V vs. NHE), reduction of O_2_ to afford O_2_^.−^ is thermodynamically challenging. Therefore, electrochemical measurements also suggest that ^1^O_2_ sensitization is the preferential path of O_2_ activation. Depending on the oxime structure, the onset of irreversible oxidation occurs in the range from +0.51 V, for pyrrole‐2‐carbaldehyde oxime **28 a**, to +1.67 V versus Fc/Fc^+^, for pivalaldehyde oxime **9 a** (Figure S12). Reaction in the presence of electron scavengers, such as nitrobenzene and S_8_,^17^ excluded participation of the photogenerated holes in the synthesis of oxadiazoles‐1,2,4 (Table S6). Addition of DMPO to the reaction mixture did not lead to the formation of the DMPO‐O_2_^.−^ adduct as evidenced by EPR spectroscopy (Figure S7). On the contrary, synthesis of 9,10‐diphenylantracene endoperoxide (Table S4), ^1^O_2_ fluorescence detection (Figure 4 f), and detection of TEMPO radical (Figure 7 b) formed in situ from 2,2,6,6‐tretramethylpiperidine univocally confirmed participation of ^1^O_2_ in the reaction.^57^

When present in equal amount, benzaldehyde oxime **2 a** is converted into oxadiazole **11** 2.91±0.15 faster than **2 a‐*d***_**1**_ (primary kinetic isotope effect, KIE, Figure 7 c). The results suggest that breaking of the C−D bond is the rate limiting step. When benzaldehyde oxime **2 a** was mixed with CH_3_CN and CD_3_CN in a 1:1 ratio, under the photocatalytic conditions using K‐PHI, CH_3_‐substituted oxadiazole **11** forms 1.11±0.04 times faster (secondary KIE) compared to the CD_3_‐substituted **17** oxadiazole. In the case of Ir(ppy)_3_, the secondary KIE is 1.31±0.05. Compared to the homogeneous catalysis, K‐PHI reduces the isotope effect due to the polarization of the C−D bonds induced by the electrostatic field on the K‐PHI surface. Zeta potential measurements resulted in a value of −40 mV for K‐PHI.^58^

Even though the vast majority of oximes quench ^1^O_2_ by means of dehydrogenation, a number of electron‐rich oximes including **26 a**–**31 a** fail to undergo click‐heterocyclization (Figure S13). Instead, aldehydes are formed as the products of C=N bond cleavage. A likely rationale is based on the formation of an intermediate charge‐transfer complex between the electron‐rich π‐conjugated system (**27 a**–**30 a**) and ^1^O_2_.^59^, ^60^ Once formed, the enriched electron density on dioxygen facilitates nucleophilic addition to the C=N bond followed by recovery of the aldehyde. Notably, carboxylic or phenolic protons as part of oximes **26 a** and **31 a** inhibit the pathway A.^61^

Taking the experimental data into account, we gather a tentative mechanism of ^1^O_2_ quenching by oximes, which is summarized in Figure 7 d. Depending on the chemical structure of oxime, its interaction with ^1^O_2_ is likely to follow two different pathways, namely oxidation to the nitrile oxide (pathway A) or addition to the C=N bond (pathway B). Pathway A is operative in most of the studied cases. Pathway B requires electron‐rich oximes or oximes bearing acidic protons. Independent support for our mechanistic conclusions came from solvent‐screening tests (Table S10). In the absence of, for example, click‐cyclizable nitriles, pathway B for benzaldehyde oxime **2 a** is activated exclusively. In stark contrast, solvents, which are capable of effectively quenching ^1^O_2_, such as 1,4‐dioxane, anisole, DMSO, DMF, inhibit both pathways. In the case of 1,4‐dioxane, the conversion of oxime **2 a** is 46 % next to 19 % of 3,5‐diphenyl‐1,2,4‐oxadiazole, which evolves as the product of nitrile oxide dimerization.

## Conclusion

Our investigations started with determining the crystal structure of K‐PHI. We showed that it crystallizes in a hexagonal lattice with unit cell parameters: *a=*12.637(3) Å, *c=*3.2998(3) Å, space group *P*31*m*. We described stacking disorder in the PHI layers and the non‐uniform distribution of K^+^‐ions in the structure. Still, with highest probability of K atoms to be found in the center of the channels so that they can form bonds with bridging nitrogen atoms N(1). Once the crystal structure of K‐PHI was defined, we performed periodic DFT modelling to calculate, the electronic band structure and the density of states. We reached a sound agreement with the experiments in terms of intraband electronic states at around +1.45 V as the origin of sizeable absorption in the near‐IR enabling energy‐transfer reactions. Once excited with visible light, a broad range of photochemical responses sets in. Most interestingly is the fact that K‐PHI undergoes significant singlet–triplet intersystem crossing across a 0.20 eV energy gap affording a reasonably long‐lived triplet excited state. In terms of dynamics, the combination of TAS and TCSPC enabled deriving 600 ps for the radiative decay of the singlet excited state, 470 ps for the non‐radiative intersystem crossing, 2.07 μs for the non‐radiative decay of the triplet excited state.

Our work was rounded off by utilizing the triplet excited states in K‐PHI to sensitize ^1^O_2_ and, in turn, to employ ^1^O_2_ in its reaction with a broad range of oximes. Hereby, two pathways of ^1^O_2_ quenching exist: first (pathway A), it is the oxime dehydrogenation, which affords nitrile oxide and, second (pathway B), it is the addition to oxime C=N bond, which results in aldehyde formation. When ^1^O_2_ quenching is performed via pathway A in, for example, the presence of reactive multiple bonds (C≡N, C=C), the nitrile oxides undergo [3+2]‐cycloaddition. In total, 25 examples of oxadiazoles‐1,2,4 and isoxazoles were synthesized in 11–82 % yield.

In the context of this work, the developed photocatalytic [3+2]‐cycloaddition reaction is applicable for functionalization of polymers, such as those with pendant multiple bonds. At the same time, molecules, other than O_2_, can be used as energy acceptors to broaden classes of organic compounds accessible via carbon nitride photocatalysis. These are ongoing projects.

## Conflict of interest

The authors declare no conflict of interest.

## Supporting information

As a service to our authors and readers, this journal provides supporting information supplied by the authors. Such materials are peer reviewed and may be re‐organized for online delivery, but are not copy‐edited or typeset. Technical support issues arising from supporting information (other than missing files) should be addressed to the authors.

Supplementary
