# Supplementary material for: Potassium Poly(Heptazine Imide): Transition Metal‐Free Solid‐State Triplet Sensitizer in Cascade Energy Transfer and [3+2]‐cycloadditions
Source: Angew Chem Int Ed Engl. 2020 Jun 9;59(35):15061–8. doi: 10.1002/anie.202004747 (PMC7496904; doi:10.1002/anie.202004747)
Supplement: Supplementary file 1 — Supplementary [file ANIE-59-15061-s001.pdf]

Angewandte Chemie
Eine Zeitschrift der Gesellschaft Deutscher Chemiker

Supporting Information

Potassium Poly(Heptazine Imide): Transition Metal-Free Solid-State Triplet Sensitizer in Cascade Energy Transfer and [3+2]-cycloadditions

Aleksandr Savateev, Nadezda V. Tarakina, Volker Strauss, Tanveer Hussain, Katharina ten Brummelhuis, José Manuel Sánchez Vadillo, Yevheniia Markushyna, Stefano Mazzanti, Alexander P. Tyutyunnik, Ralf Walczak, Martin Oschatz, Dirk M. Guldi, Amir Karton, and Markus Antonietti**

anie_202004747_sm_miscellaneous_information.pdf

Table of Contents

1. Materials.. 3

1.1. Chemicals.. 3

1.2. Preparation of oximes 5

1.2.1. A general procedure of oximes 1a, 4a, 5a, 6a, 7a, 8a, 22a, 26a, 28a, 29a, 30a, 31a preparation 5

1.2.2. A general procedure of oximes 2a, 2a-d_1, 3a, 9a, 23a, 25a, 27a preparation.. 5

1.2.3. Preparation of 1,3-diphenylprop-2-en-1-one oxime 24a 9

1.3. Synthesis of benzimidoyl chlorides 10

1.4. Synthesis of benzonitrile oxide 11

1.5. Synthesis of 3-(3-fluorophenyl)-5-phenyl-1,2,4-oxadiazole from 3-fluoro-N-hydroxybenzimidoyl chloride.. 11

1.6. Synthesis of the photocatalysts.. 11

1.6.1. Synthesis of K-PHI 11

Figure S1.K-PHI characterization.[1] 12

1.6.2. Synthesis of mpg-CN.. 12

1.6.3. Synthesis of RFT.. 13

2. Light source 14

Figure S2 14

3. Supplementary methods 15

3.1. NMR.. 15

3.2. GC-MS.. 15

3.3. High-resolution mass spectra 15

3.4. TEM investigation of K-PHI 15

3.5. X-Ray powder diffraction (XRD) of K-PHI.. 15

3.6. K-PHI XRD pattern fitting 15

Table S1. Structural data for K-PHI 16

Table S2. Atomic coordinates and isotropic thermal parameters for K-PHI. 16

3.7. Computational details 17

3.8. Fluorescence measurements 18

Figure S4. Fluorescence spectra of mpg-CN and K-PHI. 19
3.9. Phosphorescence measurements 19
Figure S5. Phosphorescence spectra of mpg-CN and K-PHI. 20
3.10. Transient absorption spectroscopy (TAS) 20
Figure S6. Transient absorption spectra of a K-PHI. 20
3.11. Singlet-oxygen fluorescence measurements 21
3.12. Cyclic voltammetry 21
3.12.1. CV of oximes under Ar atmosphere 21
3.13. Time-dependent AQY study 22
3.14. Kinetic Isotope Effect determination 22
3.15. EPR study 23
Figure S7. An attempt of DMPO-$O_2^{\bullet -}$ adduct detection by EPR. 24
3.16. Photocatalysts TON calculation of oxime 1a conversion 25
Figure S8. Dependence of v_i/D_i versus D_i. 25
3.17. K-PHI characterization methods 26
Figure S9. TGA of K-PHI and first derivative. 27
4. Photocatalytic experiments 28
4.1. Reaction of the photosensitized $^1O_2$ with 9,10-diphenylantracene 28
Table S4. Test reaction of singlet oxygen addition to 9,10-diphenylathracene.[a] 29
4.2. A general procedure of 1,2,4-oxadiazoles preparation using K-PHI photocatalyst 29
Table S5. Screening of the catalysts in synthesis of oxadiazole-1,2,4.[a] 35
Figure S10. Comparison of oxadiazoles-1,2,4 yields derived from a reaction between aldehyde oximes and either acetonitrile or benzonitrile. 36
Table S6. Electron acceptors/additives screening. 37
Table S7. Influence of oxygen concentration on 5-methyl-3-phenyl-1,2,4-oxadiazole yield.[a] 38
Table S8. Reaction conditions screening.[a] 39
Table S9. Influence of water content. 40
Table S10. Solvent screening.[a] 41
Table S11. Variation of the photocatalyst amount.[a] 42
Table S12. Solvents screening for a reaction between 3-fluorobenzaldehyde oxime and benzonitrile. 43
4.3. A general procedure of isoxazoles synthesis using K-PHI photocatalyst 44
Figure S11. Time-dependent AQY study. 45
Figure S12. Cyclic voltammetry study on aldehyde oximes. 46
Figure S13. Oximes that exclusively undergo path B (deoxymation path). 47
Figure S14. Side products identification. 47

5. NMR spectra 48
5.1. NMR spectra of oximes 1a-31a 48
5.2. NMR spectra of N-hydroxybenzimidoyl chlorides and nitrile oxide 65
5.3. NMR spectra of oxadiazoles-1,2,4 1-23 and isoxazoles 24,25 68
5.4. NMR spectra of 9,10-diphenylanthracene endoperoxide 94
5.5. NMR spectra of tentative by-products of oximes 3a and 23a oxidation 96
6. Mass spectra of tentative by-products of oximes 3a and 23a oxidation 100
6.1. Electron ionization spectra 100
6.2. High-resolution mass spectra 102
References 103

1. Materials

1.1. Chemicals

Acetonitrile (≥99.8%), tetrabutylammonium perchlorate (>98%), 3-formylbenzoic acid (97%), thionyl chloride (99+%), acrylonitrile (+99%), acetonitrile-d_3 (99.5 atom % D), dimethylsulfoxide-d_6 (99.5 atom % D), chloroform-*d* (99.8 atom % D), benzonitrile (≥99%), hydroxylamine hydrochloride (≥96%), pyridine (≥99.5%), 3-fluorobenzaldehyde (>97%), benzaldehyde (98+%), benzaldehyde-α-d_1 (98 atom % D), 3-(trifluoromethyl)benzaldehyde (>95%), 4-fluorobenzaldehdye (>97%), 4-methoxybenzaldehyde (98%), 4-(trifluoromethyl)benzaldehyde (>95%), picolinaldehyde (99%), 4-methylbenzaldehyde (>98%), pivalaldehyde (96%), butyraldehyde (>98%), 1H-pyrrole-2-carbaldehyde (98%), thiophene-2-carbaldehyde (98%), 2,6-dimethoxybenzaldehyde (99%), 4-hydroxy-3-methoxybenzaldehyde (≥98%), pyrene-1-carbaldehyde (>98%), 4-formylbenzonitrile (>98%), 2-methyl-2-phenylpropanoic acid (>96%), 2-(allyloxy)benzaldehyde (>96%), 9,10-diphenylantracene (>98%), Ir(ppy)$_3$ (99%), Ru(bpy)$_3$Cl$_2$·6H$_2$O (99.95%), riboflavin (pure, pharma grade), methylene blue·3H$_2$O (high purity), 9-Mesityl-10-methylacridinium perchlorate (95%), nitrobenzene (99.8%), methylviologen dichloride hydrate (98%), 2,2'-azobis(isobutyronitrile) (≥98%), hexaketocyclohexane octahydrate (99%), *p*-benzoquinone (≥98%), diphenyldisulfide (99%), octaatomic sulfur (≥99.5%), *tert*-butylnitrite (90%), acetone (HPLC grade), phosphate buffer (pH = 9), 1,4-dioxane (≥99.5%), dichloromethane (≥99.9%), *tert*-butanol (≥99.5%), anisole (≥99%), hexane (98%), benzene (99.8%), nitromethane (≥95%), ethyleneglycol dimethyl ether (99.5%), dimethylsulfoxide (≥99.5%), *N,N*-dimethylformamide (≥99.8%), α,α,α-trifluorotoluene (>98%), 1-Ethyl-3-methylimidazolium trifluoromethanesulfonate (≥98%), 1,1,2,2-tetrachloroethane (>97%), ethyl 2-cyanoacetate (98+%), 4-fluorobenzonitrile (>99%), 4-methoxybenzonitrile (>98%), 2-fluorobenzonitrile (>98%), butyronitrile (≥99%), cyanamide (99%), ammonium hydrogen difluoride (95%) were purchased from the vendors and used without additional purification.

2,2,6,6-tetramethylpyperidine (≥99%) was purchased from Sigma-Aldrich and distilled over KOH in vacuum (50 mbar) prior using.

Methyl 3-formylbenzoate

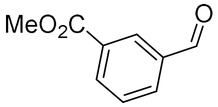

$SOCl_2$ (0.375 mL) was added dropwise to a stirred solution of 3-formylbenzoic acid (0.25 g, 1.7 mmol) in MeOH (3.75 mL) upon cooling on the ice bath. The solution was stirred at room temperature overnight and concentrated in vacuum (50°C, 30 mbar). The residue was dissolved in a mixture of dioxane/water (1:1), stirred at room temperature for 1 h, concentrated in vacuum (65°C, 30 mbar). Yield: 228 mg, 82%. ^{1}H NMR (400 MHz, Methanol-d_4) δ 10.08 (s, 1H), 8.53 (t, J = 1.5 Hz, 1H), 8.30 (dt, J = 7.7, 1.5 Hz, 1H), 8.09 (dt, J = 7.7, 1.4 Hz, 1H), 7.63 (t, J = 7.7 Hz, 1H), 3.97 (s, 3H).

1,3-Diphenylprop-2-en-1-one

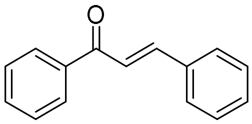

1,3-Diphenylprop-2-en-1-one was synthesized according to the literature procedure.[1]

2-Methyl-2-phenylpropanal

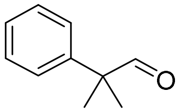

2-Methyl-2-phenylpropanal was synthesized according to the literature procedure with slight modifications.[2] $LiAlH_4$ (3.01 g, 79 mmol) was added in portions to a stirred solution of 2-methyl-2-phenylpropanoic acid (4.00 g, 24 mmol) in anhydrous Et_2O (450 mL) cooled on the ice bath within 20 min. Ice-bath was removed and stirring was continued at room temperature overnight. Suspension was cooled on the ice bath and HCl aqueous solution (40 mL, 1M) was added dropwise at stirring. Liquid was decanted. Water was added to the residue, acidified to pH = 1 and extracted with Et_2O (3 x 150 mL). Ethereal solutions were combined, washed with brine, dried over anhydrous Na_2SO_4 and concentrated in vacuum giving pale-yellow oil (3.6 g).

The oil (3.6 g) was dissolved in CH_2Cl_2 (98 mL) and cooled on the ice bath under N_2. Potassium acetate (2.87 g, 29 mmol) was added to a stirred CH_2Cl_2 solution followed by addition of pyridimiumchlorochromate (6.4 g, 29.7 mmol). The reaction mixture was stirred on the ice bath for 3 h and at room temperature overnight. The reaction mixture was diluted with ethylacetate, filtered through celite pad and concentrated in vacuum (+50°C, 50 mbar). The residue was fractioned in vacuum. The product was collected as a fraction with b.p.

+60…+80°C at $3 \cdot 10^{-5}$ bar into a receiver cooled with liquid nitrogen. Yield: 2.48 g, 70% (after two steps). ^{1}H NMR (400 MHz, Chloroform-*d*) δ 9.50 (s, 1H), 7.43 – 7.27 (m, 5H), 1.47 (s, 6H).

1.2. Preparation of oximes

1.2.1. A general procedure of oximes 1a, 4a, 5a, 6a, 7a, 8a, 22a, 26a, 28a, 29a, 30a, 31a preparation

A solution of aldehyde (typically 5 mmol, 1 equiv.), $NH_2OH \cdot HCl$ (1.2 equiv.) and $NaHCO_3$ (1.2 equiv.) in a mixture of EtOH/H_2O (1:1, 50 mL) was stirred at room temperature for 24 h until reaction is completed. The reaction mixture was concentrated in vacuum and deionized water (20 mL) was added to the residue. The solid was filtered and washed with water (3x20 mL). The solid was dried in vacuum (50°C, 30 mbar) overnight.

1.2.2. A general procedure of oximes 2a, 2a-d_1, 3a, 9a, 23a, 25a, 27a preparation

A solution of aldehyde (typically 5 mmol, 1 equiv.), $NH_2OH \cdot HCl$ (1.2 equiv.) and $NaHCO_3$ (1.2 equiv.) in a mixture of EtOH/H_2O (1:1, 50 mL) was stirred at room temperature for 24 h until reaction is completed. The reaction mixture was concentrated in vacuum and deionized water (20 mL) was added to the residue. The product was extracted with CH_2Cl_2 (3x10 mL). Organic solutions were combined, dried over anhydrous Na_2SO_4 and concentrated in vacuum (50°C, 30 mbar).

3-fluorobenzaldehyde oxime **1a**

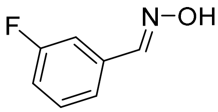

Yield: 75%. ^{1}H NMR (400 MHz, DMSO-d_6) δ 11.45 (s, 1H), 8.16 (s, 1H), 7.46 – 7.37 (m, 3H), 7.24 – 7.18 (m, 1H). ^{13}C NMR (101 MHz, DMSO-d_6) δ 162.3 (d, J = 243.4 Hz), 147.2 (d, J = 3.0 Hz), 135.6 (d, J = 8.1 Hz), 130.8 (d, J = 8.4 Hz), 122.7 (d, J = 2.7 Hz), 116.1 (d, J = 21.3 Hz), 112.6 (d, J = 22.5 Hz).

Benzaldehyde oxime **2a**

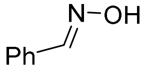

Yield: 85 %. ^{1}H NMR (400 MHz, Chloroform-*d*) δ 8.58 (br s, 1H), 8.17 (s, 1H), 7.67 – 7.51 (m, 2H), 7.43 – 7.37 (m, 3H).

3-(Trifluoromethyl)benzaldehyde oxime **3a**

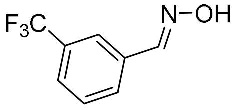

Yield: 80%. ^{1}H NMR (400 MHz, DMSO-d_6) δ 11.54 (s, 1H), 8.27 (s, 1H), 7.91 (d, *J* = 6.7 Hz, 2H), 7.74 (d, *J* = 7.8 Hz, 1H), 7.64 (t, *J* = 7.9 Hz, 1H). ^{13}C NMR (101 MHz, DMSO-d_6) δ 147.1, 134.3, 130.0, 129.5 (q, *J* = 31.5 Hz), 125.7 (q, *J* = 3.2 Hz), 124.3 (q, *J* = 270.0 Hz), 122.9 (q, *J* = 4.0 Hz).

4-Fluorobenzaldehyde oxime **4a**

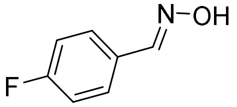

Yield: 83%. ^{1}H NMR (400 MHz, DMSO-d_6) δ 11.25 (s, 1H), 8.15 (s, 1H), 7.64 (ddd, *J* = 8.5, 5.3, 2.5 Hz, 2H), 7.27 – 7.20 (m, 2H). ^{13}C NMR (101 MHz, DMSO-d_6) δ 162.6 (d, *J* = 246.4 Hz), 147.1, 129.7 (d, *J* = 3.1 Hz), 128.5 (d, *J* = 8.4 Hz), 115.8 (d, *J* = 21.9 Hz).

4-Methoxybenzaldehyde oxime **5a**

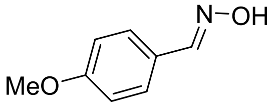

Yield: 80%. ^{1}H NMR (400 MHz, DMSO-d_6) δ 10.98 (s, 1H), 8.06 (s, 1H), 7.52 (d, *J* = 8.6 Hz, 2H), 6.95 (d, *J* = 8.6 Hz, 2H), 3.77 (s, 3H). ^{13}C NMR (101 MHz, DMSO-d_6) δ 160.1, 147.7, 127.9, 125.6, 114.2, 55.2.

4-(Trifluoromethyl)benzaldehyde oxime **6a**

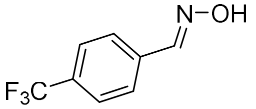

Yield: 87%. ^{1}H NMR (400 MHz, Chloroform-*d*) δ 8.2 (s, 1H), 7.7 (d, *J* = 8.4 Hz, 2H), 7.6 (d, *J* = 8.4 Hz, 2H). ^{13}C NMR (101 MHz, Chloroform-*d*) δ 149.3, 135.4 (q, *J* = 2.0 Hz), 131.9 (q, *J* = 33.0 Hz), 127.4, 125.9 (q, *J* = 3.8 Hz), 124.0 (q, *J* = 272.2 Hz).

Picolinaldehyde oxime **7a**

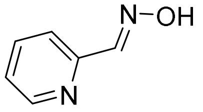

Yield: 63%. ^{1}H NMR (400 MHz, DMSO-d_6) δ 11.7 (s, 1H), 8.6 (dt, *J* = 4.9, 1.4 Hz, 1H), 8.1 (s, 1H), 7.8 – 7.8 (m, 2H), 7.4 (ddd, *J* = 6.8, 4.9, 1.7 Hz, 1H). ^{13}C NMR (101 MHz, DMSO-d_6) δ 152.1, 149.5, 149.0, 136.8, 124.0, 119.8.

4-Methylbenzaldehyde oxime **8a**

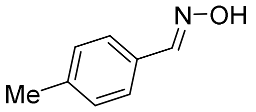

Yield: 68%. ^{1}H NMR (400 MHz, DMSO-d_6) δ 11.12 (s, 1H), 8.09 (s, 1H), 7.47 (d, *J* = 8.1 Hz, 2H), 7.20 (d, *J* = 7.9 Hz, 2H), 2.30 (s, 3H). ^{13}C NMR (101 MHz, DMSO-d_6) δ 148.0, 138.9, 130.3, 129.3, 126.4, 21.0.

Pivalaldehyde oxime **9a**

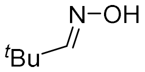

Yield: 77%. ^{1}H NMR (400 MHz, Chloroform-*d*) δ 7.85 (br s, 1H), 7.35 (s, 1H), 1.11 (s, 9H). ^{13}C NMR (101 MHz, Chloroform-*d*) δ 159.6, 33.8, 27.5.

Methyl 3-((hydroxyimino)methyl)benzoate **22a**

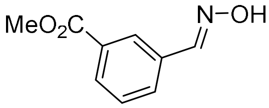

Yield: 74%. ^{1}H NMR (400 MHz, DMSO-d_6) δ 11.4 (s, 1H), 8.2 (s, 1H), 8.2 (s, 1H), 7.9 (d, *J* = 7.8 Hz, 1H), 7.9 (d, *J* = 7.8 Hz, 1H), 7.6 (t, *J* = 7.7 Hz, 1H), 3.9 (s, 3H). ^{13}C NMR (101 MHz, DMSO-d_6) δ 165.9, 147.4, 133.8, 130.8, 130.1, 129.7, 129.3, 127.0, 52.4.

2-Methyl-2-phenylpropanal oxime **23a**

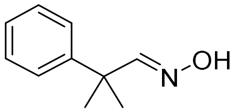

Yield: 74%. ^{1}H NMR (400 MHz, DMSO-d_6) δ 10.61 (s, 1H), 7.41 (s, 1H), 7.36 – 7.30 (m, 4H), 7.24 – 7.20 (m, 1H), 1.41 (s, 7H). ^{13}C NMR (101 MHz, DMSO-d_6) δ 155.7, 128.4, 126.3, 125.9, 40.5, 26.8.

2-(Allyloxy)benzaldehyde oxime **25a**

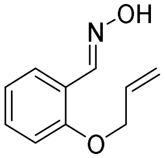

Yield: 84%. ^{1}H NMR (400 MHz, DMSO-d_6) δ 11.27 (s, 1H), 8.34 (s, 1H), 7.68 (dd, *J* = 7.7, 1.4 Hz, 1H), 7.36 – 7.31 (m, 1H), 7.05 (d, *J* = 8.3 Hz, 1H), 6.96 (t, *J* = 7.5 Hz, 1H), 6.06 (ddt, *J* = 17.1, 10.4, 5.1 Hz, 1H), 5.40 (dd, *J* = 17.3, 1.6 Hz, 1H), 5.29 – 5.25 (m, 1H), 4.61 (d, *J* = 5.1 Hz, 2H). ^{13}C NMR (101 MHz, DMSO-d_6) δ 155.8, 143.5, 133.6, 130.8, 125.5, 121.3, 120.9, 117.5, 113.0, 68.6.

3-((Hydroxyimino)methyl)benzoic acid **26a**

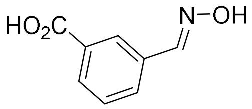

Yield: 73%. ^{1}H NMR (400 MHz, DMSO-d_6) δ 11.40 (s, 1H), 8.23 (s, 1H), 8.16 (s, 1H), 7.92 (d, *J* = 7.8 Hz, 1H), 7.82 (d, *J* = 7.8 Hz, 1H), 7.52 (t, *J* = 7.7 Hz, 1H).

Butyraldehyde oxime **27a**

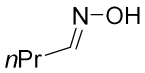

Yield: 49%. Mixture of cis- and trans-isomers (~1:1). ^{1}H NMR (400 MHz, DMSO-d_6) δ 10.73 (s, 1H), 10.37 (s, 1H), 7.28 (t, *J* = 5.9 Hz, 1H), 6.63 (t, *J* = 5.3 Hz, 1H), 2.20 (td, *J* = 7.4, 5.4 Hz, 2H), 2.07 (td, *J* = 7.3, 6.0 Hz, 2H), 1.43 (m, *J* = 7.4 Hz, 2H), 1.42 (m, *J* = 7.4 Hz, 2H), 0.89 (t, *J* = 7.4 Hz, 2H), 0.88 (t, *J* = 7.4 Hz, 2H). ^{13}C NMR (101 MHz, DMSO-d_6) δ 150.3, 149.4, 31.0, 26.6, 19.7, 19.1, 13.9, 13.6.

1H-Pyrrole-2-carbaldehyde oxime **28a**

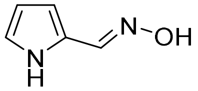

Yield: 37%. ^{1}H NMR (400 MHz, DMSO-d_6) δ 11.18 (s, 1H), 11.14 (s, 1H), 7.26 (s, 1H), 6.88 (q, *J* = 2.6 Hz, 1H), 6.55 (dt, *J* = 3.7, 2.2 Hz, 1H), 6.11 (q, *J* = 2.5 Hz, 1H). ^{13}C NMR (101 MHz, DMSO-d_6) δ 137.3, 124.0, 121.1, 114.0, 108.5.

Thiophene-2-carbaldehyde oxime **29a**

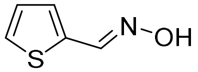

Yield: 47%. ^{1}H NMR (400 MHz, DMSO-d_6) δ 11.88 (s, 1H), 7.85 (s, 1H), 7.73 (d, *J* = 5.1 Hz, 1H), 7.47 (dd, *J* = 3.7, 1.0 Hz, 1H), 7.13 (dd, *J* = 5.1, 3.7 Hz, 1H). ^{13}C NMR (101 MHz, DMSO-d_6) δ 139.9, 131.2, 131.1, 131.1, 126.3.

2,6-Dimethoxybenzaldehyde oxime **30a**

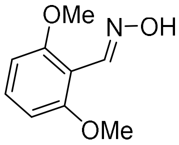

Yield: 86%. ^{1}H NMR (400 MHz, DMSO-d_6) δ 11.07 (s, 1H), 8.17 (s, 1H), 7.30 (t, *J* = 8.4 Hz, 1H), 6.69 (d, *J* = 8.4 Hz, 2H), 3.77 (s, 6H). ^{13}C NMR (101 MHz, DMSO-d_6) δ 158.3, 142.4, 130.5, 109.3, 104.2, 55.8.

4-Hydroxy-3-methoxybenzaldehyde oxime **31a**

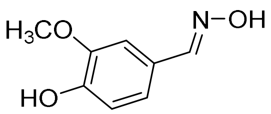

Yield: 76%. ^{1}H NMR (400 MHz, DMSO-d_6) δ 10.85 (s, 1H), 9.36 (s, 1H), 7.99 (s, 1H), 7.16 (d, *J* = 1.8 Hz, 1H), 6.97 (dd, *J* = 8.1, 1.8 Hz, 1H), 6.77 (d, *J* = 8.1 Hz, 1H), 3.77 (s, 3H). ^{13}C NMR (101 MHz, DMSO-d_6) δ 148.1, 148.0, 147.8, 124.4, 120.5, 115.4, 109.1, 55.4.

1.2.3. Preparation of 1,3-diphenylprop-2-en-1-one oxime 24a

The compound was prepared according to the literature procedure with slight modification.[3]

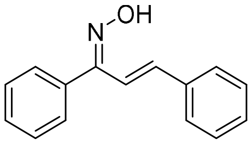

A mixture of chalcone (0.208 g, 1 mmol), $NH_2OH\cdot HCl$ (1.2 equiv.) and pyridine (0.18 mL) in MeOH (10 mL) was stirred at room temperature for 5 days. Solvent was evaporated in vacuum (50°C, 30 mbar). The residue was washed with purified by gradient flash column chromatography with hexane:ethylacetate = 5:1 as the initial eluent followed by washing with ethylacetate. The product was recovered after evaporation of ethylacetate and drying in vacuum (55°C, 10^{-2} mbar). The product was obtained as mixture of isomers ca. 1:0.6. ^{1}H NMR (400 MHz, Chloroform-*d*) δ 7.67 (d, *J* = 16.4 Hz, 1H), 7.58 – 7.34 (m, 10H), 7.06 (d, *J* = 16.6 Hz), 6.90 (d, *J* = 16.4 Hz), 6.76 (d, *J* = 17.0 Hz).

1.3. Synthesis of benzimidoyl chlorides

N-hydroxybenzimidoyl chloride

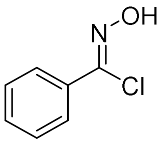

N-chlorosuccine imide (0.49 g, 4.1 mmol) was added in portions to a stirred solution of benzaldehyde oxime **2a** (0.54 g, 4.1 mmol) in DMF (8 mL). The reaction mixture was stirred at room temperature for 24 h. Water (30 mL) was added to the solution and the product was extracted with CH_2Cl_2 (3x5 mL). CH_2Cl_2 solutions were combined, washed with water (2x5 mL), dried over anhydrous Na_2SO_4 and concentrated in vacuum (+50°C, 150 mbar). The oily residue was dried in vacuum ($2\cdot10^{-5}$ bar, +40°C). Yield: 610 mg, 97%. ^1H NMR (400 MHz, Chloroform-*d*) δ 8.47 (br s, 1H), 7.87 – 7.83 (m, 2H), 7.47 – 7.38 (m, 3H).

3-Fluoro-*N*-hydroxybenzimidoyl chloride

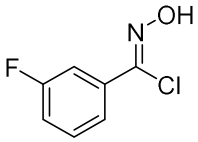

N-chlorosuccine imide (96 mg, 0.72 mmol) was added to a stirred solution of 3-fluorobenzaldehyde oxime **1a** (100 mg, 0.72 mmol) in DMF (1.5 mL). The reaction mixture was stirred at room temperature for 3 h. Water (10 mL) was added to the solution and the product was extracted with CH_2Cl_2 (3x5 mL). CH_2Cl_2 solutions were combined, washed with water (3x10 mL), dried over anhydrous Na_2SO_4 and concentrated in vacuum (+50°C, 150 mbar). The oily residue was washed with hexane (3x2 mL) and dried in vacuum ($5\cdot10^{-5}$ bar, +40°C). Yield: 120 mg, 96%. ^1H NMR (400 MHz, Chloroform-*d*) δ 7.65 (ddd, *J* = 7.9, 1.6, 1.0 Hz, 1H), 7.56 (ddd, *J* = 9.9, 2.6, 1.7 Hz, 1H), 7.38 (td, *J* = 8.1, 5.8 Hz, 1H), 7.15 (tdd, *J* = 8.3, 2.6, 0.9 Hz, 1H).

1.4. Synthesis of benzonitrile oxide

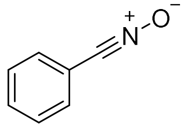

Et_3N (10 µL, 72 µmol) was added to a solution of *N*-hydroxybenzimidoyl chloride (4 mg, 26 µmol) in $CDCl_3$ (0.5 mL) in NMR tube. The solution was briefly agitated and ^1H NMR spectrum was acquired. ^1H NMR (400 MHz, Chloroform-*d*) δ 7.52 – 7.45 (m, 3H), 7.45 – 7.37 (m, 2H).

1.5. Synthesis of 3-(3-fluorophenyl)-5-phenyl-1,2,4-oxadiazole from 3-fluoro-N-hydroxybenzimidoyl chloride

N,*N*-Diisopropylethylamine (39 µL, 0.23 mmol) was added to a stirred solution of 3-fluoro-*N*-hydroxybenzimidoyl chloride (19.7 mg, 0.11 mmol) in benzonitrile (3 mL). The solution was stirred at room temperature in dark for 24 h. Benzonitrile was evaporated under reduced pressure (+50°C, $5\cdot10^{-5}$ bar). The residue was dissolved in $CDCl_3$ and the yield of the 1,2,4-oxadiazole was calculated from the ^1H NMR spectrum.

1.6. Synthesis of the photocatalysts

1.6.1. Synthesis of K-PHI

A blend of potassium chloride (2.75 g), lithium chloride (2.25 g), and 5-aminotetrazole mohonydrate (1.21 g) was grinded in a ball mill at a frequency 25 Hz for 5 min. The flour-like powder was transferred to the porcelain crucible and heated under nitrogen flow (15 L min^{-1}) using the following program: 1) heating from room temperature to 550 °C for 4 h, 2) calcination at 550 °C for 4 h. The crucibles were spontaneously cooled to room temperature. The cake and deionized water (100 mL) were brought together in a beaker and stirred at room temperature for 3 h. Solid was separated by centrifugation (4000 min^{-1}, 15 min) followed by washing with water (3x2 mL) and drying in vacuum (20 mbar) at 55 °C for 15 h.

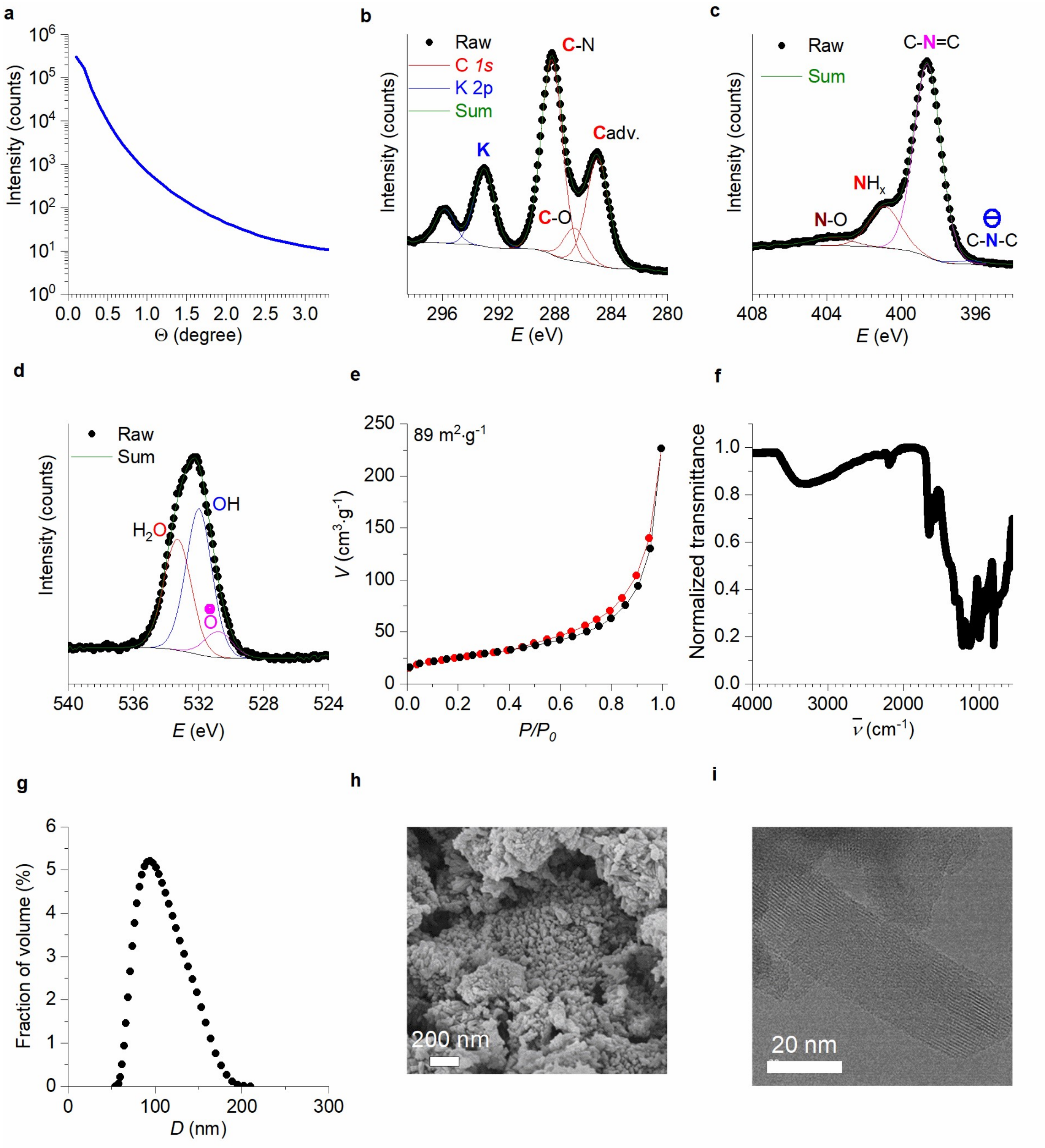

Figure S1.K-PHI characterization.[1]

a) Small angle X-Ray scattering pattern of K-PHI; b) XPS C 1s and K 2p spectra of K-PHI; c) XPS N 1s spectrum of K-PHI; d) XPS O 1s spectrum of K-PHI; e) N2 sorption isotherm measured at 77 K. BET surface area; f) FT-IR spectrum of K-PHI; g) DLS analysis of K-PHI suspension in water; h) representative secondary electrons scanning electron microscopy image of K-PHI; i) HRTEM image of K-PHI.

1.6.2. Synthesis of mpg-CN

Cyanamide (3.0 g) and Ludox HS-40 (7.5 g) were mixed in a 10 mL glass vial. The mixture was stirred at room temperature for 30 min until cyanamide has completely dissolved. The resultant solution was stirred at +60°C for 16 h until water has completely evaporated. Magnetic stir bar was removed and white solid was transferred to the porcelain crucible and

heated under N_2 flow in the oven. The temperature was increased from room temperature to 550°C within 4 h and maintained at 550°C for 4 h. The crucible was spontaneously cooled to room temperature. The solid from the crucible was briefly grinded in the mortar and transferred to the polypropylene bottle. A solution of $(NH_4)HF_2$ (0.24 g·mL^{-1}, 50 mL) was added and suspension was stirred at room temperature for 24 h. The solid was filtered, thoroughly washed with water, once with ethanol and dried in vacuum (55°C, 20 mbar) overnight.

1.6.3. Synthesis of RFT

RFT was prepared according to the literature procedure.[4]

2. Light source

In this work the following light sources were used: blue LED module with regulated irradiance (declared input power 50 W, emission maximum λ = 461 nm, measured irradiance 88 $mW \cdot cm^{-2}$); white LED module (declared input power 50 W, measured irradiance 106 $mW \cdot cm^{-2}$). Emission spectra are shown in Figure 2. Irradiance of the LED modules was measured using PM400 Optical Power and Energy Meter equipped with the integrating sphere S142C and purchased from Thorlabs.

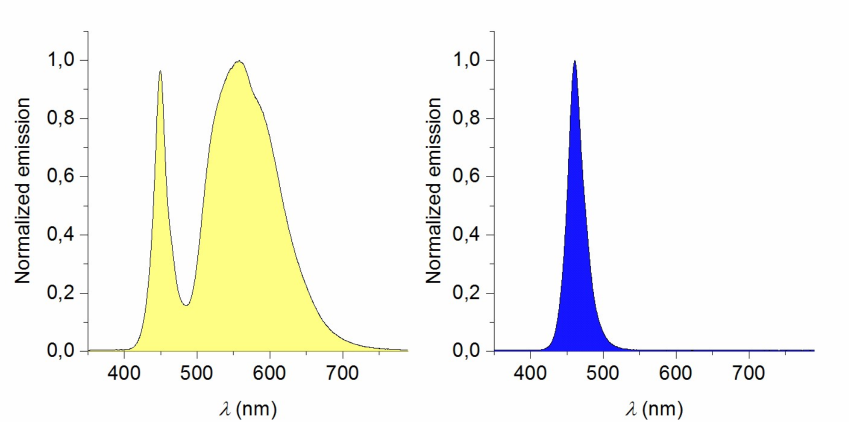

Figure S2. Emission spectra of LEDs used in this study.

3. Supplementary methods

3.1. NMR

^{1}H and ^{13}C NMR spectra were recorded on Agilent 400 MHz (at 400 MHz for Protons and 101 MHz for Carbon-13). Chemical shifts are reported in ppm versus solvent residual peak: chloroform-*d* 7.26 ppm (^{1}H NMR), 77.16 ppm (^{13}C NMR); DMSO-d_6 2.50 ppm (^{1}H NMR), 39.52 ppm (^{13}C NMR).

3.2. GC-MS

Agilent 6890 Network GC System coupled with Agilent 5975 Inert Mass Selective detector (electron ionization) were used for reaction mixture composition analysis and to obtain mass spectra of the products.

3.3. High-resolution mass spectra

The data were obtained using Waters XEVO G2-XS QTOF with Aquity H-Class (HPLC).

3.4. TEM investigation of K-PHI

For high-resolution transmission electron microscopy (HRTEM) observations, a suspension of the sample in ethanol was sonicated for 10 minutes and then drop-casted to a Cu grid with a holey carbon support and dried for 10 minutes. The TEM study was performed using a double Cs corrected JEOL JEM-ARM200F (S)TEM operated at 80kV equipped with a cold-field emission gun.

3.5. X-Ray powder diffraction (XRD) of K-PHI

XRD patterns for Rietveld refinement were collected at room temperature on a transmission STADI-P (STOE, Germany) diffractometer equipped with a linear mini-PSD detector using Cu Kα1 radiation in the 2θ range 2° - 120° with a step of 0.03°. Polycrystalline silicon (a = 5.43075(5) Å) was used as an external standard. The GSAS program package[5],[6] was used for structure refinement from powder data.

3.6. K-PHI XRD pattern fitting

The peak profiles were fitted with a pseudo-Voigt function, $I(2\theta) = x*L(2\theta) + (1-x)*G(2\theta)$ (where L and G are the Lorentzian and Gaussian part, respectively). The angular dependence of the peak width was described by the relation $(\mathrm{FWHM})^2 = \mathrm{U}tg^2\theta + \mathrm{V}tg\theta + \mathrm{W}$, where FWHM is the full line width at half maximum. During refinement an additional type of anisotropic broadening function was introduced to describe two classes of reflections with different size anisotropy. The necessity to apply this function points towards the presence of stacking faults mentioned above. The background level was described by a 36-order Chebyshev polynomial. The absorption correction function for a flat plate sample in transmission geometry has been applied.

Table S1. Structural data for K-PHI

Space group, #	*P*31*m*, 157
The number of formula units, *Z*	1
Cell constants:	
a=*b*, Å	12.637(3)
c, Å	3.2998(3)
V, $Å^3$	456.4(2)
*D*x, g cm^{-3}	1.88
wRp, %	3.48
Rp, %	2.60
R(F^2), %	5.25
χ^2	8.142

Table S2. Atomic coordinates and isotropic thermal parameters for K-PHI.

Atom	x/*a*	y/*b*	z/*c*	Fraction	U_{iso} ($Å^2$)
N(1)	0.685(2)	0	0	1.000	0.144(2)
N(2)	0.572(8)	0.122(1)	0	1.000	0.144(2)
N(3)	0.448(1)	0.229(1)	0	1.000	0.144(2)
N(4)	1/3	2/3	0	1.000	0.144(2)
C(1)	0.670(2)	0.102(1)	0	1.000	0.144(2)
C(2)	0.556(1)	0.2238(4)	0	1.000	0.144(2)
K(1)*	0.121(2)	0.121(2)	0.860(2)	0.7032	0.144(2)
K(2)*	0.252(2)	0	0.421(2)	0.2064	0.144(2)
K(3)*	0.229(2)	0	0.835(2)	0.2551	0.144(2)

* It is important to mention that the K positions have multiplicity 3, and with partial occupancy they give a total number of K atoms in the unit cell of about 3.5 (which is higher than expected); however, these positions just show possible positions for K atoms, with the fractions showing the probabilities of these positions to be occupied.

Although a structure solution has been presented earlier,[7] we analyzed the structure of the samples that consists of smaller crystallites (10-20 nm) with the highest catalytic activity, based only on a general assumption of the structure independently from the existing publication. We performed a Fourier map analysis to locate K atoms in the structure, and a Rietveld refinement taking into account information about defects obtained from HR-TEM. We believe that the crystal structure of K-PHI is prone to the formation of stacking faults, which shows that in the given synthesis conditions different polytypes with approximately similar energies can form. This is why we believe that describing the nanocrystalline sample based on the model obtained for bigger crystals (as it is done in reference [7]) restricts the diversity to only one specific polytype.

The standard deviation for the lattice parameters found from TEM is very high, clearly indicating that there is variation in the local structure. The accuracy of measurements is

lower than usual since tilting of small crystallites to the exact orientation due to their size and their high sensitivity to the beam was not possible. In some cases small deviations in focus and exact orientation can occur, which influences the average parameters calculated from FFT transforms.

The ratio between the intensities of the 110 peak at about 14 degrees and the 001 peak at 26 degrees is mainly influenced by the distribution of potassium atoms in the structure. In our structure, potassium atoms tend to sit closer to the center of the triangular pore so that they can form bonds with bridging nitrogen atoms. Such configuration allows to describe the peak intensities in the best way. However, in the structure refinements from Lotsch et al. the best intensity description (as they show in Figure S30) was obtained when potassium ions are consistently moved off center, in-plane, toward one corner of the triangular pore.[7]

Although a high water content has been reported for K-PHI,[7] analysis of the TG curve of the sample used in this study indicates the presence of about 12wt.% of adsorbed water in the sample (Figure S9), which is released at temperatures below 200°C, and about 3-4wt.% of water that bounded strongly. Thus we believe that only about one third of K atoms forms complexes with water. The presence of a few % of strongly bounded water might explain why we obtained a slightly high content of K per unit cell.

3.7. Computational details

Spin-polarized density functional theory (DFT) calculations were performed using the Vienna *Ab initio* Simulation Package (VASP).[8],[9],[10] We employed the generalized gradient approximation (GGA) functional PBE whereas the electron-ion interactions were modelled using the projector-augmented wave (PAW) method.[11],[12] Dispersion van der Waals corrections were included using the empirical (DFT-D3) approach.[13] In the DFT calculations, a cut-off energy of 500 eV was used for the plane wave basis set. To examine the electronic structures, Brillouin zone was sampled by using K points mesh of 2×2×5 for structural relaxation and a thicker mesh of 4×4×7 for obtaining the density of states under Monkhorst–Pack scheme.[14] For the geometry optimization and singlet-triplet energies, we used a unit cell of K-PHI having 32 atoms in total (C = 12, N = 17, K = 3). All the systems were optimized until the convergence and force criteria of 10^{-6} eV and 0.01 eV/Å, respectively were reached.

To reduce computation costs for calculations we took the K-PHI structure bearing three K(1) potassium atoms (those with a fraction 0.7032 in Table S2, brown spheres on Figure 1). It has been reported that K-PHI contains water in pores.[7] In this study we did not include crystallization water, due to low content of the latter, as deduced from the TGA curve (3 wt. %, Figure S9).

As evidenced by the K-PHI crystal structure refinement, models with different CN-layer stackings, in particular *ccp* (ABCABC), *hcp* (ABAB) and mixed stacking (AABB, etc.) allowed to partially describe the positions and the broadening of the peaks, but did not give a better description of the XRD pattern, suggesting the presence of stacking faults rather than a

second structural modification in the sample. Therefore, AAA stacking forming continuous channels along the *c* direction has been chosen to build a model for further calculations.

Zero on the X-axis of the DOS plots corresponds to the Fermi energy (E_F). The values are given versus NHE. Conduction band minimum (CBM) was determined as the interception of the tangent to the TDOS curve in the linear region (-1.07…-0.89 eV) and the X-axis. Valence band maximum (VBM) was determined as the interception of the tangent to the TDOS curve in the linear region (+2.76…+2.94 eV) and the X-axis. Onset of IBS on the side pointing towards the CBM was determined as the interception between the tangent to the TDOS curve in the linear region (+1.11…+1.34 eV) and the X-axis. Onset of IBS on the side pointing towards the VBM was determined as the interception between the tangent to the TDOS curve in the linear region (+2.13…+2.31 eV) and the X-axis.

Although, GW approximation is suggested for correcting the band structure of carbon nitrides,[15],[16] for K-PHI it would increase significantly the computational costs. Herein, the predicted CBM is -0.8 V vs NHE and VBM is +2.68 V, while determined experimentally values are -0.50 V and +2.63 V respectively that gives reasonable agreement between theory and experiment.

CBs and VBs of the singlet excited state are built by the C and N atoms (Figure S3). For the triplet excited state, both CBs and VBs are shifted by +1 V to more positive values. Dissymmetry of TDOSs is rationalized by the energy level splitting due to repelling parallel spins.

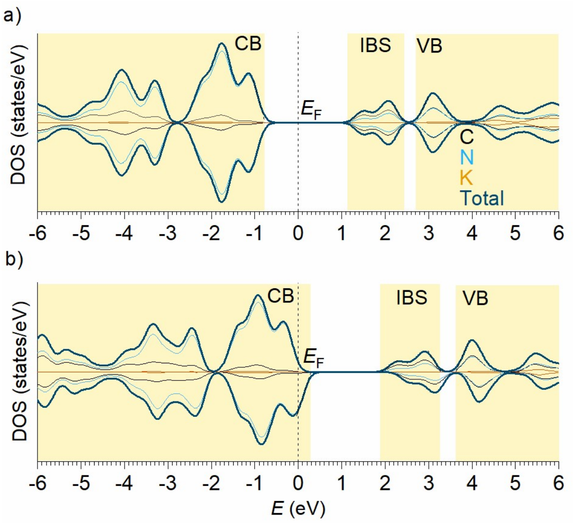

Figure S3. Partial and total DOS of singlet (S) and triplet (T) excited state of K-PHI.

3.8. Fluorescence measurements

Fluorescence of carbon nitride powder was measured on Jasco FP-8300 fluorescence spectrometer. The response time has been set to 0.8 s. The excitation wavelength was set to 360 nm. Internal Quantum Efficiency (IQE) of fluorescence was determined using integrating sphere. To ensure sufficient signal intensity, K-PHI powder was analysed in neat.

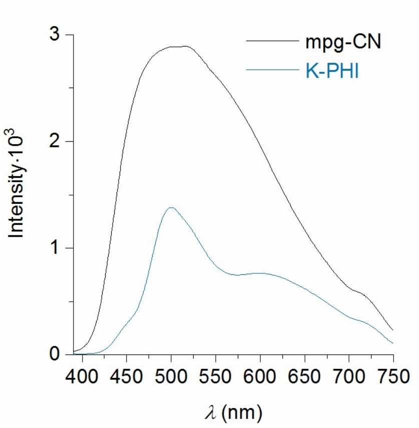

Figure S4. Fluorescence spectra of mpg-CN and K-PHI.

Table S3. Contribution of the peaks to the total fluorescence.

Entry	Peak center, eV	Area	Relative area, %
1	2.76	0.03124	5.0
2	2.53	0.09542	15.2
3	2.43	0.20037	31.9
4	2.05	0.29947	47.7
5	1.72	0.00135	0.2

3.9. Phosphorescence measurements

Phosphorescence of carbon nitride powder was measured on Jasco FP-8300 fluorescence spectrometer in phosphorescence measurement mode. The following parameters were used: excitation wavelength 360 nm, chopping period 25 ms, delay 10 ms, integration time 5 ms, response 2.5 s. To ensure sufficient signal intensity, K-PHI powder was analysed in neat.

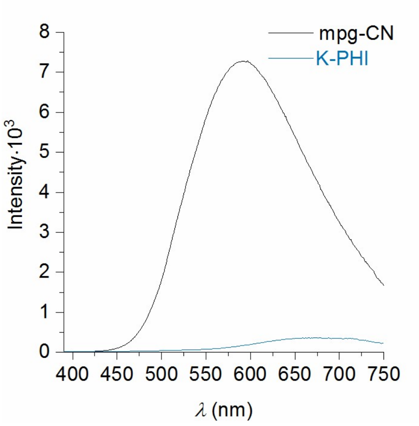

Figure S5. Phosphorescence spectra of mpg-CN and K-PHI.
Spectra were recorded applying chopping period 25 ms. All spectra were recorded upon sample excitation with 360 nm.

3.10. Transient absorption spectroscopy (TAS)

Femtosecond transient absorption studies were carried out using a Helios transient absorption pump/probe system from Ultrafast Systems with laser pulses fed by 387 nm laser pump pulses (1 kHz, 150 fs pulse width) from an amplified Ti:sapphire laser system (CPA-2101) from Clark-MXR Inc. All spectra were recorded in 2 mm quartz (OS) cuvettes at ambient conditions while continuous stirring during the measurements. Lifetimes were obtained by multi-wavelength analysis of the transient absorption spectra. Five-exponential fitting functions were used to adequately fit the time-absorption profiles.

To ensure comparison of TAS measurements results with the results of photocatalytic reactions, the conditions were kept as close as possible to that in the photocatalytic experiments. Namely, TAS measurements were performed suspending K-PHI in MeCN (concentration 1.7 mg mL^{-1}) without degassing the suspension to account for possible O_2 influence. A quartz cuvette with optical path 0.2 cm was used for measurements. The data was fitted with five-exponential fitting function to achieve R^2-coefficient close to unity.

TAS measurements also suggest that K-PHI is chemically pure since we observe the same deactivation behavior throughout the analyzed spectral range, i.e. five lifetimes were observed at all wavelengths. Contributions of other chemical species (inhomogeneities) would be expected to give rise to additional excited state features in the spectra.

To confirm the material-intrinsic nature of the observed spectroscopic features, we compared measurements in the presence and absence of molecular oxygen, a triplet quencher, as well as in the presence of benzylamine, an electron donor, and essentially no significant difference in the excited state dynamics was observed (Figure S6).

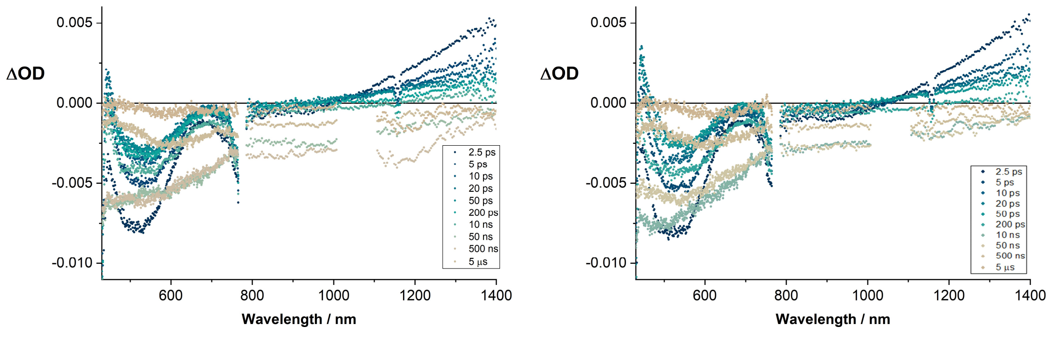

Figure S6. Transient absorption spectra of a K-PHI.
Spectra were acquired using a suspension of K-PHI in MeCN (1.7 mg mL^{-1}) acquired using λ_{ex} = 387 nm in the presence of molecular oxygen (left) and benzylamine (right).

The measurement of particulate species in the reaction medium requires high-quality dispersions. The quality of mpg-CN suspension even after prolonged sonication was too poor to obtain reasonable data. Much better stability of colloidal solution of K-PHI is explained by smaller particle size and negative zeta-potential.[17]

In the tested pump fluence range between 1.3 and 2.9 J cm^{-2}, no significant change in the deactivation behavior was found.

3.11. Singlet-oxygen fluorescence measurements

Fluorescence measurements were conducted on a Floruolog 3 (Horiba Scientific) equipped with an InGaAs symphony as the detection unit using mass concentrations was 0.1 g L-1 for mpg-CN and 0.01 g L-1 for K-PHI in MeCN. The optical density at 320 nm was 0.35.

To ensure maximum fluorescence intensity, a suspension was purged with O_2 prior measurements.

3.12. Cyclic voltammetry

Electrochemical measurements were performed in three electrode cell equipped with the Ar inlet and magnetic stir bar using Biologic potentiostat to control the potential of the working electrode (WE) and EC-Lab v. 10.40 software for data logging. Glassy carbon (diameter 3 mm) was used as a WE, Ag wire in $AgNO_3$ (0.01M) with tetrabutylammonium perchlorate (0.1M) in MeCN as a reference electrode (RE), Pt wire as a counter electrode. Measurements were performed at room temperature (20-25°C). A solution of tetrabutylammonium perchlorate (0.1M) in MeCN was used as electrolyte. The electrochemical cell was placed in the grounded Faraday's cage in order to reduce noise.

3.12.1. CV of oximes under Ar atmosphere

Electrochemical cell was filled with the electrolyte (10 mL). Solution was stirred while dry argon was passed through the solution for ca. 10 min at the rate ca. 5 mL·min^{-1}. Cycling voltammetry (CV) curves were acquired after every 2-3 min in order to confirm absence of the redox peak related to the $O_2/O_2^{\bullet-}$ redox couple centred at ca. −1.19 V vs. RE. CV measurements were performed without stirring, while flow of argon was by-passed above the solution. When no apparent redox peaks at ca. −1.19 V vs RE were observed, oxime (20 µmol) was added to the electrolyte. The mixture was briefly stirred in order to dissolve the compound. CV curves were acquired scanning continuously the region from 0 V to +2 V, from +2 V to −2 V and back from −2 V to 0 V. Typically 3 scans were acquired. The scanning rate was 50 mV·s^{-1}. After the CV measurement was finished, ferrocene (3.72 mg, 20 µmol) was added to the electrolyte. The solution was stirred for ca. 2 min in order to completely dissolve ferrocene. CV measurements were repeated scanning the region from +0.5 V to −0.5 V at the scanning rate 50 mV·s^{-1}. The potential of the WE was then recalculated using the equation:

$$U_{corr} = U_{WE} - E_{Fc/Fc^{+}}$$

where U_{WE} – potential of the WE versus RE, V; E_{Fc/Fc^+} – the redox potential of the Fc/Fc^+ couple versus the RE. Determined as an average between reduction and oxidation peaks of the Fc/Fc^+, V.

3.13. Time-dependent AQY study

A glass reactor equipped with a magnetic stir bar, cold finger and inlet for O_2 supply was charged with benzaldehyde oxime (16.8 mg, 135 μmol), K-PHI (13.5 mg) and acetonitrile (8.1 mL). The reactor headspace was connected to the O_2 balloon (ca. 1 bar, absolute pressure) and covered with a black screen. The reaction mixture was vigorously stirred under blue light irradiation (461 nm, 88 mW·cm^{-2}). Light was delivered to the reaction mixture through the rectangular hole (2.2 cm^2) in the screen. Temperature of the reaction mixture was maintained at +35°C. Samples (0.3 mL) of the reaction mixture were taken at certain times, concentrated in vacuum (30 mbar, +50°C). $CDCl_3$ was added to the residue and suspension was quantitatively transferred into the NMR tube. 1,1,2,2-dichloroethane (0.5 μL) was added to the NMR tube. The yield (μmol) of the 1,2,4-oxadiazole was calculated from the ^{1}H NMR. AQY was calculated as a ratio of the 1,2,4-oxadiazole yield per photon emitted to the reactor during the measured period of time.

3.14. Kinetic Isotope Effect determination

Primary Kinetic Isotope Effect (competition reaction). The experiments were performed in triplicate. Average KIE and standard deviation are reported. A glass tube was loaded with benzaldehyde oxime **2a** (3 mg, 25 μmol), benzaldehyde oxime-d_1 **2a**-d_1 (3 mg, 25 μmol), K-PHI (5 mg) and MeCN (3 mL). After addition a magnetic stir bar, the tube was closed with a rubber septum. An air balloon (ca. 1 bar, absolute pressure) was connected to the reactor head space. The reaction mixture was stirred under blue light irradiation (461 nm, 88 mW·cm^{-2}) for 8 h. The catalyst was separated by centrifugation (13000 rpm, 5 min) and washed with MeCN (2 x 2 mL). The solutions were combined in a flask and concentrated in vacuum (+50°C, 150 mbar). The residue was dissolved in $CDCl_3$ and quantitatively transferred into the NMR tube. 1,1,2,2-tetrachloroethane (2 mg, 12 μmol), internal standard, was added to the NMR tube and a solution was analysed by ^{1}H NMR. Primary KIE was calculated as a ratio of consumed benzaldehyde oxime to benzaldehyde oxime-d_1.

Secondary Kinetic Isotope Effect (competition reaction). The experiments were performed in triplicate. Average KIE and standard deviation are reported. A glass tube was loaded with benzaldehyde oxime **2a** (6 mg, 50 μmol), K-PHI (5 mg), MeCN (393 mg, 9.6 mmol) and CD_3CN (422 mg, 9.6 mmol). After addition a magnetic stir bar, the tube was closed with a rubber septum. An air balloon (ca. 1 bar, absolute pressure) was connected to the reactor head space. The reaction mixture was stirred under blue light irradiation (461 nm, 88 mW·cm^{-2}) for 8 h. The catalyst was separated by centrifugation (13000 rpm, 5 min) and washed with MeCN (2 x 2 mL). The solutions were combined in a flask and concentrated in vacuum (+50°C, 150 mbar). The residue was dissolved in $CDCl_3$ and quantitatively transferred into the NMR tube. 1,1,2,2-tetrachloroethane (2 mg, 12 μmol), internal standard, was added to the NMR tube and a solution was analysed by ^{1}H NMR. The

secondary KIE was calculated as a ratio of 5-methyl-3-phenyl-1,2,4-oxadiazole to 5-(methyl-d3)-3-phenyl-1,2,4-oxadiazole.

3.15. EPR study

EPR study was conducted on Bruker EMXnano benchtop X-Band EPR spectrometer. The following settings have been used for all spectra acquisition unless other is specified: Center Field 3444.05 G, Sweep Width 200 G, Receiver Gain 60 dB, Modulation Amplitude 1.000 G, Number of Scans 1, Microwave Attenuation 10 dB.

3.15.1. Photocatalytic TEMPO generation.

A glass tube with an inlet for gas connection and a ground joint for 'cold finger' connection equipped with a magnetic stir bar (5x10 mm) was charged with a mixture of K-PHI (5 mg) and 2,2,6,6-tetramethylpiperidine (8.5 μL) in MeCN (3 mL). The mixture was vigorously stirred at room temperature for 10 min. A capillary (IntraMark, volume 50 μL, purchased from BRAND GMBH + CO KG) was sealed in the flame of gas burner from one side. The capillary was charged with a reaction mixture (40 μL). The capillary was placed into an EPR tube (ID 3 mm, OD 4 mm, length 250 mm). EPR spectrum was acquired and used as a reference (0 min, in dark).

The reaction mixture was briefly purged with O_2. Cold finger was immersed into the suspension and cooling water circulation was enabled maintaining the reaction mixture temperature at 20-25°C. A balloon with O_2 was connected to the reactor headspace *via* gas inlet. The reaction mixture was vigorously stirred under blue light irradiation (430 mW cm^{-2}) for a specified time. Aliquot (40 μL) was transferred into the capillary and EPR spectrum acquisition was repeated using the same settings as for the reference measurement.

3.15.2. An attempt of DMPO-$O_2^{\bullet-}$ adduct detection.

A glass tube with an inlet for gas connection and a ground joint for 'cold finger' connection equipped with a magnetic stir bar (5x10 mm) was charged with a freshly prepared solution of DMPO (0.017 M, 3 mL) in MeCN followed by addition of K-PHI (5 mg). The mixture was vigorously stirred at room temperature for 10 min and briefly purged with O_2. A capillary (IntraMark, volume 50 μL, purchased from BRAND GMBH + CO KG) was sealed in the flame of gas burner from one side. The capillary was charged with a reaction mixture (40 μL). The capillary was placed into an EPR tube (ID 3 mm, OD 4 mm, length 250 mm). EPR spectrum was acquired and used as a reference (0 min, in dark).

'Cold finger' was immersed into the suspension and cooling water circulation was enabled maintaining the reaction mixture temperature at 20-25°C. A balloon with O_2 was connected to the reactor headspace via gas inlet. The reaction mixture was vigorously stirred under blue light irradiation (430 mW cm^{-2}) for a specified time. Aliquot (40 μL) was transferred into the capillary and EPR spectrum acquisition was repeated using the same settings as for the reference measurement (Figure S7).

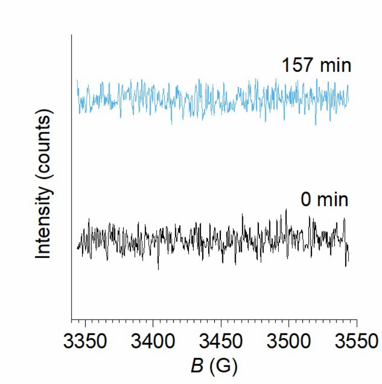

Figure S7. An attempt of DMPO-$O_2^{\bullet-}$ adduct detection by EPR.

3.16. Photocatalysts TON calculation of oxime 1a conversion

TON of homogeneous photocatalysts was calculated as a ratio between a number of oxime **1a** molecules and a number of the photocatalysts molecules:

$$TON = \frac{n_{oxime}}{n_{PC}}$$

TON of K-PHI was calculated using following assumptions:

1. K-PHI particles are approximated as spheres of diameter *D;*

2. Particle size distribution data was taken from dynamic light scattering measurements (Figure S1g) in order to take into account dissymmetry of particle size distribution, *i.e.* larger volume fraction of bigger particles;

3. One K-PHI crystal cell is considered as one catalytic centre;

4. Crystal cells on the surface of K-PHI particles are considered to catalyse oxime **1a** conversion;

5. TON was calculated using equation

$$TON = \frac{S_{cell}}{6 \cdot 10^{21} \cdot \frac{m_{K-PHI}}{d_{K-PHI}} \cdot \sum \frac{v_i}{D_i}} \cdot n_{oxime} \cdot N_A = 305$$

where S_{cell} – surface of the K-PHI cell base (1.383 nm^2); m – K-PHI mass in a typical experiment (0.005 g), d_{K-PHI} – K-PHI density (1.88 g·cm^3); v_i – volume fraction of K-PHI particles of diameter D_i (derived from data in Figure S1g, shown in Figure S8); n_{oxime} – number of oxime molecules (5·10^{-5} mol), N_A – Avogadro constant (6.02·10^{-23} mol^{-1}).

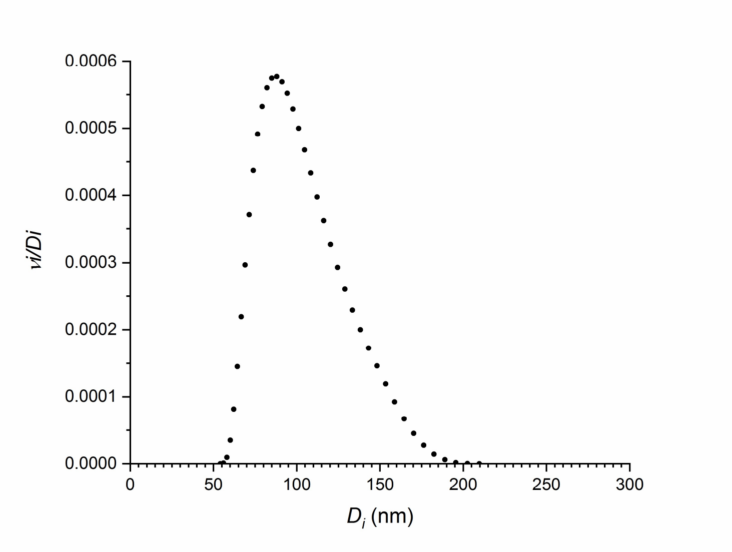

Figure S8. Dependence of v_i/D_i versus D_i.

3.17. K-PHI characterization methods

3.17.1. Fourier transform infrared (FT-IR) spectra were recorded on Thermo Scientific Nicolet iD5 spectrometer.

3.17.2. Nitrogen adsorption/desorption measurements were performed after degassing the samples at 150 °C for 20 hours using a Quantachrome Quadrasorb SI-MP porosimeter at 77.4 K. The specific surface areas were calculated by applying the Brunauer-Emmett-Teller (BET) model to adsorption isotherms for $0.05 < p/p0 < 0.3$ using the QuadraWin 5.11 software package.

3.17.3. Scanning electron microscopy (SEM) images were obtained on a LEO 1550-Gemini microscope.

3.17.4. The X-ray photoelectron spectroscopy (XPS) measurements were carried out in an ultrahigh vacuum (UHV) spectrometer equipped with a VSW Class WA hemispherical electron analyzer. A dual anode Al Kα X-ray source (1486.6 eV) was used as incident radiation. Survey and high resolution spectra were recorded in constant pass energy mode (44 and 22 eV, respectively). During the UPS (He I excitation energy $h\nu$=21.23 eV) measurements a bias of 15.32 V was applied to the sample, in order to avoid interference of the spectrometer threshold in the UP spectra. Zero on the X-axis defines Fermi energy (E_F). The VBM was determined as the interception between the tangent to the curve in the linear region (+4.00…+3.13 eV) and the baseline. IBS onset was determined as the interception between the tangent to the curve in the linear region (+2.59…+1.48 eV) and the baseline. The baseline was defined as a straight line passing through the linear region (-1.17…+1.38 eV) of the curve. The relationship between the NHE scale and vacuum scale is defined by the equation:

$$E_{vac} = -4.50 - E_{NHE}$$

3.17.5. Optical absorbance spectra of powders were measured on a Shimadzu UV 2600 equipped with an integrating sphere. The optical band gap (π-π* transitions) has been determined as the interception of the tangent to the linear part of curve (range of photon energies 2.75-2.85 eV) and X-axis. Onset of n-π* transitions has been determined as the interception of the tangent to the linear part of the curve (range of photon energies 2.00-2.18 eV).

Extended optical absorption in the near IR is explained mainly by the properties of K-PHI itself rather than the presence of tentative second phase. As evidenced by the XRD pattern fitting study, K-PHI has polytypes. On the other hand, calculated XRD pattern fits well to the experimental one (wRp = 3.48%). Therefore, we can conclude that contribution of the tentative, second phase, is below 4%. Of course, assuming that it is crystalline. However, we cannot exclude completely the presence of the tentative amorphous phase that is not detected by X-Ray.

3.17.6. Emission spectra were recorded on Jasco FP-8300 instrument. The excitation wavelength was 360 nm.

3.17.7. The TEM measurements were acquired using a double-corrected Jeol ARM200F, equipped with a cold field emission gun and a Gatan GIF Quantum. The used acceleration voltage was 200kV and the emission was set to 10μA in order to reduce beam damage. An objective aperture with a diameter of 60μm was introduced into the beam to improve the contrast while still allowing for atomic resolution.

3.17.8. Dynamic light scattering (DLS) measurements were performed using Nano-ZS Zetasizer ZEN3500 (Malvern Instruments LTD).

3.17.9. Thermogravimetric analysis (TGA) was performed on NETZSCH TG209 F1 Libra. The sample was heated in Pt crucible under flow of nitrogen (5 mL min^{-1}) from 28°C to 1000°C.

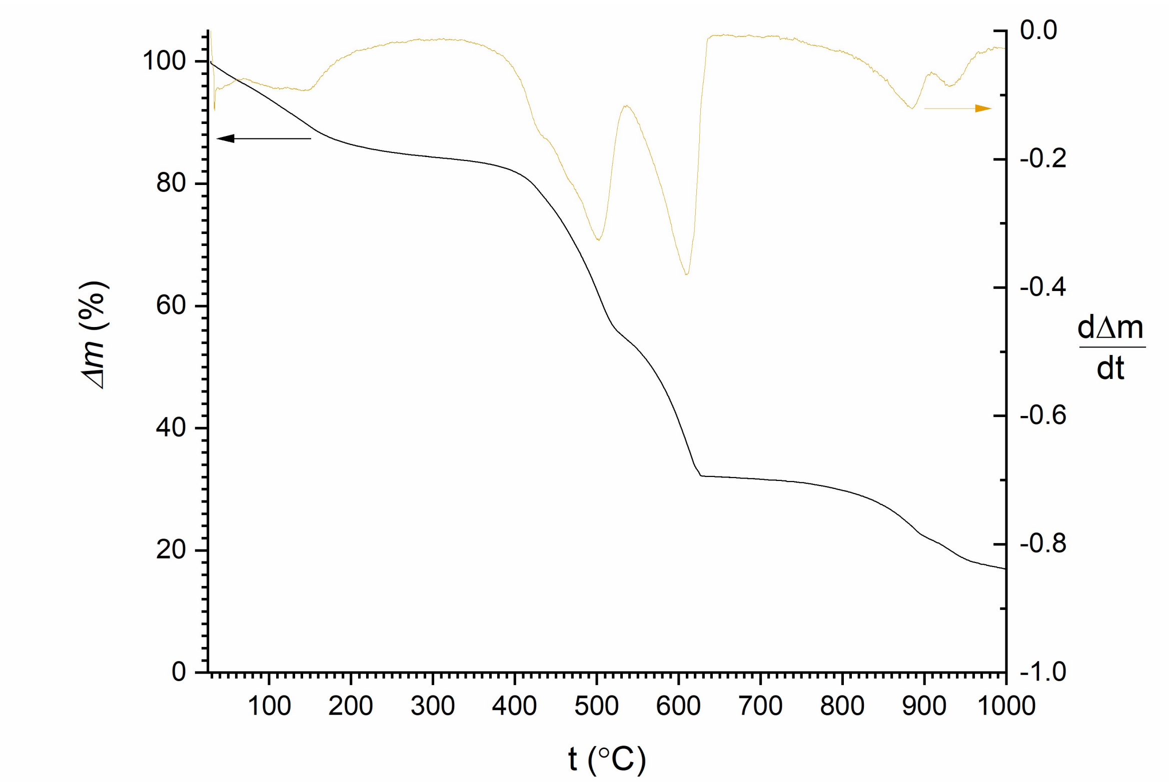

Figure S9. TGA of K-PHI and first derivative.

3.17.10. Band structure

The conduction band minimum (CBM, E_{CBM} = −0.5 eV) was calculated from Mott-Schottky plot and taken from the literature.[18] The valence band maximum (VBM, E_{VBM} = +2.68 eV) was determined from UPS. The difference between CBM and VBM is denoted as an energy gap (E_g = E_{VBM}-E_{CBM} = 3.18 eV). From the DRUV-vis the optical band gap was determined (E_{OG} = 2.64 eV). The optical band gap is narrower than the energy gap of the material.[19]

4. Photocatalytic experiments

4.1. Reaction of the photosensitized $^1O_2$ with 9,10-diphenylantracene

A glass tube was charged with 9,10-diphenylantracene (16.5 mg, 50 μmol), MeCN (3 mL) and catalyst. The following amount of the catalyst was used: K-PHI 5 mg, mpg-CN 5 mg, $Ir(ppy)_3$ (1.5 mg, 2.3μmol, 5 mol.%), RFT (1.3 mg, 2.3μmol, 5 mol.%), Methylene Blue·$3H_2O$ (0.7 mg, 2.3μmol, 5 mol.%), $Ru(bpy)_3Cl_2·6H_2O$ (1.7 mg, 2.3μmol, 5 mol.%), $[Mes\text{-}Acr]^+ClO_4$ (1.0 mg, 2.3μmol, 5 mol.%). Magnetic stir bar was placed in the tube. The tube was closed with the rubber septum and a balloon filled with O_2 was connected to the reaction mixture head space via needle. The reaction mixture was vigorously stirred under white light irradiation (106 mW·cm^2) for 24 h. In case of using the heterogeneous catalyst, it was separated by centrifugation, washed with MeCN (2x2 mL). MeCN solutions were combined and concentrated in vacuum. In case of the homogeneous catalyst, the reaction mixture was concentrated in vacuum. The residue after MeCN distillation was dissolved in $CDCl_3$ and analyzed by ^1H and ^{13}C NMR.

9,10-diphenyl-9,10-dihydro-9,10-epidioxyanthracene

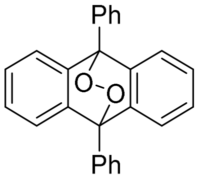

^1H NMR (400 MHz, Chloroform-*d*) δ 7.74 – 7.70 (m, 4H), 7.65 (t, *J* = 7.6 Hz, 4H), 7.58 – 7.53 (m, 2H), 7.25 – 7.18 (m, 8H). ^1H and ^{13}C NMR spectra are identical to the reported.[20]

$^1O_2$ sensitization and utilization in organic synthesis was first analyzed via the reaction of $^1O_2$ addition to 9,10-diphenylanthracene (DPA) (Table S3). Comparable yields, 75-88%, of the peroxy-adduct were obtained for K-PHI, mpg-CN and methylene blue, the 'classical' $^1O_2$ sensitizer. Among other homogeneous sensitizers $Ir(ppy)_3$ gave the peroxy-adduct in 15% yield, while $Ru(bpy)_3Cl_2$, RFT and $[Mes\text{-}Acr]^+ClO_4^-$ did not produce the peroxy-adduct, although DPA was completely consumed.

Table S4. Test reaction of singlet oxygen addition to 9,10-diphenylathracene.[a]

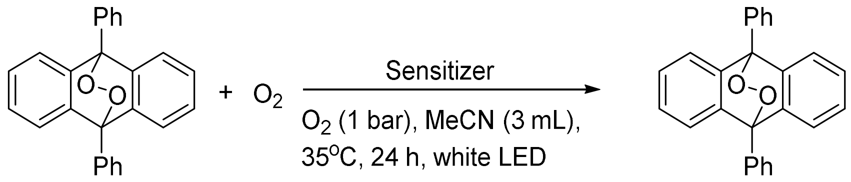

Entry	Sensitizer	9,10-diphenyl-9,10-dihydro-9,10-epidioxyanthracene yield,[b] %
1	K-PHI (5 mg)	88
2	mpg-CN (5 mg)	76
3	$Ir(ppy)_3$ (2.3 µmol)	15
4	$Ru(bpy)_3Cl_2\cdot 6H_2O$ (2.3 µmol)	Trace
5	RFT (4.6 µmol)	0
6	Methylene blue·$3H_2O$ (2.1 µmol)	75
7	$[AcrMes]^+ClO_4^-$ (4.8 µmol)	Trace
8	-	65

[a] Conditions: 9,10-diphenylathracene 50 µmol; MeCN (3 mL); O_2 1 bar; white LED (106 mW·cm[2]); T=35°C; 24 h;

[b] NMR yield

4.2. A general procedure of 1,2,4-oxadiazoles preparation using K-PHI photocatalyst

A 5 mL glass tube equipped with a magnetic stir bar was charged with oxime (50 µmol), K-PHI (5 mg) and nitrile (3 mL). The tube was covered with a rubber septum. A rubber balloon filled with air (ca. 1 bar, absolute pressure) was connected to the reaction mixture headspace via a steel needle. The reaction mixture was vigorously stirred at 35°C under blue light irradiation (461 nm, 88 mW·cm^{-2}) for 24 h. K-PHI was separated by centrifugation (13000 rpm, 3 min), dispersed in MeCN (2 mL) and separated by centrifugation (13000 rpm, 3 min). K-PHI was rinsed in total 3 times. The solutions after K-PHI washing were combined and concentrated in vacuum (30 mbar) at 50°C. In case high boiling nitrile was used as a reagent, it was distilled off in vacuum ($7\cdot10^{-5}$ bar). The residue was purified by column chromatography on silica gel using hexane:ethylacetate (19:1).

3-(3-fluorophenyl)-5-phenyl-1,2,4-oxadiazole **1**

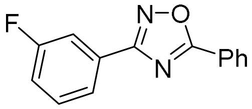

Yield 82%. ^{1}H NMR (400 MHz, Chloroform-*d*) δ 8.25 – 8.20 (m, 2H), 7.98 (dt, *J* = 7.7, 1.2 Hz, 1H), 7.89 (ddd, *J* = 9.5, 2.6, 1.5 Hz, 1H), 7.66 – 7.61 (m, 1H), 7.59 – 7.55 (m, 2H), 7.49 (td, *J* =

8.0, 5.7 Hz, 1H), 7.23 (tdd, *J* = 8.4, 2.7, 1.0 Hz, 1H). ^{13}C NMR (101 MHz, Chloroform-*d*) δ 176.1, 168.3 (d, *J* = 3.1 Hz), 163.0 (d, *J* = 246.6 Hz), 133.1, 130.7 (d, *J* = 8.1 Hz), 129.3, 129.1 (d, *J* = 8.5 Hz), 128.3, 124.2, 123.4 (d, *J* = 3.1 Hz), 118.3 (d, *J* = 21.2 Hz), 114.7 (d, *J* = 23.7 Hz). ^{19}F NMR (376 MHz, Chloroform-*d*) δ -112.0 (td, *J* = 9.0, 5.7 Hz).

3,5-diphenyl-1,2,4-oxadiazole **2**

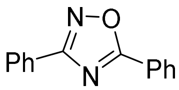

Yield 48%. ^{1}H NMR (400 MHz, Chloroform-*d*) δ 8.25 – 8.22 (m, 2H), 8.20 – 8.16 (m, 2H), 7.65 – 7.60 (m, 1H), 7.59 – 7.49 (m, 5H). ^{13}C NMR (101 MHz, Chloroform-*d*) δ 175.9, 169.1, 132.9, 131.3, 129.3, 129.0, 128.3, 127.7, 127.1, 124.4.

5-phenyl-3-(3-(trifluoromethyl)phenyl)-1,2,4-oxadiazole **3**

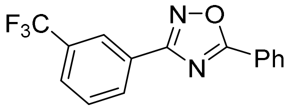

Yield 75%. ^{1}H NMR (400 MHz, Chloroform-*d*) δ 8.47 (s, 1H), 8.38 (d, *J* = 7.8 Hz, 1H), 8.26 – 8.22 (m, 2H), 7.79 (d, *J* = 7.8 Hz, 1H), 7.68 – 7.62 (m, 2H), 7.61 – 7.55 (m, 2H). ^{13}C NMR (101 MHz, Chloroform-*d*) δ 176.3, 168.1, 133.2, 131.6 (q, *J* = 32.9 Hz), 130.8 (d, *J* = 1.0 Hz), 129.6, 129.3, 128.4, 128.0, 127.9 (q, *J* = 3.5 Hz), 124.7 (q, *J* = 3.8 Hz), 124.11, 123.9(q, *J* = 272.0 Hz). ^{19}F NMR (376 MHz, Chloroform-*d*) δ -62.8.

3-(4-fluorophenyl)-5-phenyl-1,2,4-oxadiazole **4**

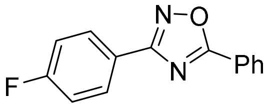

Yield: 49%. ^{1}H NMR (400 MHz, Chloroform-*d*) δ 8.24 – 8.16 (m, 4H), 7.65 – 7.54 (m, 3H), 7.24 – 7.17 (m, 2H). ^{13}C NMR (101 MHz, Chloroform-*d*) δ 175.9, 168.3, 164.7 (d, *J* = 251.6 Hz), 133.0, 129.8 (d, *J* = 8.7 Hz), 129.3, 128.3, 124.3, 123.3 (d, *J* = 3.3 Hz), 116.2 (d, *J* = 22.0 Hz).

3-(4-methoxyphenyl)-5-phenyl-1,2,4-oxadiazole **5**

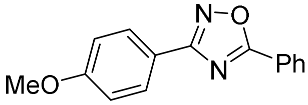

Yield: 70%. ^{1}H NMR (400 MHz, Chloroform-*d*) δ 8.23 – 8.20 (m, 2H), 8.14 – 8.10 (m, 2H), 7.64 – 7.53 (m, 3H), 7.04 – 7.00 (m, 2H), 3.89 (s, 3H). ^{13}C NMR (101 MHz, Chloroform-*d*) δ 175.6, 168.8, 162.0, 132.8, 129.3, 129.2, 128.3, 124.5, 119.5, 114.4, 55.6.

5-phenyl-3-(4-(trifluoromethyl)phenyl)-1,2,4-oxadiazole **6**

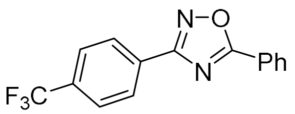

Yield: 61%. ^{1}H NMR (400 MHz, Chloroform-*d*) δ 8.31 (d, *J* = 8.0 Hz, 2H), 8.25 – 8.22 (m, 2H), 7.78 (d, *J* = 8.2 Hz, 2H), 7.67 – 7.56 (m, 3H). ^{13}C NMR (101 MHz, Chloroform-*d*) δ 176.3, 168.1, 133.2, 132.9, 130.5 (d, *J* = 1.4 Hz), 129.4, 128.9 (q, *J* = 30.7 Hz), 128.4, 128.0, 126.0 (q, *J* = 3.7 Hz), 124.1, 124.0 (q, J = 271.0 Hz).

5-phenyl-3-(pyridin-2-yl)-1,2,4-oxadiazole **7**

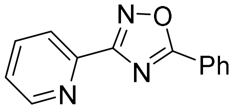

Yield: 73%. ^{1}H NMR (400 MHz, Chloroform-*d*) δ 8.90 – 8.86 (m, 1H), 8.31 – 8.28 (m, 2H), 8.26 (d, *J* = 7.8 Hz, 1H), 7.93 (t, *J* = 7.7 Hz, 1H), 7.66 – 7.61 (m, 1H), 7.59 – 7.54 (m, 2H), 7.50 (dd, *J* = 7.1, 5.1 Hz, 1H). ^{13}C NMR (101 MHz, Chloroform-*d*) δ 176.7, 168.6, 150.3, 146.2, 137.7, 133.2, 129.3, 128.5, 125.8, 124.1, 123.5.

5-phenyl-3-(p-tolyl)-1,2,4-oxadiazole **8**

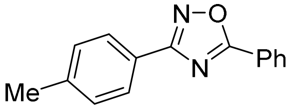

Yield: 65%. ^{1}H NMR (400 MHz, Chloroform-*d*) δ 8.25 – 8.21 (m, 2H), 8.09 – 8.05 (m, 2H), 7.64 – 7.53 (m, 3H), 7.32 (d, *J* = 7.9 Hz, 2H), 2.44 (s, 3H). ^{13}C NMR (101 MHz, Chloroform-*d*) δ 175.7, 169.1, 141.7, 132.8, 129.7, 129.2, 128.3, 127.6, 124.5, 124.2, 21.8.

3-(tert-butyl)-5-phenyl-1,2,4-oxadiazole **9**

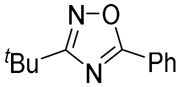

Yield: 59%. ^{1}H NMR (400 MHz, Chloroform-*d*) δ 8.15 – 8.11 (m, 2H), 7.60 – 7.49 (m, 3H), 1.43 (s, 9H). ^{13}C NMR (101 MHz, Chloroform-*d*) δ 178.5, 175.3, 132.6, 129.1, 128.2, 124.7, 28.6.

3-(3-fluorophenyl)-5-methyl-1,2,4-oxadiazole **10**

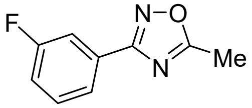

Yield: 31%. ^{1}H NMR (400 MHz, Chloroform-*d*) δ 7.86 (dt, *J* = 7.8, 1.2 Hz, 1H), 7.77 (ddd, *J* = 9.5, 2.4, 1.5 Hz, 1H), 7.46 (td, *J* = 8.0, 5.8 Hz, 1H), 7.20 (tdd, *J* = 8.4, 2.6, 0.9 Hz, 1H), 2.67 (s, 3H). ^{13}C NMR (101 MHz, Chloroform-*d*) δ 177.0, 167.7, 163.0 (d, *J* = 246.7 Hz), 130.7 (d, *J* =

8.1 Hz), 129.0 (d, J = 8.6 Hz), 123.2 (d, J = 3.2 Hz), 118.3 (d, J = 21.2 Hz), 114.5 (d, J = 23.7 Hz). ^{19}F NMR (376 MHz, Chloroform-d) δ -112.0 (td, J = 9.0, 5.7 Hz).

5-methyl-3-phenyl-1,2,4-oxadiazole **11**

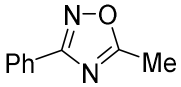

Yield: 51%. ^{1}H NMR (400 MHz, Chloroform-d) δ 8.08 – 8.04 (m, 2H), 7.51 – 7.45 (m, 3H), 2.66 (s, 3H). NMR spectra are identical to the reported in the literature.[21]

3-(4-methoxyphenyl)-5-methyl-1,2,4-oxadiazole **12**

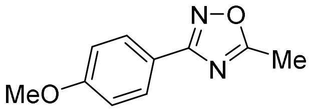

Yield: 42%. ^{1}H NMR (400 MHz, Chloroform-d) δ 8.02 – 7.98 (m, 2H), 7.00 – 6.97 (m, 2H), 3.87 (s, 3H), 2.64 (s, 3H). ^{13}C NMR (101 MHz, Chloroform-d) δ 176.4, 168.2, 162.0, 129.1, 119.4, 114.4, 55.5, 12.6.

5-methyl-3-(3-(trifluoromethyl)phenyl)-1,2,4-oxadiazole **13**

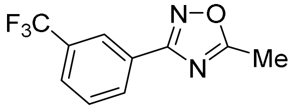

Yield: 31%. ^{1}H NMR (400 MHz, Chloroform-d) δ 8.35 (s, 1H), 8.26 (d, J = 7.8 Hz, 1H), 7.76 (d, J = 7.8 Hz, 1H), 7.62 (t, J = 7.8 Hz, 1H), 2.68 (s, 3H). ^{13}C NMR (101 MHz, Chloroform-d) δ 177.2, 167.5, 131.6 (q, J = 32.9 Hz), 130.6 (q, J = 1 Hz), 129.6, 127.9 (q, J = 3.2 Hz), 125.2, 124.5 (q, J = 3.8 Hz), 122.5, 12.6. ^{19}F NMR (376 MHz, Chloroform-d) δ -62.9.

5-methyl-3-(4-(trifluoromethyl)phenyl)-1,2,4-oxadiazole **14**

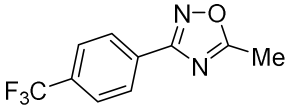

Yield: 49%. ^{1}H NMR (400 MHz, Chloroform-d) δ 8.19 (d, J = 8.0 Hz, 2H), 7.75 (d, J = 8.2 Hz, 2H), 2.69 (s, 3H). ^{13}C NMR (101 MHz, Chloroform-d) δ 177.2, 167.6, 133.0 (q, J = 32.7 Hz), 130.3 (q, J = 1.0 Hz), 127.8, 126.0 (q, J = 3.8 Hz), 124.0 (q, J = 270.0 Hz), 12.6. ^{19}F NMR (376 MHz, Chloroform-d) δ -63.0.

5-methyl-3-(p-tolyl)-1,2,4-oxadiazole **15**

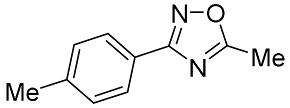

Yield: 33%. ^{1}H NMR (400 MHz, Chloroform-*d*) δ 7.95 (d, *J* = 8.2 Hz, 2H), 7.28 (d, *J* = 7.9 Hz, 2H), 2.65 (s, 3H). ^{13}C NMR (101 MHz, Chloroform-*d*) δ 176.5, 168.5, 141.6, 129.7, 127.4, 124.1, 21.7, 12.6.

3-(4-fluorophenyl)-5-methyl-1,2,4-oxadiazole **16**

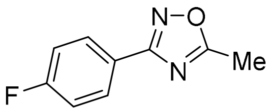

Yield: 39%. ^{1}H NMR (400 MHz, Chloroform-*d*) δ 8.09 – 8.04 (m, 2H), 7.20 – 7.14 (m, 2H), 2.66 (s, 3H). ^{13}C NMR (101 MHz, Chloroform-*d*) δ 176.8, 167.7, 164.6 (d, *J* = 251.3 Hz), 129.6 (d, *J* = 8.7 Hz), 123.2 (d, *J* = 3.2 Hz), 116.2 (d, *J* = 22.0 Hz), 12.6. ^{19}F NMR (376 MHz, Chloroform-*d*) δ -108.6 (tt, *J* = 8.4, 5.4 Hz).

5-(methyl-d_3)-3-phenyl-1,2,4-oxadiazole **17**

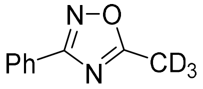

Yield: 19%. ^{1}H NMR (400 MHz, Chloroform-*d*) δ 8.08-8.04 (m, 2H), 7.52-7.46 (m, 3H). ^{13}C NMR (101 MHz, Chloroform-*d*) δ 176.6, 168.5, 131.3, 129.0, 127.5, 126.9.

Ethyl 2-(3-phenyl-1,2,4-oxadiazol-5-yl)acetate **18**

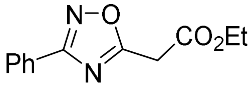

Yield: 42%. ^{1}H NMR (400 MHz, Chloroform-*d*) δ 8.13 – 8.05 (m, 2H), 7.53 – 7.46 (m, 3H), 4.26 (q, *J* = 7.1 Hz, 2H), 4.05 (s, 2H), 1.30 (t, *J* = 7.1 Hz, 3H). ^{13}C NMR (101 MHz, Chloroform-*d*) δ 172.8, 168.8, 165.9, 131.5, 129.0, 127.6, 126.6, 62.5, 33.4, 14.2.

Ethyl 2-(3-(4-methoxyphenyl)-1,2,4-oxadiazol-5-yl)acetate **19**

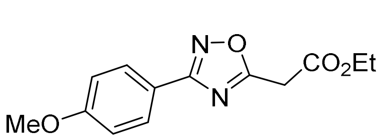

Yield: 28%. ^{1}H NMR (400 MHz, Chloroform-*d*) δ 8.04 – 8.00 (m, 2H), 7.01 – 6.97 (m, 2H), 4.26 (q, *J* = 7.2 Hz, 2H), 4.03 (s, 2H), 3.87 (s, 3H), 1.30 (t, *J* = 7.1 Hz, 3H). NMR spectra are similar to the reported in the literature.[22]

3,5-bis(4-fluorophenyl)-1,2,4-oxadiazole **20**

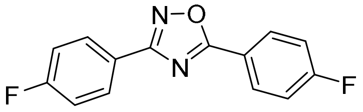

Yield: 73%. ^{1}H NMR (400 MHz, Chloroform-*d*) δ 8.23 (dd, *J* = 8.8, 5.3 Hz, 1H), 8.17 (dd, *J* = 8.8, 5.4 Hz, 1H), 7.25 (t, *J* = 8.7 Hz, 1H), 7.20 (t, *J* = 8.7 Hz, 1H). ^{13}C NMR (101 MHz, Chloroform-*d*) δ 175.0, 168.3, 165.6 (d, *J* = 255.5 Hz), 164.8 (d, *J* = 252.5 Hz), 130.8 (d, *J* = 9.2 Hz), 129.8 (d, *J* = 8.7 Hz), 123.2 (d, *J* = 3.2 Hz), 120.7 (d, *J* = 3.2 Hz), 116.7 (d, *J* = 22.3 Hz), 116.2 (d, *J* = 22.0 Hz). ^{19}F NMR (376 MHz, Chloroform-*d*) δ -104.9 (tt, *J* = 8.5, 5.4 Hz), -108.4 (tt, *J* = 8.4, 5.4 Hz).

5-(4-Methoxyphenyl)-3-(4-(trifluoromethyl)phenyl)-1,2,4-oxadiazole (RA16540) **21**

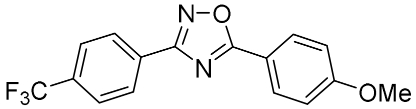

Yield: 26%. ^{1}H NMR (400 MHz, Chloroform-*d*) δ 8.30 (d, *J* = 8.1 Hz, 2H), 8.17 (d, *J* = 8.9 Hz, 2H), 7.77 (d, *J* = 8.3 Hz, 2H), 7.06 (d, *J* = 8.9 Hz, 2H), 3.92 (s, 3H). ^{19}F NMR (564 MHz, Chloroform-*d*) δ -63.0. NMR spectra are similar to the reported in the literature.[23]

Methyl 3-(5-(2-fluorophenyl)-1,2,4-oxadiazol-3-yl)benzoate (PTC124-OMe) **22**

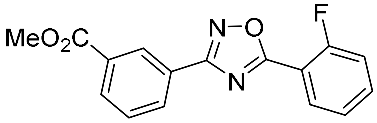

Yield: 51%. ^{1}H NMR (400 MHz, Chloroform-*d*) δ 8.85 (t, *J* = 1.5 Hz, 1H), 8.38 (dt, *J* = 7.8, 1.4 Hz, 1H), 8.25 (td, *J* = 7.7, 1.8 Hz, 1H), 8.21 (dt, *J* = 7.8, 1.4 Hz, 1H), 7.66 – 7.59 (m, 2H), 7.36 (td, *J* = 7.8, 1.0 Hz, 1H), 7.34 – 7.28 (m, 1H), 3.98 (s, 3H), 1.25 (s, 17H), 0.91 – 0.84 (m, 2H), 0.59 (dd, *J* = 9.6, 6.0 Hz, 1H). ^{13}C NMR (101 MHz, Chloroform-*d*) δ 173.2, 173.2, 168.2, 166.5, 160.9 (d, *J* = 260.7 Hz), 134.9 (d, *J* = 8.7 Hz), 132.4, 131.9, 131.1, 129.1 (d, *J* = 36.1 Hz), 127.3, 124.9 (d, *J* = 3.8 Hz), 117.4 (d, *J* = 20.9 Hz), 112.8 (d, *J* = 11.4 Hz), 52.5.

Table S5. Screening of the catalysts in synthesis of oxadiazole-1,2,4.[a]

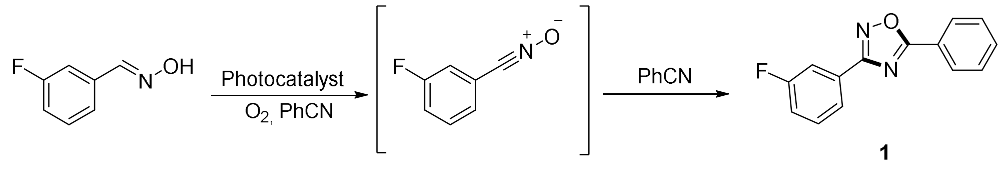

**Entry**	**Photocatalyst**	**Oxadiazole yields, %**	**TON[b]**
1	K-PHI	82	305
2	mpg-CN	55	—
3	$Ir(ppy)_3$	73	20
4	$Ru(bpy)_3Cl_2$	—	20
5	RFT	43	20
6	$[Mes\text{-}Acr]^+ClO_4^-$	15	20
7[c]	—	68	—

[a] Conditions: 3-fluorobenzaldehyde oxime: 50 µmol; benzonitrile: 3 mL; air; T 35°C; photocatalyst: 5 mol. % (for homogeneous catalysts) or 5 mg for K-PHI and mpg-CN; light: 461 nm, 88 mW·cm^{-2}; time: 24 h; controlled by ^{1}H NMR. [b] Non-optimized TON of the photocatalyst in oxime **1a** conversion. TON of K-PHI was calculated taking into account crystal cell parameters (a = b = 1.26370 nm, c = 3.2998 nm, α = 120°, cell volume 0.4564 nm^3) and particle size distribution from Figure S1g. Due to relatively low surface area of K-PHI (does not possess porosity), it is assumed that only unit cells located on the surface of K-PHI particle are involved in the photocatalytic reaction. TON of homogeneous catalysts was calculated as a ratio between a number of oxime molecules to a number of photocatalyst molecules (see SI for detailed calculations). [c] from 3-fluoro-N-hydroxybenzimidoyl chloride, Et_3N, PhCN.

In all cases complete conversion of the oxime occurred within 24 h. Both K-PHI and mpg-CN gave the target oxadiazole **1** in 82% and 55% yield respectively. Among the transition metal based sensitizers, $Ir(ppy)_3$ gave the target oxadiazole **1** with 73% yield, while $[Ru(bpy)_3]Cl_2$ did not give any product. RFT and $[Acr\text{-}Mes]^+ClO_4^-$ gave the **1** in 43% and 15% yield. Knowledge of K-PHI crystal structure allowed to calculate accurately the TON of carbon nitride in a photocatalytic reaction assuming that only unit cells on the surface of K-PHI particles participate in the reaction. The TON of K-PHI in oxime conversion is ca. 15 times higher than that for homogeneous photocatalysts. Oxadizole **1** can be also obtained in 68% yield from 3-fluoro-*N*-hydroxybenzimidoyl chloride in the presence of Et_3N, a common approach of nitrile oxides synthesis. In order to check if oxadiazoles-1,2,4 can be synthesized photocatalytically *via* electron transfer between oxime and K-PHI, we have tested a broad scope of electron acceptors in O_2-free environment (Table S5). Regardless of the used electron acceptor no oxadiazole was formed suggesting that $^1O_2$ is a key reagent in this transformation.

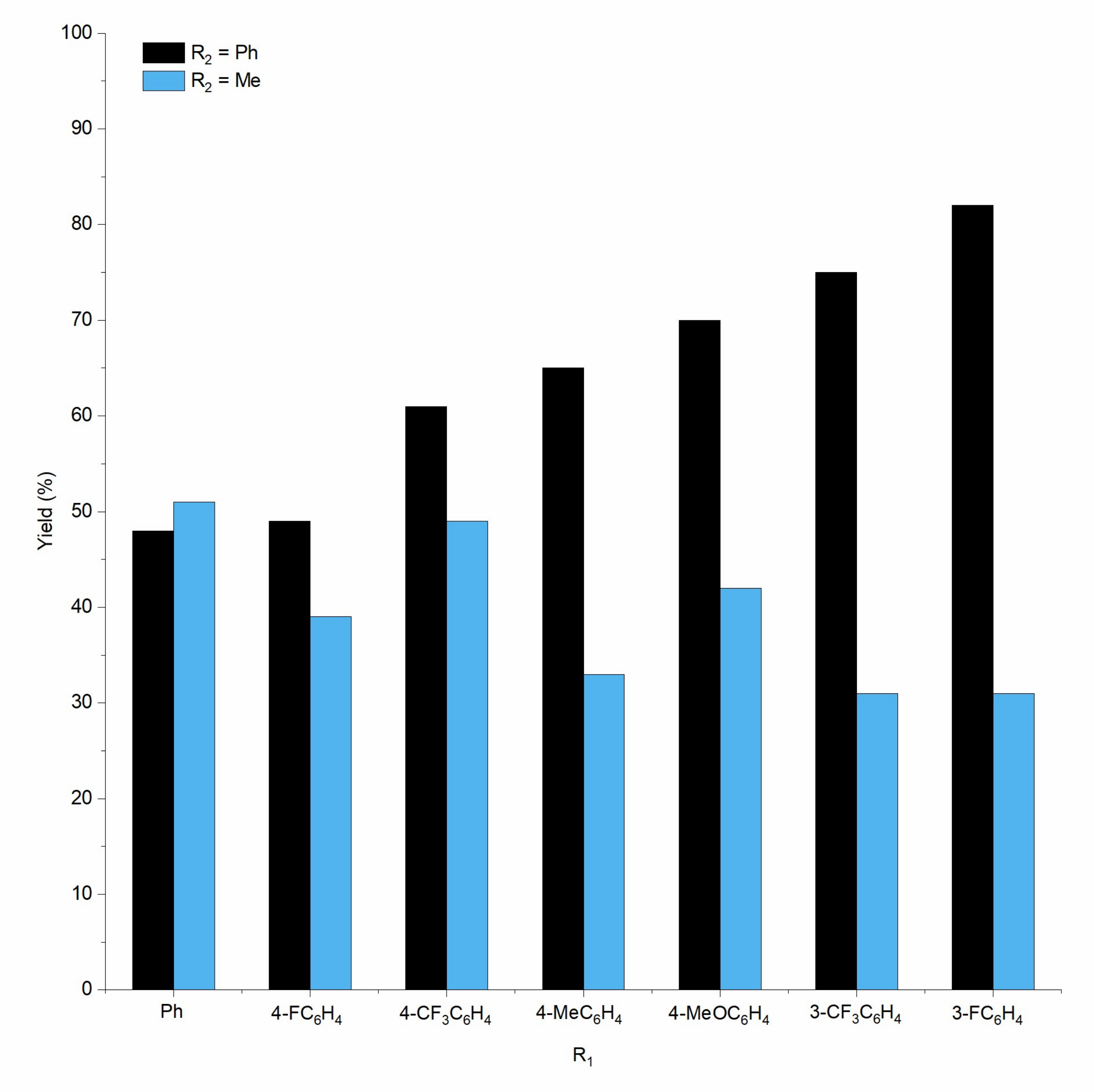

Figure S10. Comparison of oxadiazoles-1,2,4 yields derived from a reaction between aldehyde oximes and either acetonitrile or benzonitrile.

Table S6. Electron acceptors/additives screening.

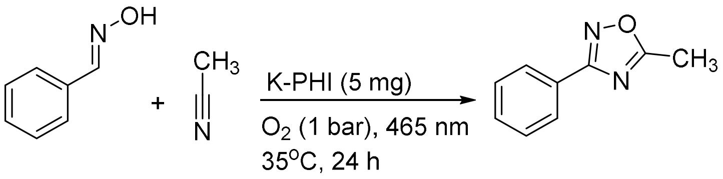

Entry	Electron acceptor/additive	Oxime conversion, %	5-methyl-3-phenyl-1,2,4-oxadiazole, %	Example of the electron scavenger using [Reference]
1	$PhNO_2$ (140 µmol)	0	0	[24]
2	$MV^{2+}2Cl^-$ (9 µmol)	0	0	[25]
3	AIBN (102 µmol)	0	0	-
4	$(CO)_6x8H_2O$ (90 umol)	0	0	-
5	*p*-benzoquinone (106 µmol)	0	0	-
6	PhSSPh (59 umol)	10	0	[26]
7[b]	S_8 219 umol (sulfur atoms)	60	0[g]	[27]
8	^{t}BuONO (126 umol)	66	10	-
9[f]	^{t}BuONO (126 umol)	100	44	-
10[b,c,d]	Acetone (135 umol)	1	1	[1]
11[d,e]	Phosphate buffer (pH = 9) (1.5 mL)	10	1	-

[a] Conditions: benzaldehyde oxime (50 µmol); MeCN 3 mL; K-PHI 5 mg; N_2 1 bar; T=35°C; 24 h; 461 nm (88 mW·cm^{-2}). Reaction mixture composition was analysed by GC-MS.

[b] 64 h;

[c] Argon;

[d] Reaction mixture composition was analysed by ^{1}H NMR;

[e] MeCN 1.5 mL; Air 1 bar;

[f] in dark; without K-PHI;

[g] benzonitrile (54%) was formed as a main product;

O_2 is required in a stoichiometric amount with the respect to the oxime (Table S6).

Table S7. Influence of oxygen concentration on 5-methyl-3-phenyl-1,2,4-oxadiazole yield.[a]

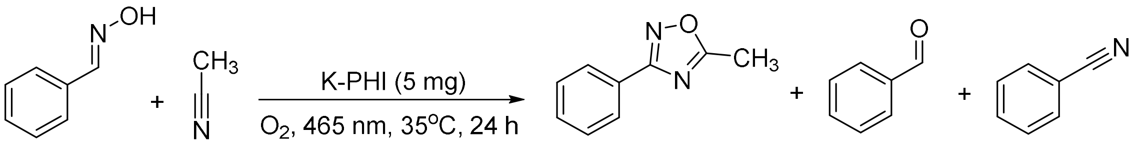

Entry	O_2	Oxime conversion, %	Products distribution		
			Benzaldehyde, %	Benzonitrile, %	5-methyl-3-phenyl-1,2,4-oxadiazole, %
1	O_2 1 bar (excess)	100	32	19	49
2	O_2 2 mL (ca. 90 μmol)	100	37	18	44
3	O_2 0.2 mL (ca. 9 μmol)	47	20	45	34

[a] Conditions: benzaldehyde oxime (50 μmol); MeCN 3 mL; K-PHI 5 mg; T=35°C; 24 h; 461 nm (88 mW·cm^{-2}). Reaction mixture composition was analysed by GC-MS.

The reaction does not proceed in the absence of the photocatalyst and light (Table S7).

Table S8. Reaction conditions screening.[a]

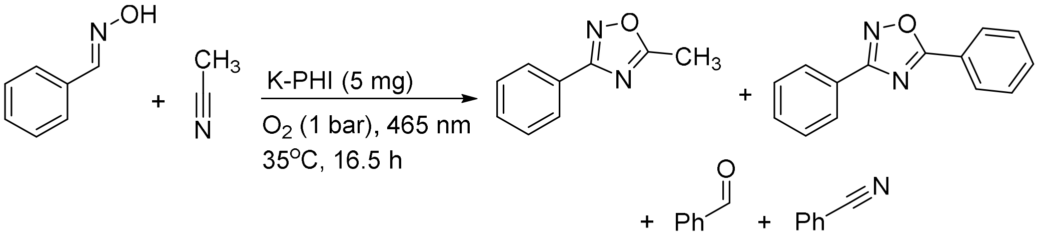

Entry	Photocatalyst	O_2	Light	Oxime conversion, %	Products distribution			
					Benzaldehyde, %	Benzonitrile, %	3,5-diphenyl-1,2,4-oxadiazole, %	5-methyl-3-phenyl-1,2,4-oxadiazole, %
1	+	+	+	100	31	18	<1	48
2	+	+	-	13	<1	100	0	0
3	+	-	-	15	<1	100	0	0
4	+	-	+	20	11	82	5	3
5	-	+	+	8	<1	100	0	0
6	-	-	+	8	<1	100	0	0
7	-	+	-	7	<1	100	0	0
8	-	-	-	10	<1	100	0	0
9[b]	-	-	-	19	<1	100	0	0

[a] Conditions: benzaldehyde oxime (50 µmol); K-PHI 5 mg; O_2 1 bar; T=35°C; 16.5 h; 461 nm (88 mW·cm^{-2}). Reaction mixture composition was analysed by GC-MS.

[b] benzaldehyde oxime in MeCN (17 mM) injected in GC-MS.

Furthermore, the reaction is tolerant to water content up to at least 10 vol. % (Table S8).

Table S9. Influence of water content.

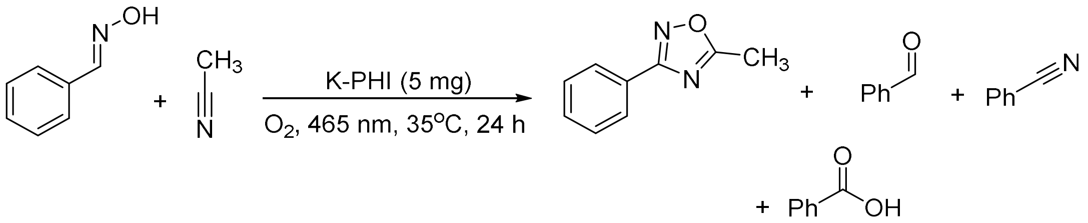

Entry	Water concentration, %	Oxime conversion, %	Benzaldehyde, %	Benzonitrile, %	Benzoic acid, %	5-methyl-3-phenyl-1,2,4-oxadiazole, %
1	<0.1	100	26	17	17	39
2	0.3	100	22	17	14	46
3[b]	3	100	8	-	27	38
4[b]	10	100	2	-	34	35

[a] Conditions: benzaldehyde oxime (50 μmol); MeCN 3 mL; Air 1 bar; K-PHI 5 mg; T=35°C; 24 h; 461 nm (88 mW·cm^{-2}). Reaction mixture composition was analysed by GC-MS.

[b] Reaction mixture composition was analysed by ^{1}H NMR

Table S10. Solvent screening.[a]

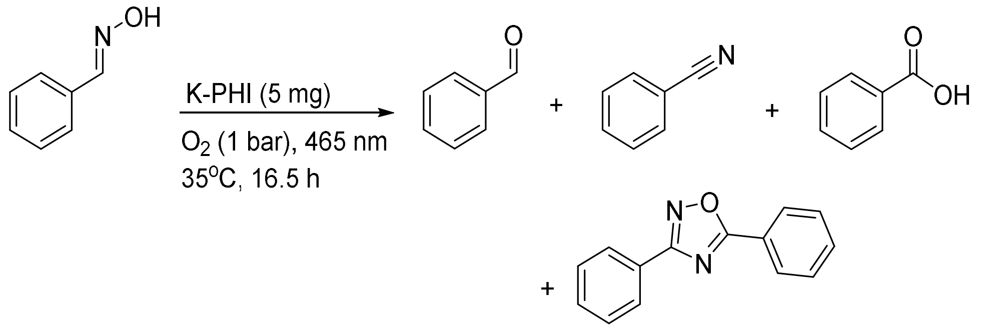

Entry	Solvent	Oxime conversion, %	Products distribution			
			Benzaldehyde, %	Benzonitrile, %	Benzoic acid, %	3,5-diphenyl-1,2,4-oxadiazole, %
1	CH_2Cl_2 (3mL)	100	6	10	42	-
2	1,4-dioxane (3 mL)	46	39	36	-	19
3[b]	^{t}BuOH (3 mL)	100	62	20	18	-
4	Acetone (3 mL)	100	50	14	12	-
5	Anisole (3mL)	54	68	30	-	2
6[b]	Hexane (3mL)	100	-	4	96	-
7[b]	Benzene (3 mL)	100	28	<1	43	-
8	$MeNO_2$ (3 mL)	100	36	21	7	<1
9	$MeOCH_2CH_2OMe$ (3 mL)	100	2	<1	6	-
10	DMSO (3mL)	50	25	25	-	-
11	DMF (3 mL)	68	41	59	-	<1
12	$PhCF_3$ (3 mL)	100	24	25	30	-
13	[EMIM] OTf^- (1 mL)	93	46	30	-	-
14[c,d]	1,4-dioxane:water (9:1) 3 mL	10	5	-	-	-
15[c,d]	Hexane (3 mL)	100	24	-	31	-

[a] Conditions: benzaldehyde oxime (50 µmol); K-PHI 5 mg; O_2 1 bar; T=35°C; 24 h; 461 nm (88 mW·cm^{-2}). Reaction mixture composition was analysed by GC-MS;

[b] 64 h;

[c] air 1 bar;

[d] Reaction mixture composition was analysed by ^{1}H NMR;

Table S11. Variation of the photocatalyst amount.[a]

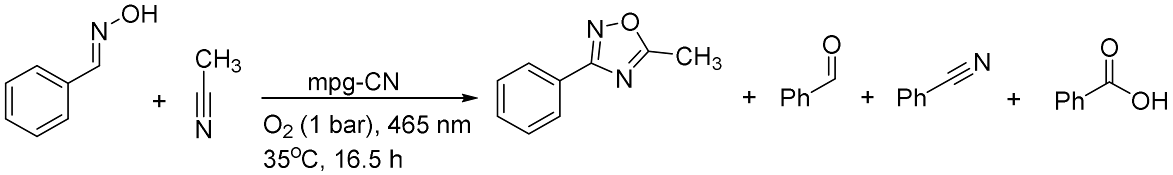

Entry	mpg-CN, mg	Oxime conversion, %	Products distribution			
			Benzaldehyde, %	Benzonitrile, %	Benzoic acid, %	5-methyl-3-phenyl-1,2,4-oxadiazole, %
1[b]	5	100	35	14	12	39
2	5	100	40	15	12	34
3	5	100	51	15	0	35
4	10	100	30	14	12	41
5	20	100	25	16	20	39

[a] Conditions: benzaldehyde oxime (50 µmol); photocatalyst mpg-CN; MeCN 3 mL; O_2 1 bar; 24 h; 461 nm (88 mW·cm^{-2}). Reaction mixture composition was analysed by GC-MS;

[b] Photocatalyst: mpg-CN (2.5 mg) + K-PHI (2.5 mg);

Table S12. Solvents screening for a reaction between 3-fluorobenzaldehyde oxime and benzonitrile.[a]

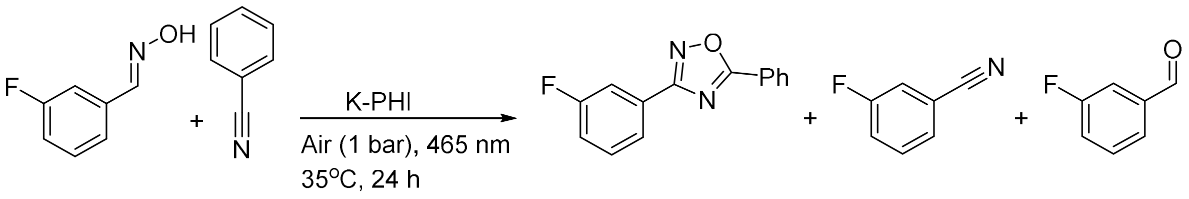

Entry	Solvent	Oxime conversion, %	Products distribution		
			3-(3-fluorophenyl)-5-phenyl-1,2,4-oxadiazole, %	3-fluorobenzonitrile, %	3-fluorobenzaldehyde, %
1	Dioxane-1,4	43	0	75	25
2	DMF	16	0	100	0
3	DMSO	16	0	100	0
4	$MeNO_2$	68	0	48	52
5	MeCN	100	31[b]	32	37

[a] Conditions: 3-fluorobenzaldehyde oxime 50 μmol; PhCN 100 μmol; photocatalyst K-PHI 5 mg; solvent 3 mL; Air 1 bar; 24 h; 461 nm (88 mW·cm^{-2}). Reaction mixture composition was analysed by GC-MS;

[b] 3-(3-fluorophenyl)-5-methyl-1,2,4-oxadiazole was formed instead of 3-(3-fluorophenyl)-5-phenyl-1,2,4-oxadiazole;

4.3. A general procedure of isoxazoles synthesis using K-PHI photocatalyst

A 5 mL glass tube equipped with a magnetic stir bar was charged with 1,3-diphenylprop-2-en-1-one oxime (27 µmol), K-PHI (5 mg) and MeCN (3 mL). The tube was covered with a rubber septum. A rubber balloon filled with air (ca. 1 bar, absolute pressure) was connected to the reaction mixture headspace via a steel needle. The reaction mixture was vigorously stirred at 35°C under blue light irradiation (461 nm, 88 mW·cm^{-2}) for 24 h. K-PHI was separated by centrifugation (13000 rpm, 3 min), dispersed in MeCN (2 mL) and separated by centrifugation (13000 rpm, 3 min). K-PHI was rinsed in total 3 times. Solutions after K-PHI washing were combined and concentrated in vacuum (30 mbar) at 50°C. The residue was purified by column chromatography on silica gel using hexane:ethylacetate (19:1).

3,5-diphenylisoxazole **24**

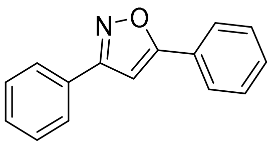

Yield: 28%. ^{1}H NMR (400 MHz, Chloroform-*d*) δ 7.89 – 7.84 (m, 4H), 7.53 – 7.47 (m, 6H), 6.84 (s, 1H). ^{13}C NMR (101 MHz, Chloroform-*d*) δ 170.5, 163.1, 130.4, 130.2, 129.2, 129.1, 127.6, 127.0, 126.7, 126.0, 97.6.

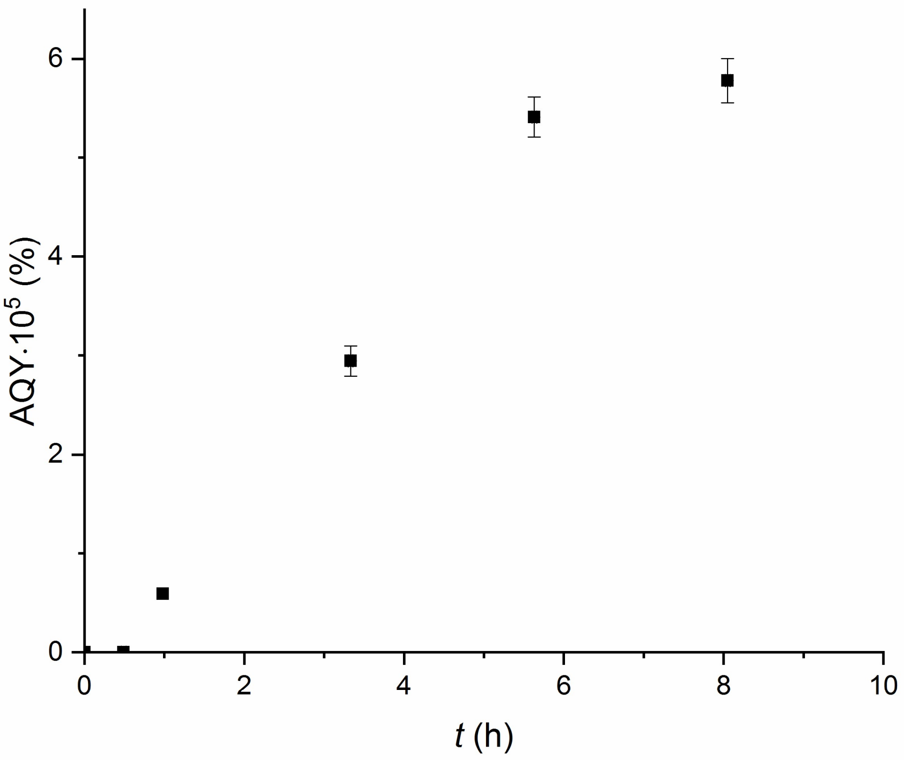

Figure S11. Time-dependent AQY study.

Conditions: benzaldehyde oxime 16.8 mg, K-PHI 13.5 mg, MeCN 8.1 mL, O_2 1 bar, T = 35°C, 461 nm (88 mW·cm^{-2}). Reaction progress was monitored by ^{1}H NMR using 1,1,2,2-tetrachloroethane as internal standard. Error bars represent standard deviation based on 3 measurements.

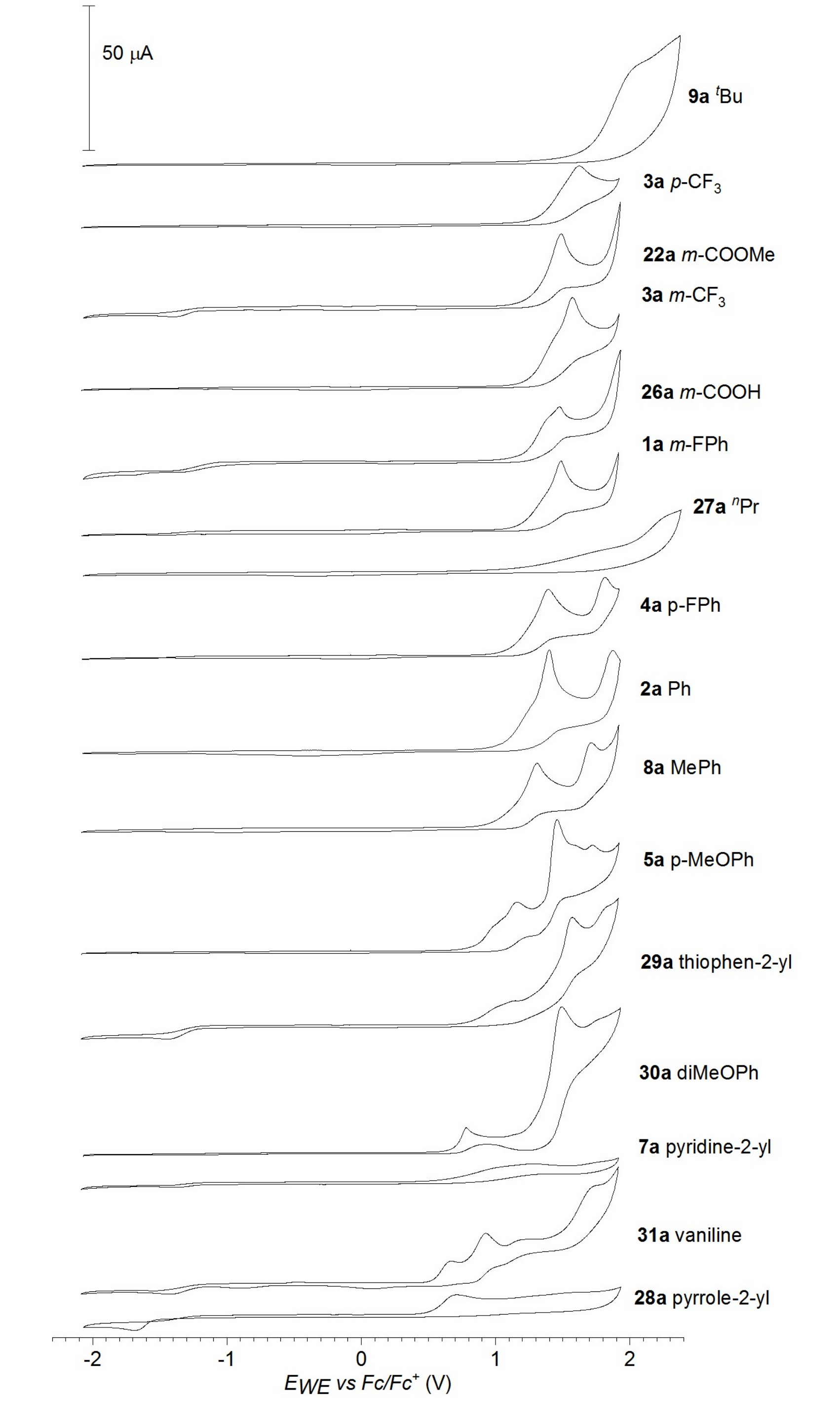

Figure S12. Cyclic voltammetry study on aldehyde oximes.

The CV curves are arranged from low oxidation potential (bottom) to high (top). Glassy carbon (diameter 3 mm) was used as a WE, Ag wire in $AgNO_3$ (0.01M) with tetrabutylammonium perchlorate (0.1M) in MeCN as a reference electrode (RE), Pt wire as a counter electrode. Electrolyte: a solution of $(n\text{Bu})_4N^+$ ClO_4^- in MeCN (0.1 M), oxime concentration 2 mM.

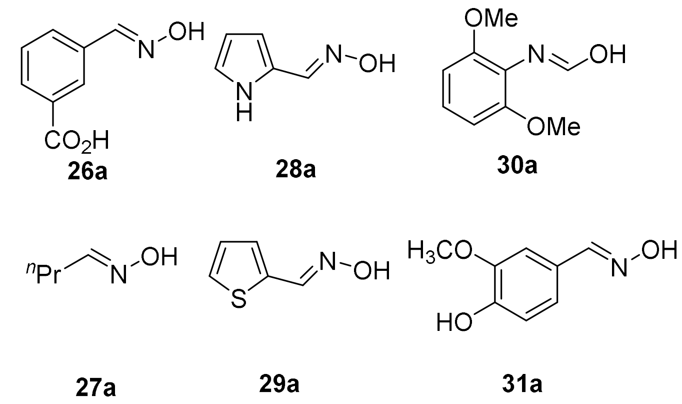

Figure S13. Oximes that exclusively undergo path B (deoxymation path).

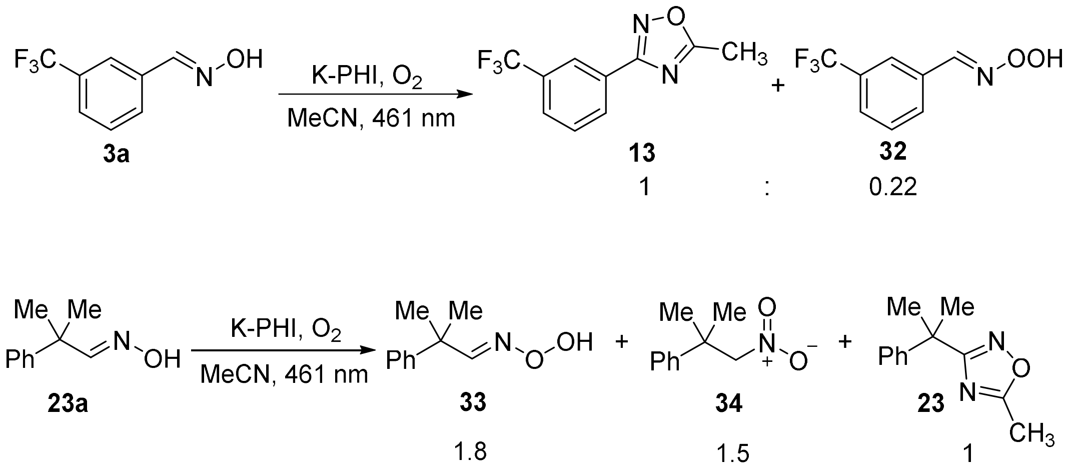

Figure S14. Side products identification.

3-(trifluoromethyl)benzaldehyde oxime **3a** gave the target oxadiazole **13** and a product with a tentative structure **32** in 1:0.22 ratio. Even stronger tendency was observed in case of 2-methyl-2-phenylpropanal oxime **23a**. Herein, we also observed formation of nitrocompound **34**.

5. NMR spectra

5.1. NMR spectra of oximes 1a-31a

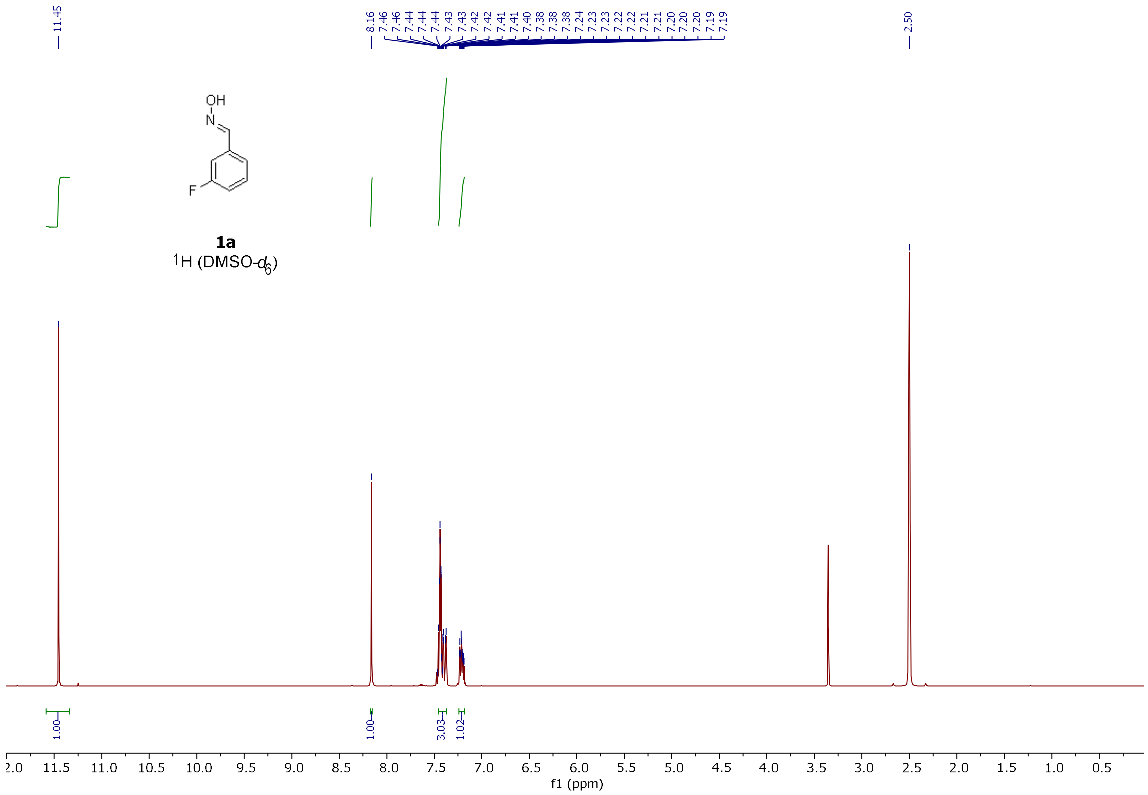

Figure S15. ^{1}H NMR spectrum of **1a**.

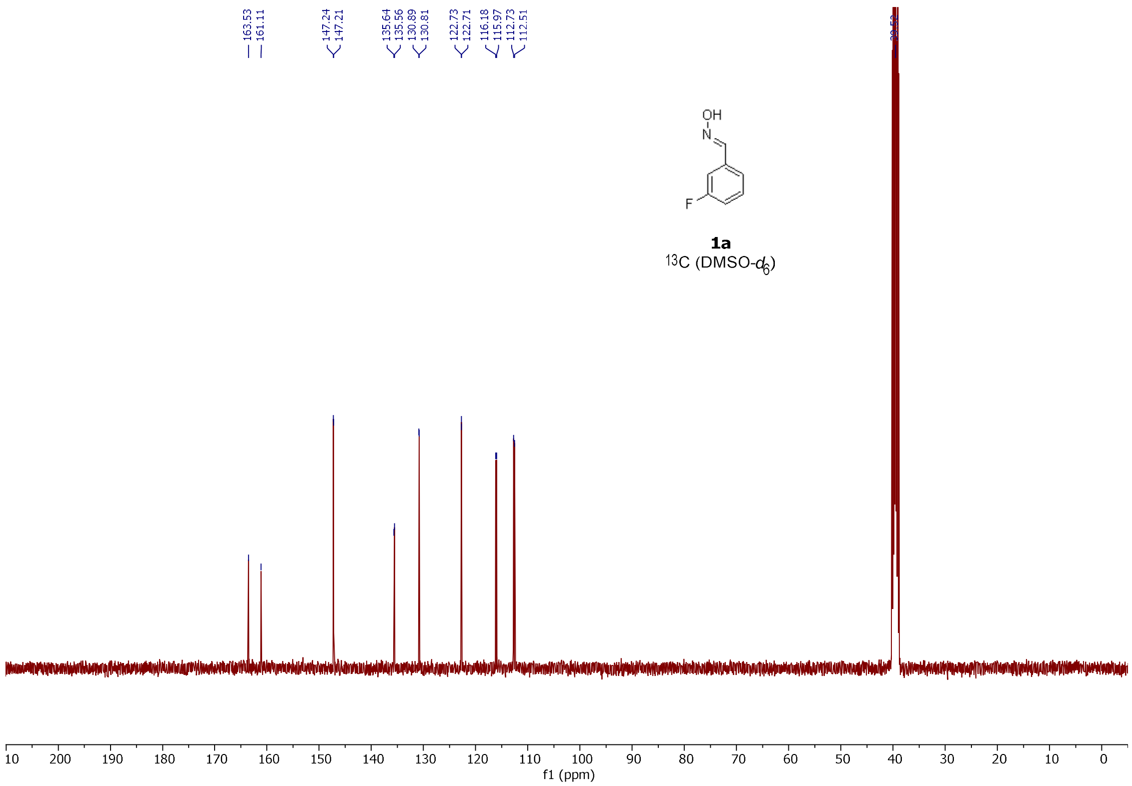

Figure S16. 13C NMR spectrum of **1a**.

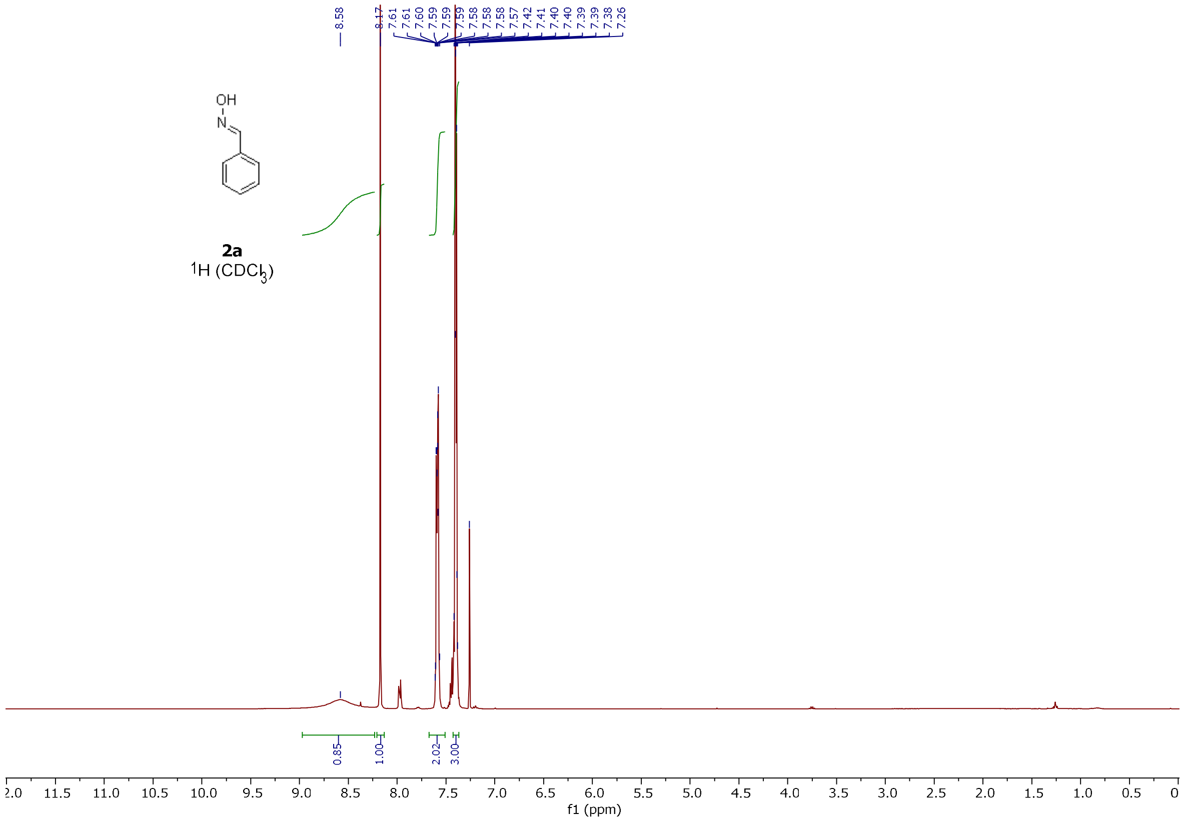

Figure S17. 1H NMR spectrum of **2a**.

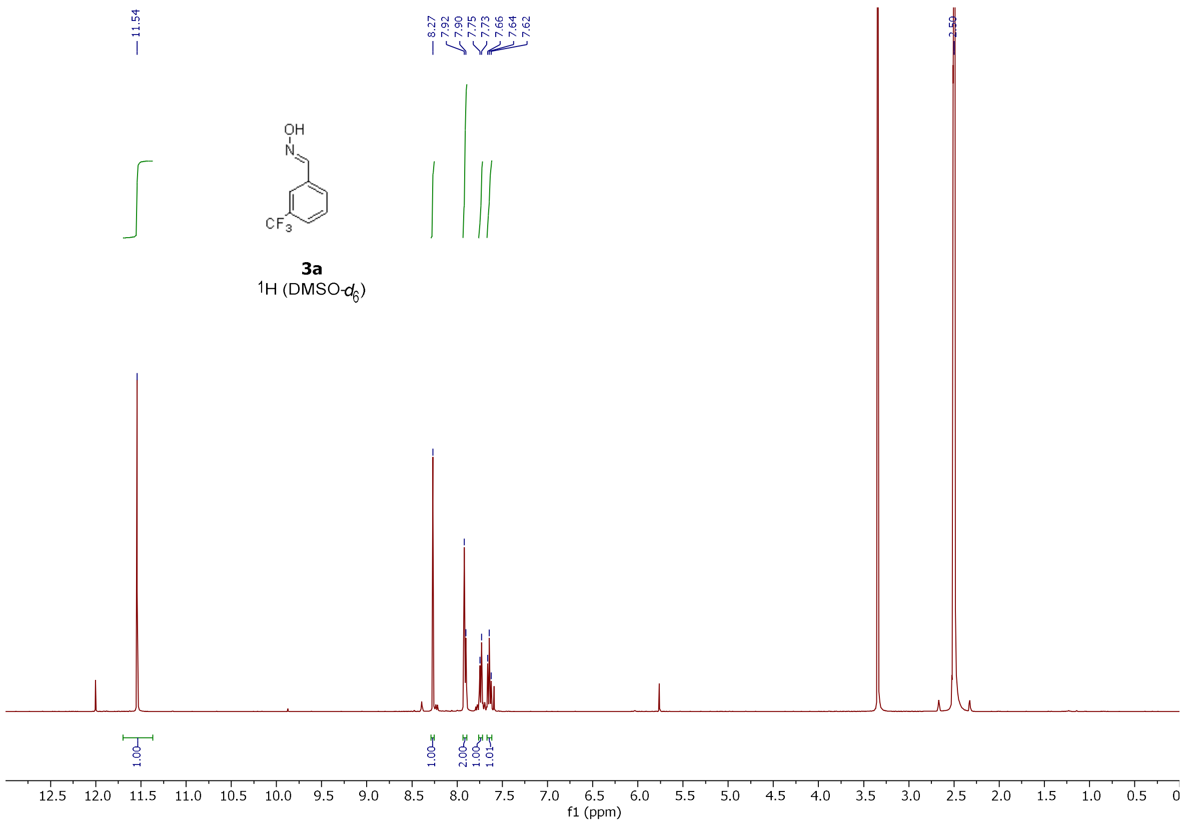

Figure S18. ^{1}H NMR spectrum of **3a**.

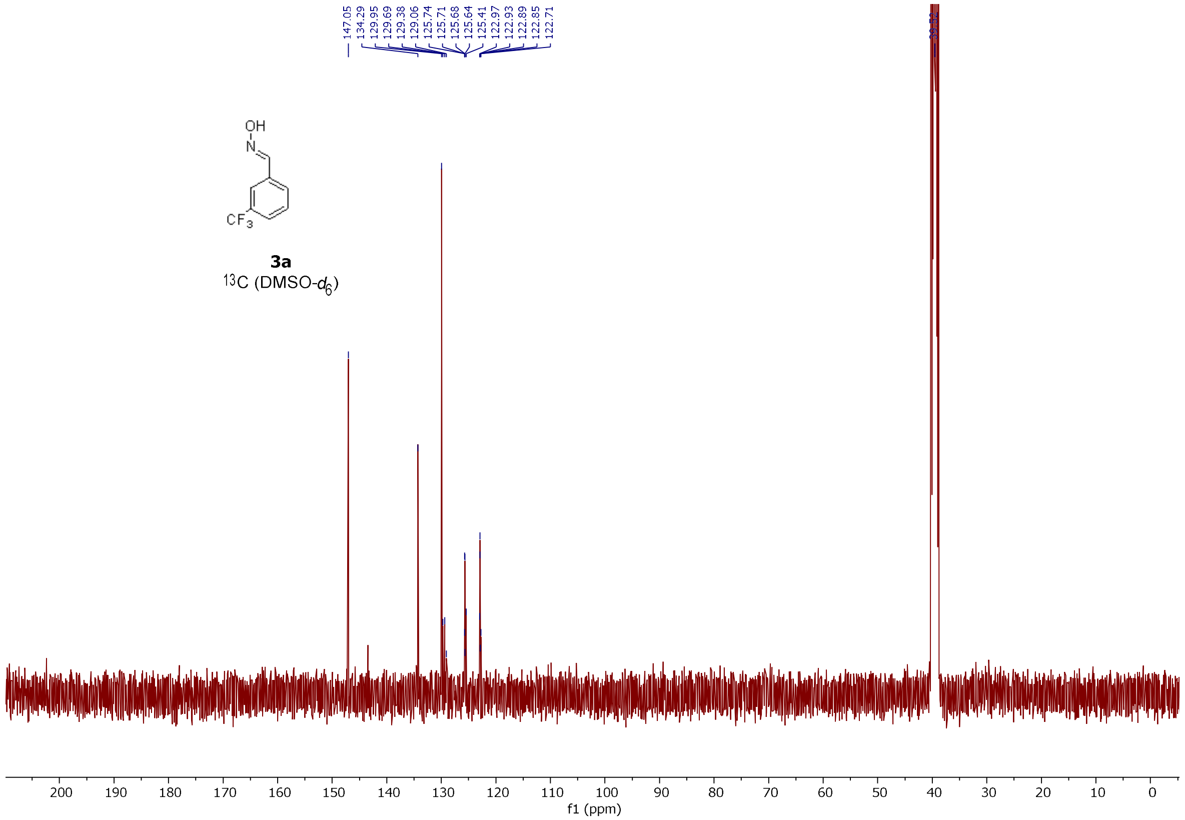

Figure S19. ^{13}C NMR spectrum of **3a**.

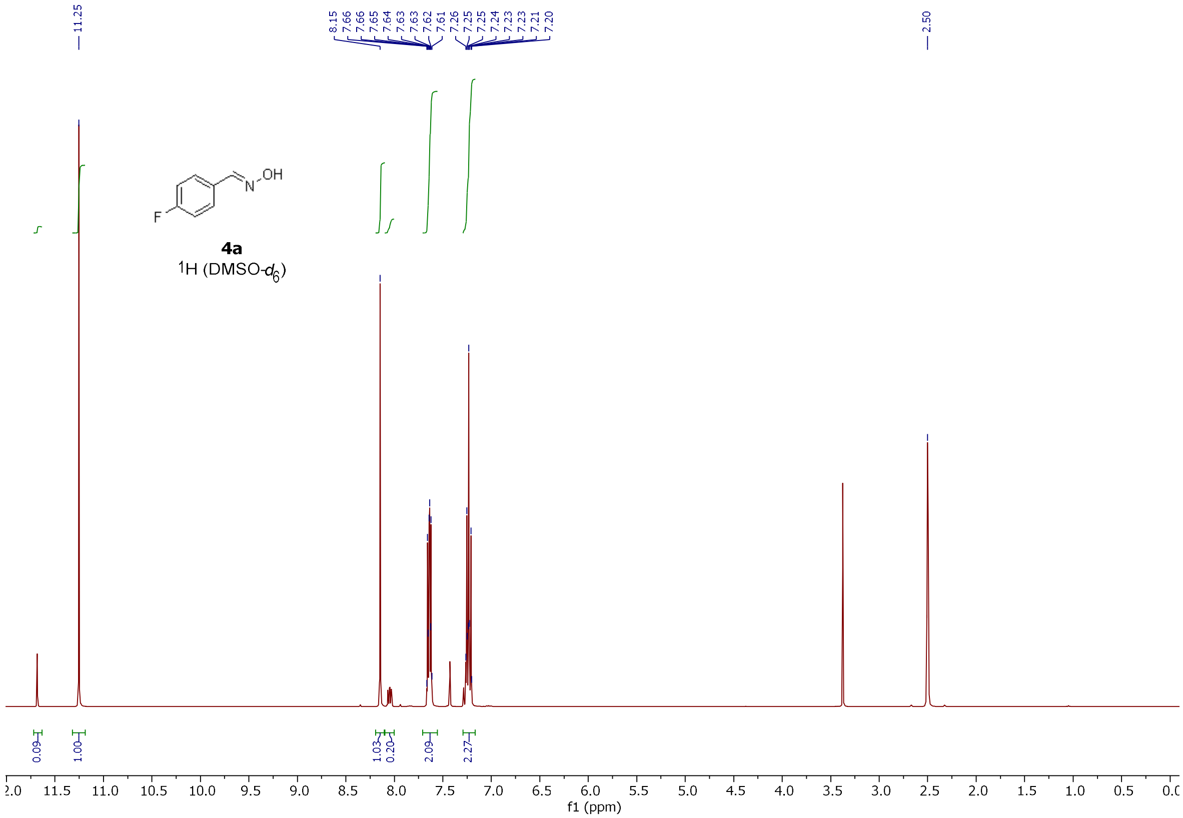

Figure S20. ^{1}H NMR spectrum of **4a.**

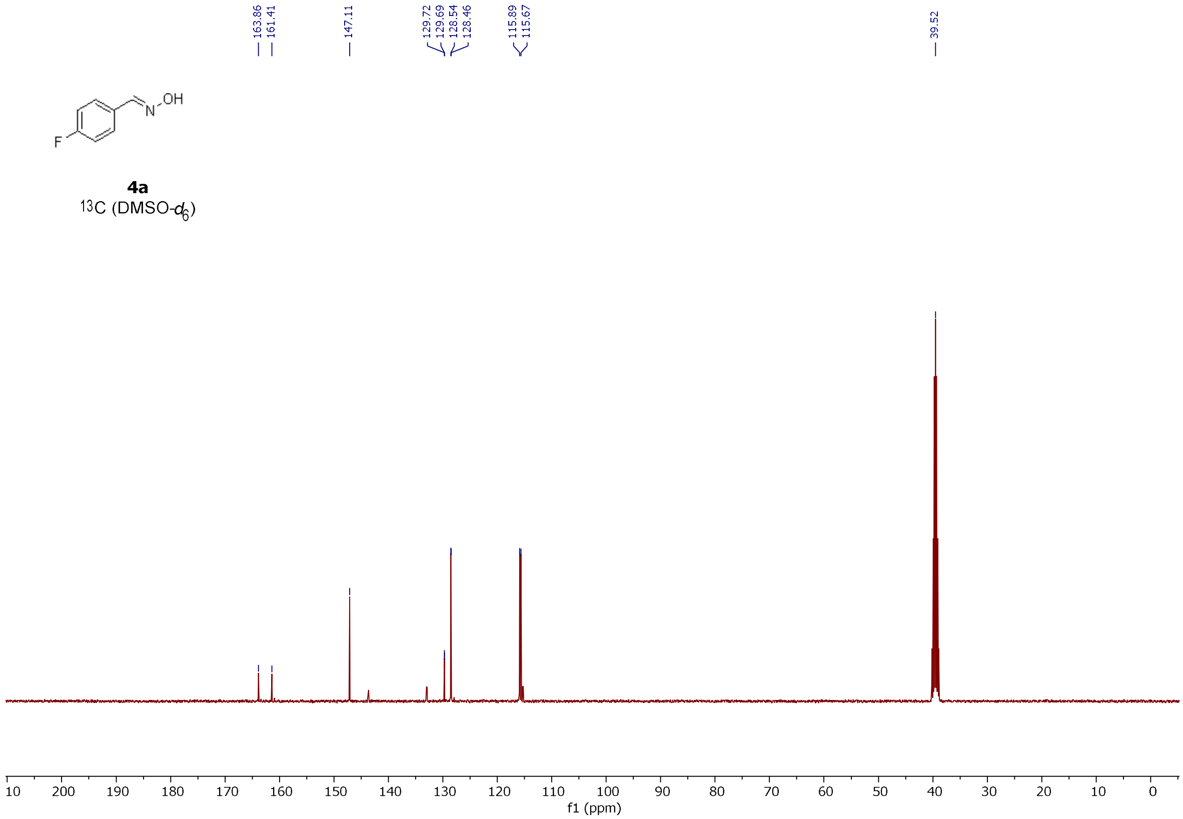

Figure S21. ^{13}C NMR spectrum of **4a.**

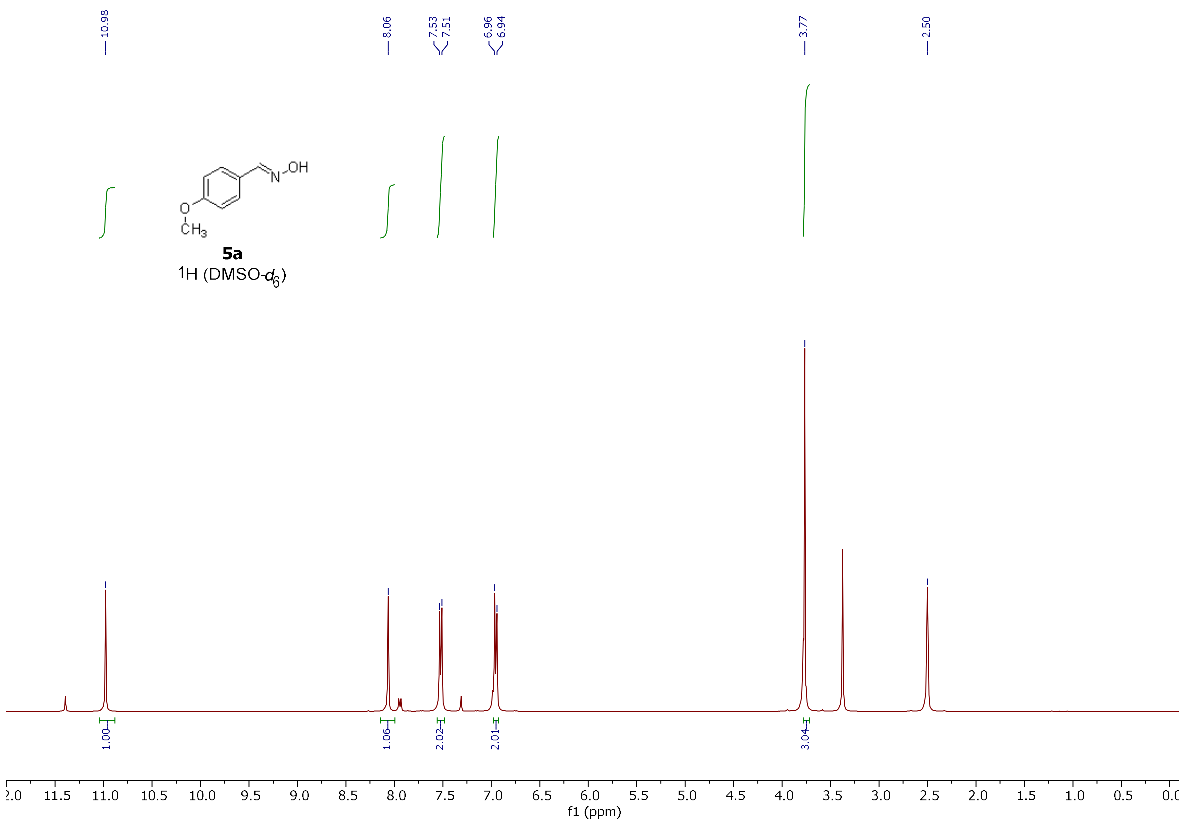

Figure S22. ^{1}H NMR spectrum of **5a.**

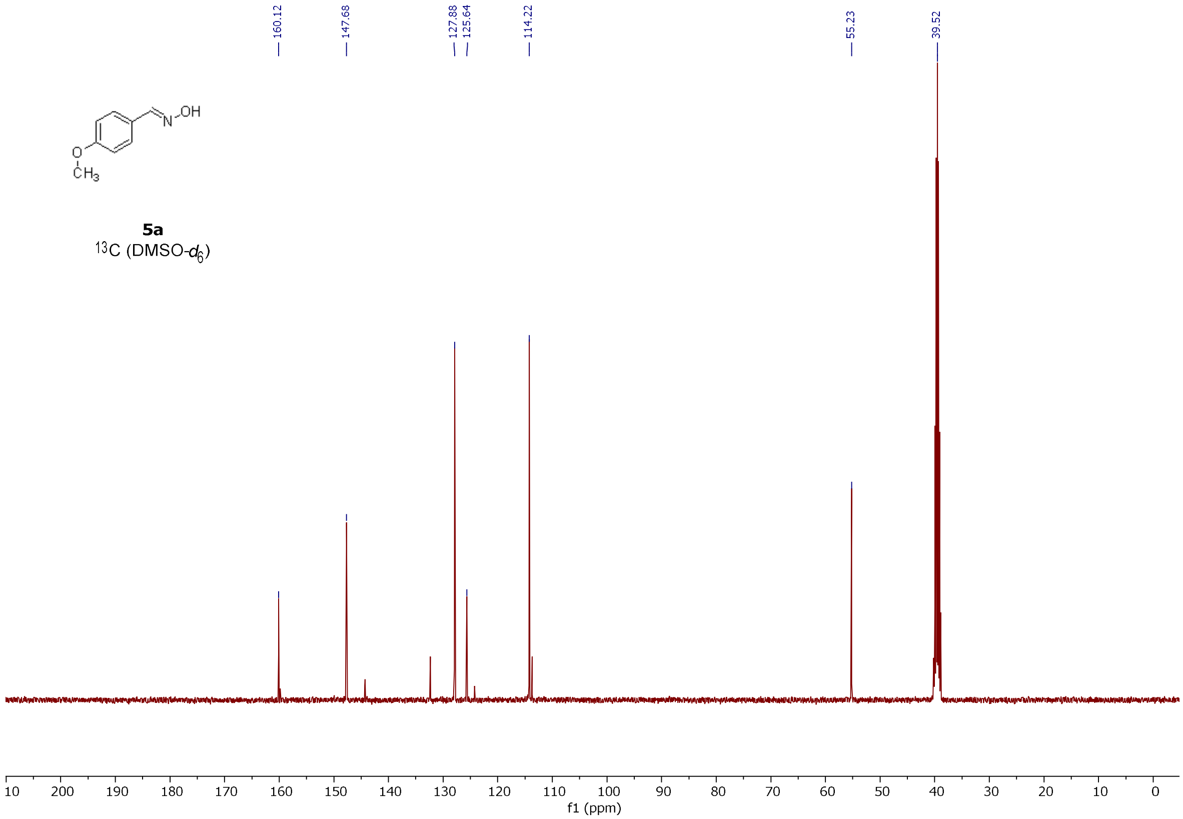

Figure S23. ^{13}C NMR spectrum of **5a.**

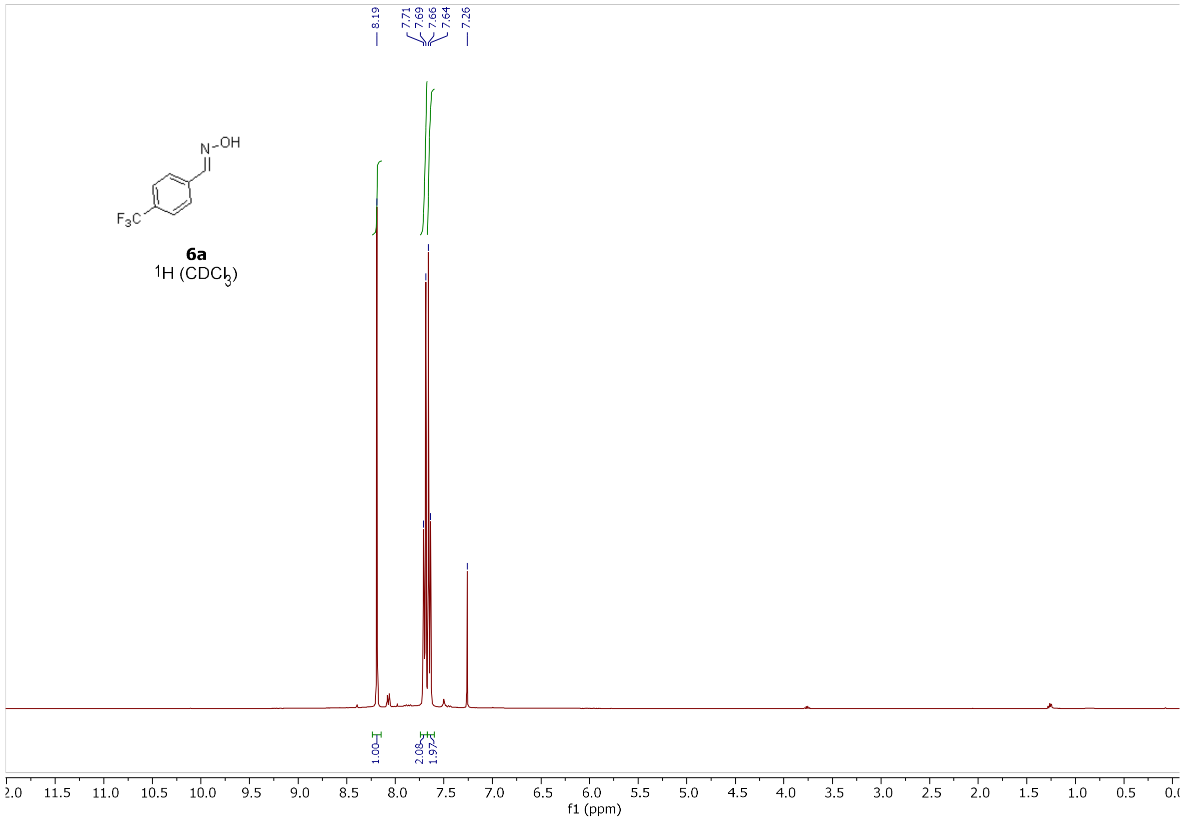

Figure S24. ^{1}H NMR spectrum of **6a.**

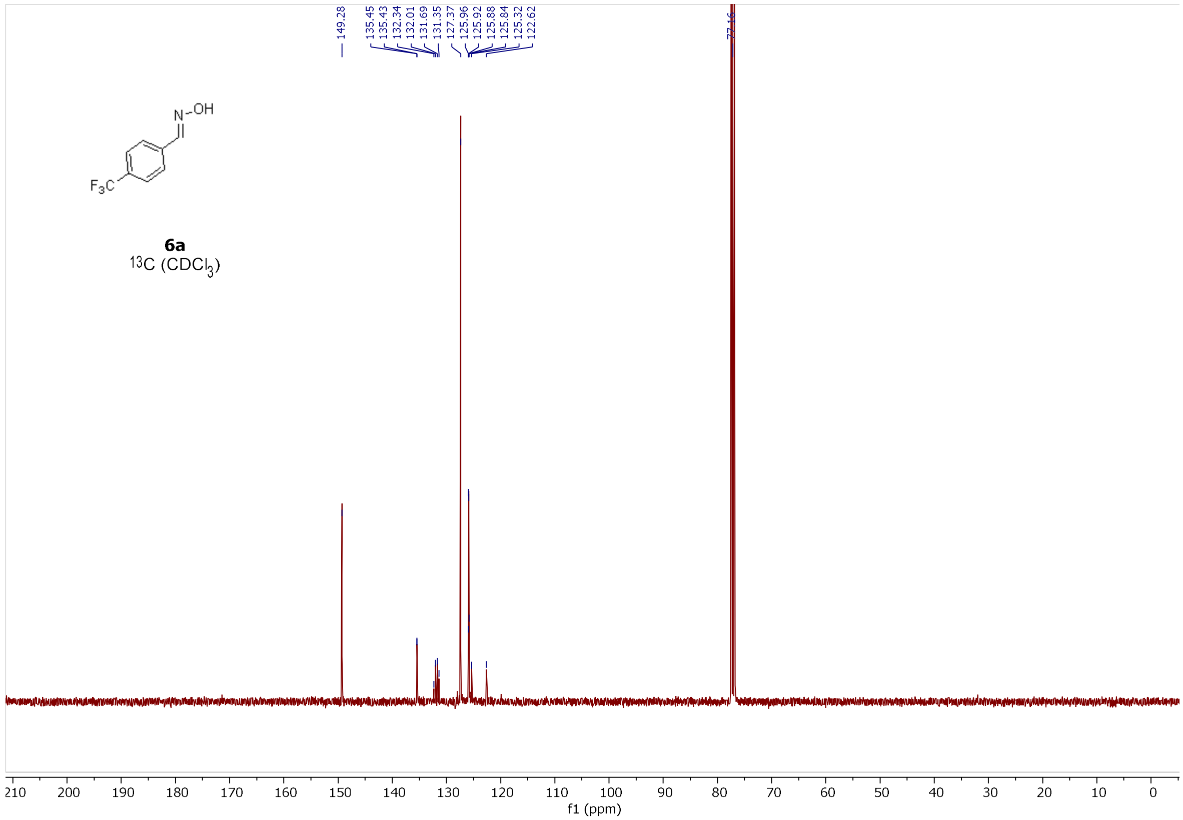

Figure S25. ^{13}C NMR spectrum of **6a.**

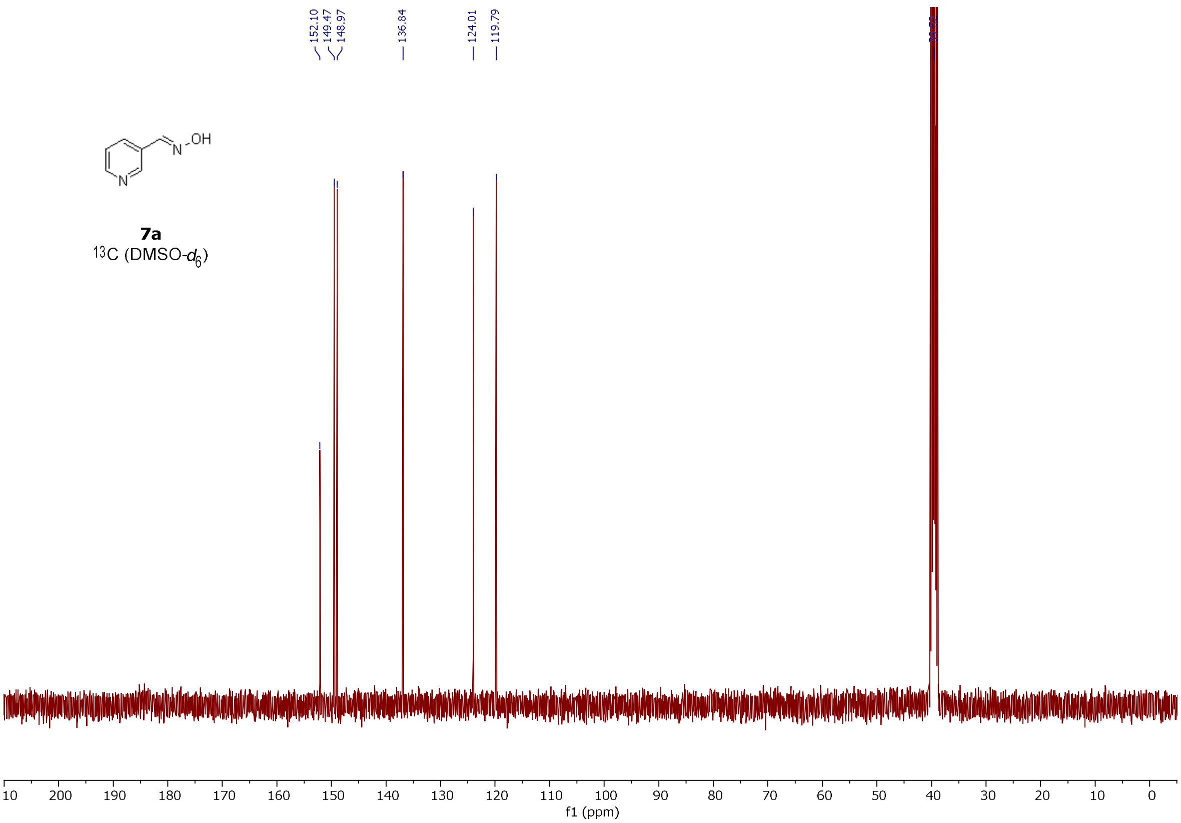

Figure S26. ^{13}C NMR spectrum of **7a.**

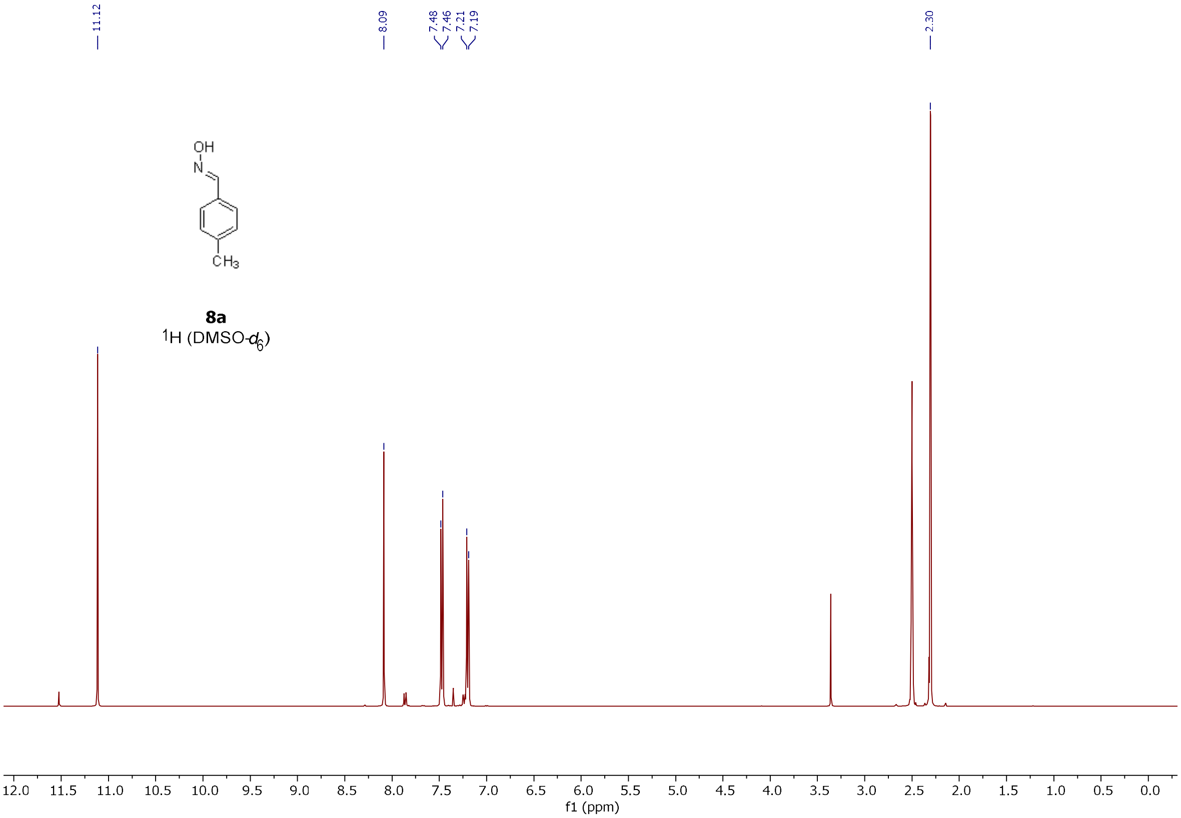

Figure S27. ^{1}H NMR spectrum of **8a.**

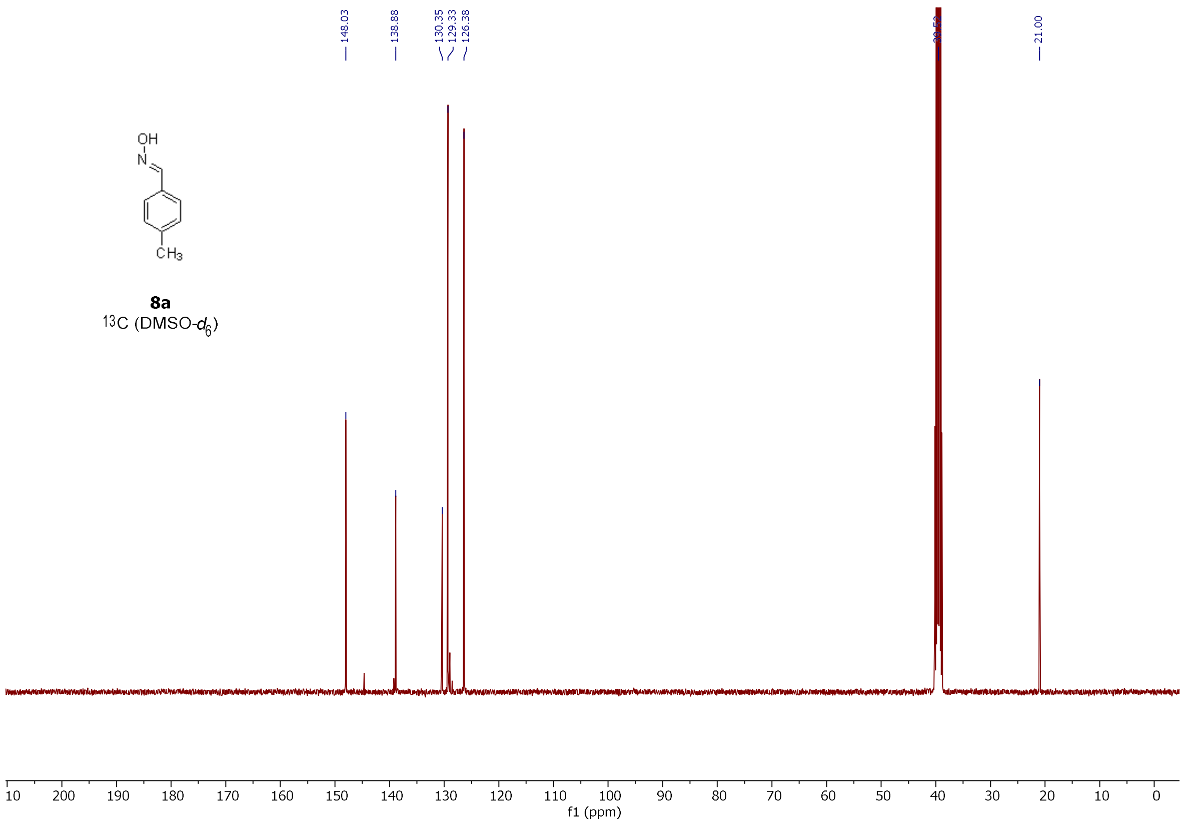

Figure S28. ^{13}C NMR spectrum of **8a.**

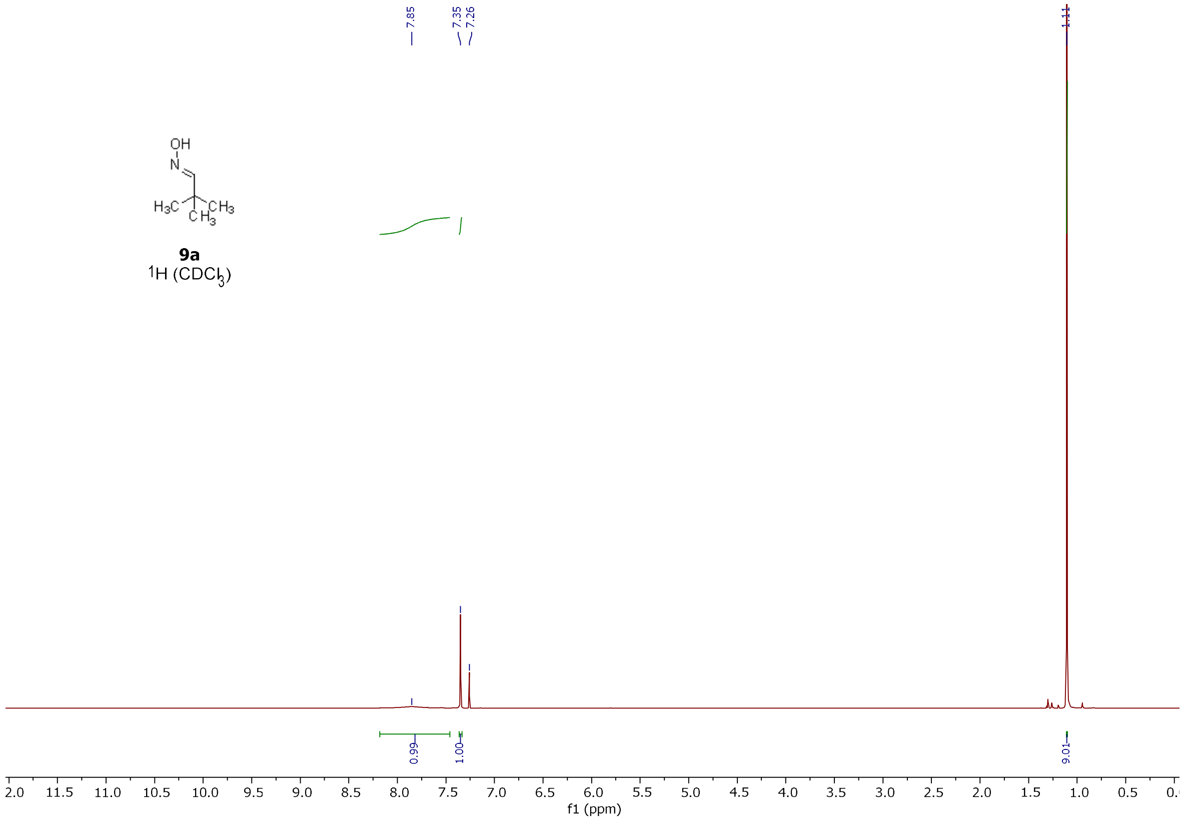

Figure S29. ^{1}H NMR spectrum of **9a.**

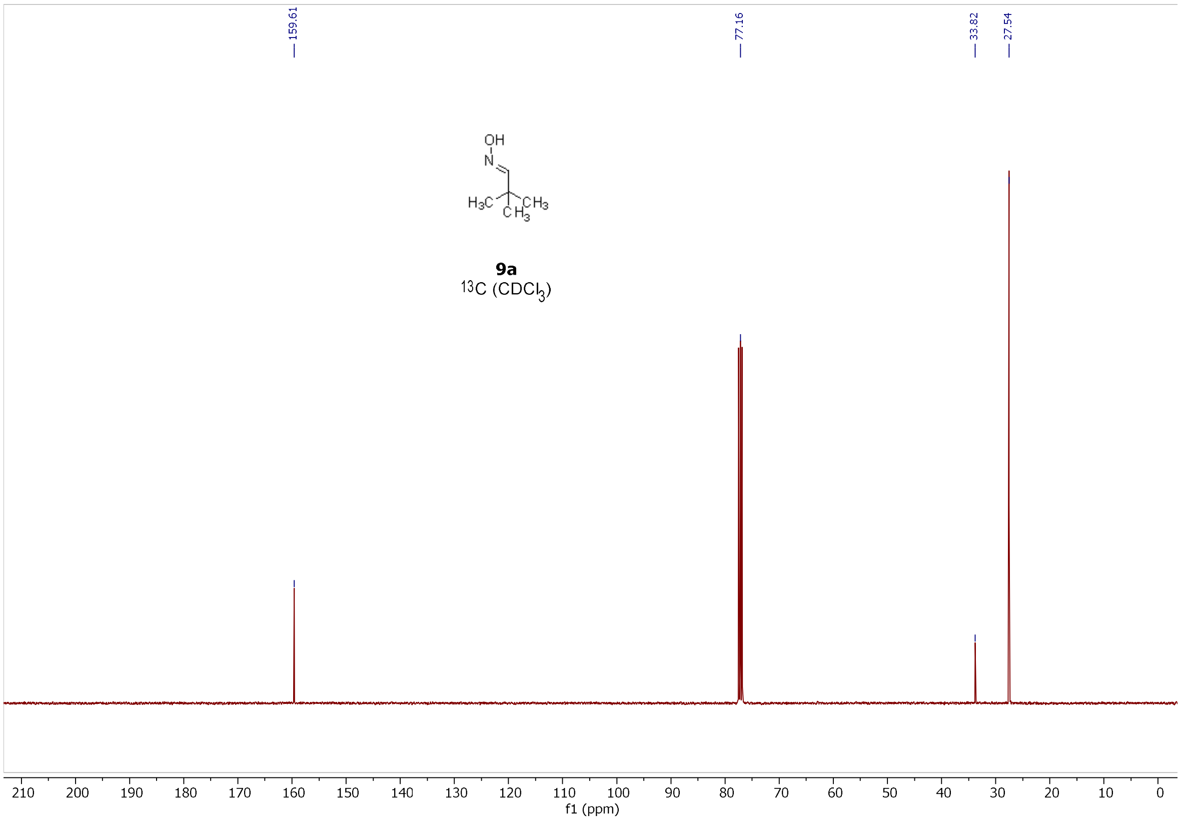

Figure S30. ^{13}C NMR spectrum of **9a.**

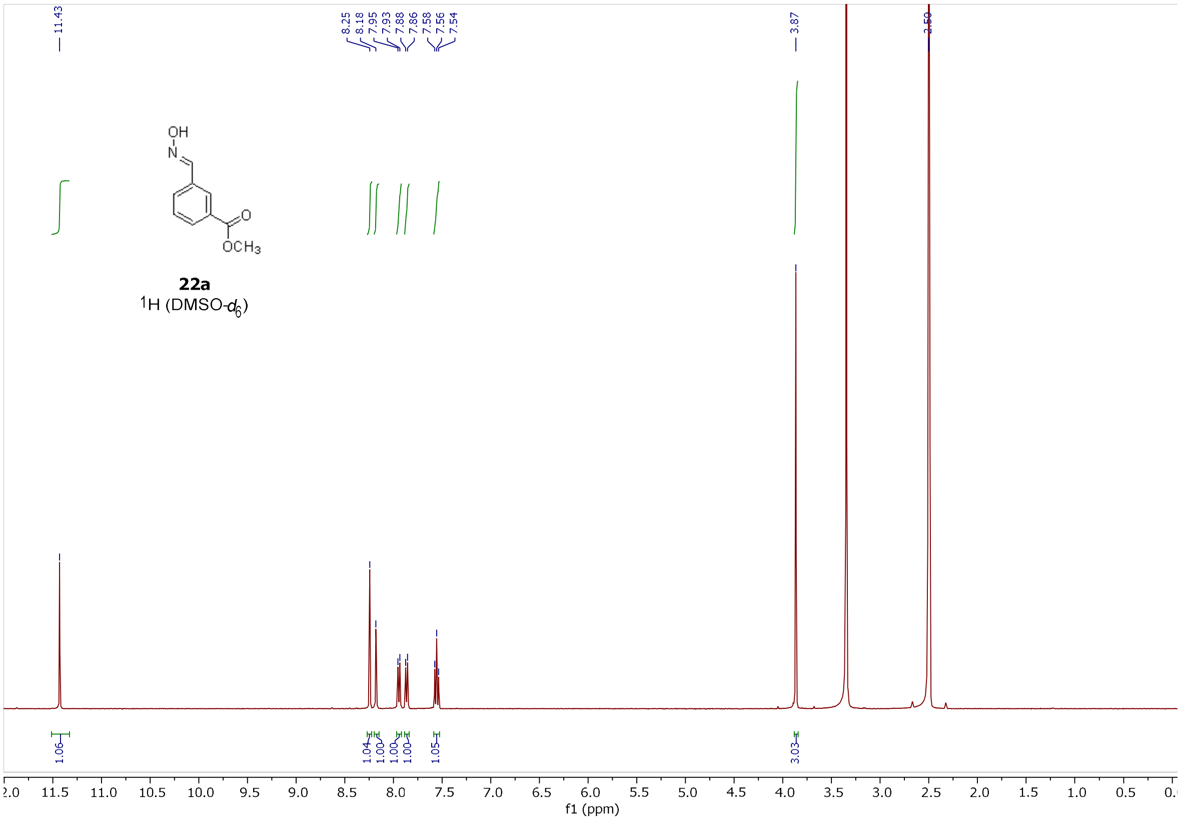

Figure S31. ^{1}H NMR spectrum of **22a.**

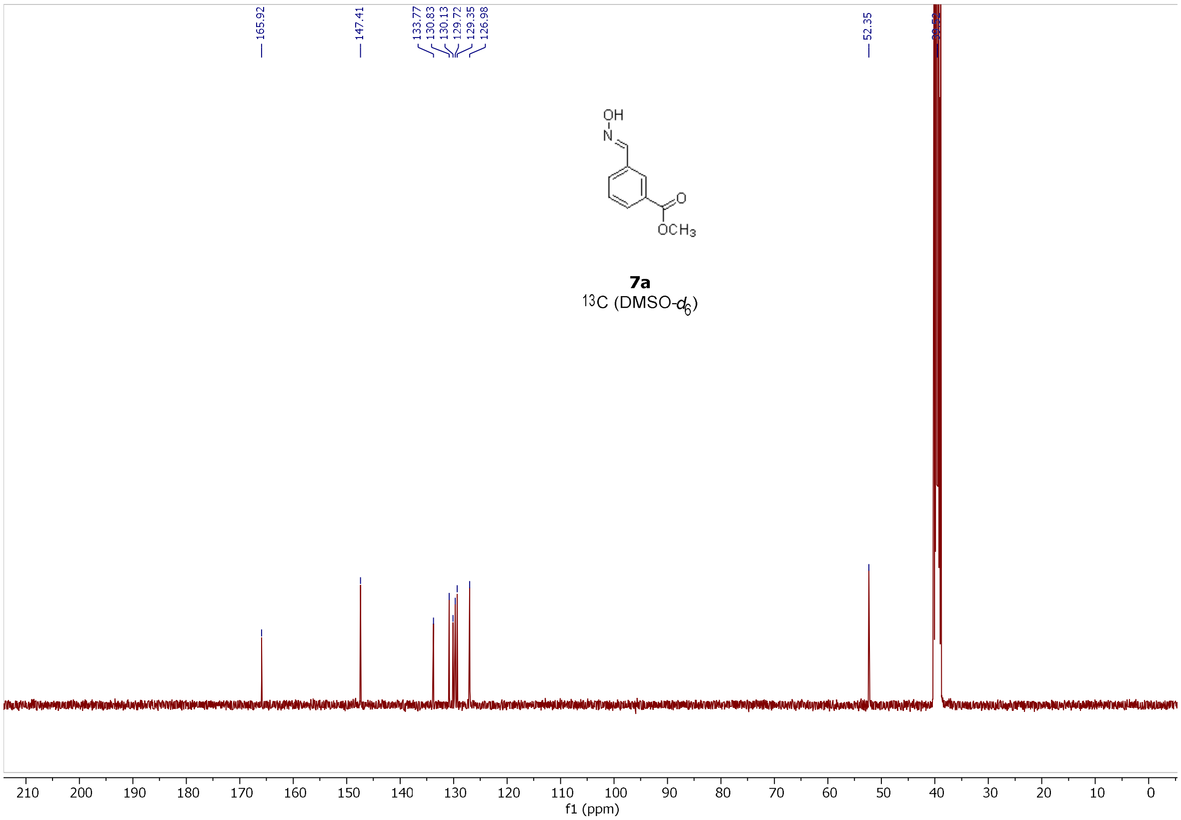

Figure S32. ^{13}C NMR spectrum of **7a.**

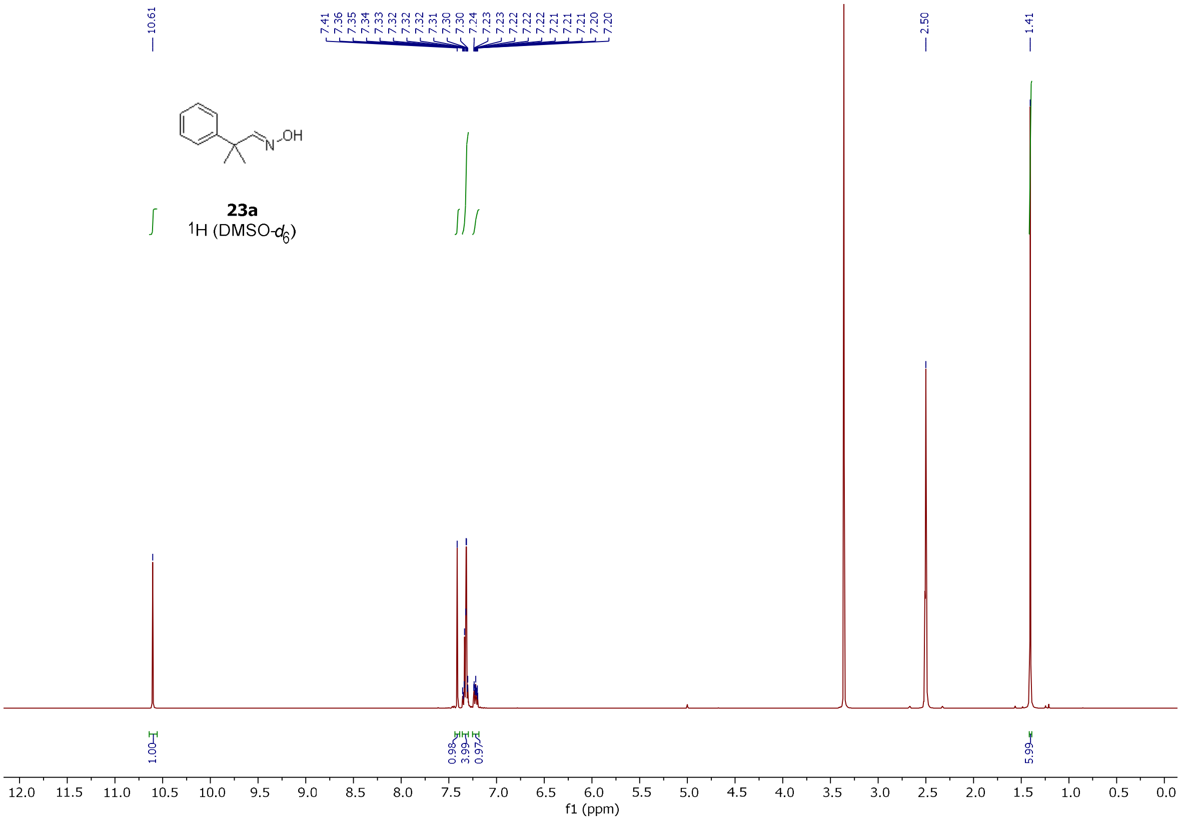

Figure S33. ^{1}H NMR spectrum of **23a.**

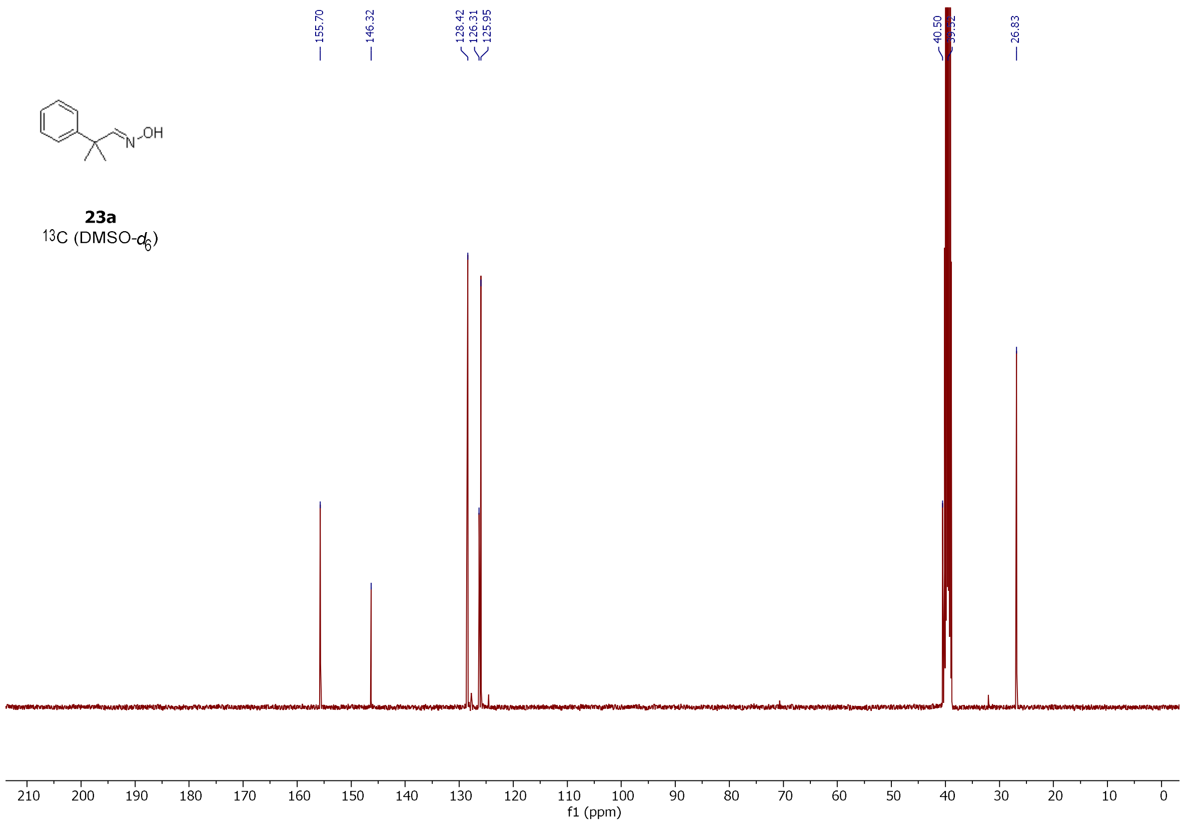

Figure S34. ^{13}C NMR spectrum of **23a.**

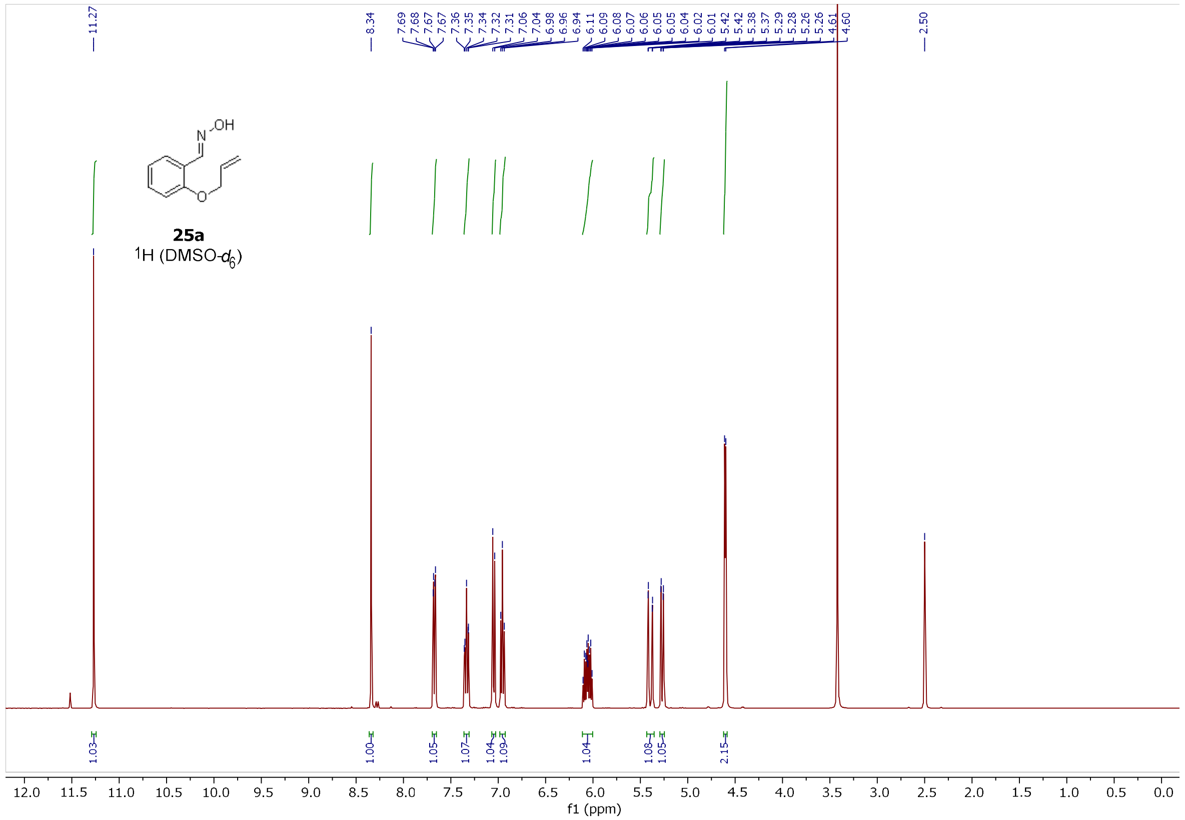

Figure S35. ^{1}H NMR spectrum of **25a.**

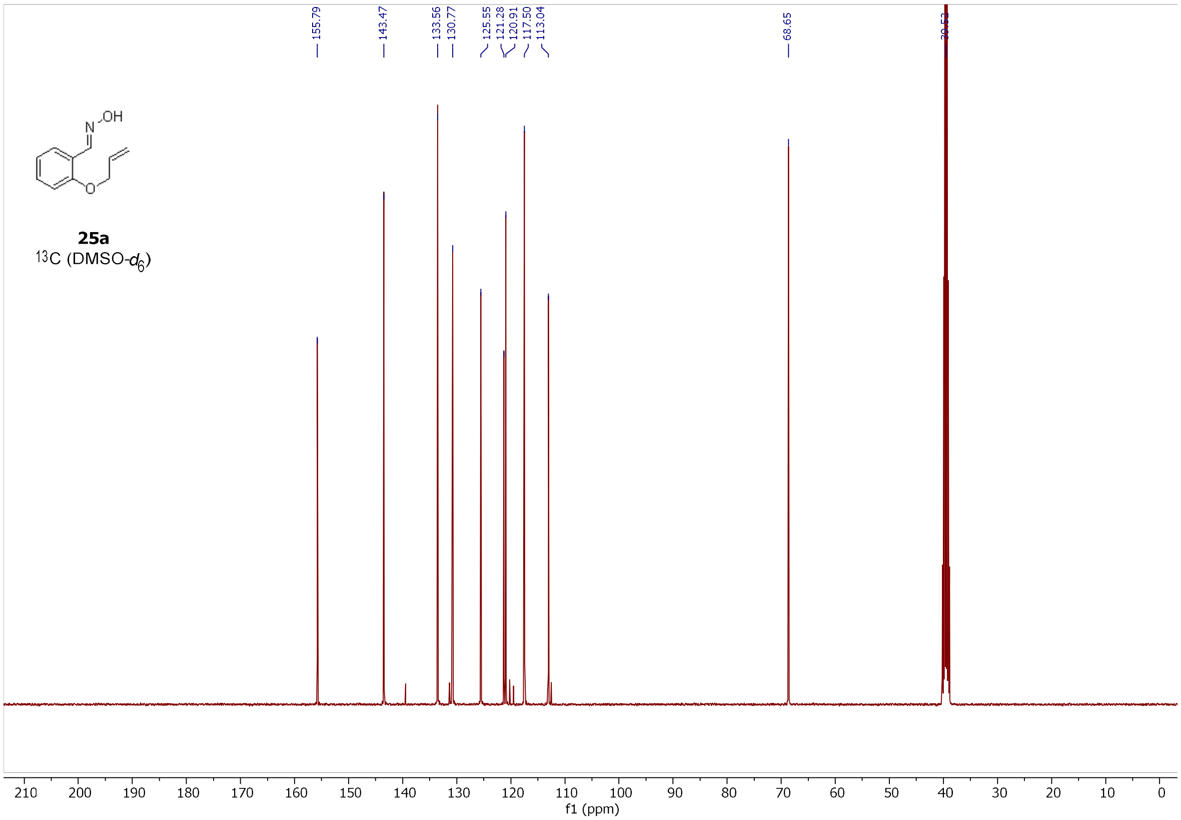

Figure S36. ^{13}C NMR spectrum of **25a.**

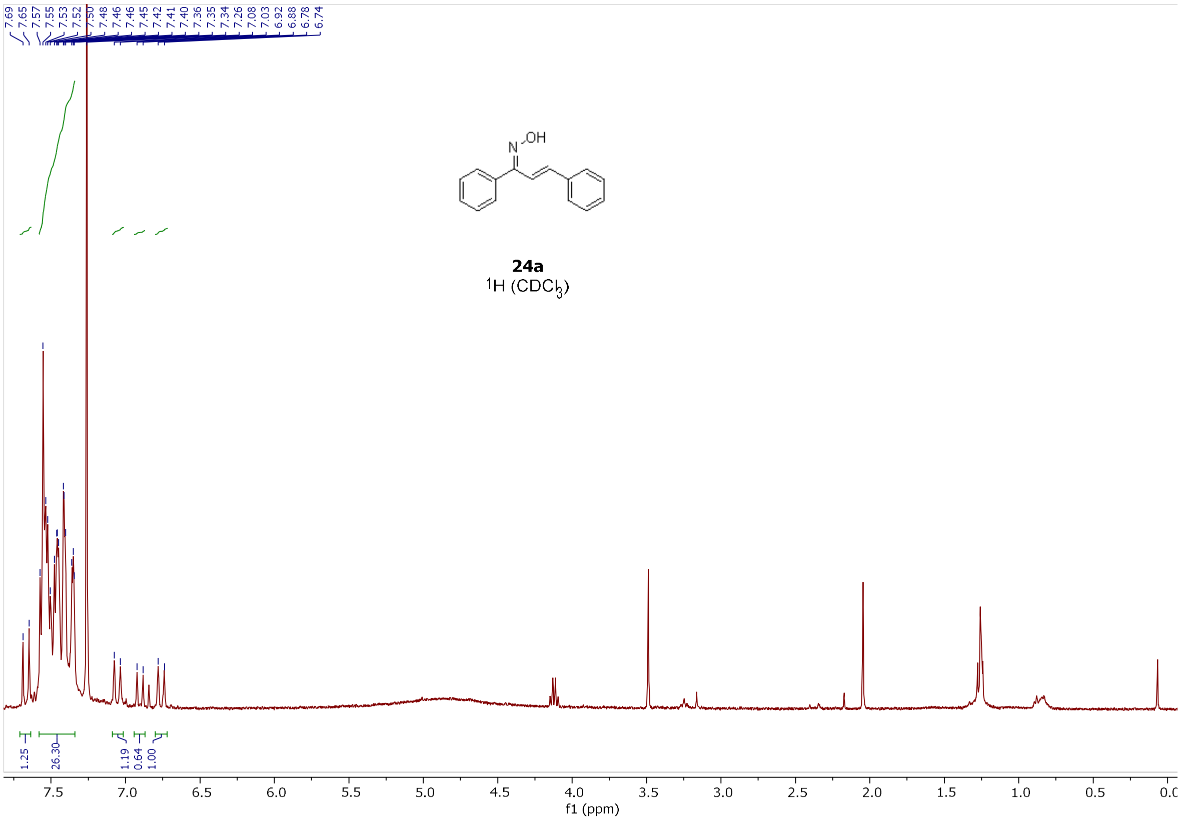

Figure S37. ^{1}H NMR spectrum of **24a.**

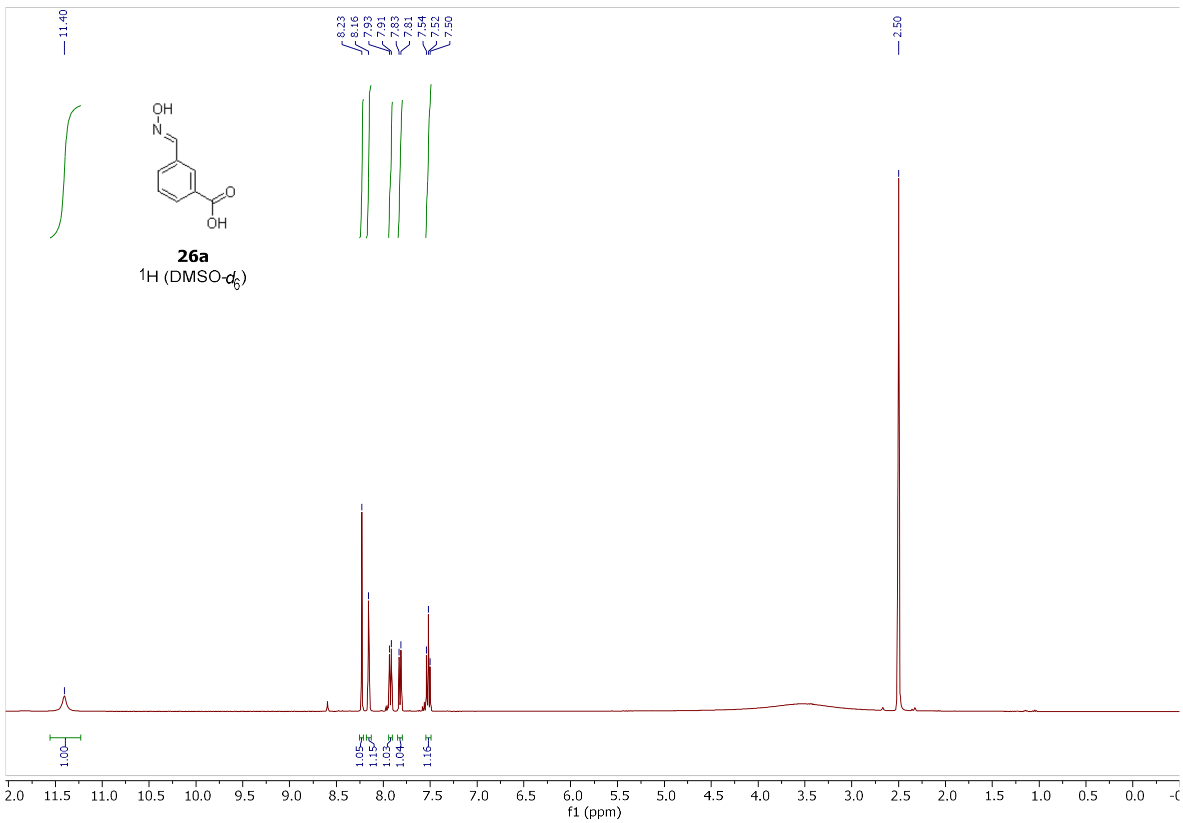

Figure S38. ¹H NMR spectrum of **26a.**

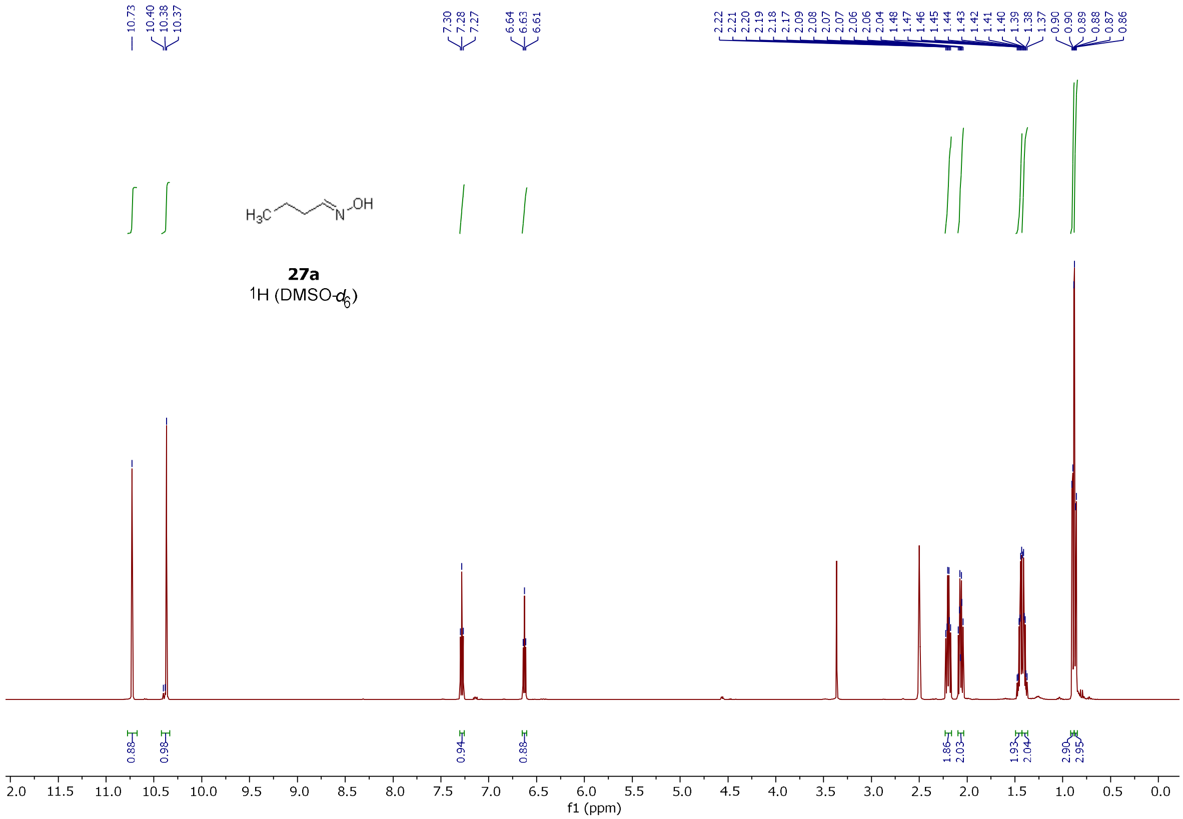

Figure S39. ¹H NMR spectrum of **27a.**

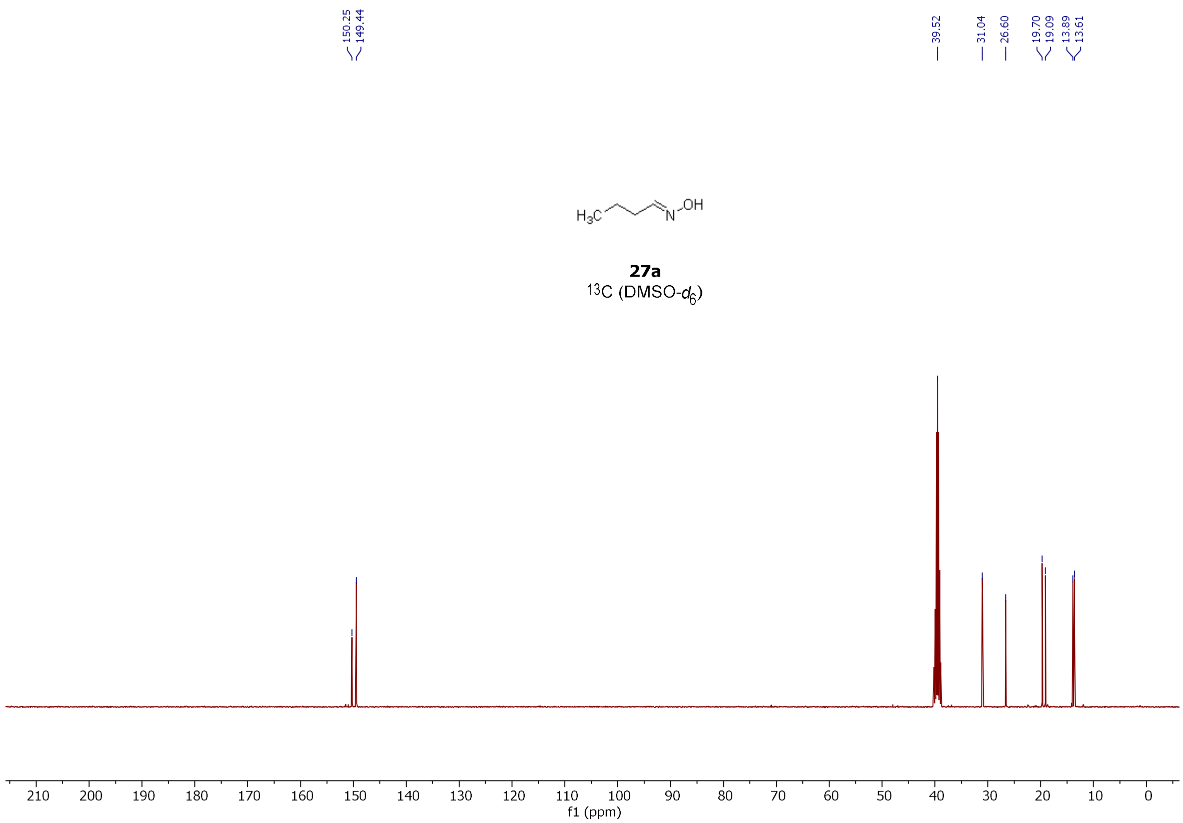

Figure S40. ^{13}C NMR spectrum of **27a.**

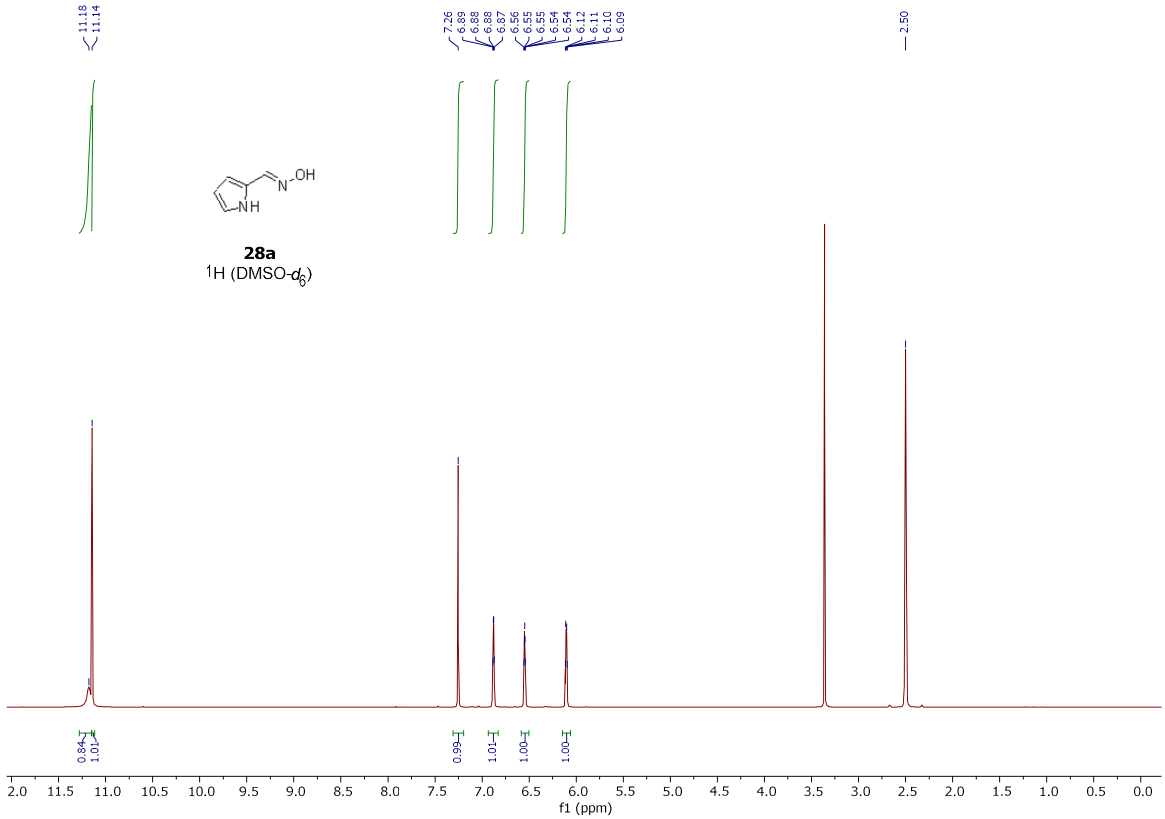

Figure S41. ^{1}H NMR spectrum of **28a.**

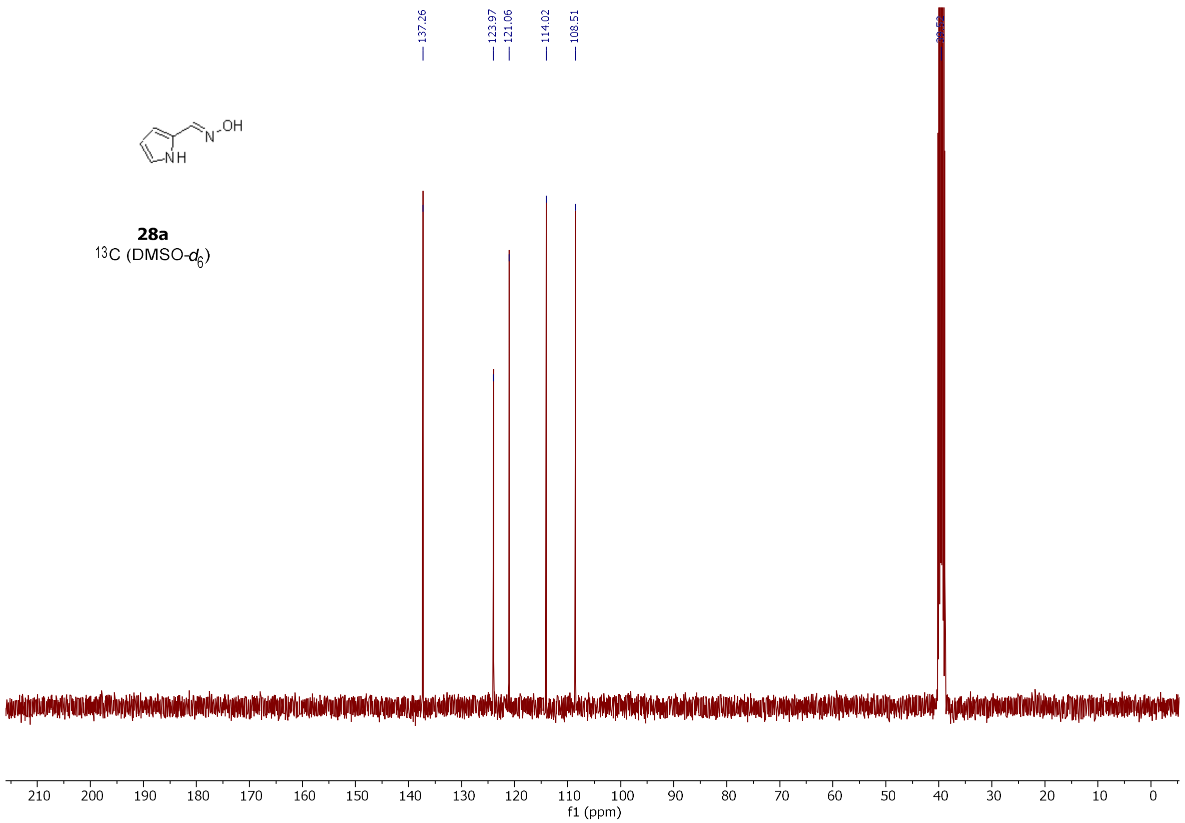

Figure S42. ¹³C NMR spectrum of **28a.**

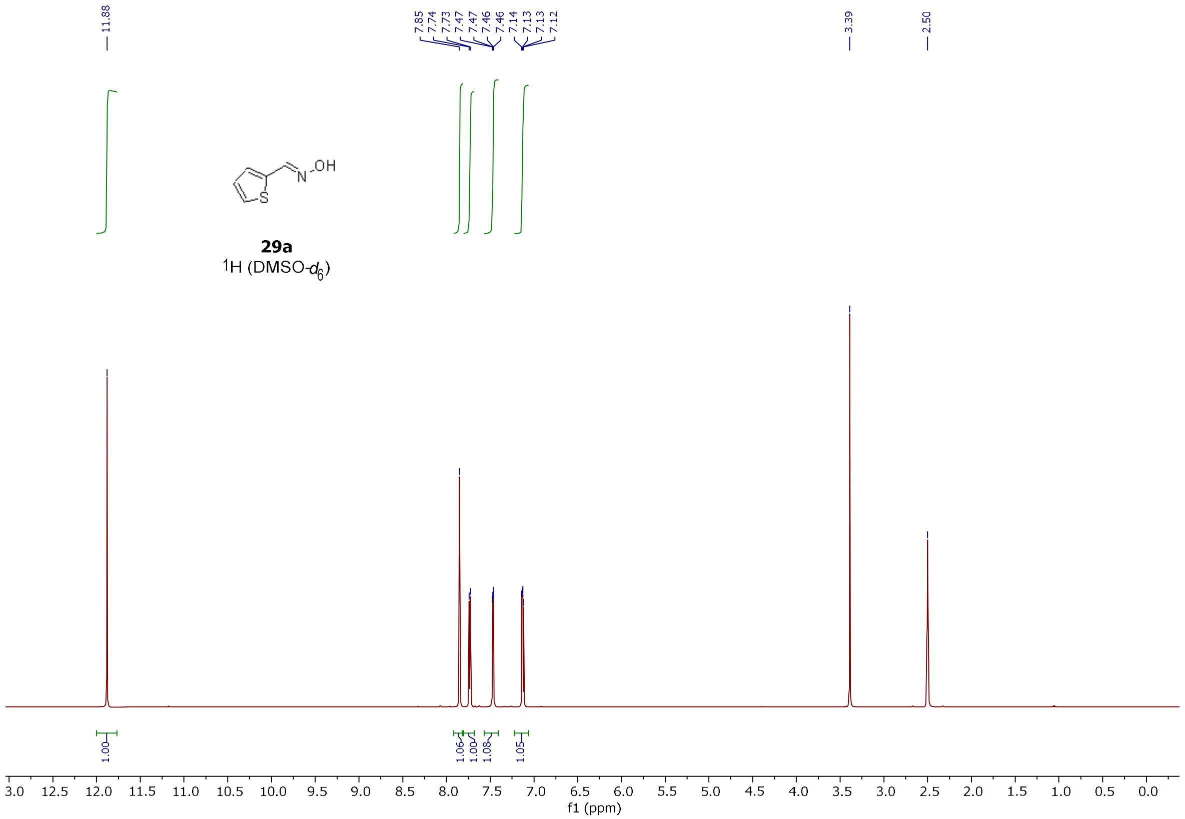

Figure S43. ¹H NMR spectrum of **29a.**

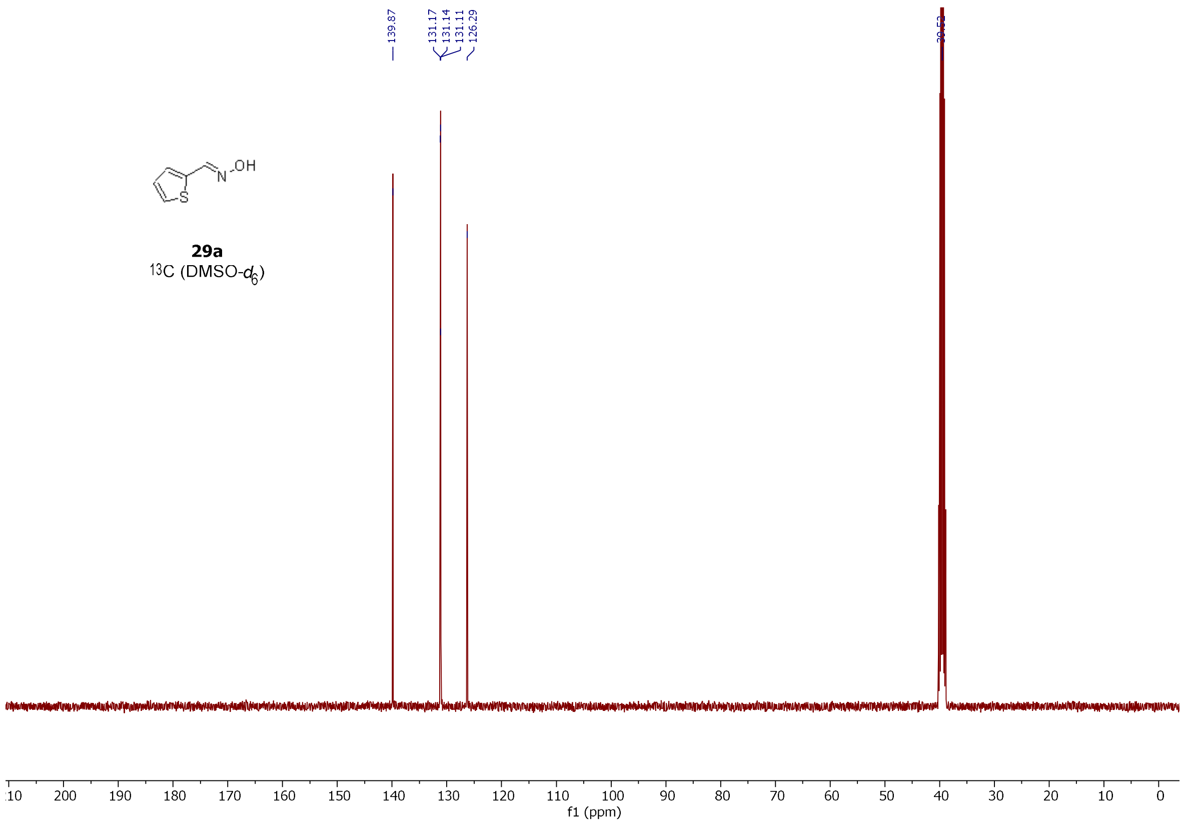

Figure S44. ^{13}C NMR spectrum of **29a**.

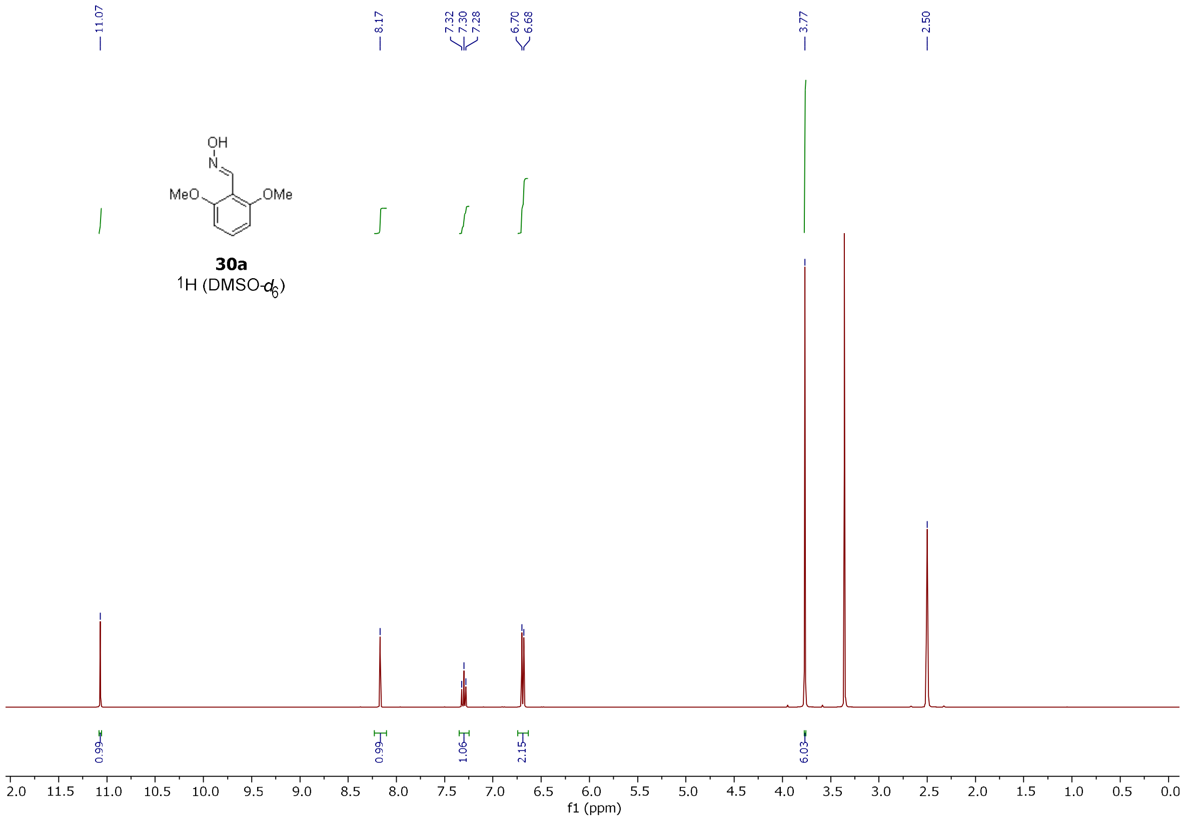

Figure S45. ^{1}H NMR spectrum of **30a**.

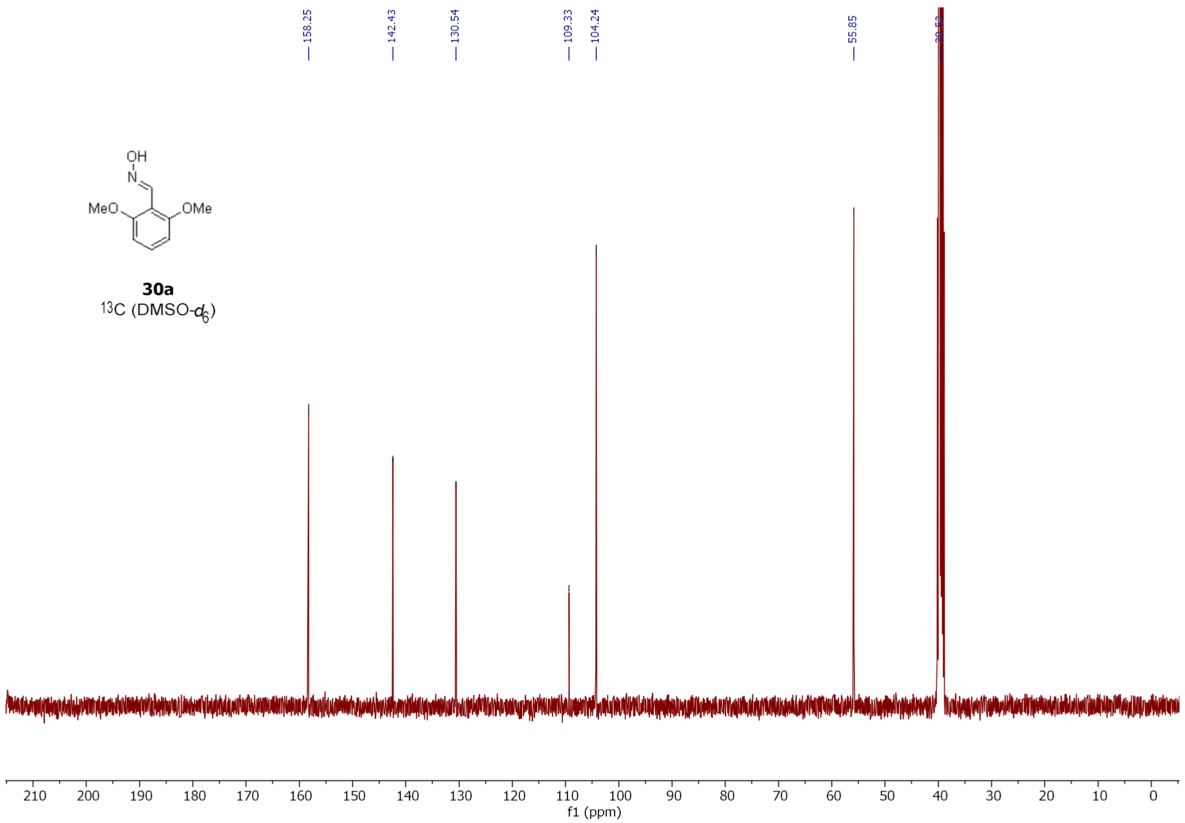

Figure S46. ^{13}C NMR spectrum of **30a.**

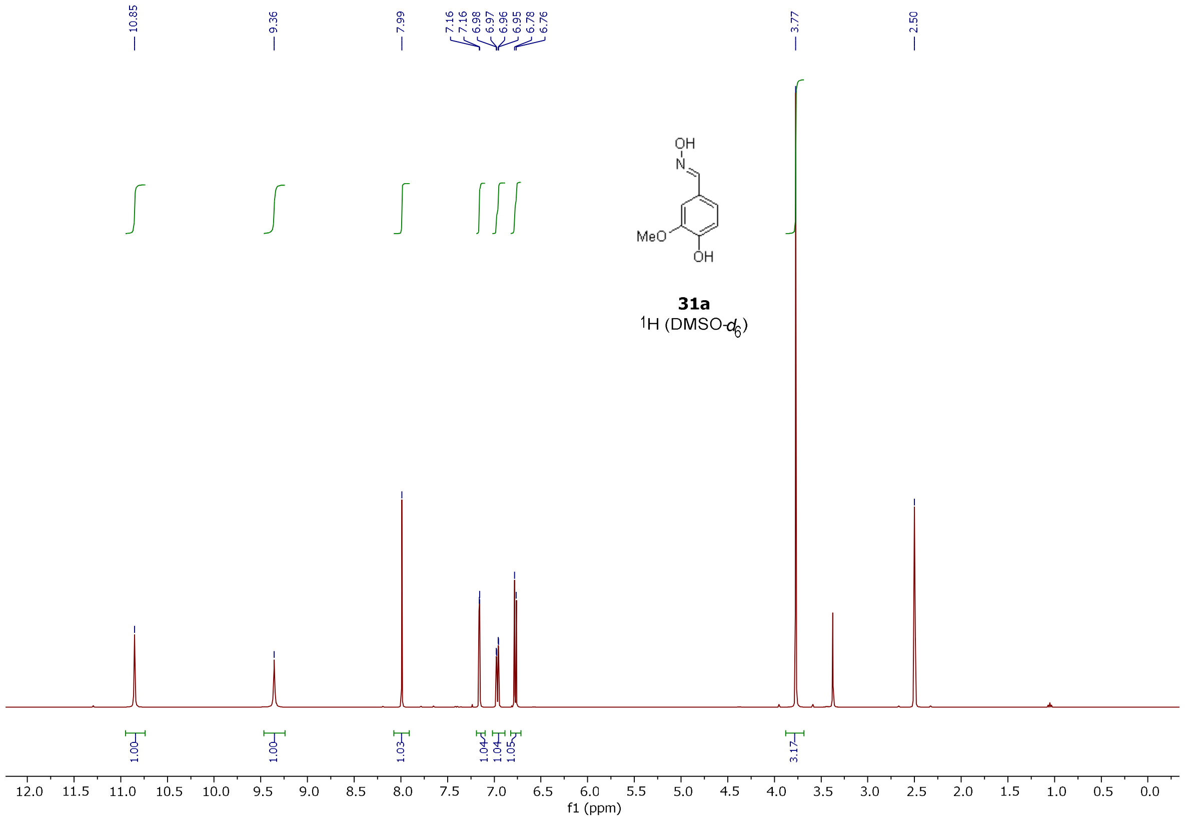

Figure S47. ^{1}H NMR spectrum of **31a.**

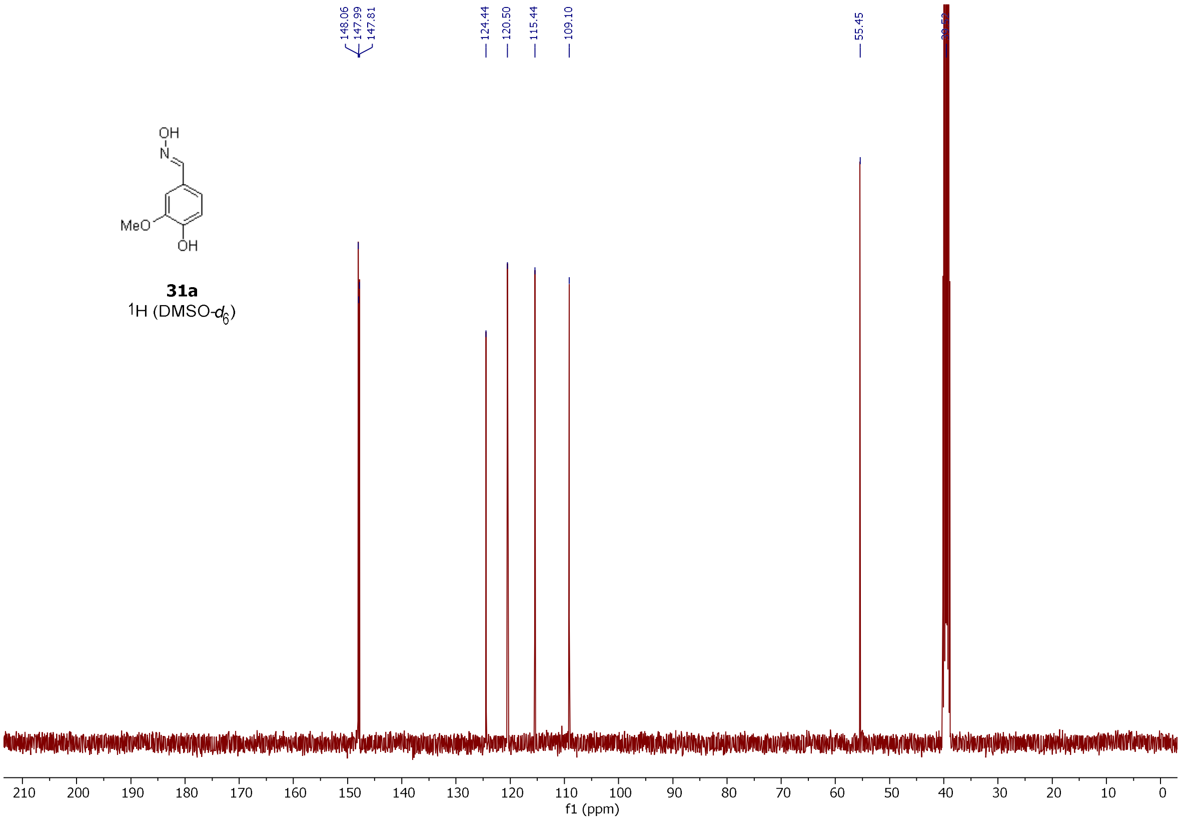

Figure S48. ^{13}C NMR spectrum of **31a**.

5.2. NMR spectra of N-hydroxybenzimidoyl chlorides and nitrile oxide

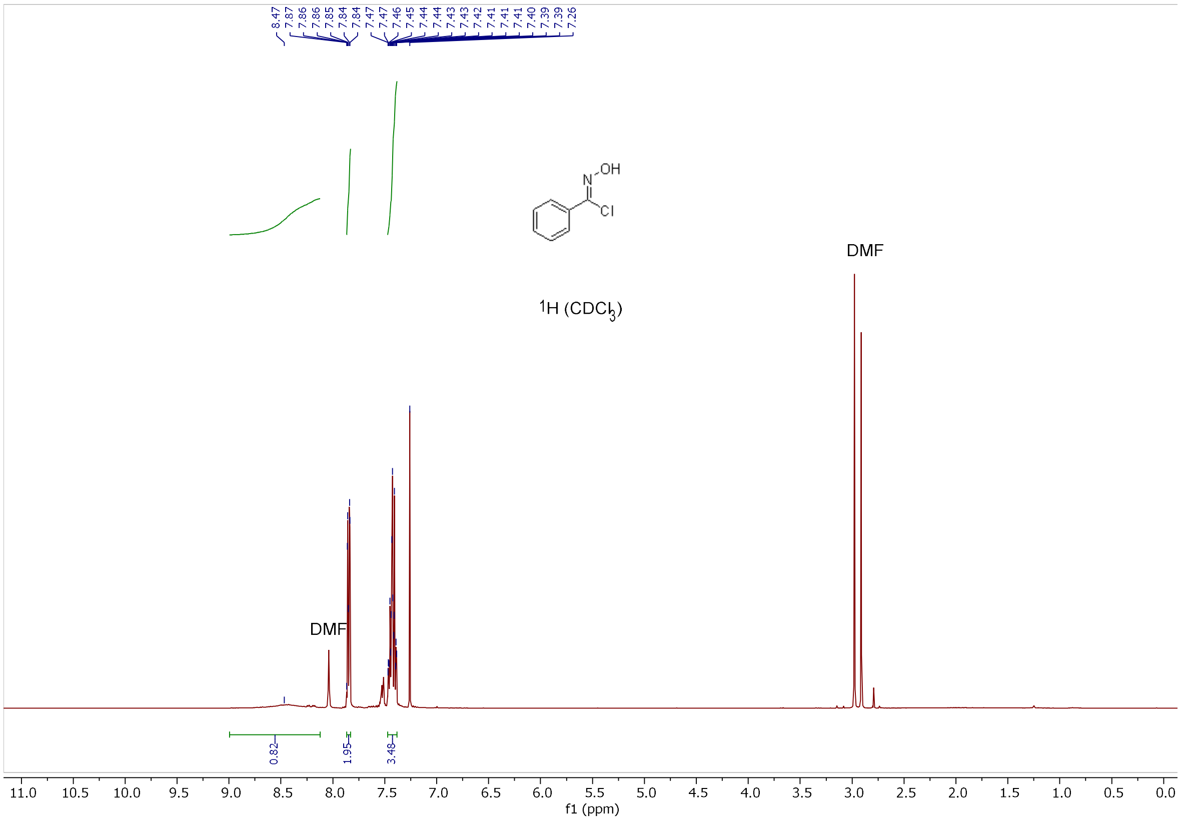

Figure S49. ^{1}H NMR spectrum of **N-hydroxybenzimidoyl chloride**.

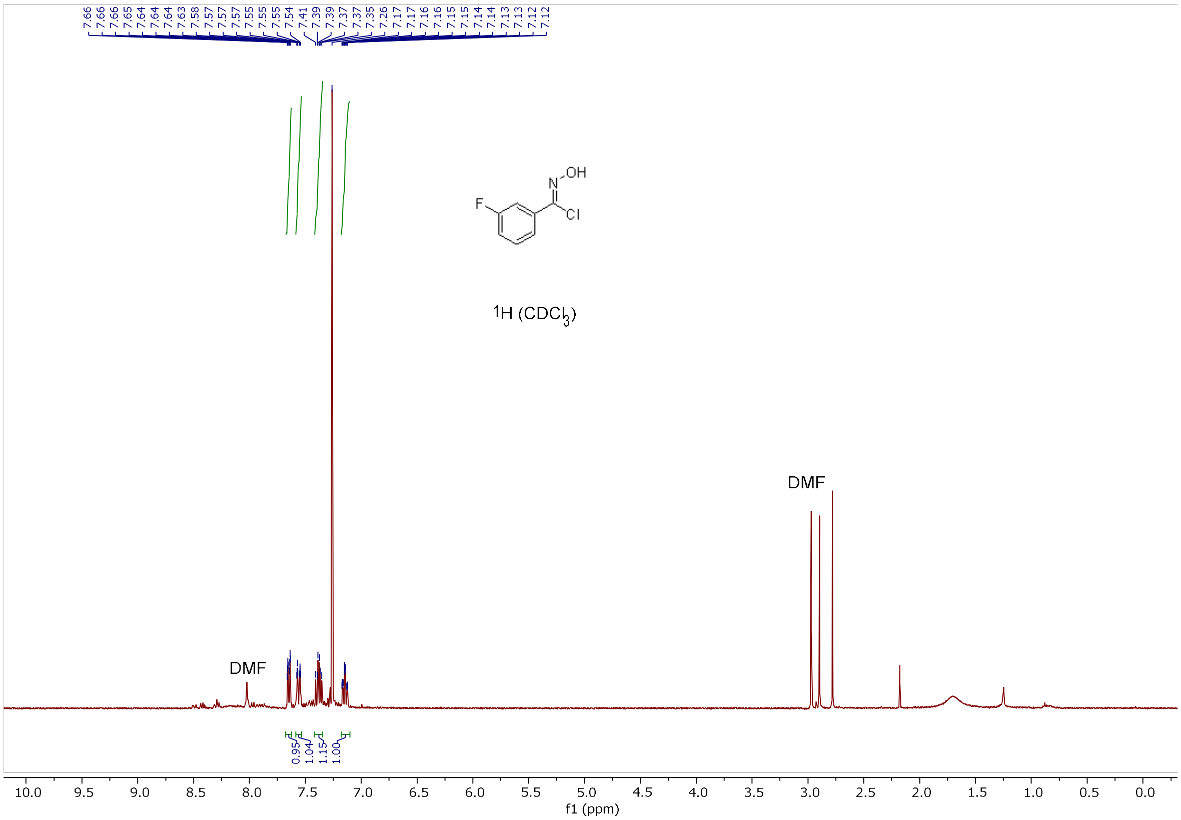

Figure S50. ^{1}H NMR spectrum of **3-fluoro-*N*-hydroxybenzimidoyl chloride**.

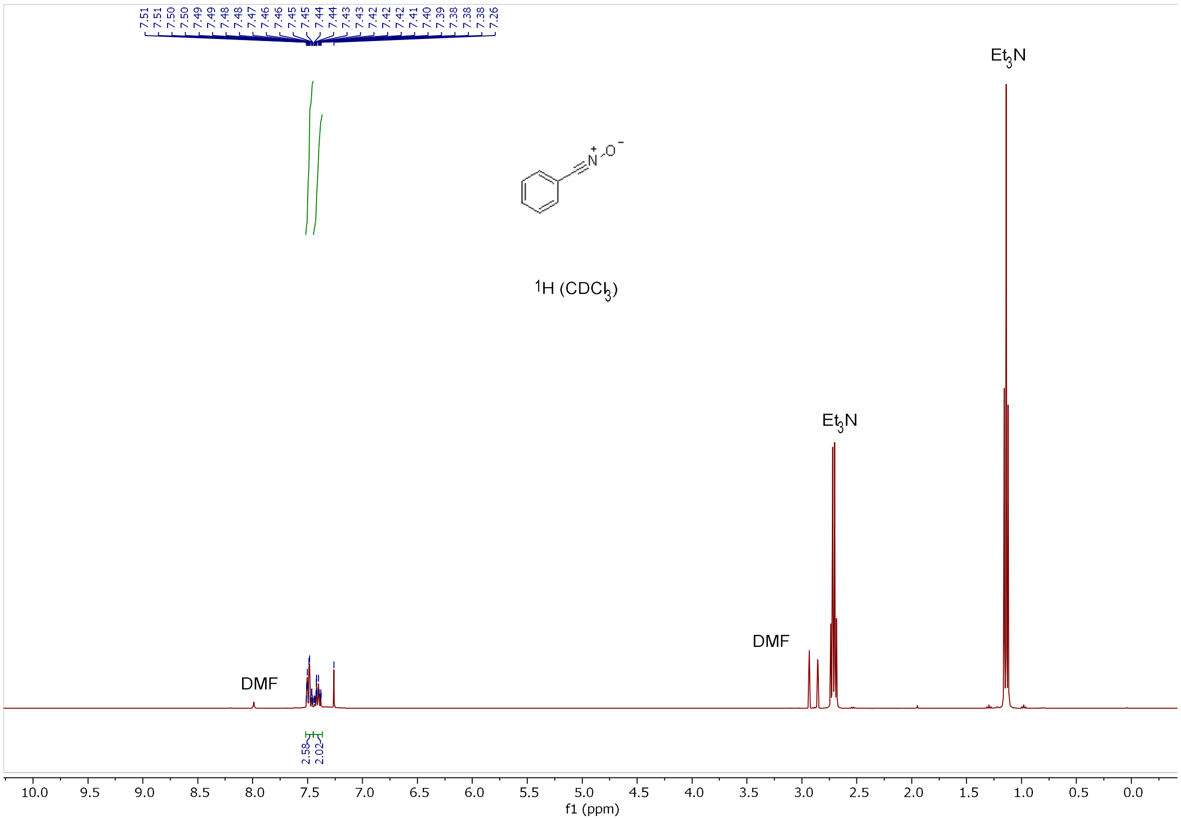

Figure S51. ^{1}H NMR spectrum of **nitrile oxide**.

5.3. NMR spectra of oxadiazoles-1,2,4 1-23 and isoxazoles 24,25

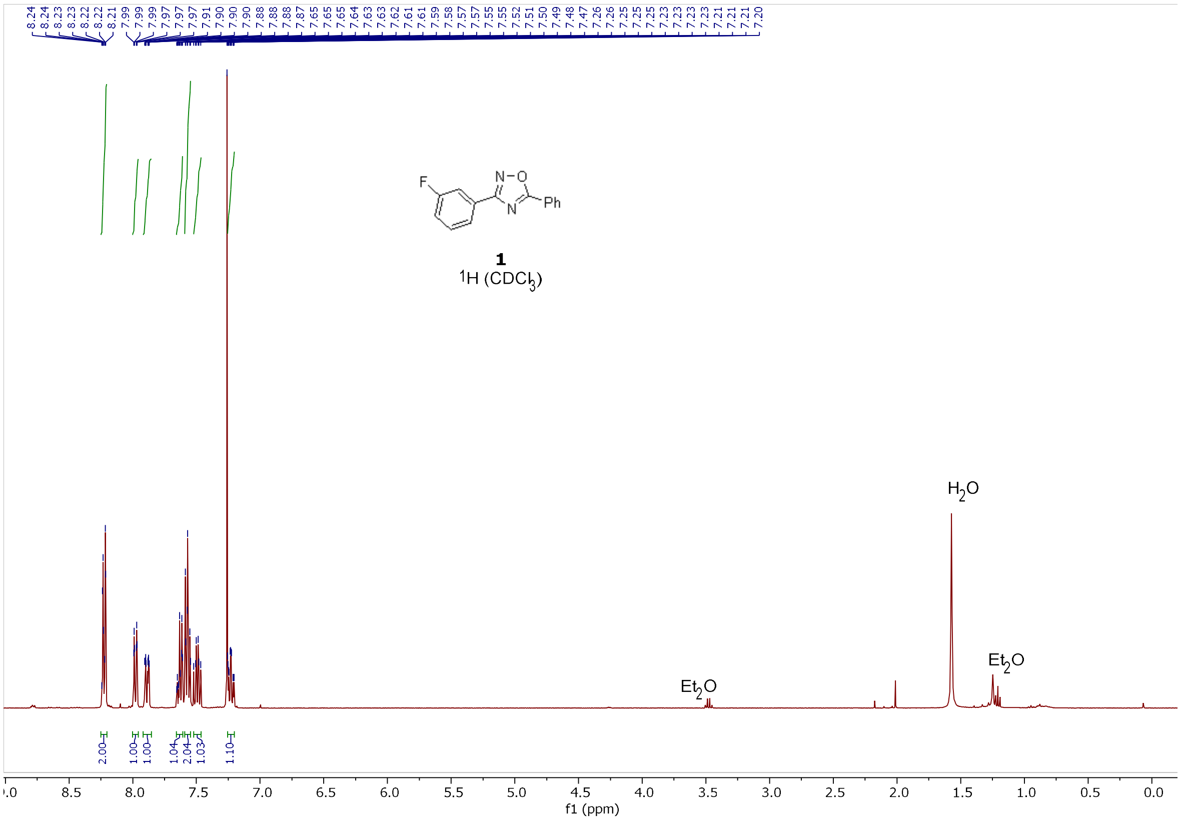

Figure S52. ^{1}H NMR spectrum of **1.** (3-(3-fluorophenyl)-5-phenyl-1,2,4-oxadiazole)**.**

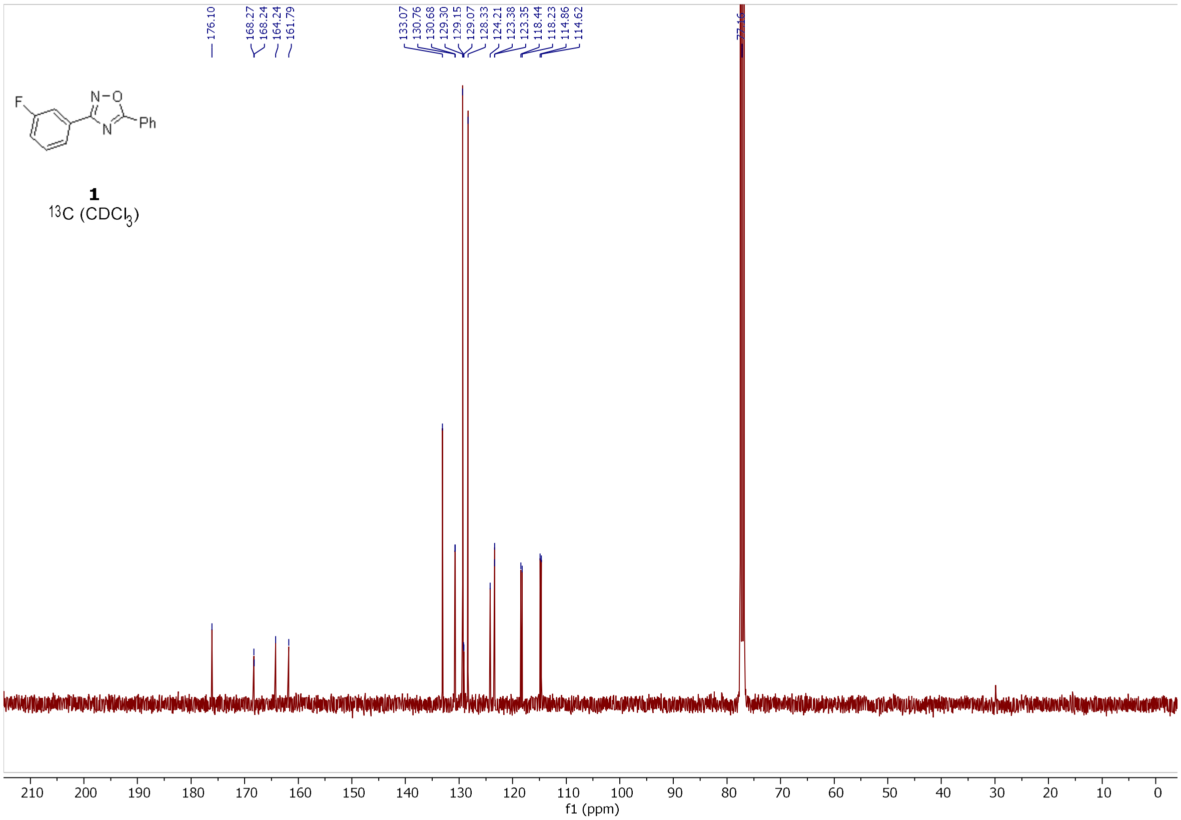

Figure S53. ^{13}C NMR spectrum of **1**. (3-(3-fluorophenyl)-5-phenyl-1,2,4-oxadiazole).

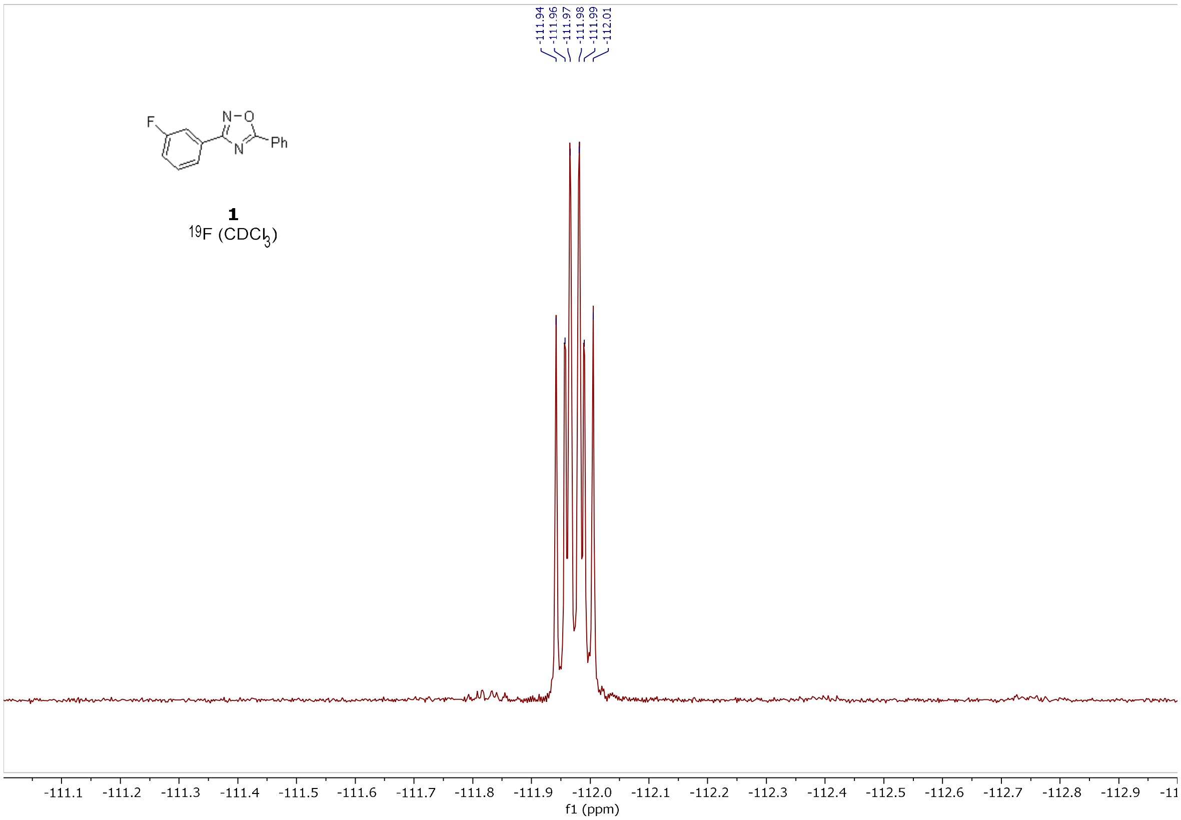

Figure S54. ^{19}F NMR spectrum of **1**. (3-(3-fluorophenyl)-5-phenyl-1,2,4-oxadiazole).

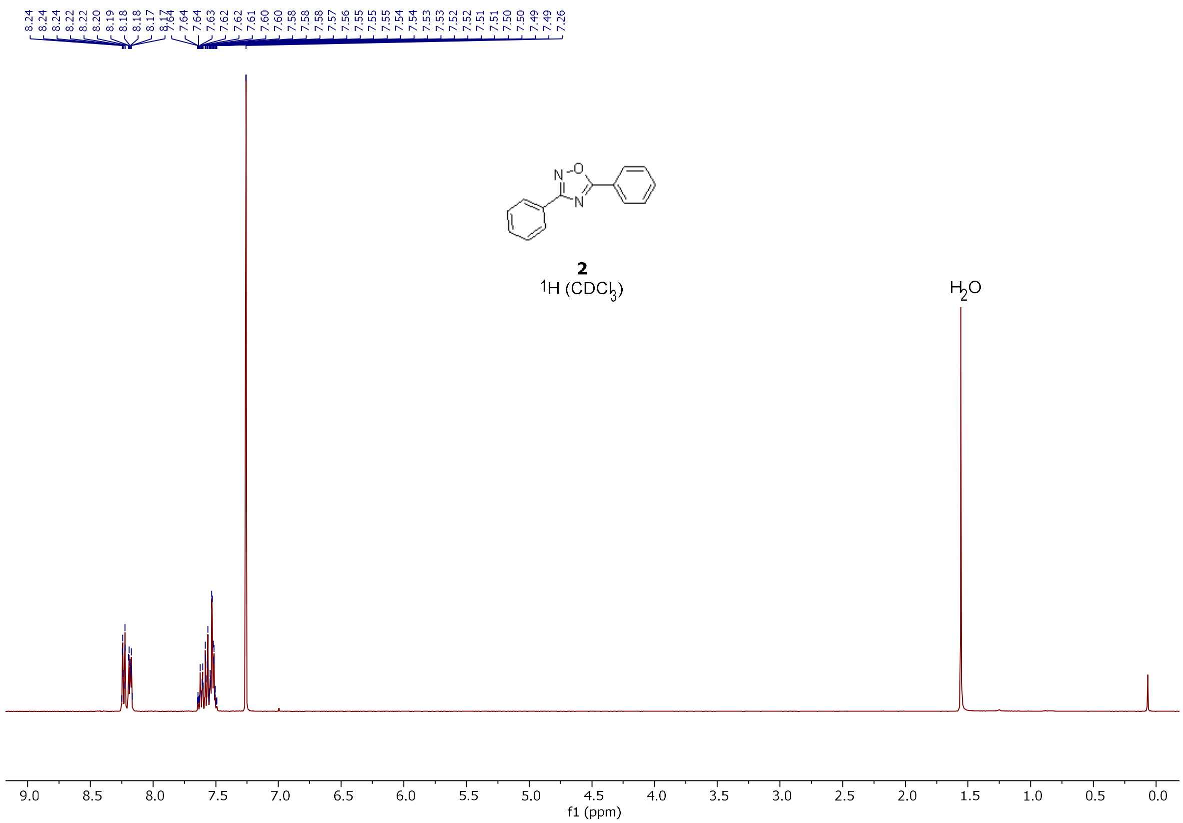

Figure S55. ^{1}H NMR spectrum of **2**. (3,5-diphenyl-1,2,4-oxadiazole).

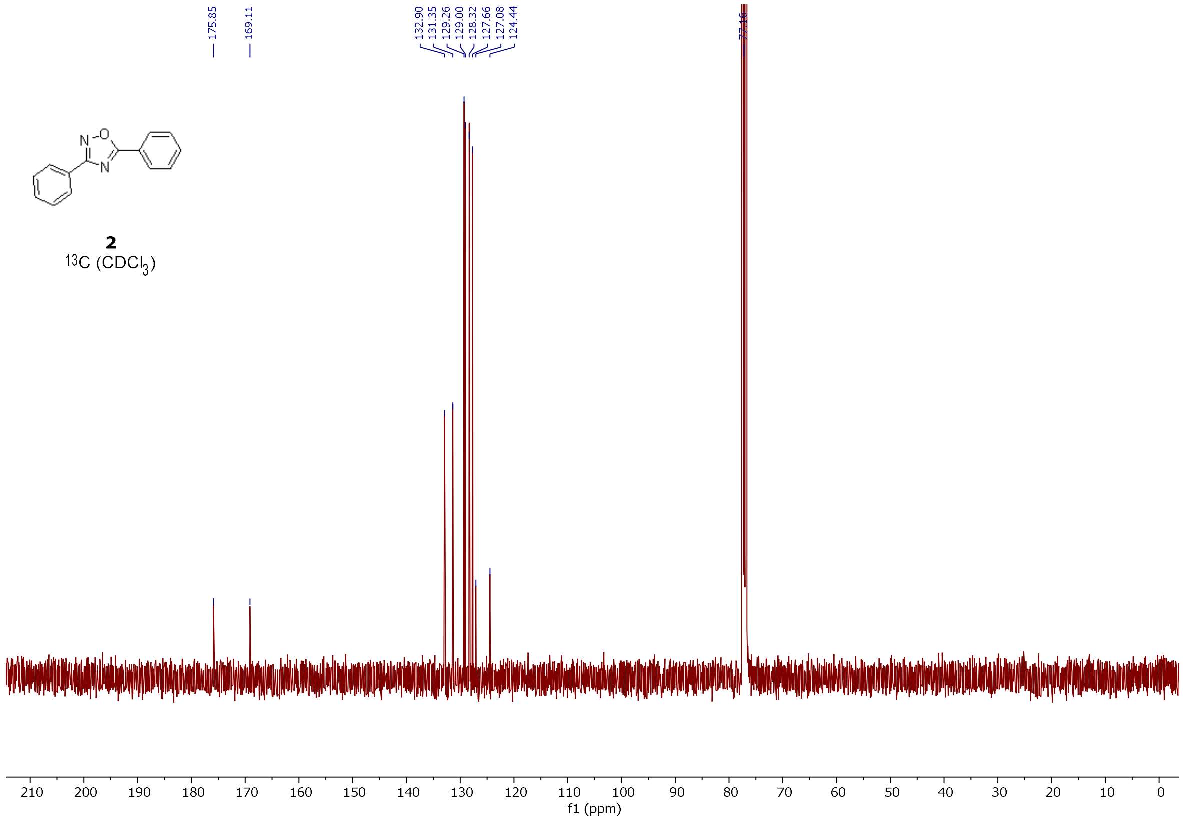

Figure S56. ^{13}C NMR spectrum of **2**. (3,5-diphenyl-1,2,4-oxadiazole).

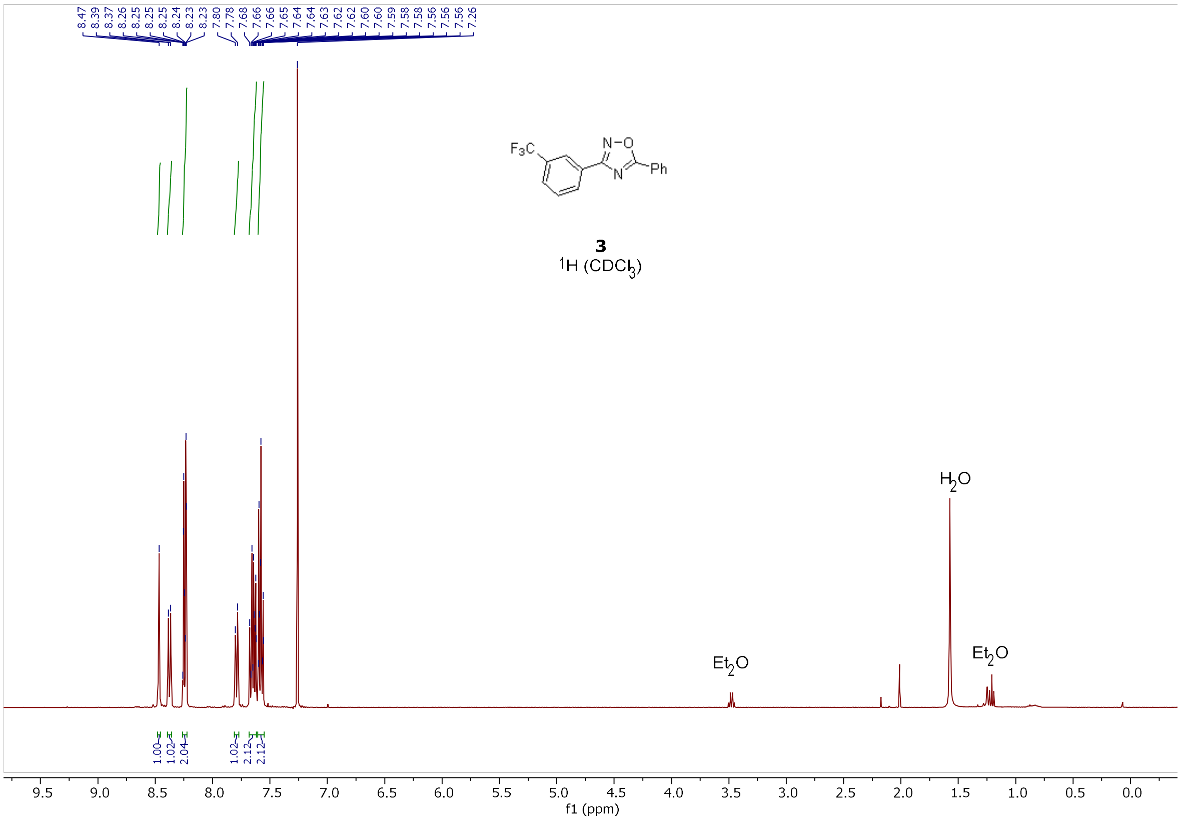

Figure S57. ^{1}H NMR spectrum of **3**. (5-phenyl-3-(3-(trifluoromethyl)phenyl)-1,2,4-oxadiazole).

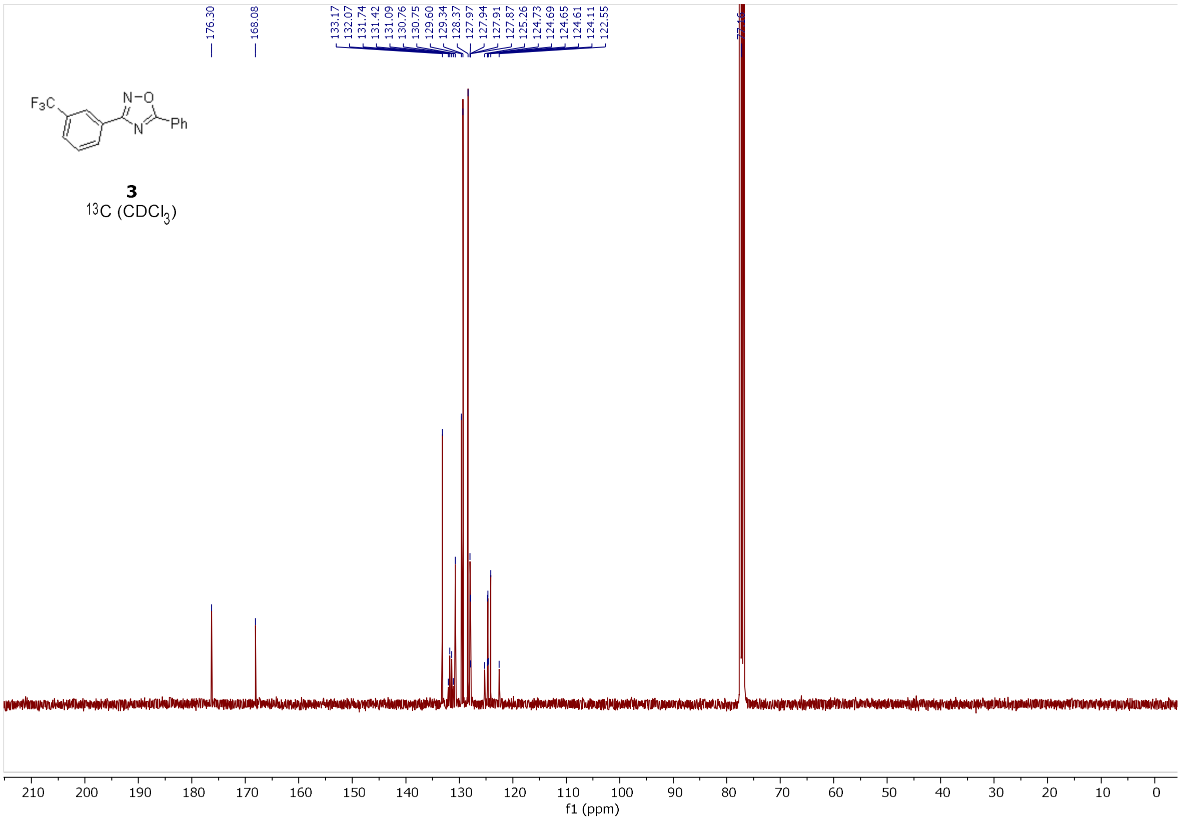

Figure S58. ^{13}C NMR spectrum of **3**. (5-phenyl-3-(3-(trifluoromethyl)phenyl)-1,2,4-oxadiazole).

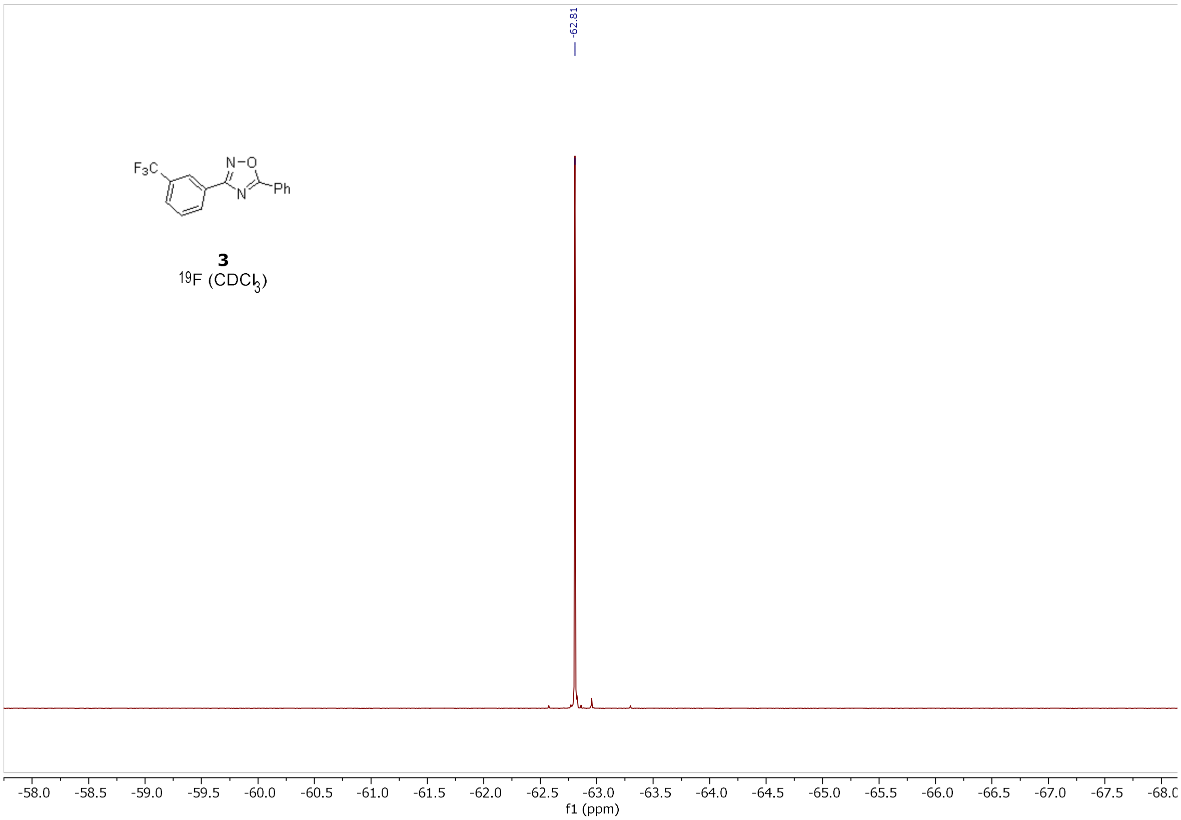

Figure S59. ^{19}F NMR spectrum of **3**. (5-phenyl-3-(3-(trifluoromethyl)phenyl)-1,2,4-oxadiazole)

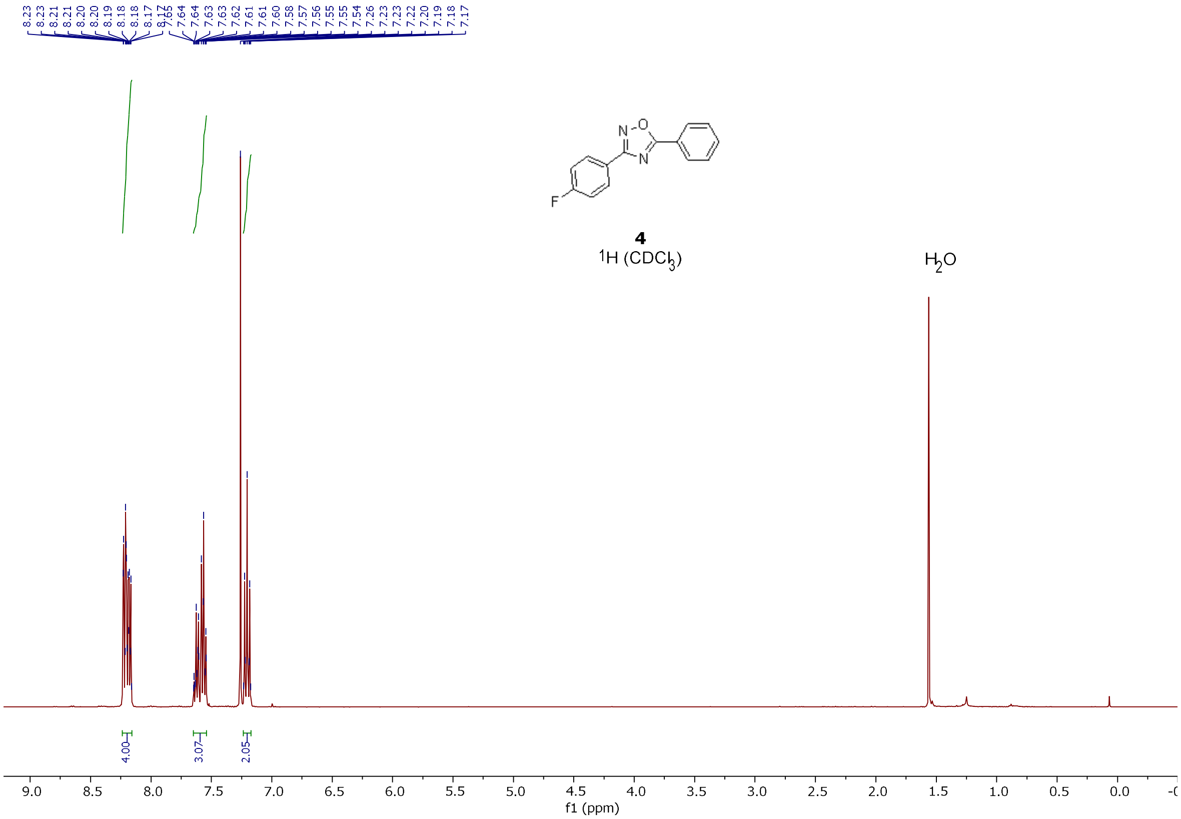

Figure S60. ^{1}H NMR spectrum of **4**.

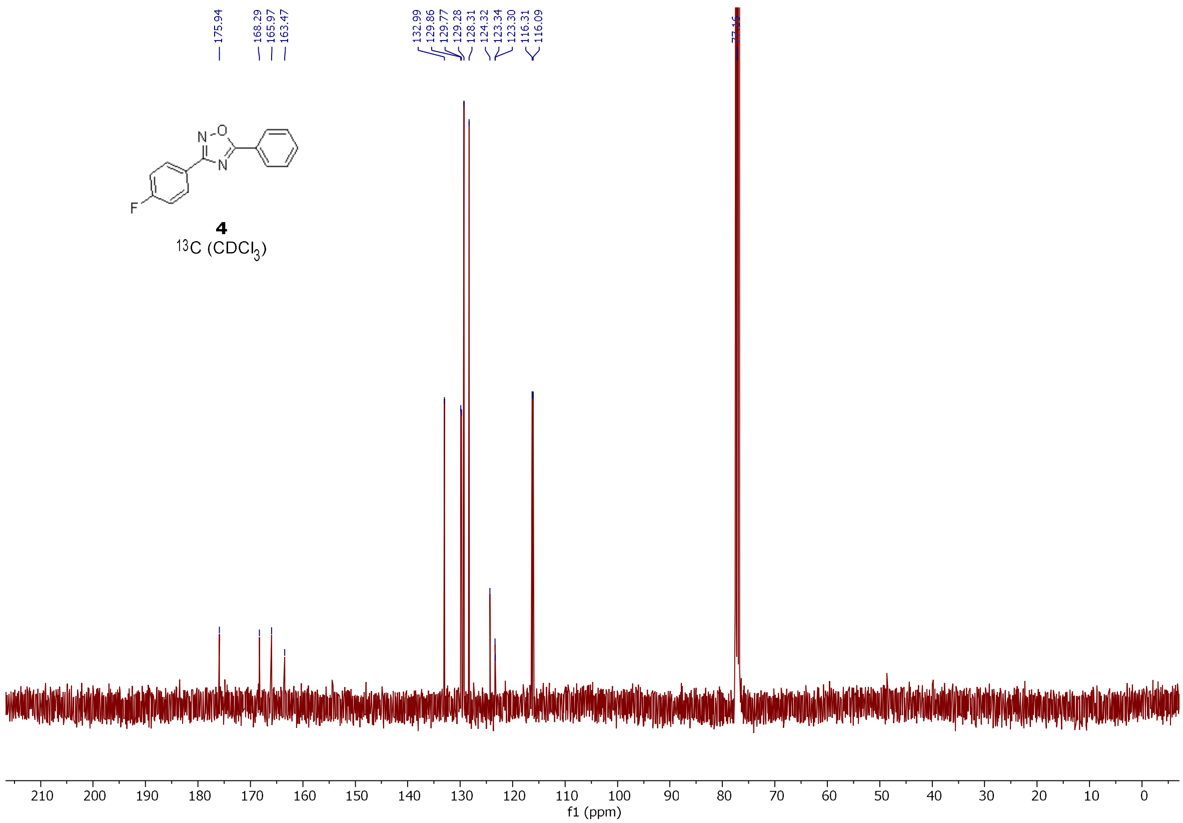

Figure S61. ^{13}C NMR spectrum of **4.**

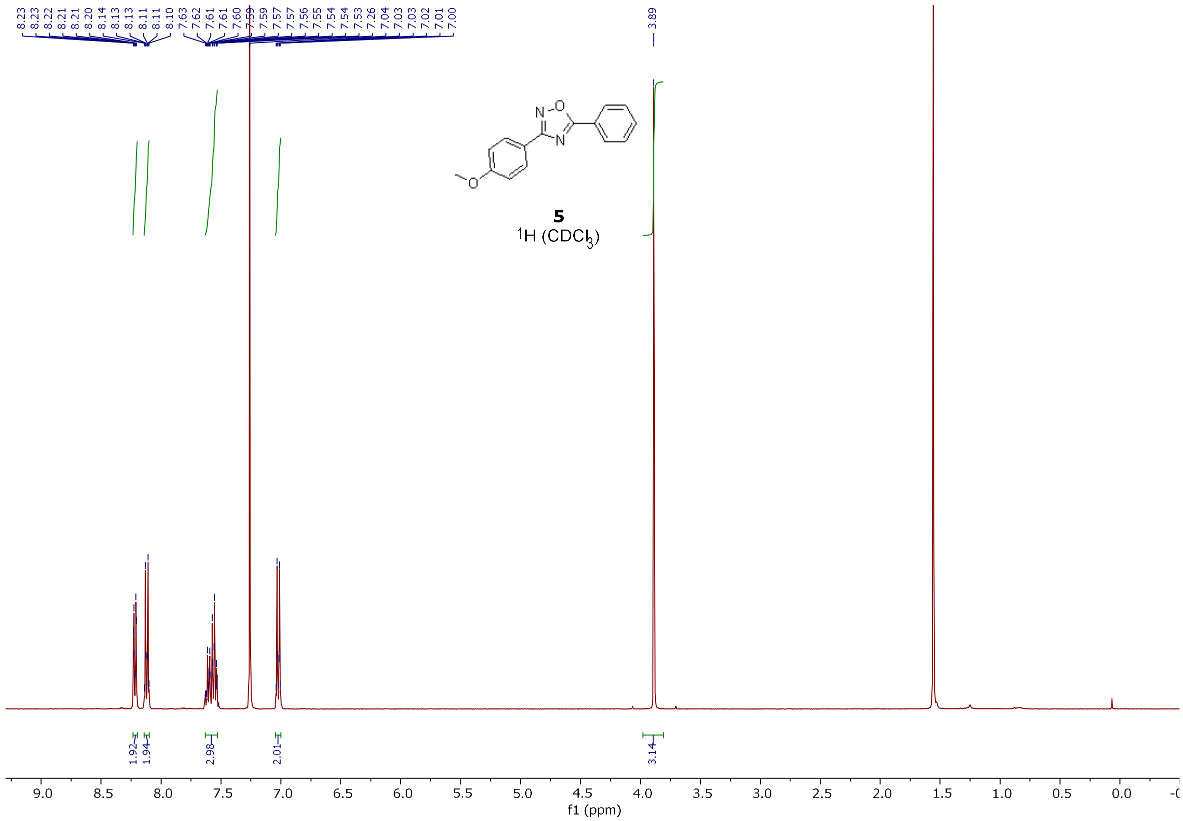

Figure S62. ^{1}H NMR spectrum of **5**.

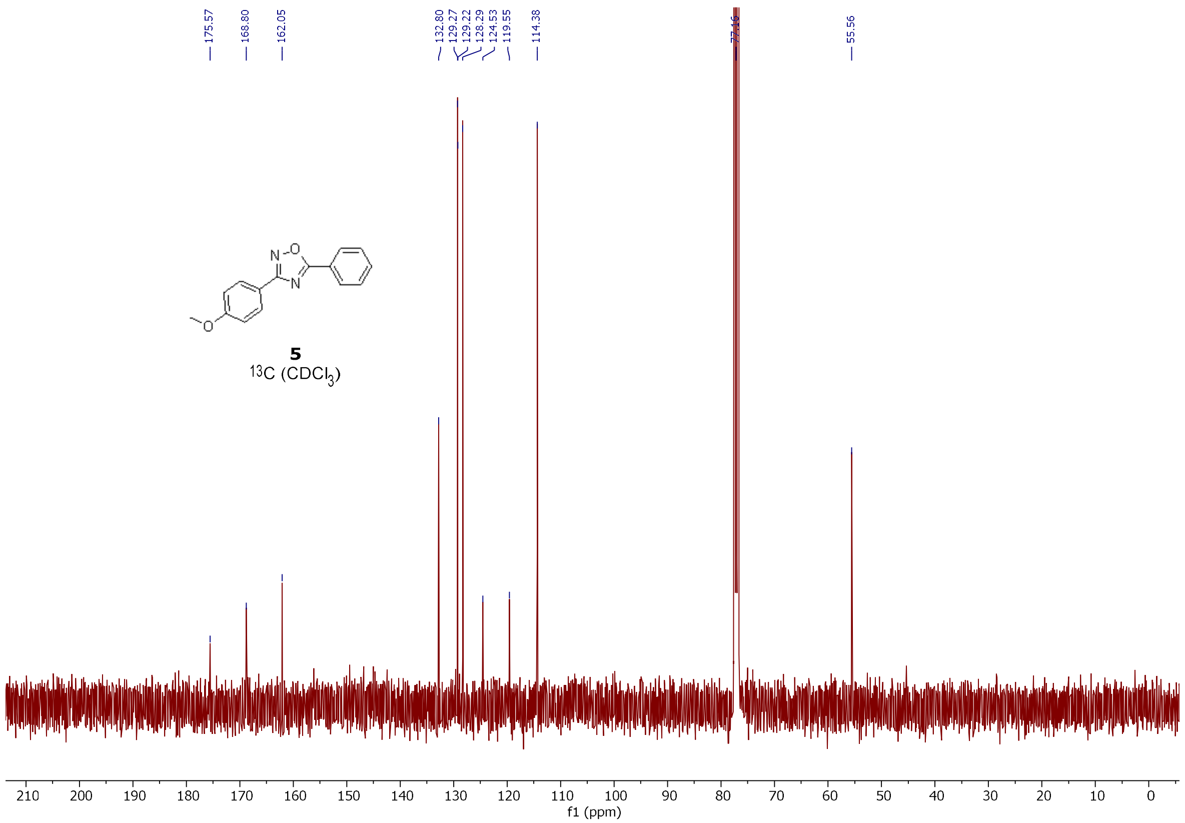

Figure S63. ^{13}C NMR spectrum of **5**.

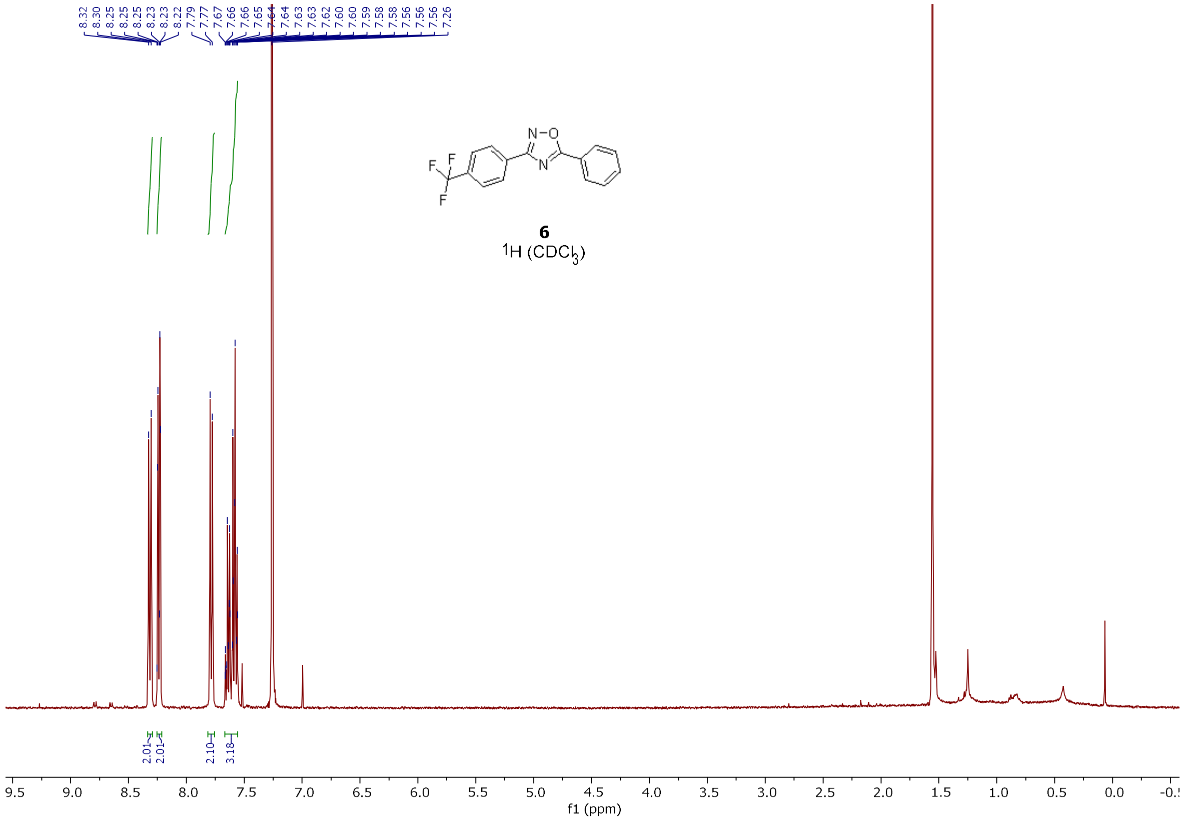

Figure S64. ^{1}H NMR spectrum of **6**.

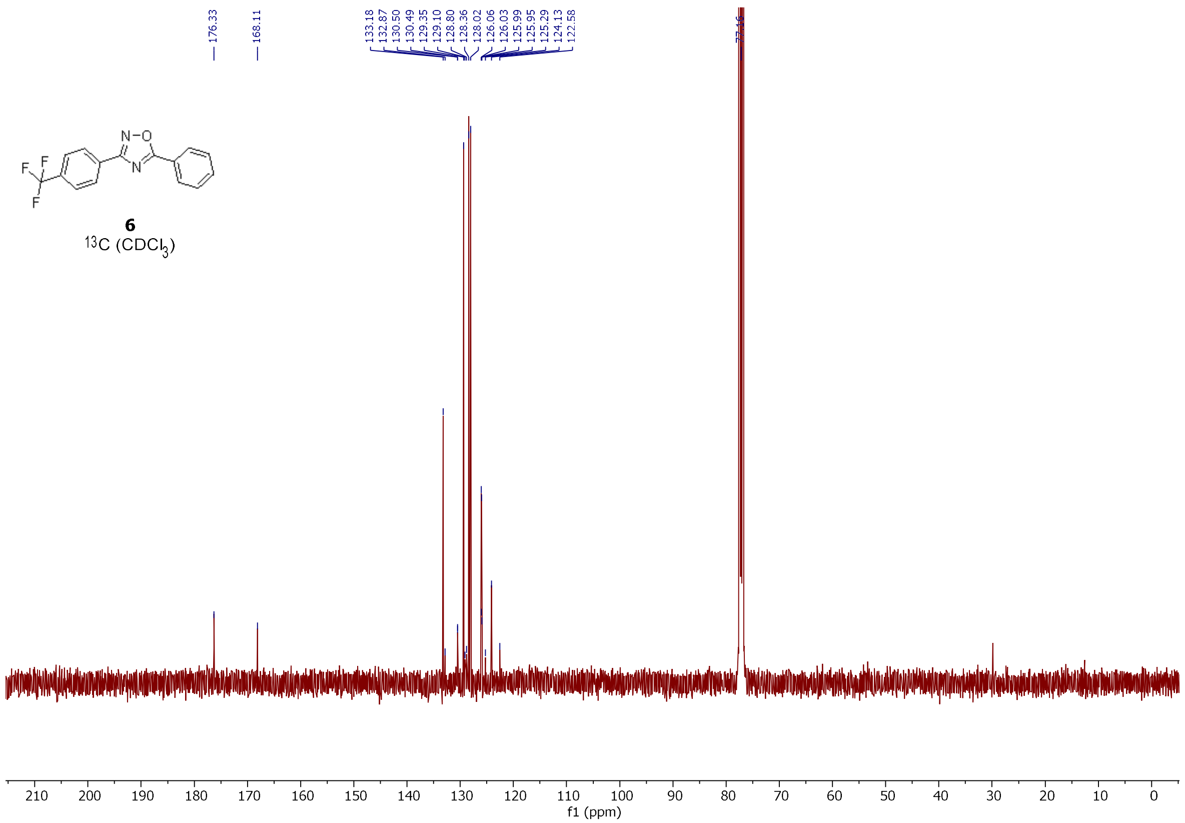

Figure S65. ^{13}C NMR spectrum of **6**.

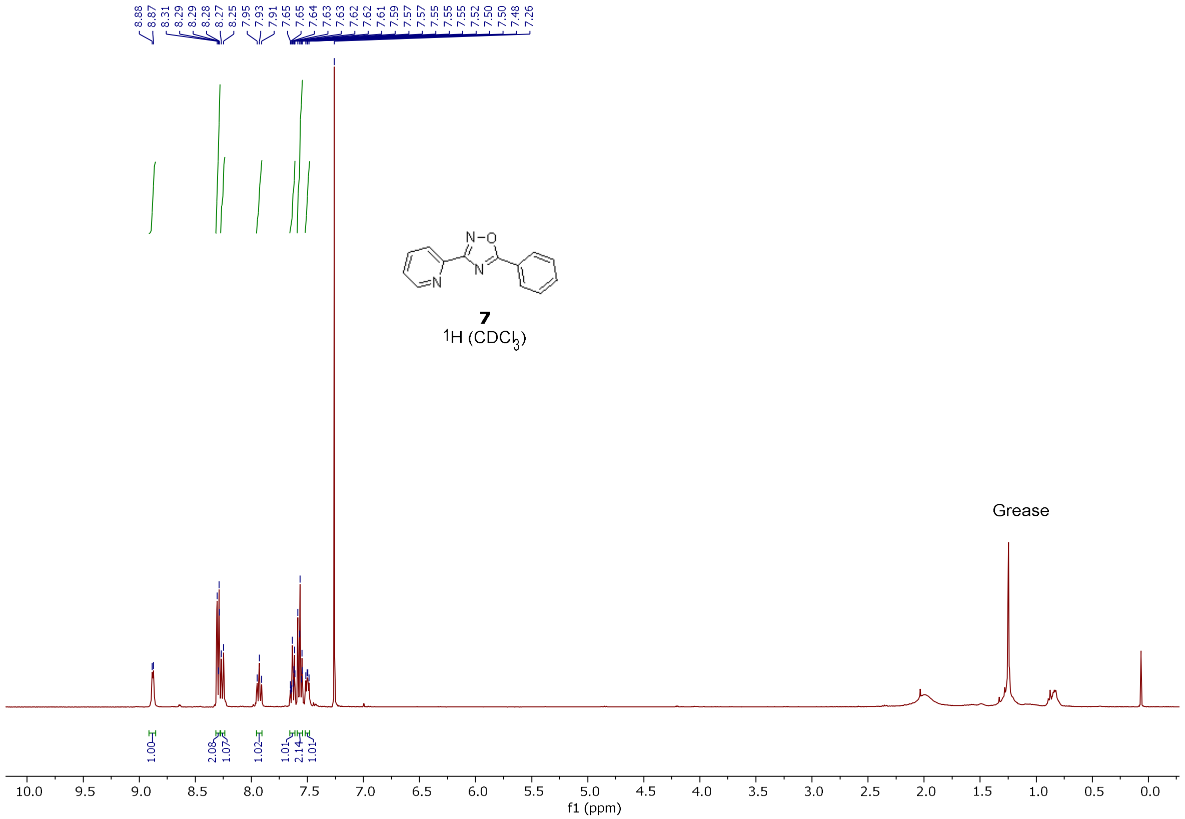

Figure S66. ^{1}H NMR spectrum of **7**.

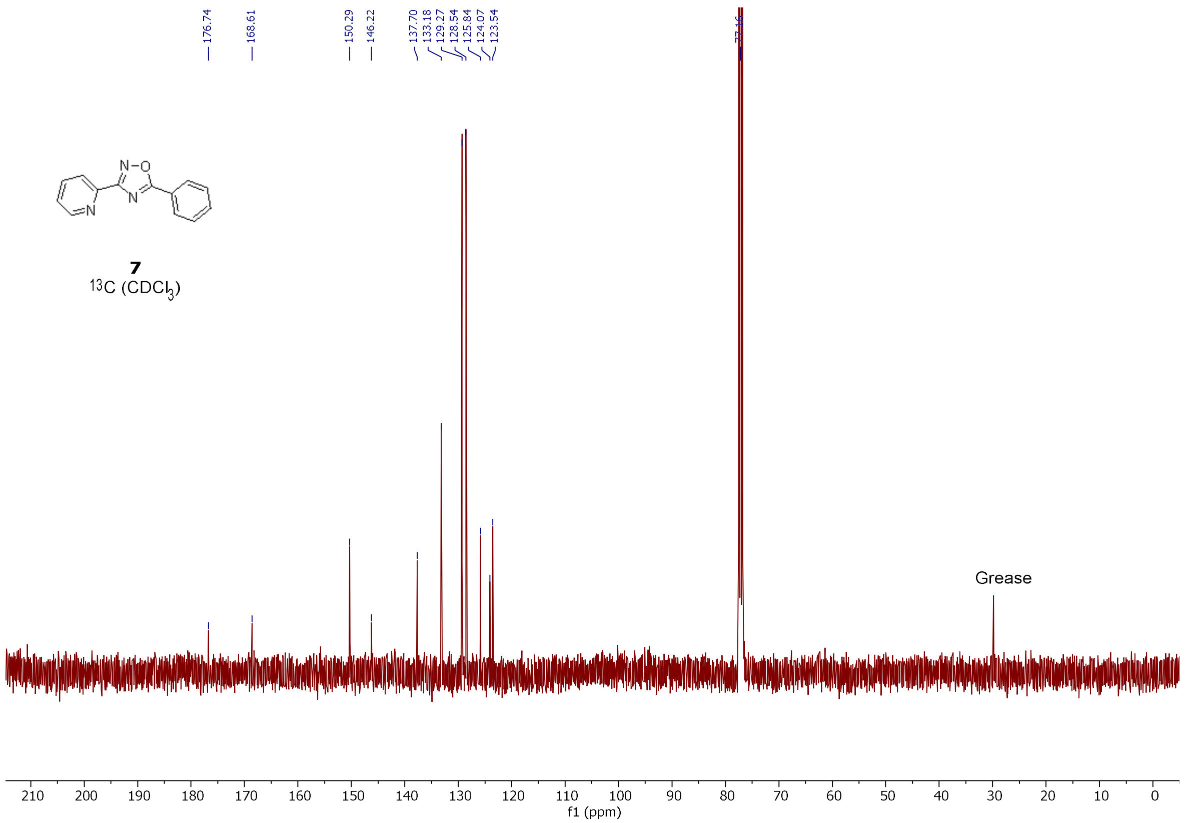

Figure S67. ^{13}C NMR spectrum of **7**.

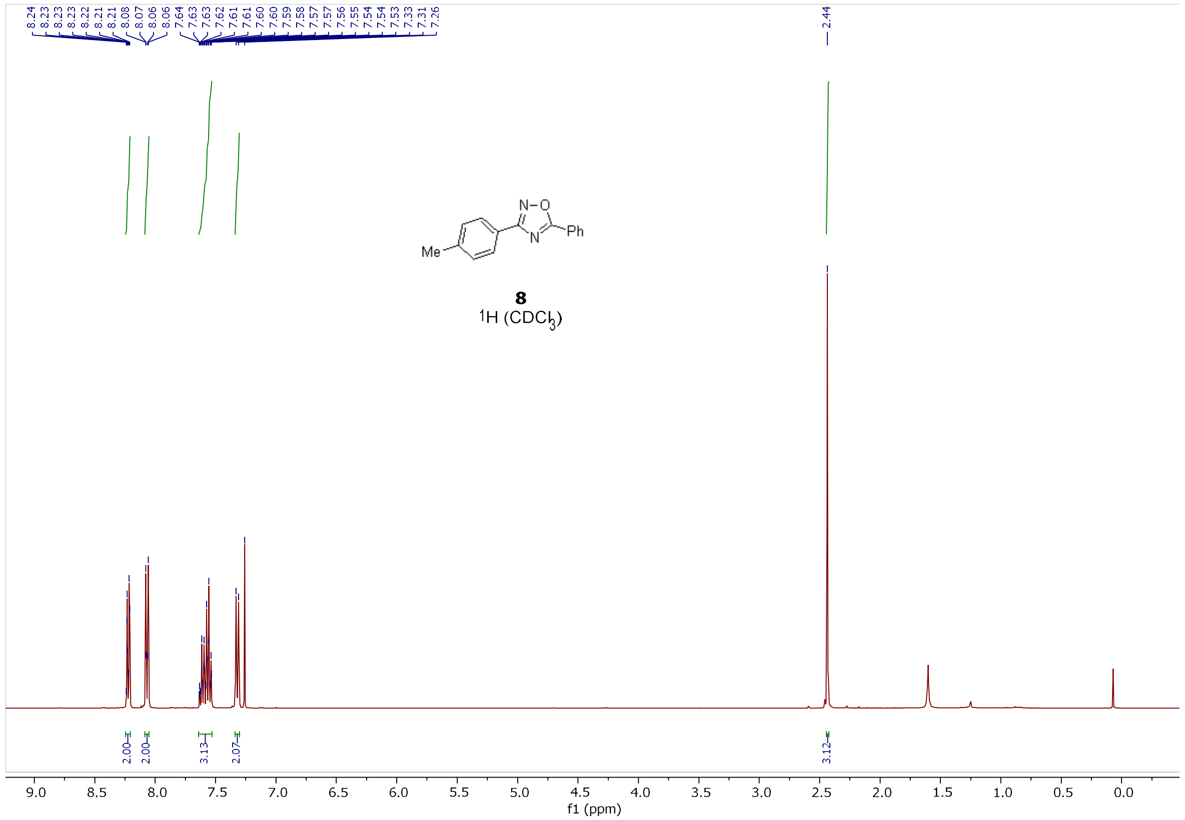

Figure S68. ^{1}H NMR spectrum of **8**.

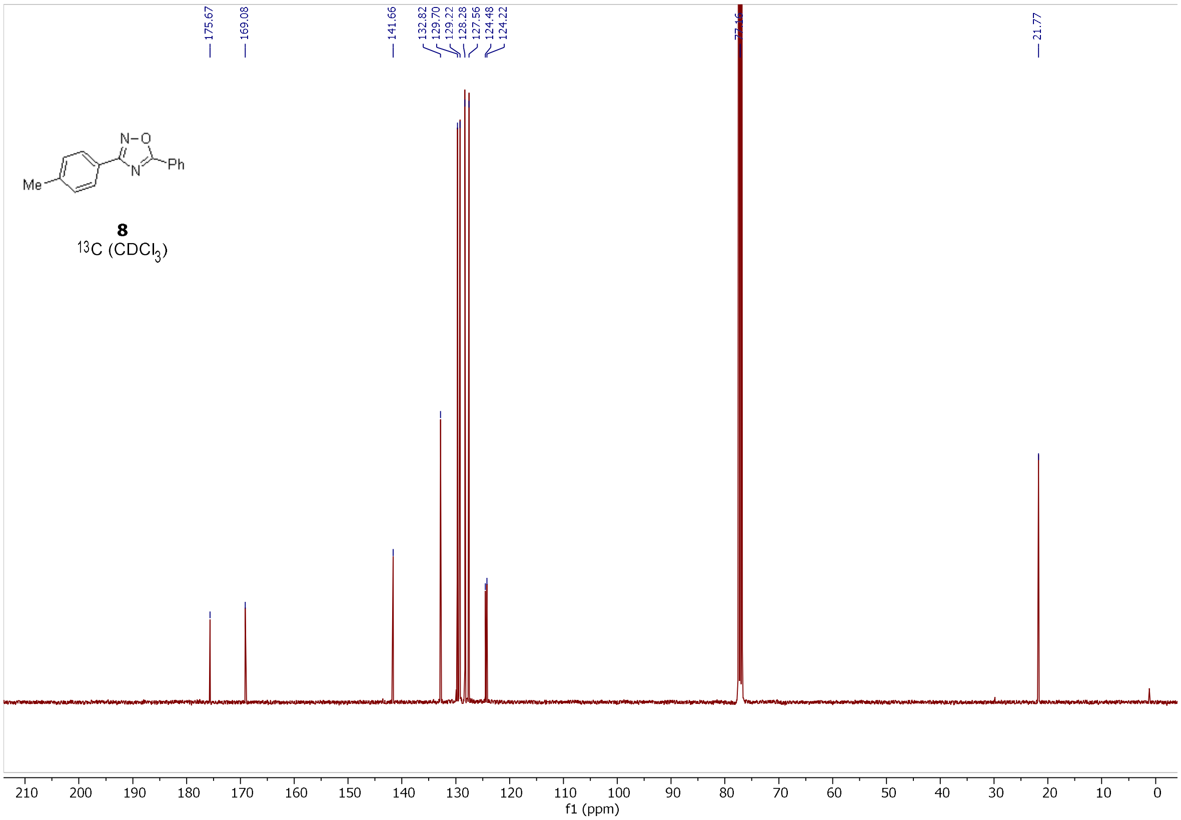

Figure S69. ^{13}C NMR spectrum of **8**.

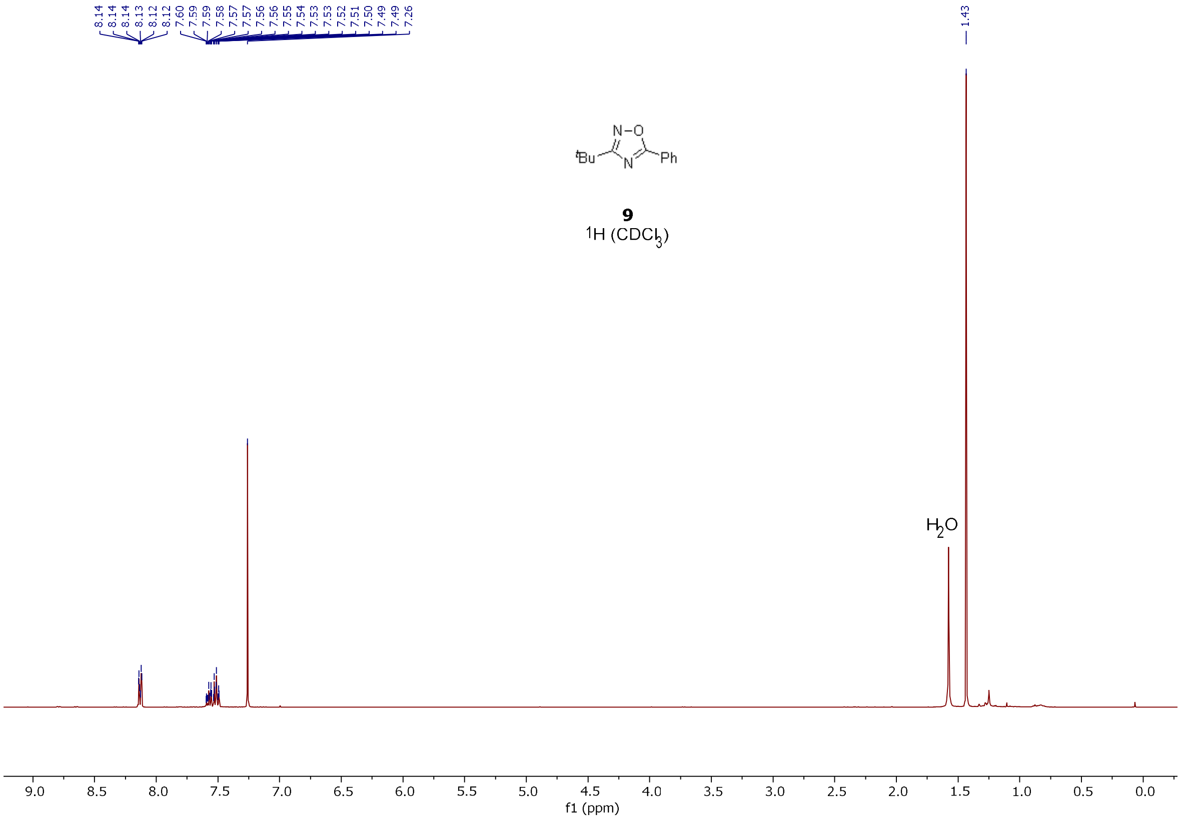

Figure S70. ^{1}H NMR spectrum of **9**.

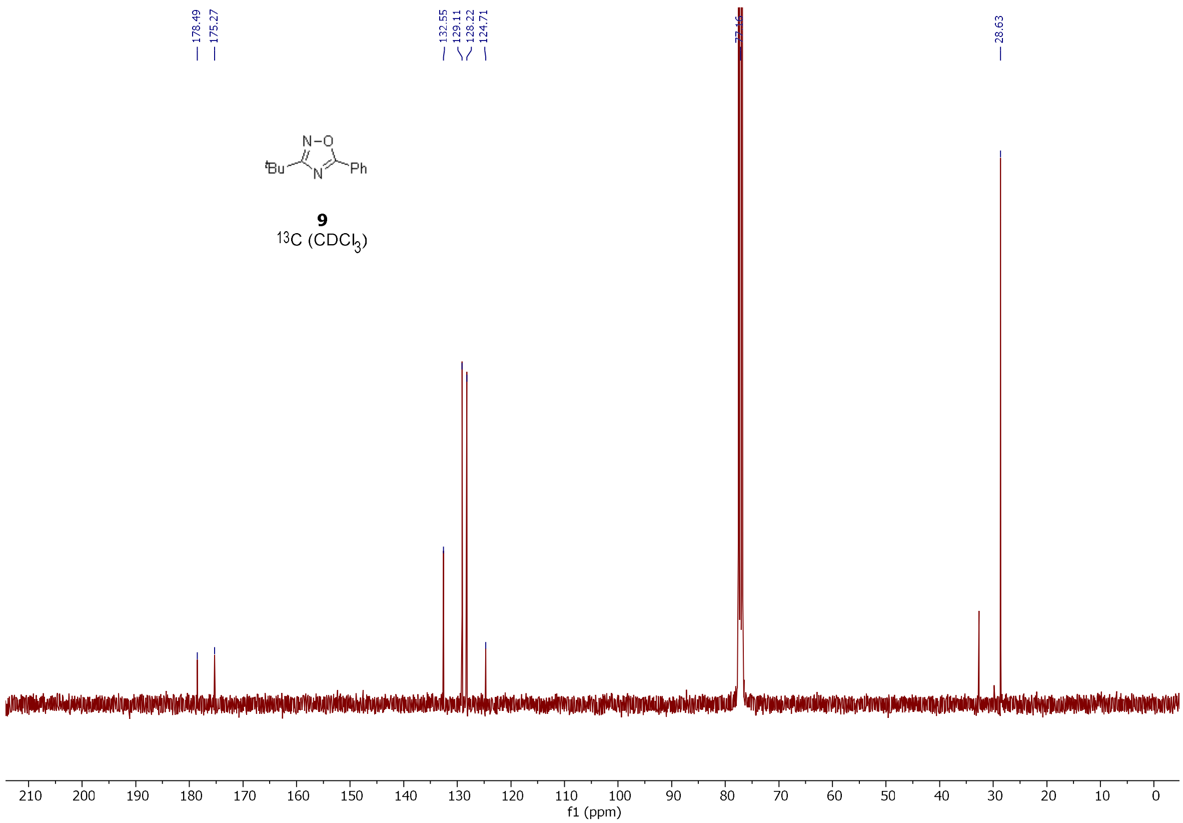

Figure S71. ^{13}C NMR spectrum of **9**.

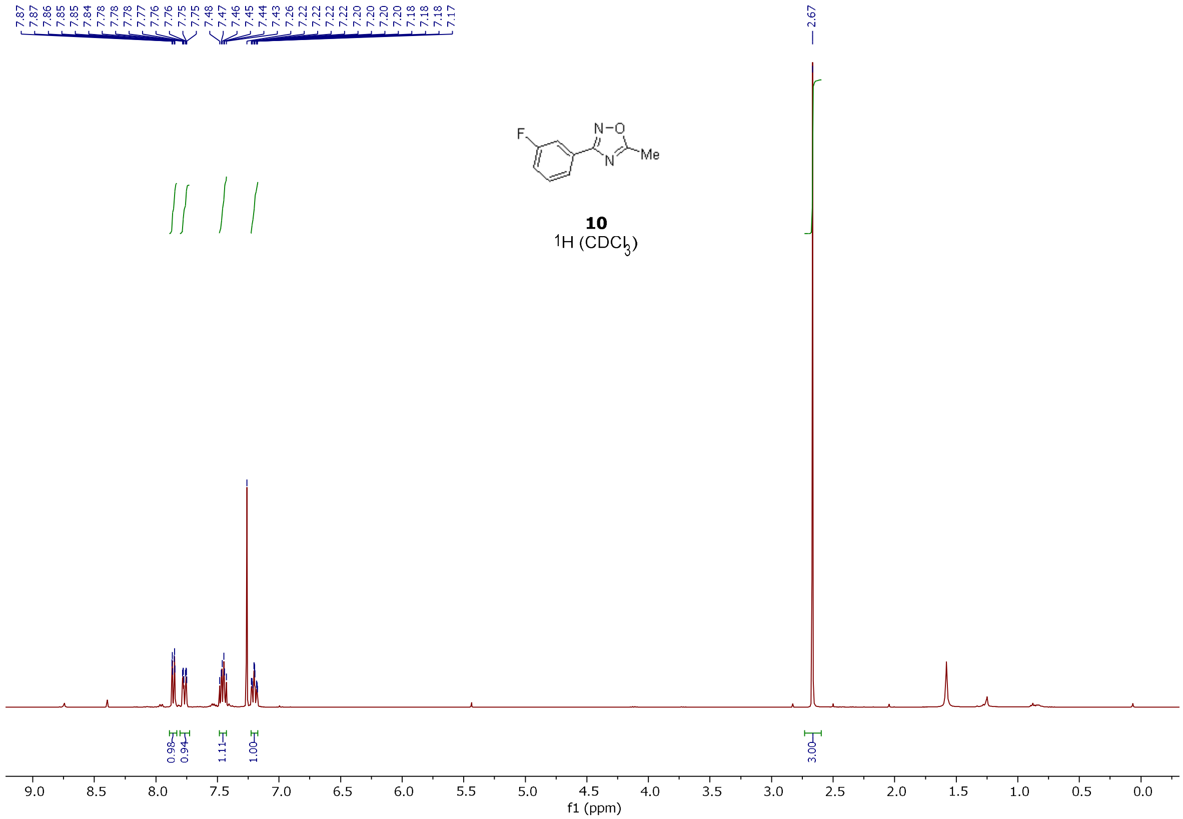

Figure S72. ^{1}H NMR spectrum of **10**.

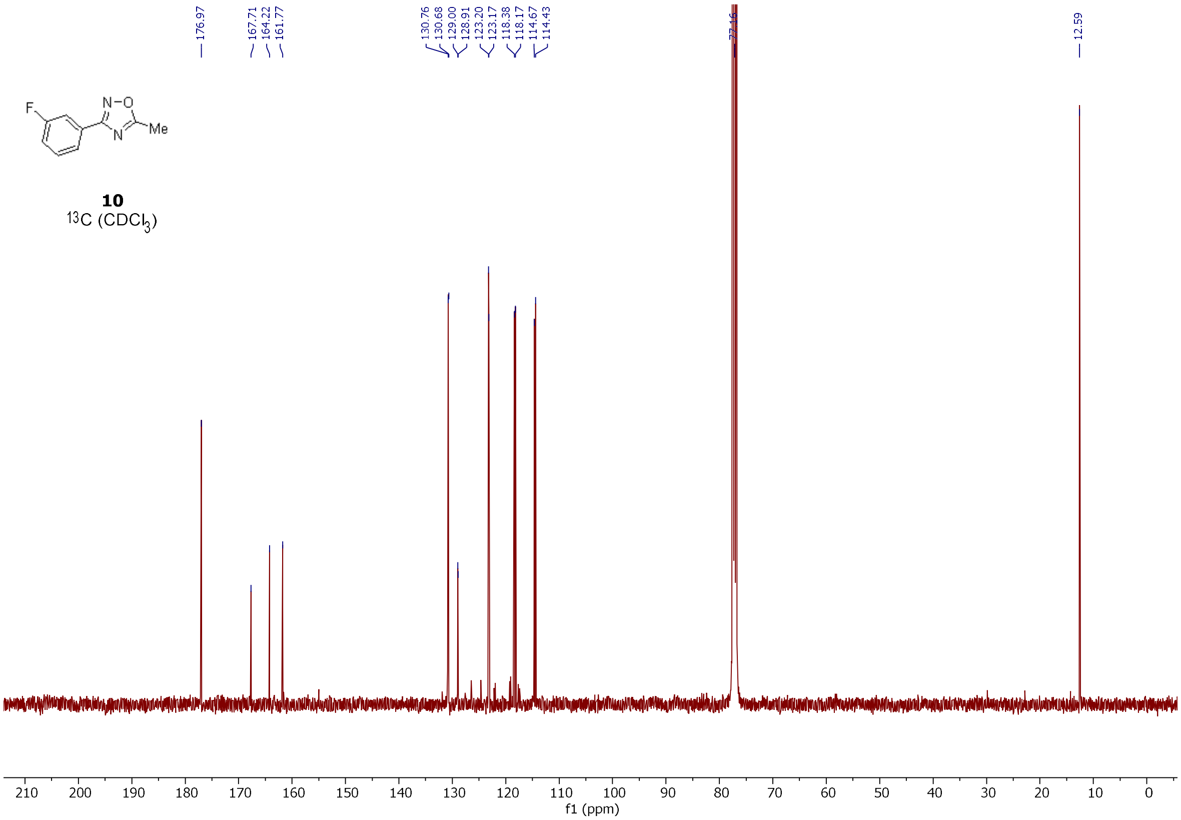

Figure S73. ^{13}C NMR spectrum of **10**.

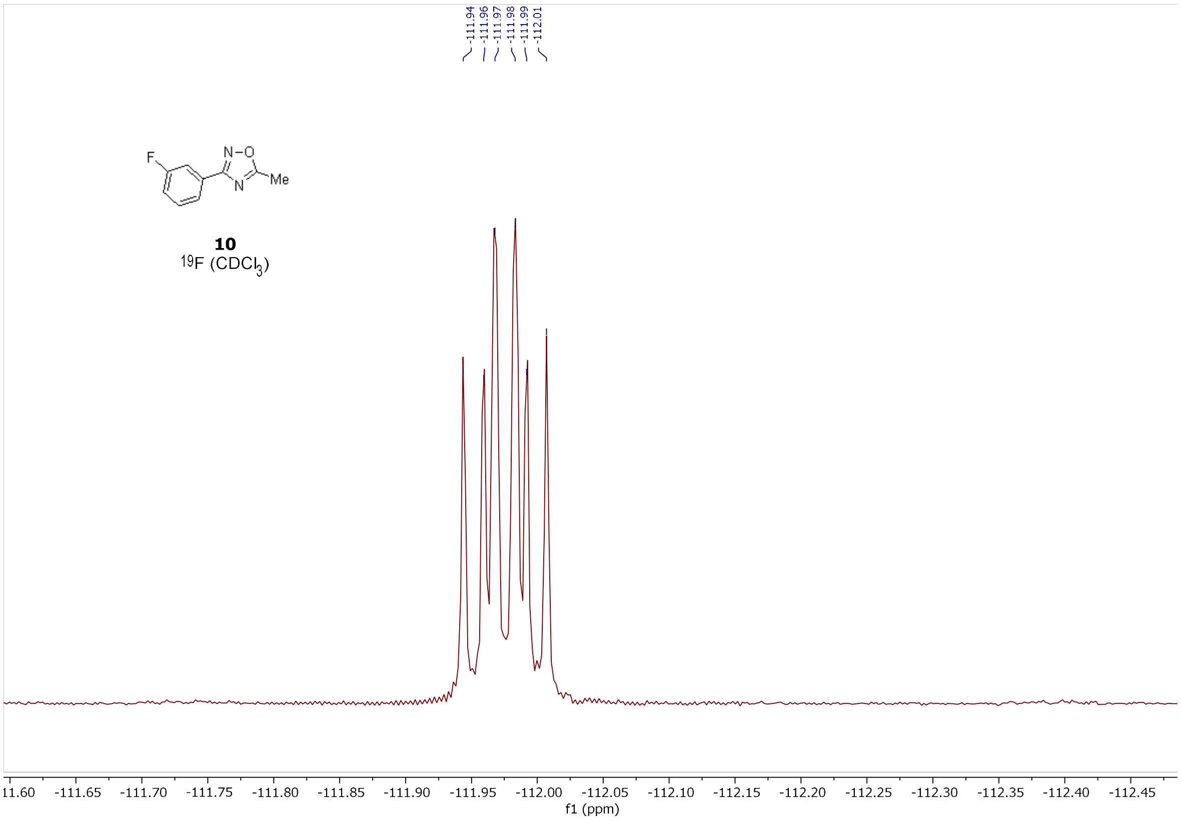

Figure S74. ^{19}F NMR spectrum of **10**.

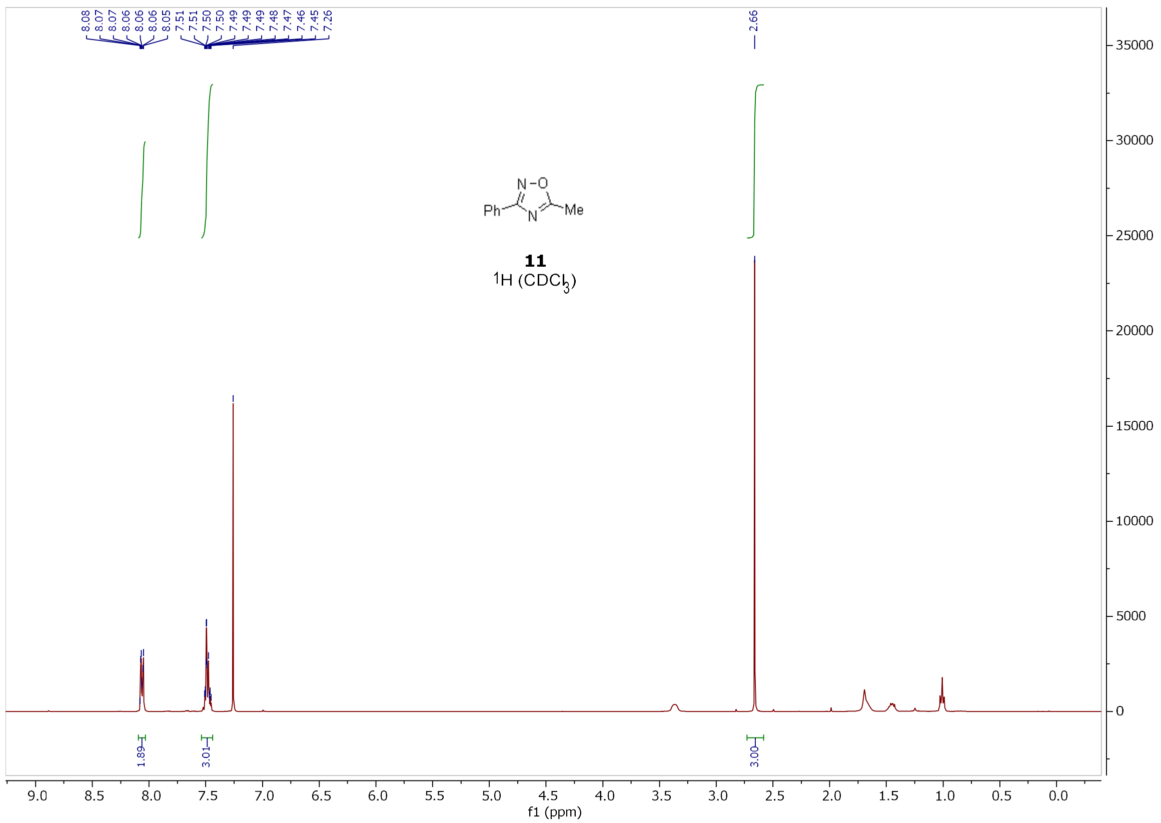

Figure S75. ^{1}H NMR spectrum of **11**.

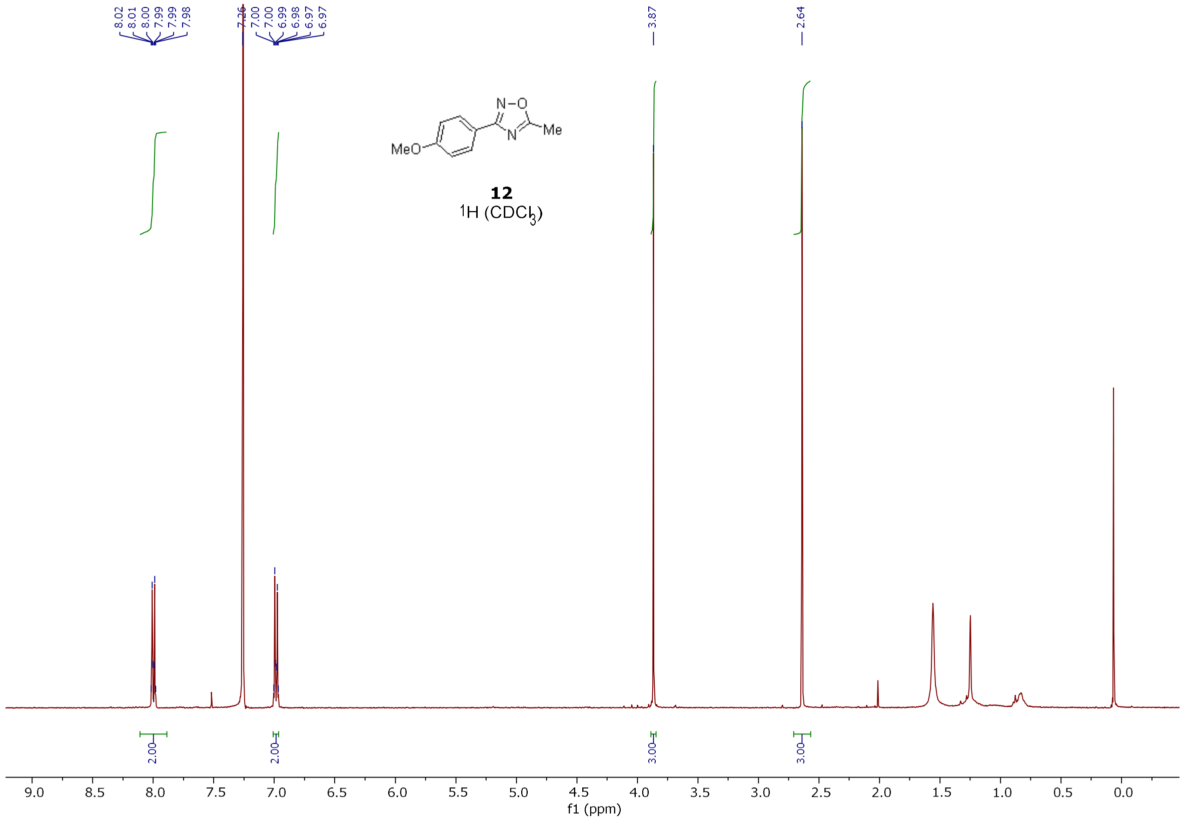

Figure S76. ^{1}H NMR spectrum of **12**.

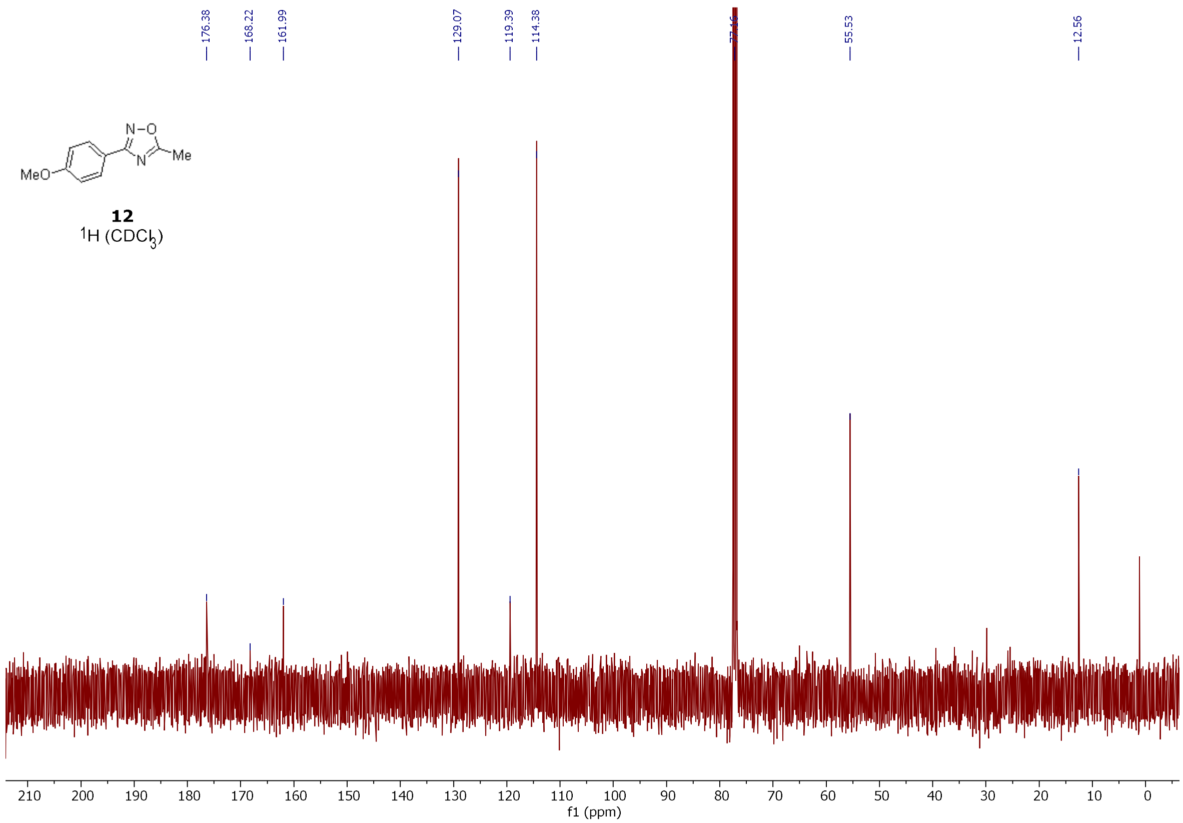

Figure S77. ^{13}C NMR spectrum of **12**.

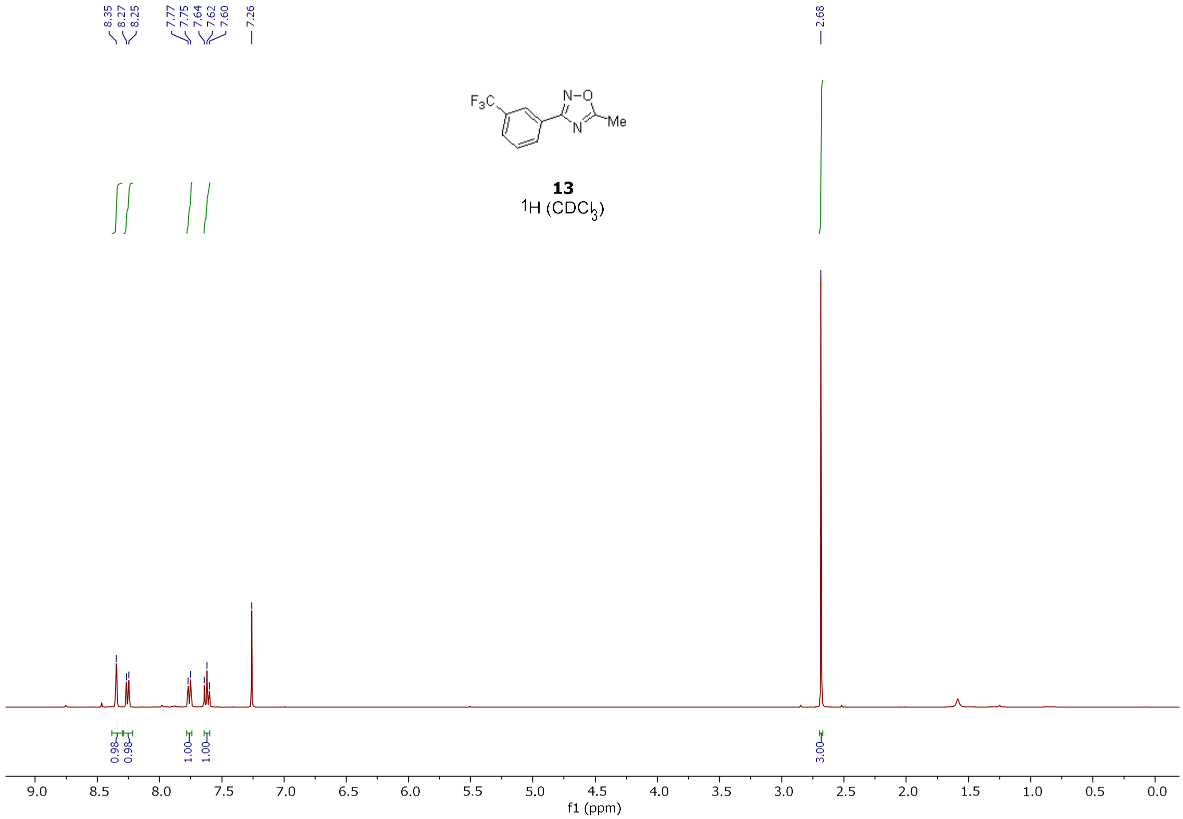

Figure S78. ^{1}H NMR spectrum of **13**.

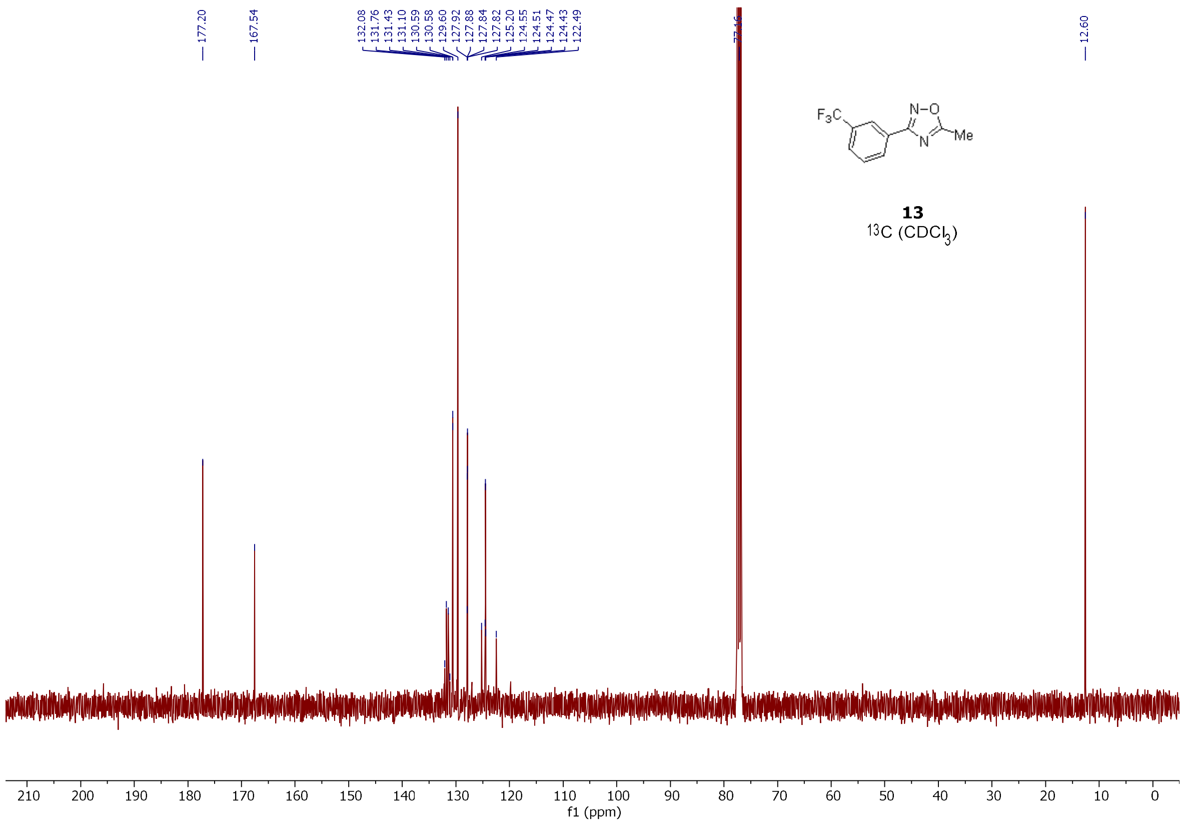

Figure S79. ^{13}C NMR spectrum of **13**.

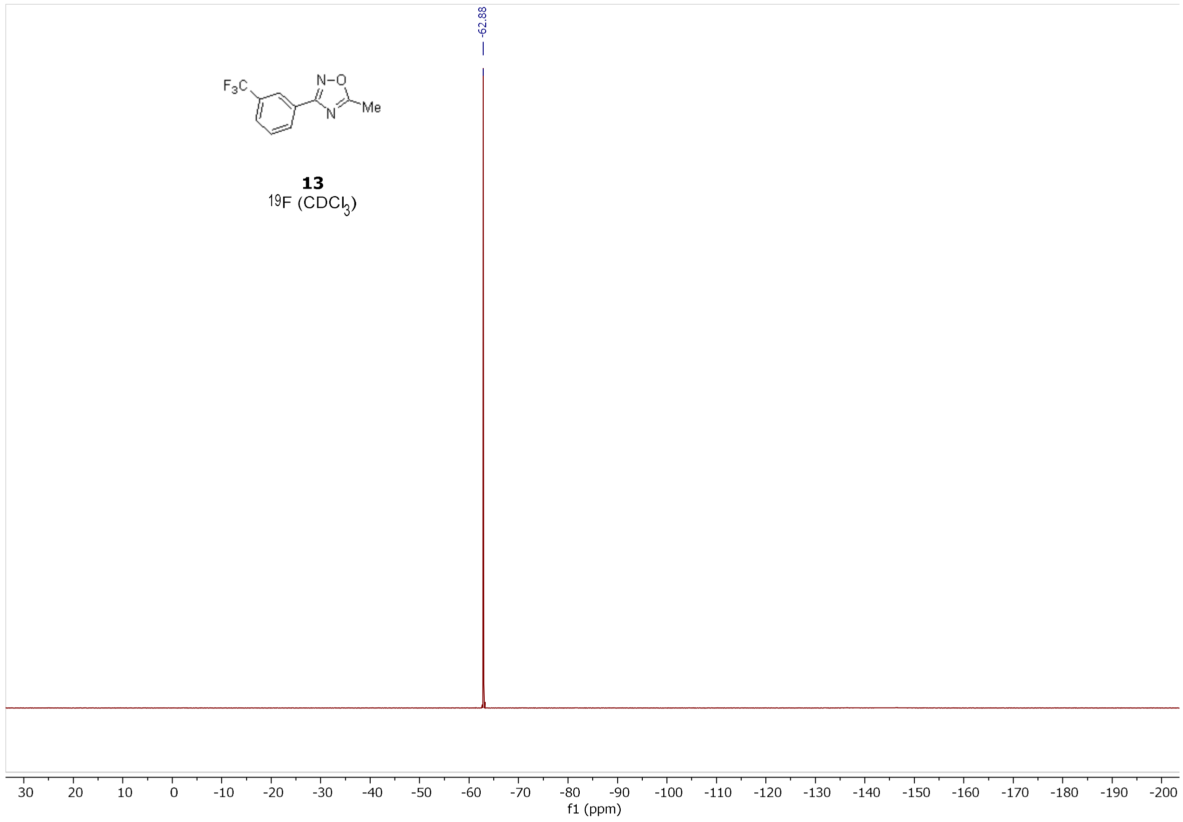

Figure S80. ^{19}F NMR spectrum of **13**.

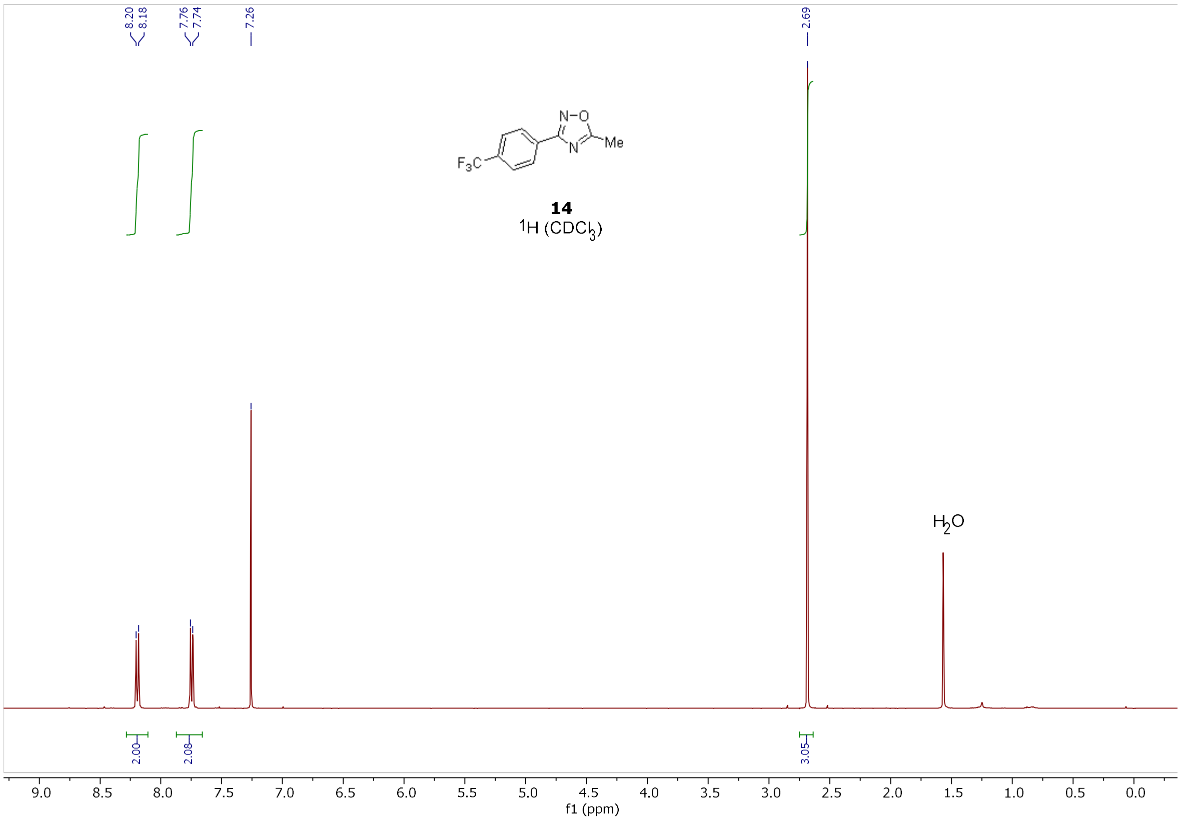

Figure S81. ^{1}H NMR spectrum of **14**.

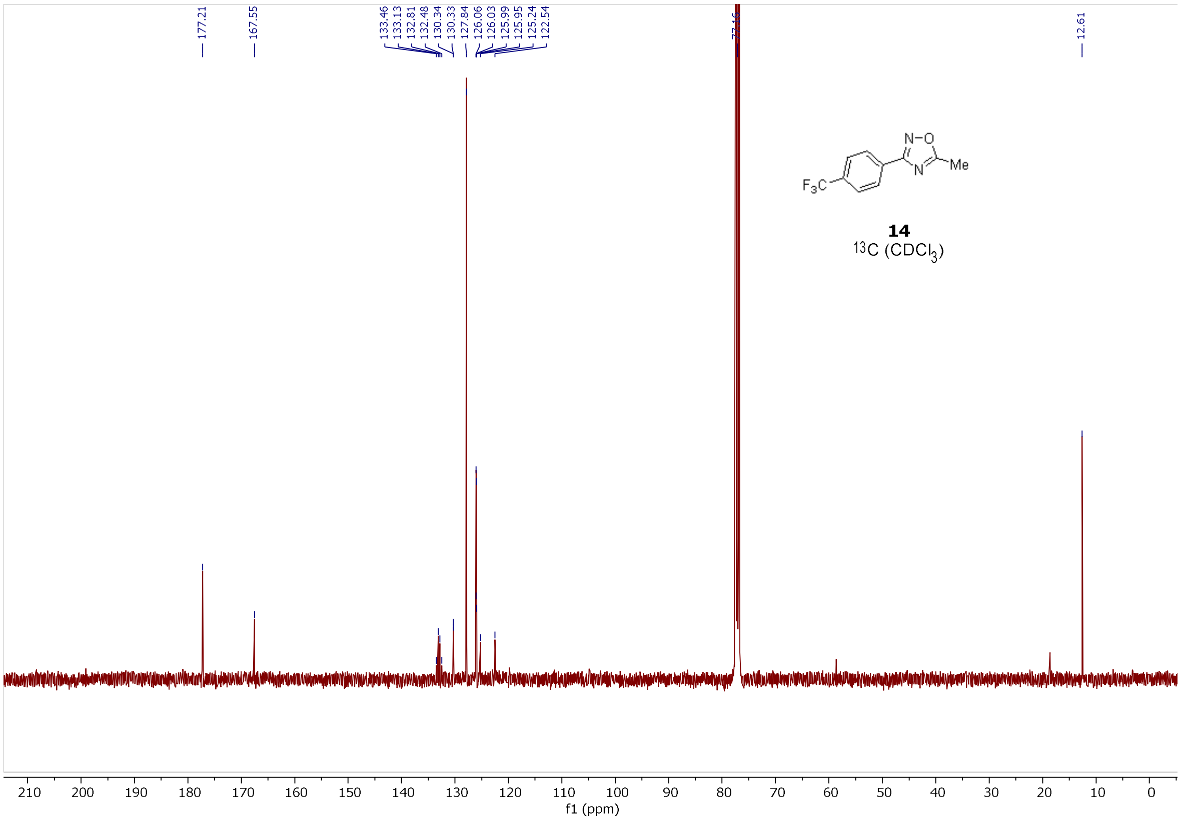

Figure S82. ^{13}C NMR spectrum of **14**.

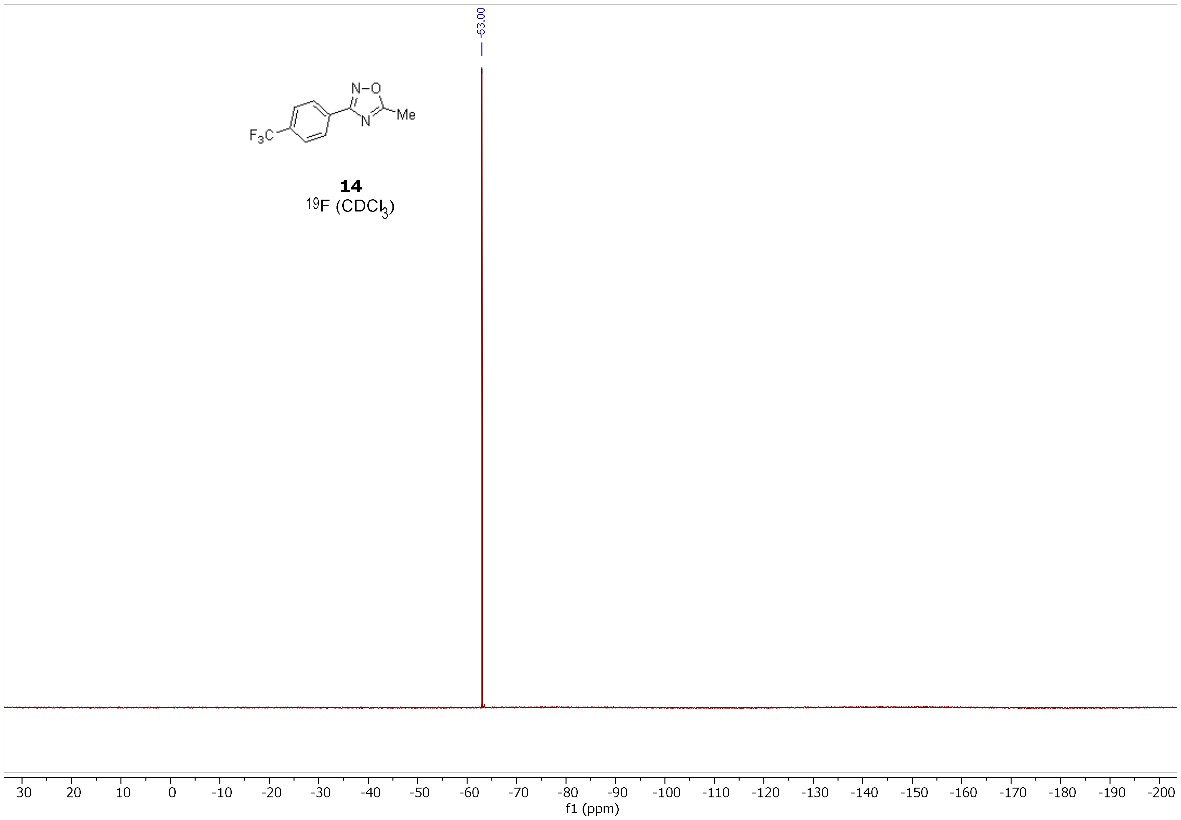

Figure S83. ^{19}F NMR spectrum of **14**.

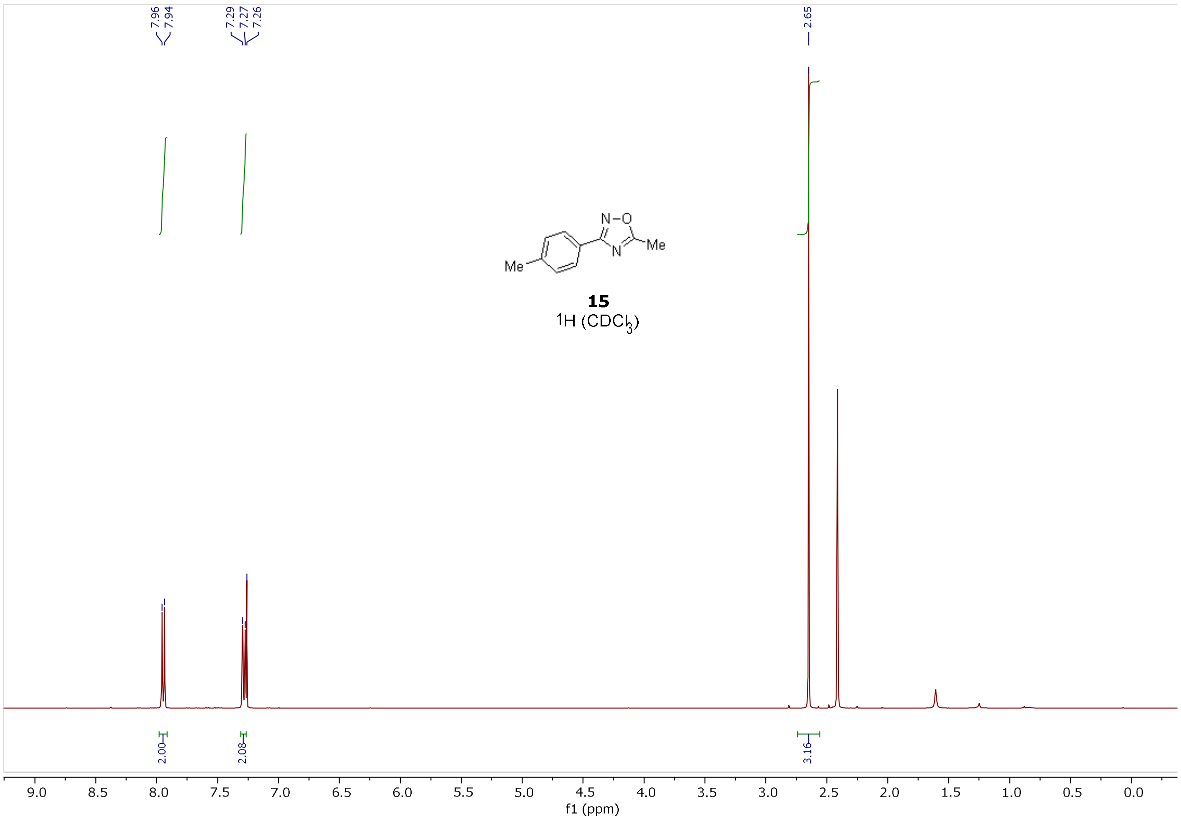

Figure S84. ^{1}H NMR spectrum of **15**.

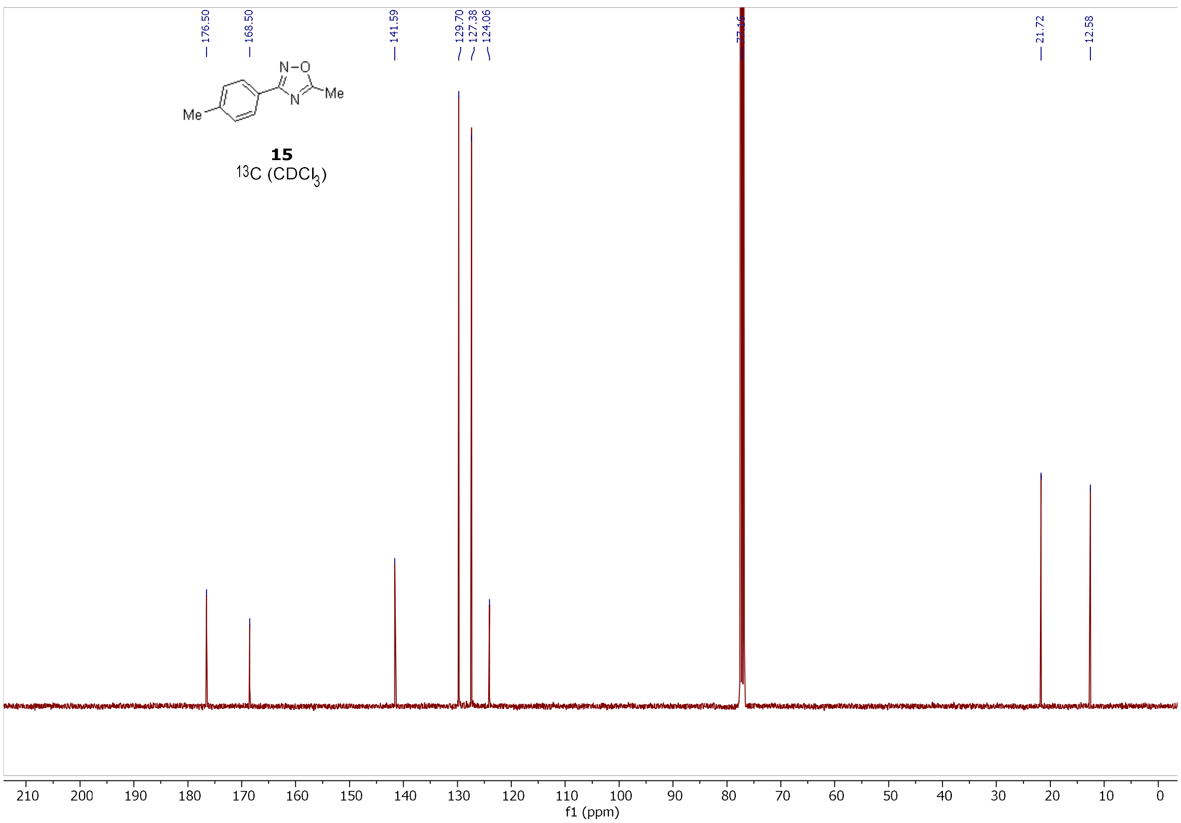

Figure S85. ^{13}C NMR spectrum of **15**.

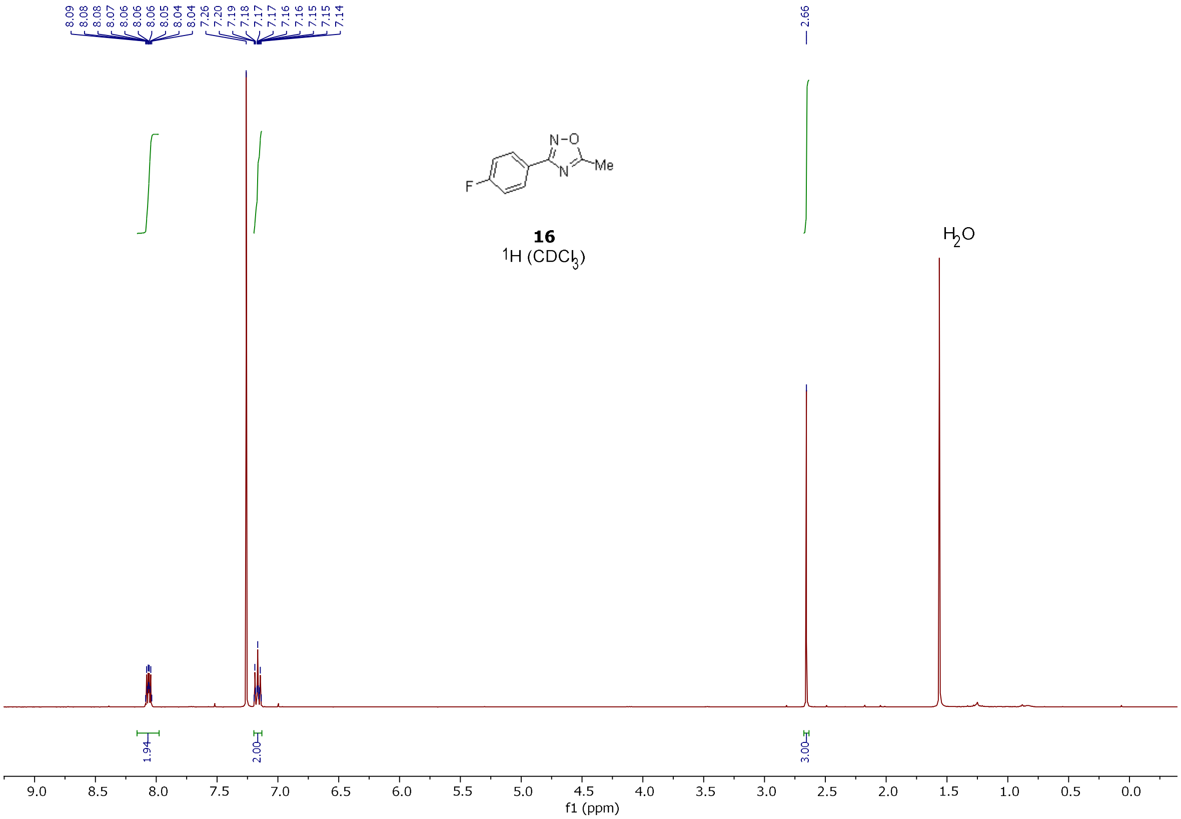

Figure S86. ^{1}H NMR spectrum of **16**.

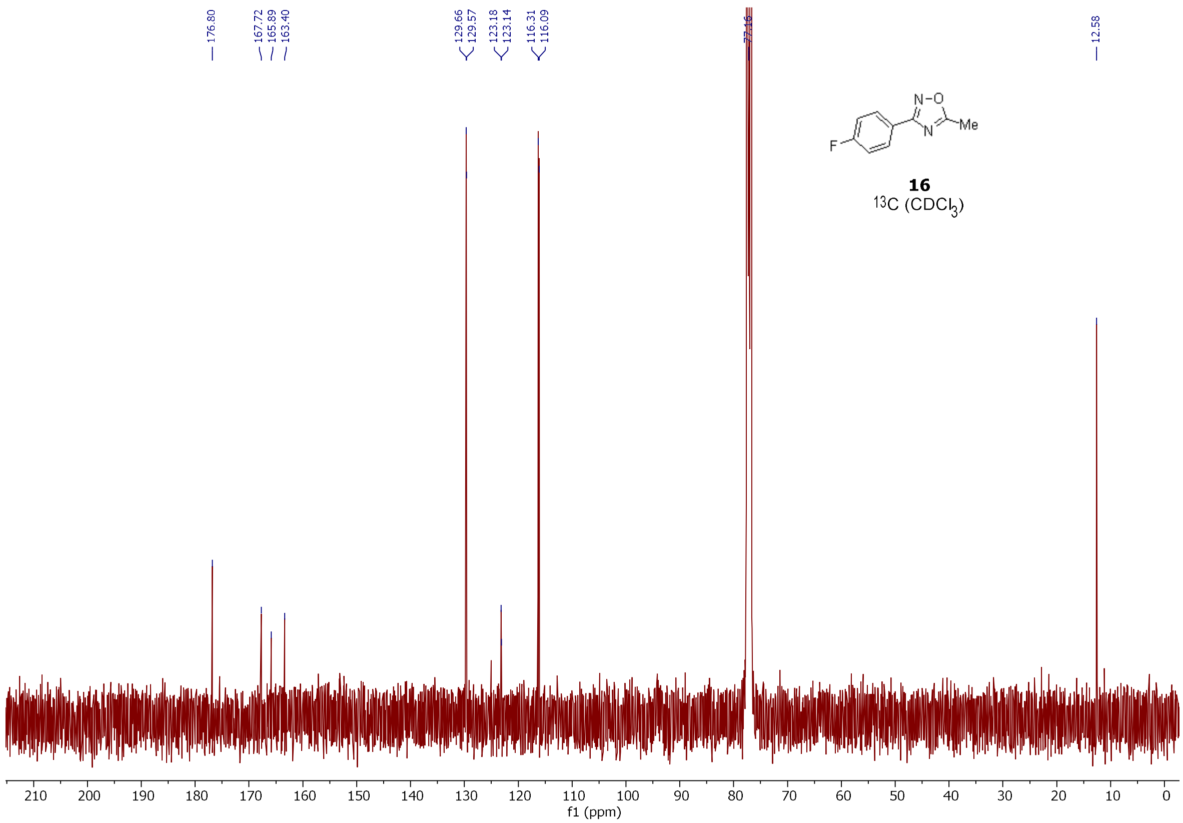

Figure S87. ^{13}C NMR spectrum of **16**.

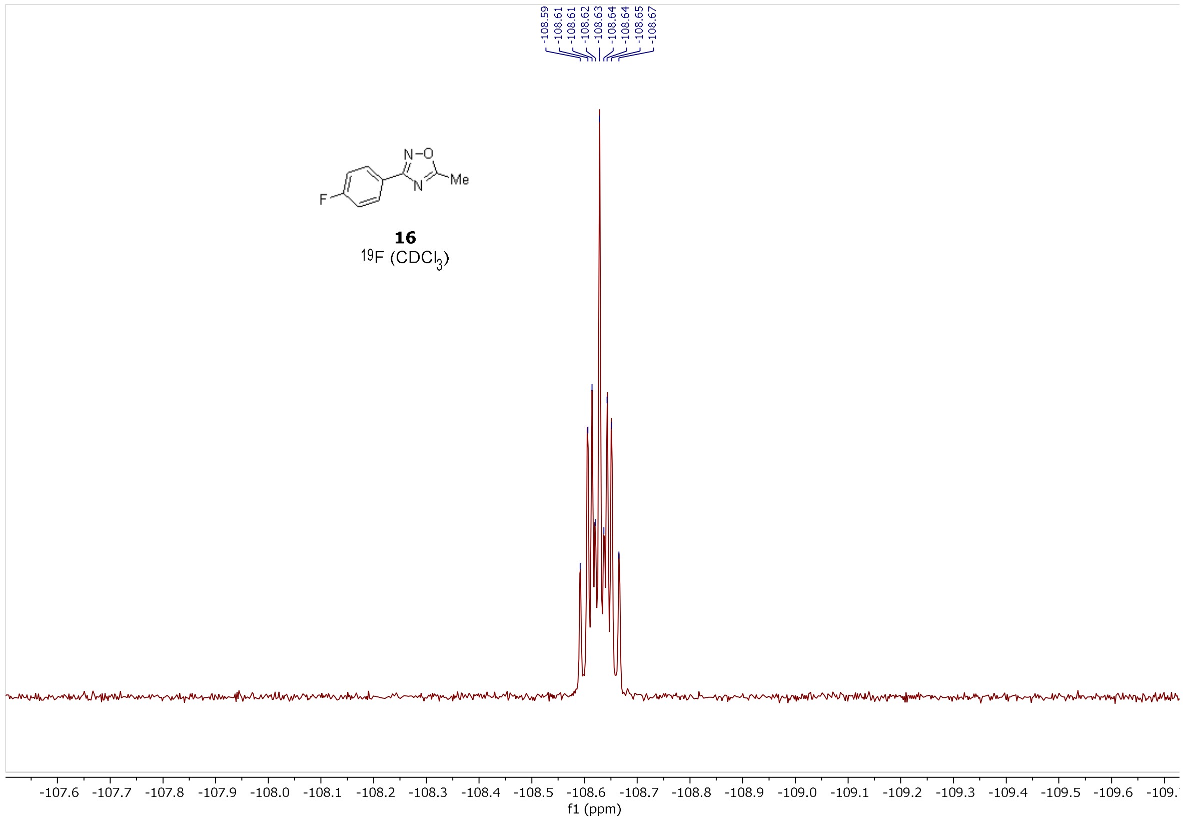

Figure S88. ^{19}F NMR spectrum of **16.**

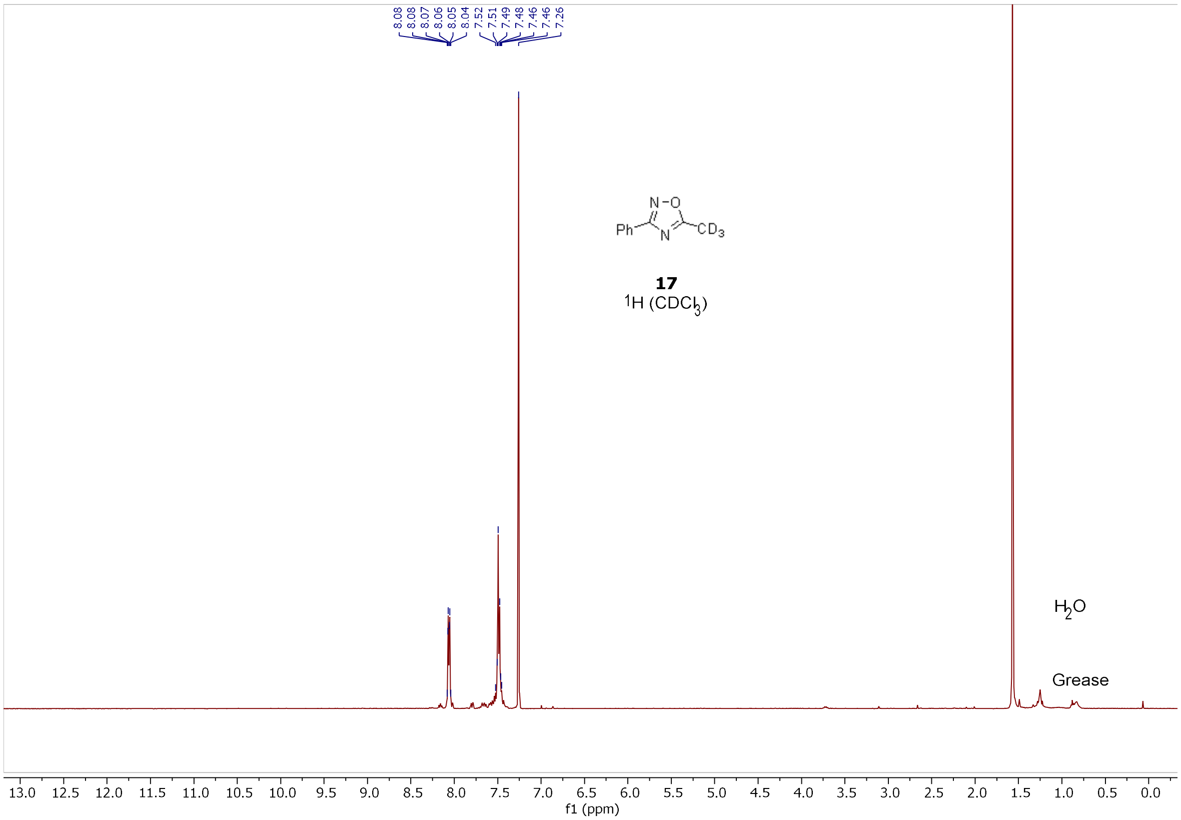

Figure S89. ^{1}H NMR spectrum of **17**.

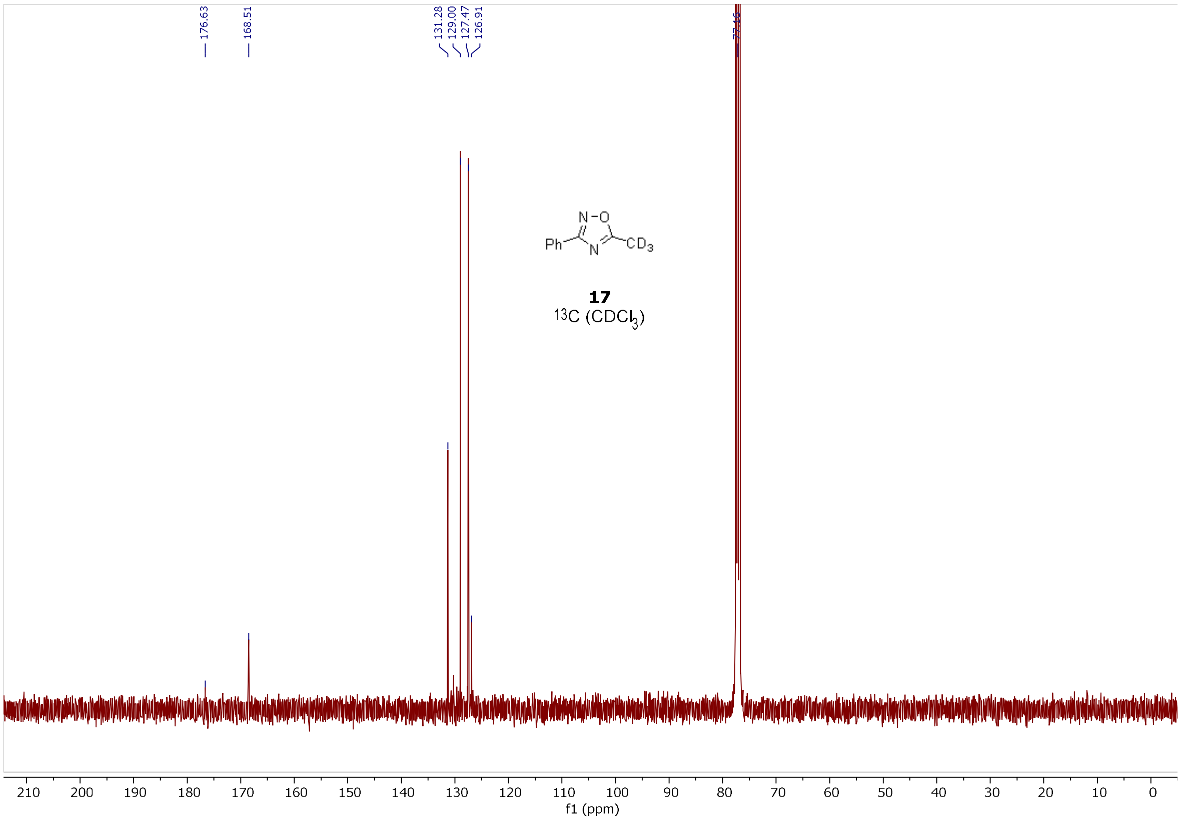

Figure S90. ^{13}C NMR spectrum of **17**.

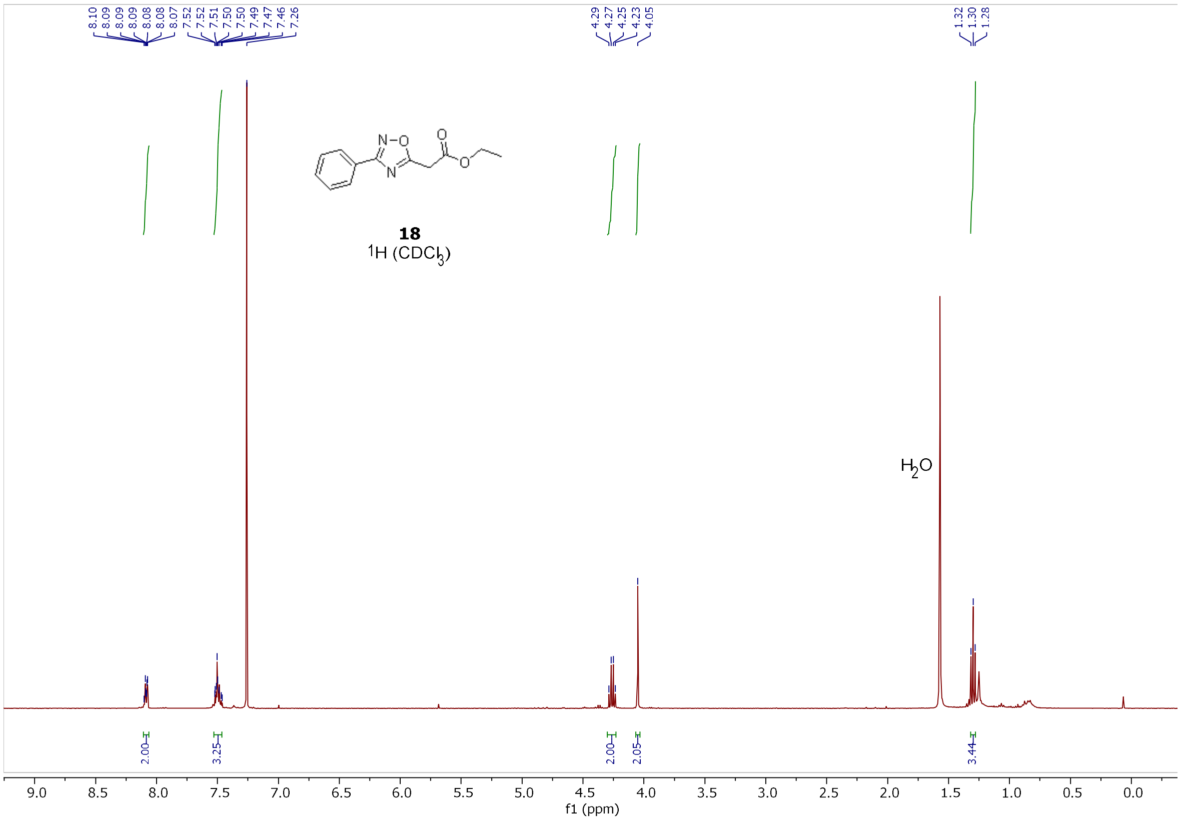

Figure S91. ^{1}H NMR spectrum of **18**.

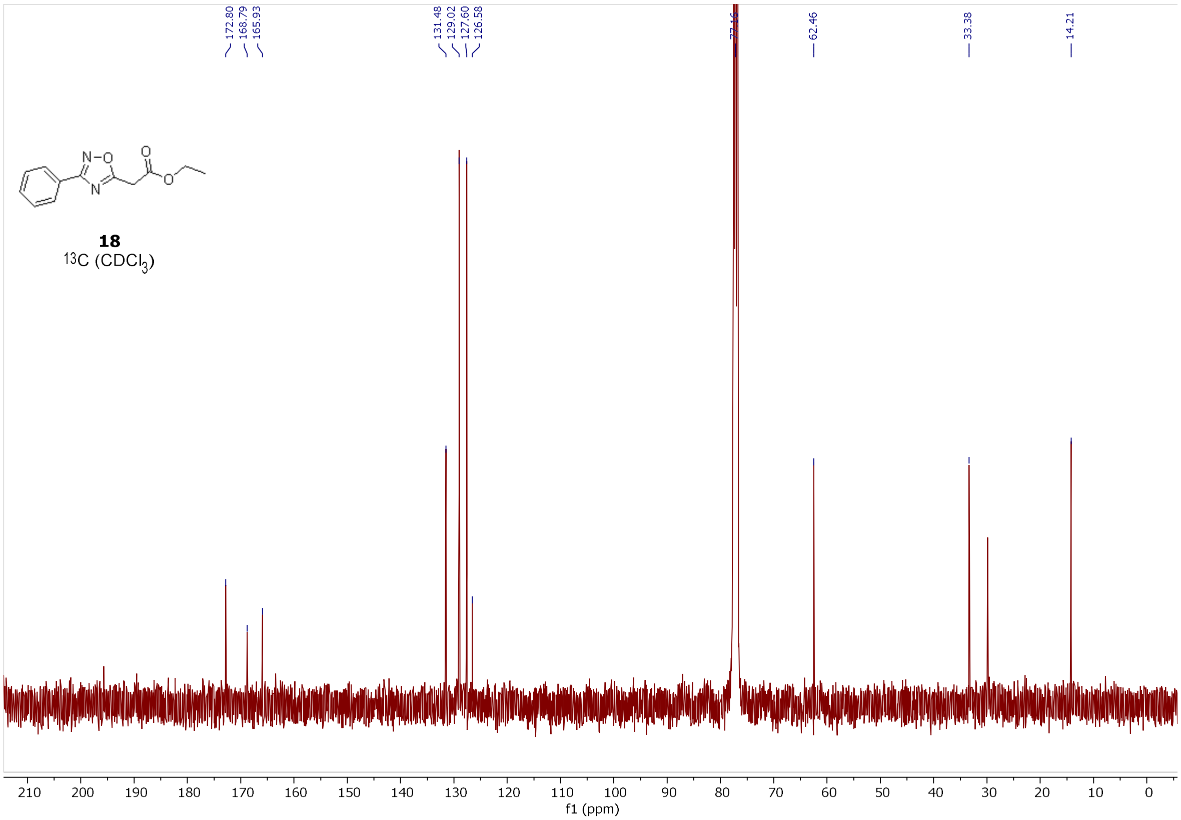

Figure S92. ^{13}C NMR spectrum of **18**.

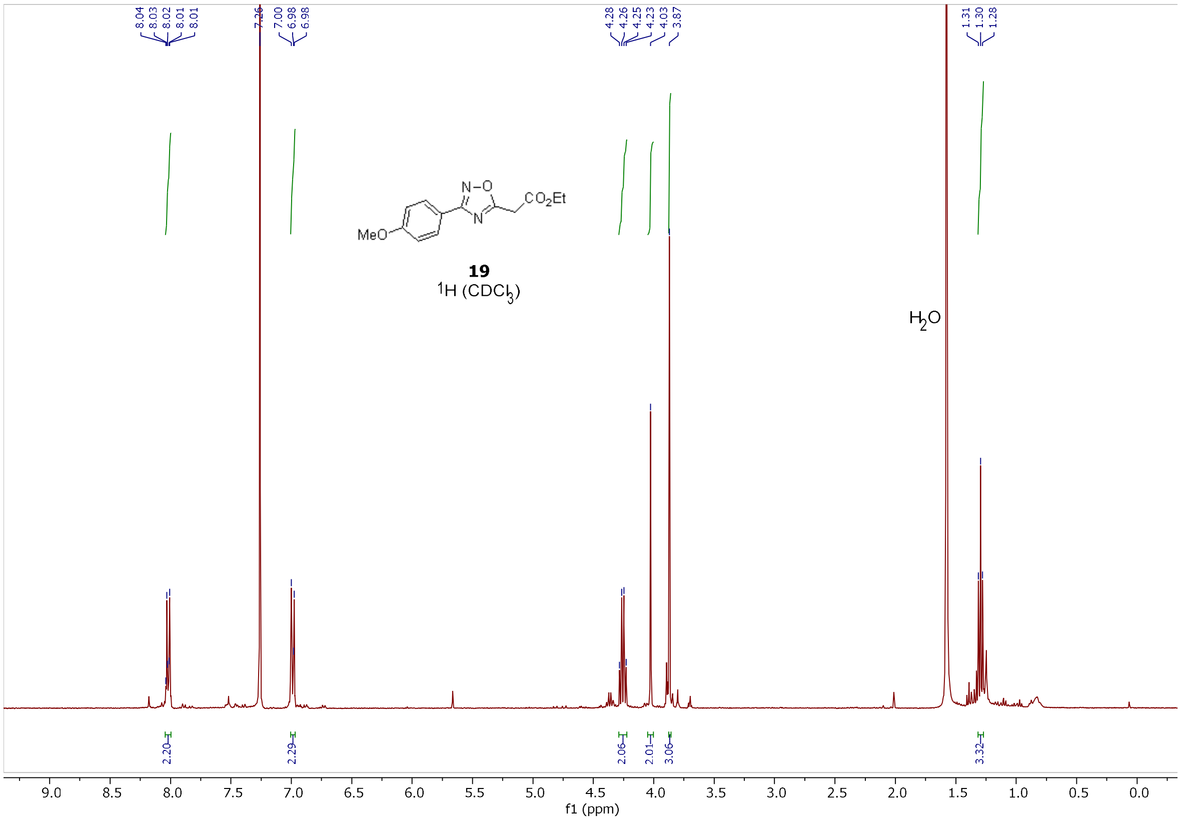

Figure S93. ^{1}H NMR spectrum of **19**.

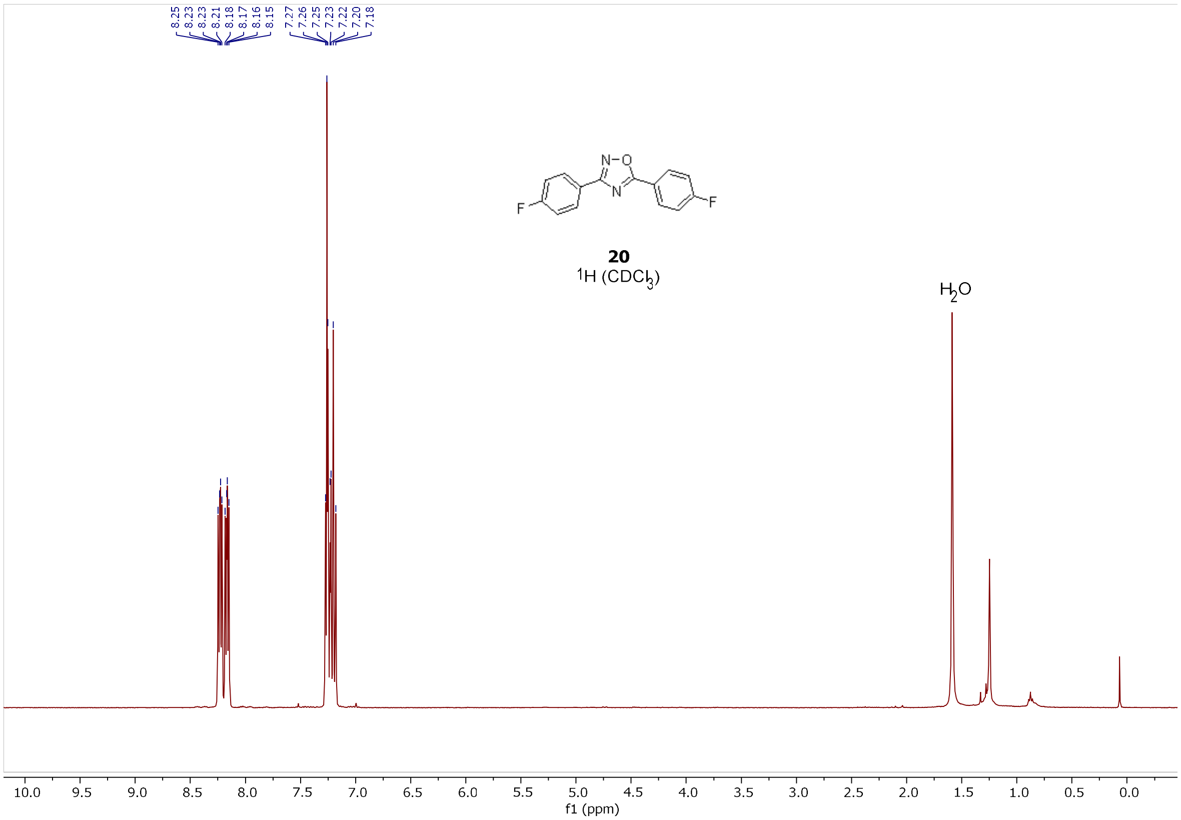

Figure S94. ^{1}H NMR spectrum of **20**.

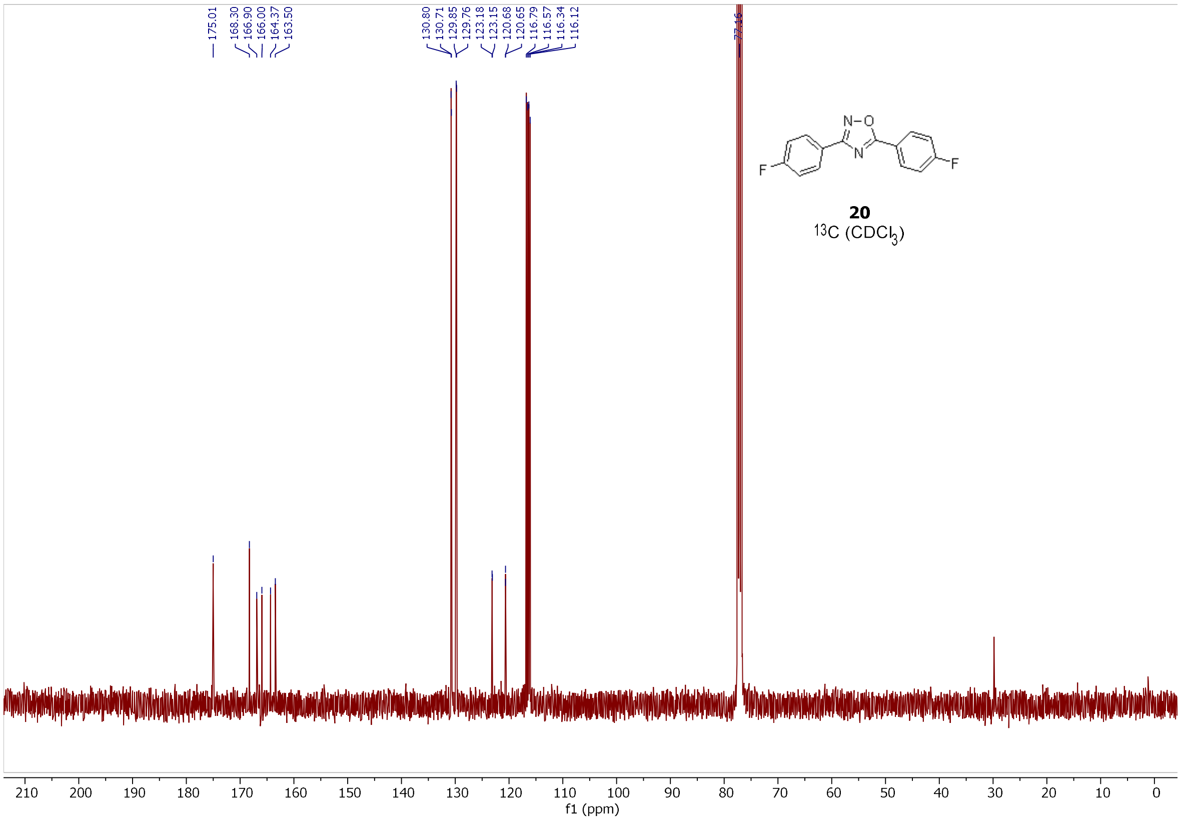

Figure S95. ^{13}C NMR spectrum of **20**.

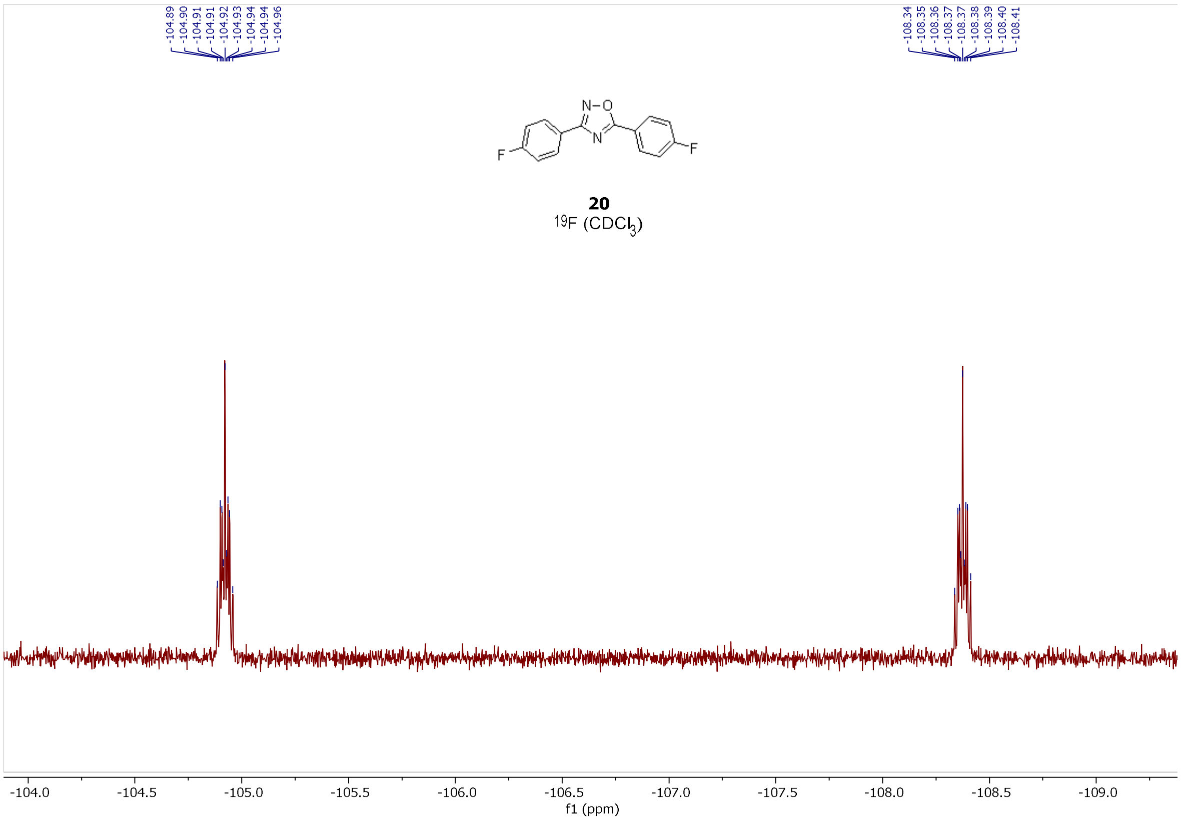

Figure S96. ^{19}F NMR spectrum of **20**.

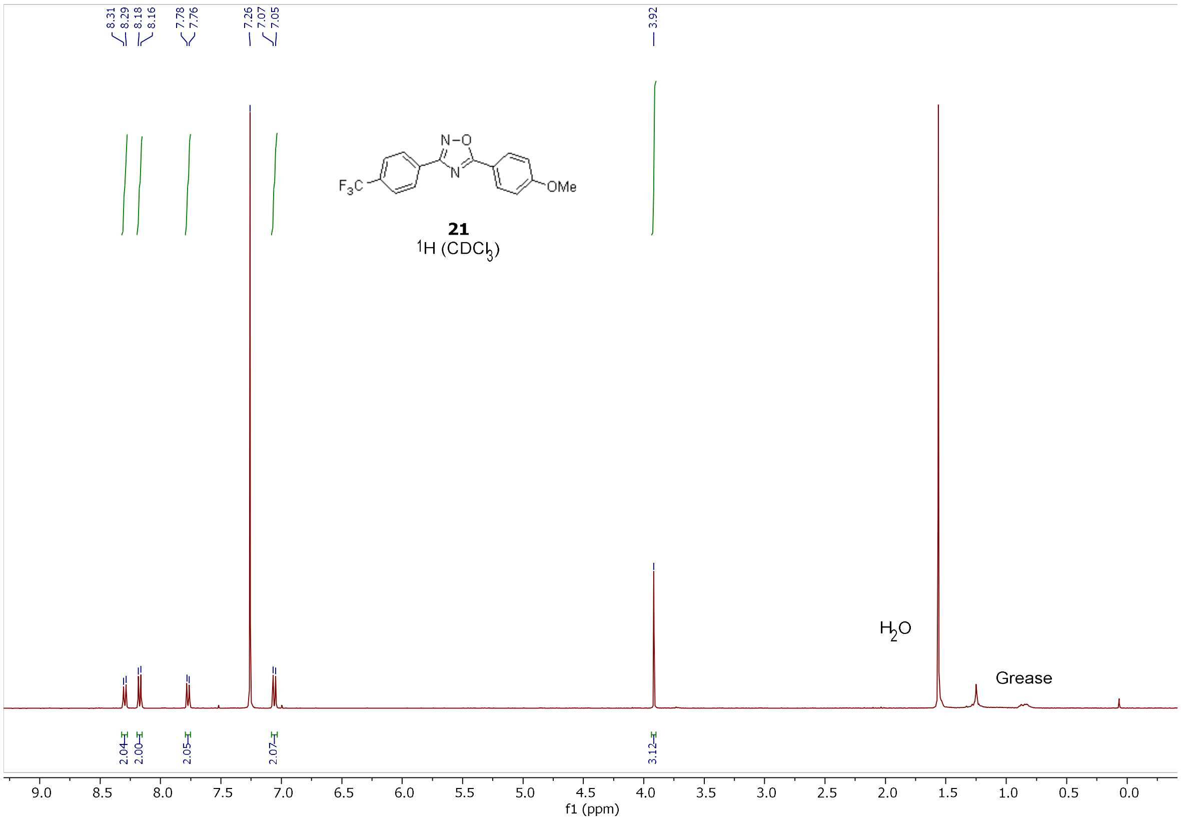

Figure S97. ^{1}H NMR spectrum of **21**.

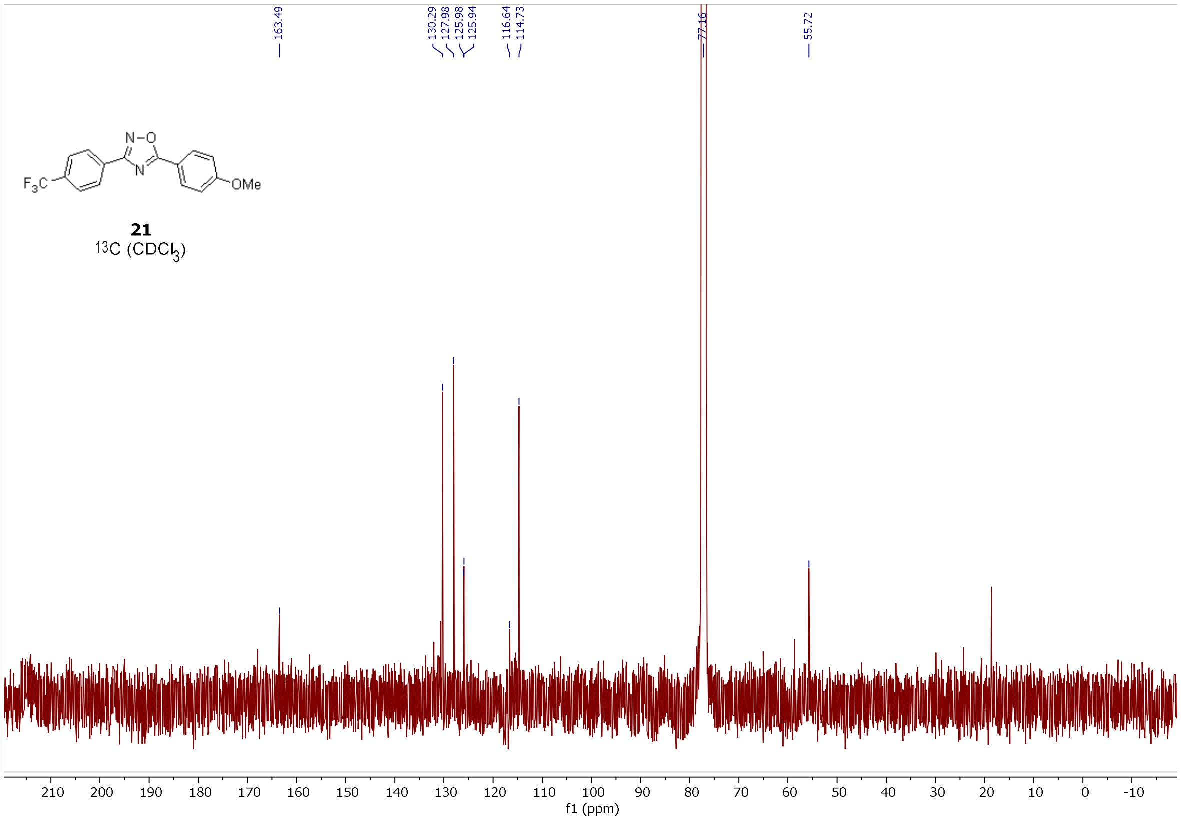

Figure S98. ^{13}C NMR spectrum of **21**.

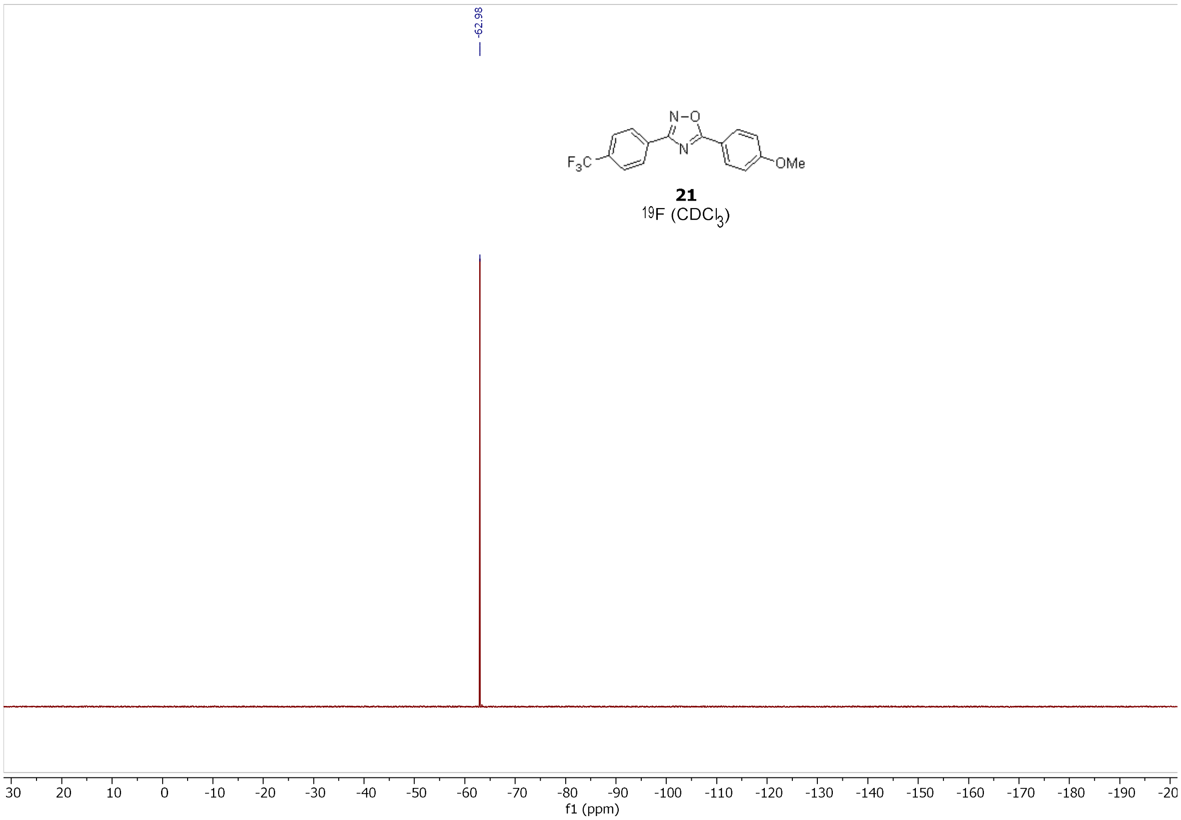

Figure S99. ^{19}F NMR spectrum of **21**.

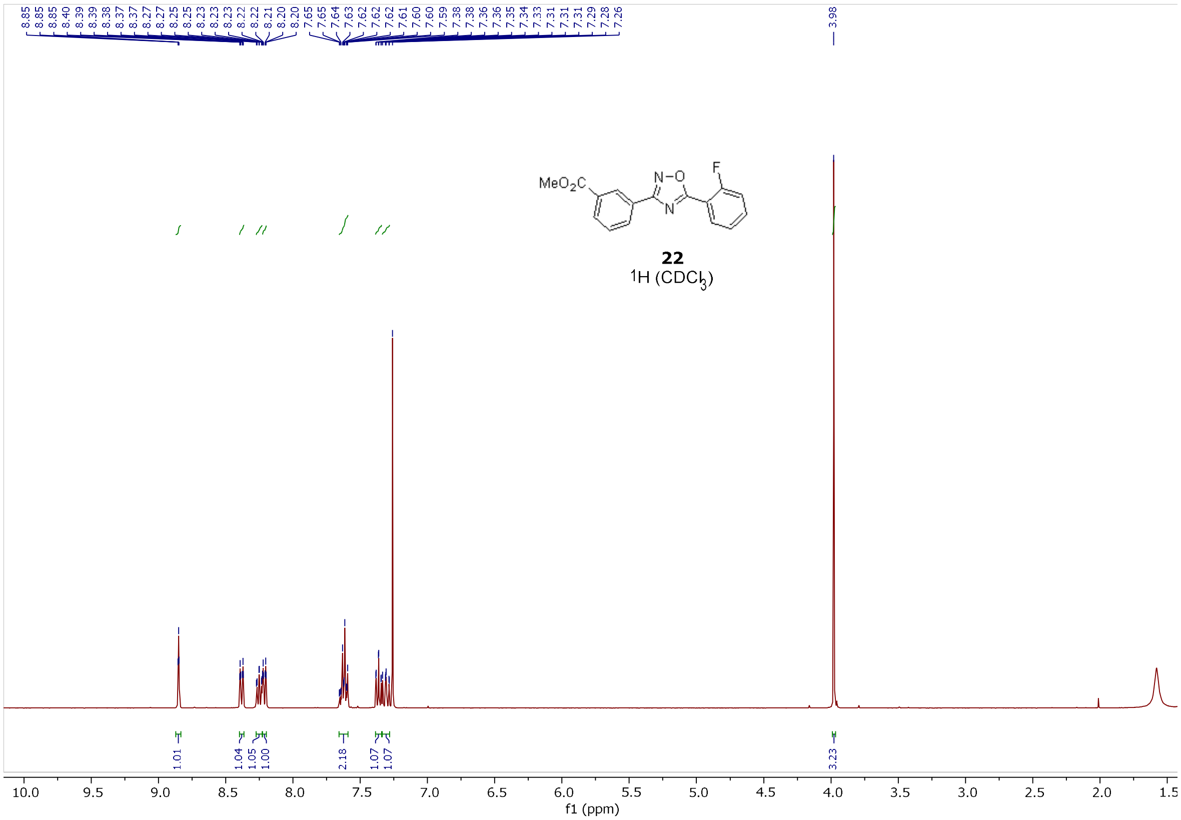

Figure S100. ^{1}H NMR spectrum of **22**.

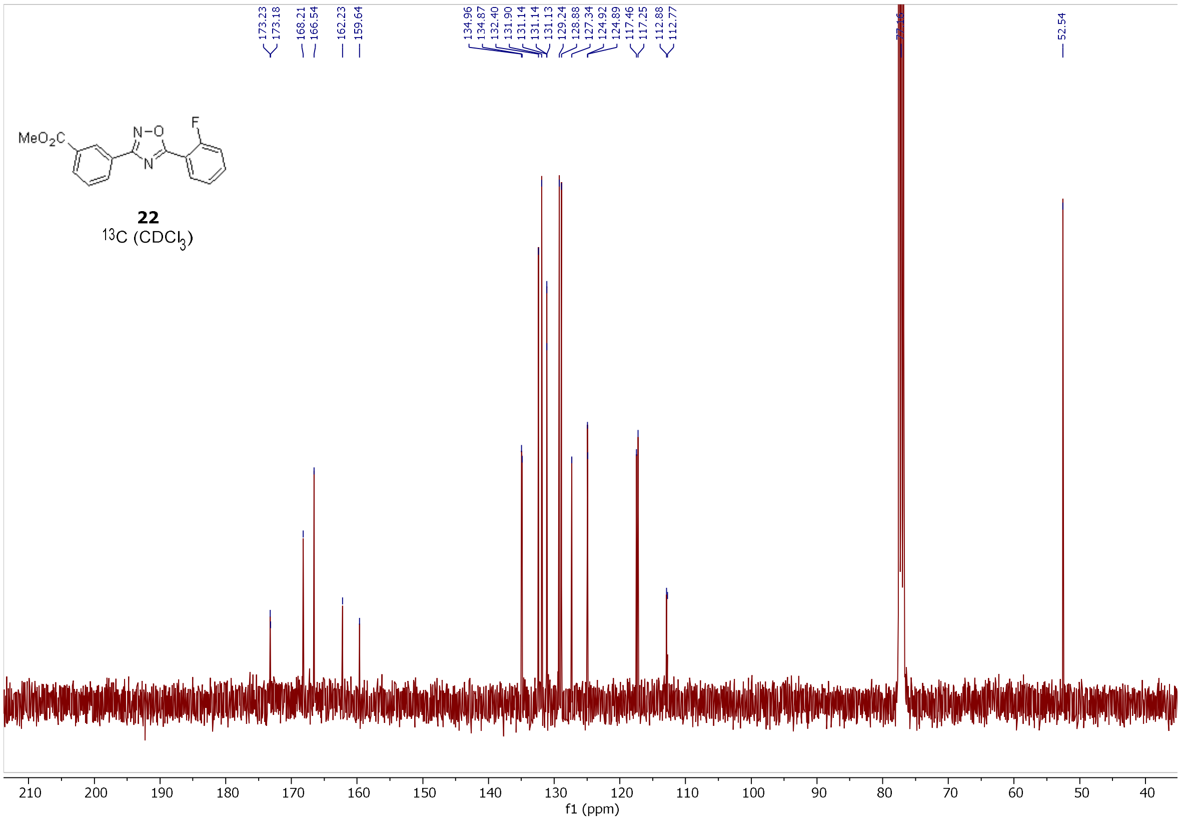

Figure S101. ^{13}C NMR spectrum of **22**.

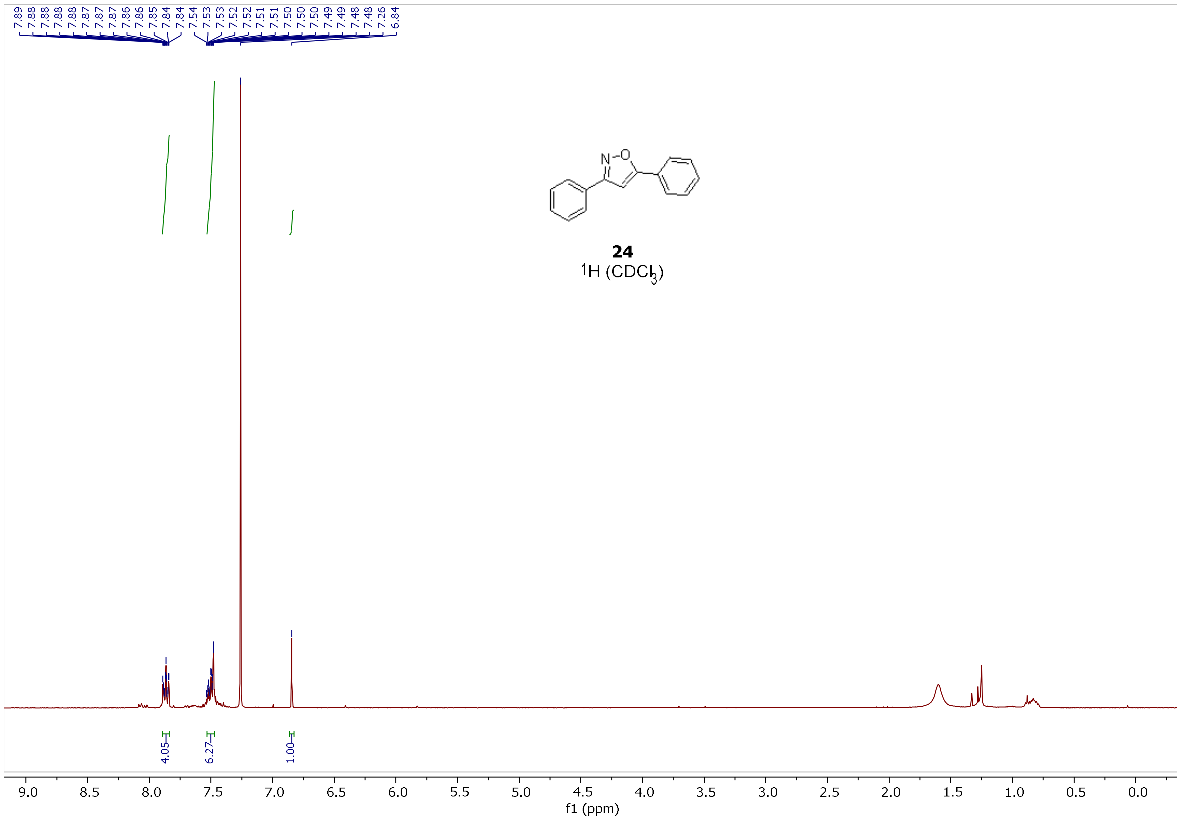

Figure S102. ^{1}H NMR spectrum of **24**.

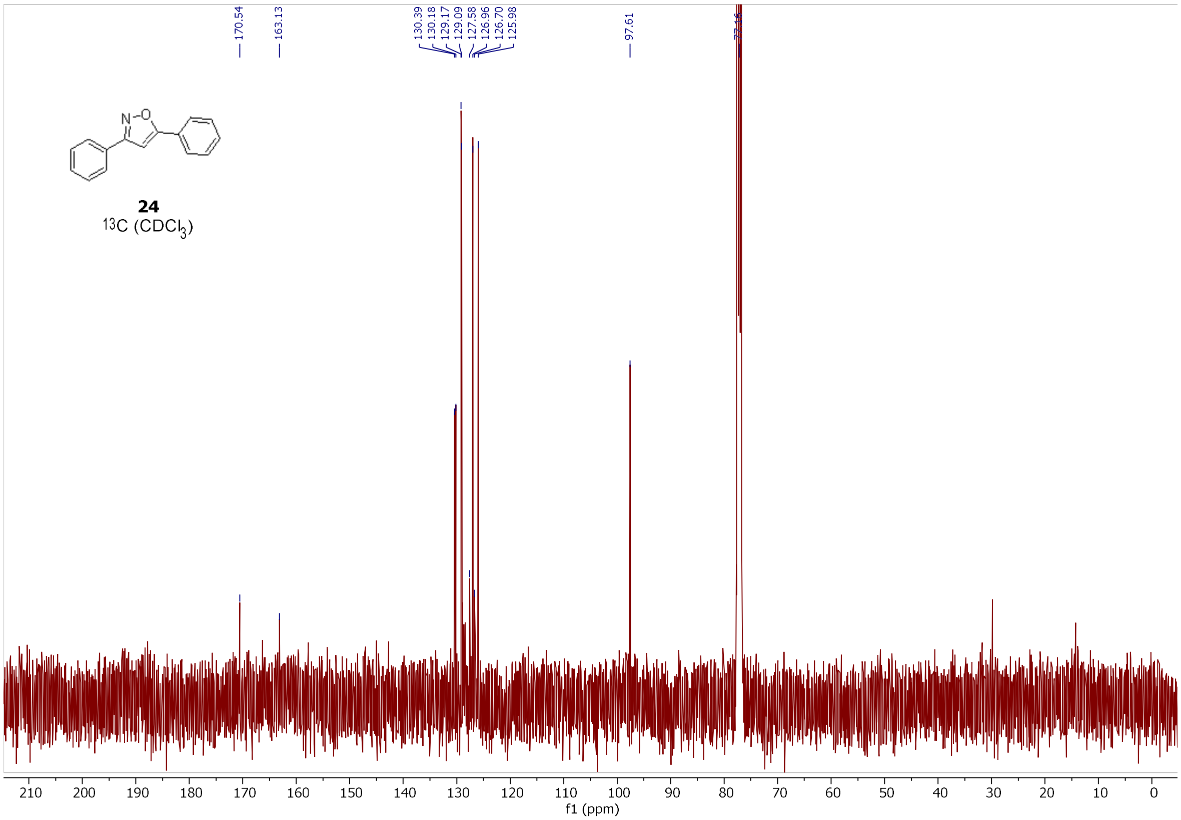

Figure S103. ^{13}C NMR spectrum of **24**.

5.4. NMR spectra of 9,10-diphenylanthracene endoperoxide

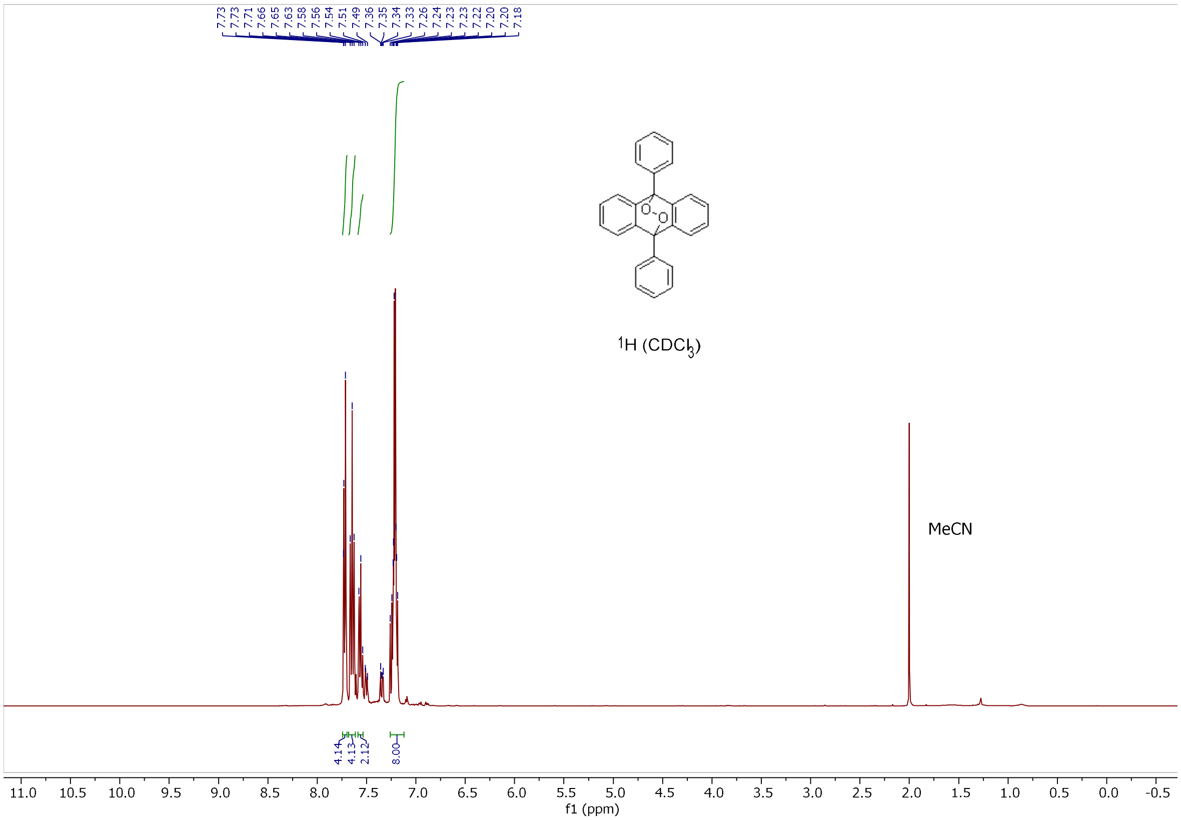

Figure S104. ^{1}H NMR spectrum of **9,10-diphenyl-9,10-dihydro-9,10-epidioxyanthracene**.

5.5. NMR spectra of tentative by-products of oximes 3a and 23a oxidation

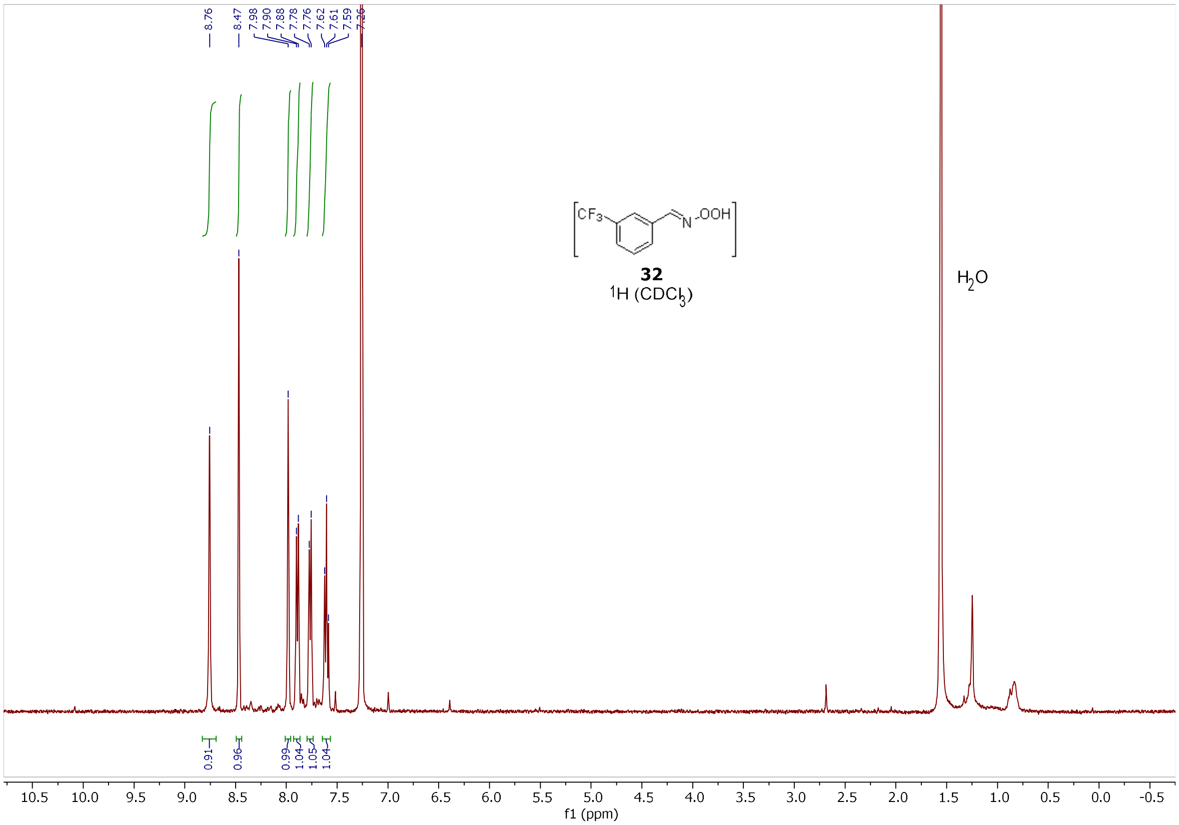

Figure S105. ^{1}H NMR spectrum of **32**.

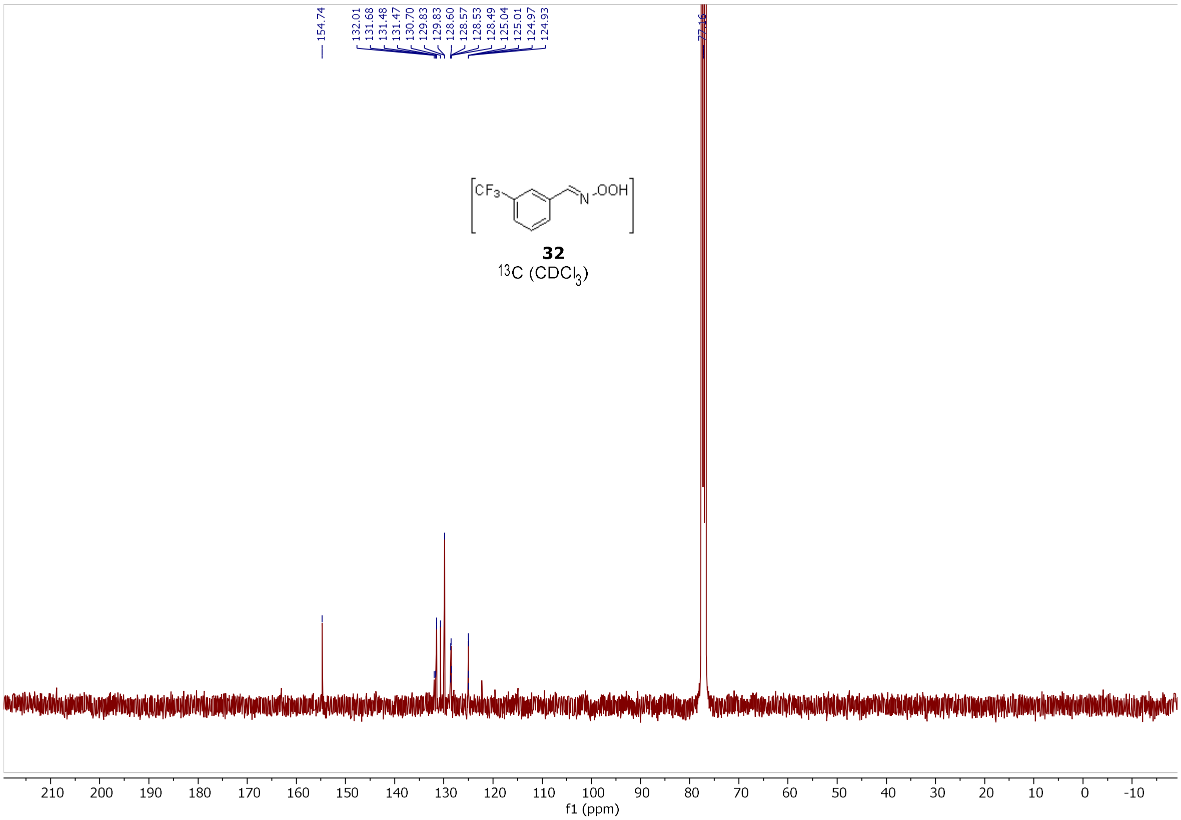

Figure S106. ^{13}C NMR spectrum of **32**.

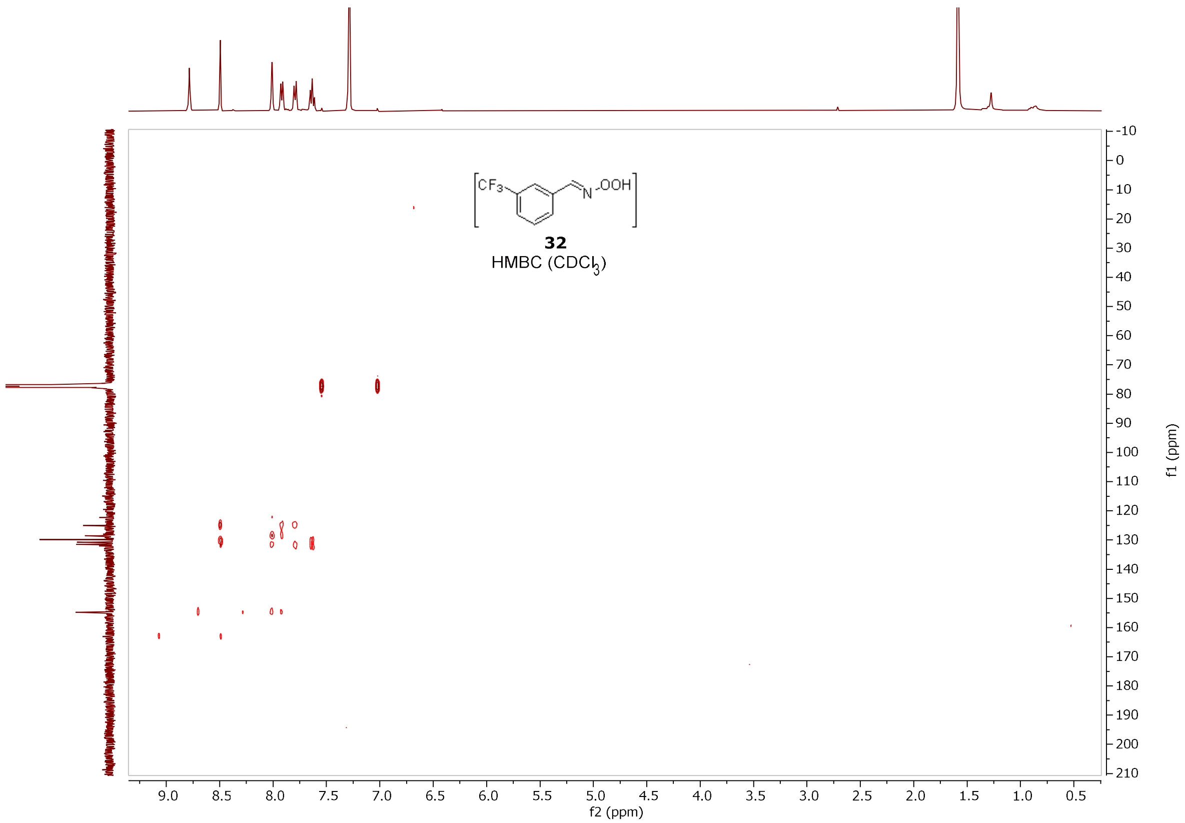

Figure S107. HBMC-NMR spectrum of **32**.

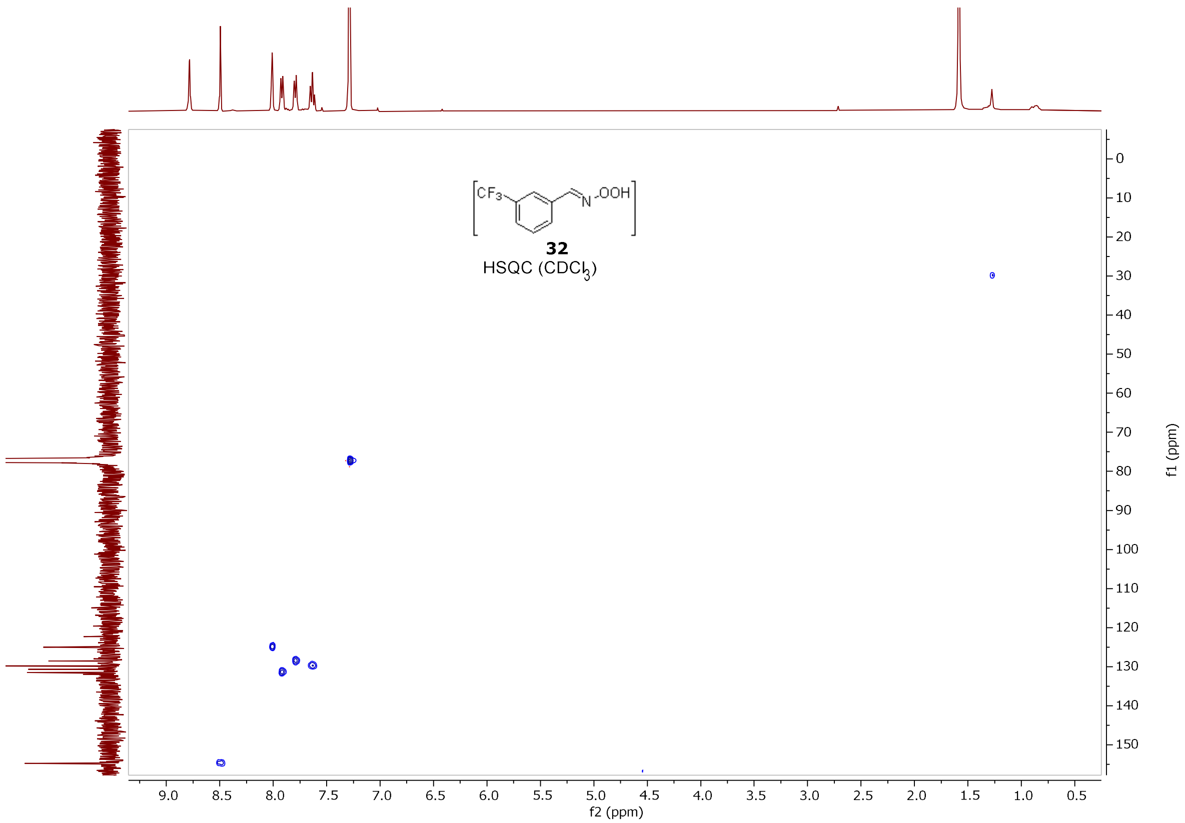

Figure S108. HSQC NMR spectrum of **32**.

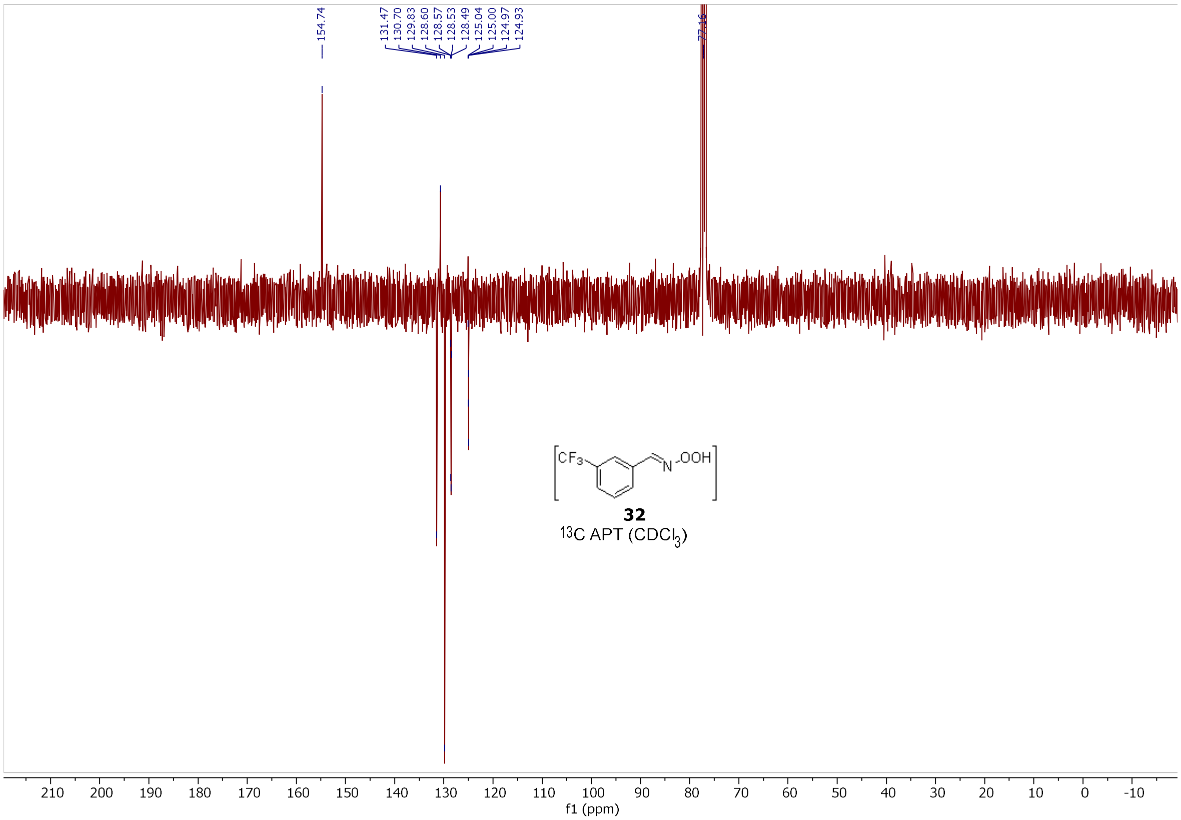

Figure S109. ^{13}C APT NMR spectrum of **32**.

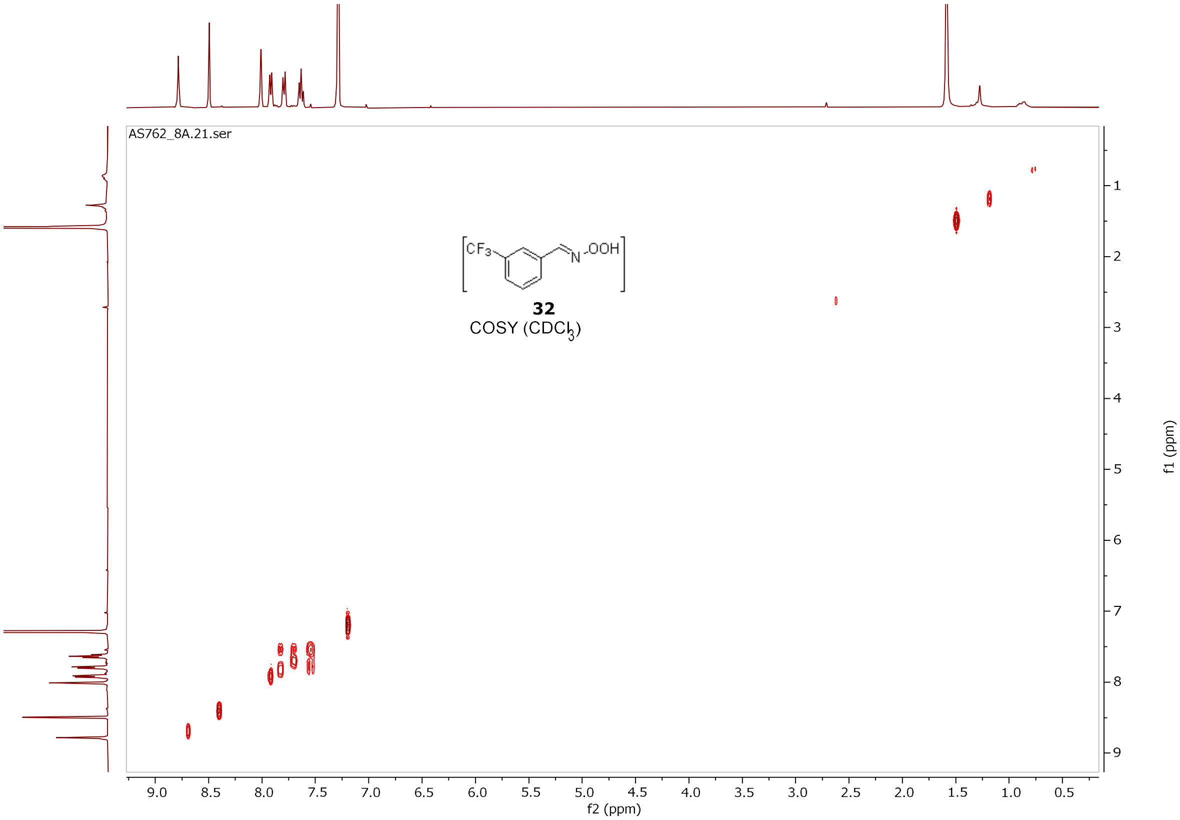

Figure S110. COSY NMR spectrum of **32**.

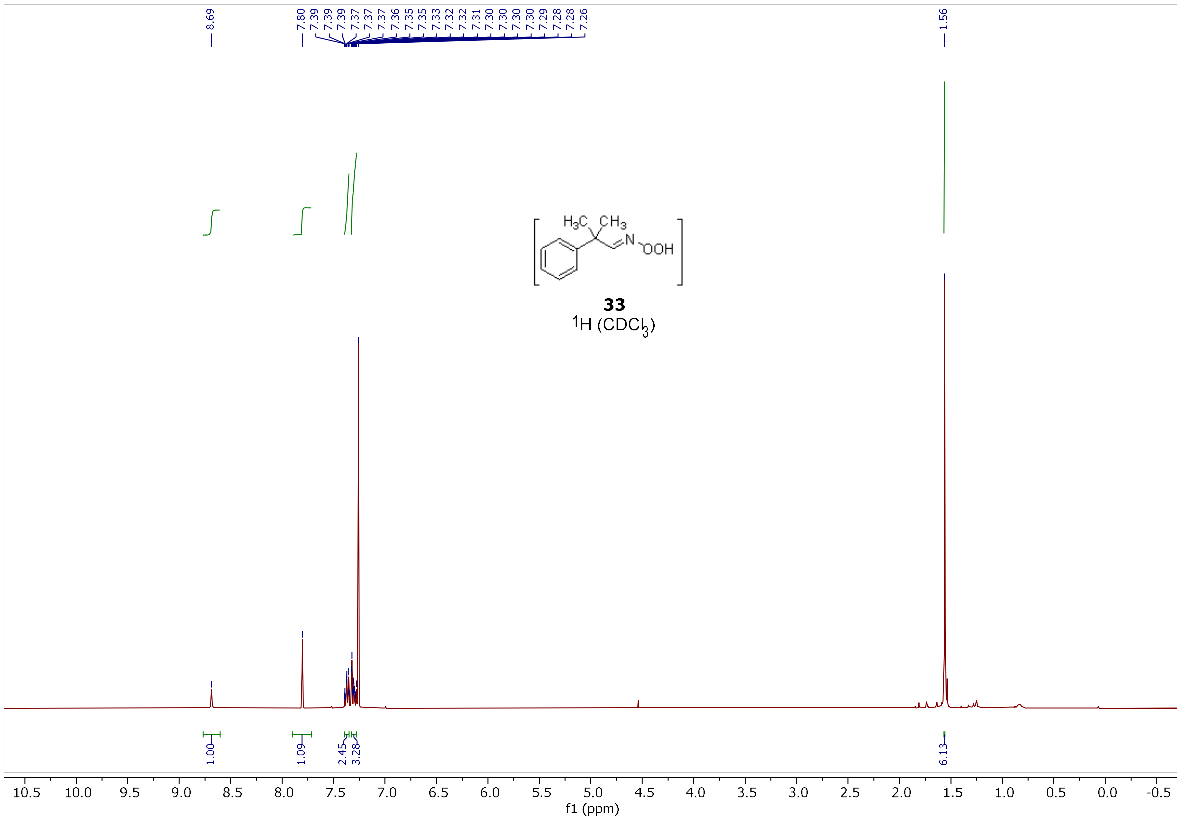

Figure S111. ^{1}H NMR spectrum of **33**.

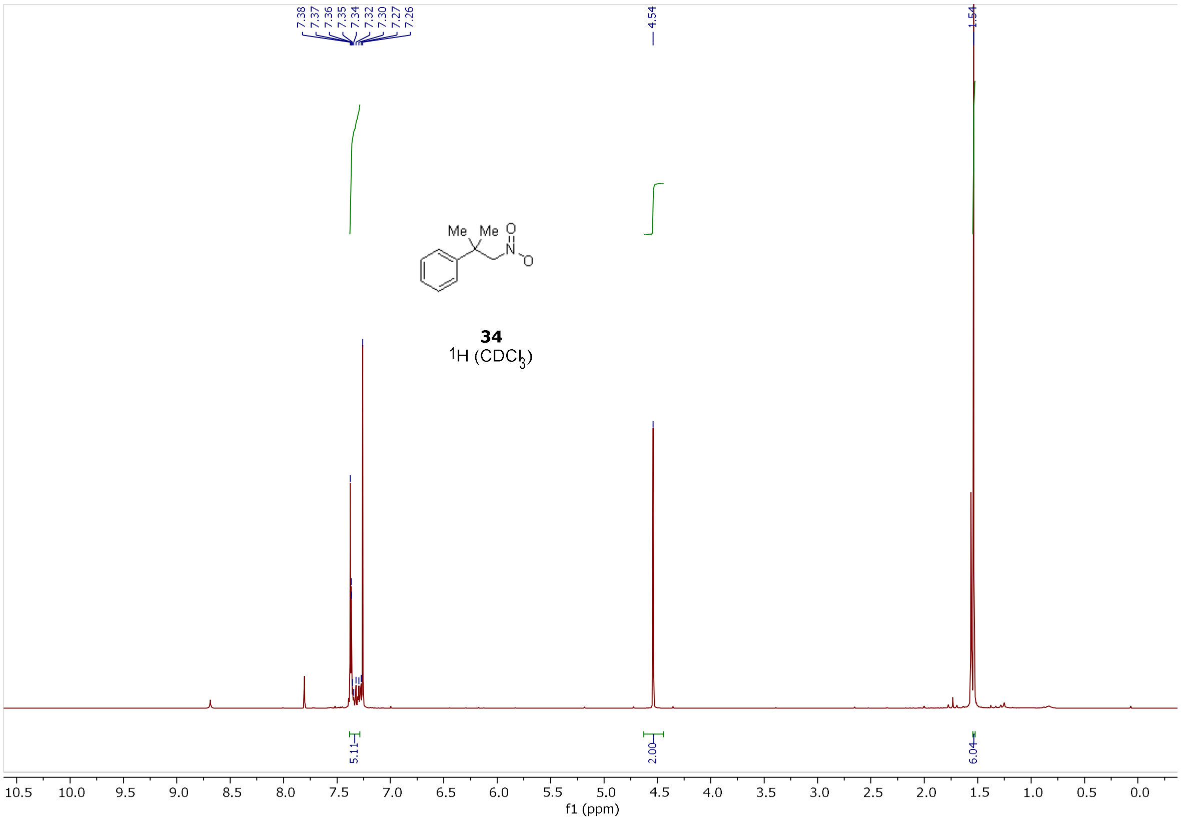

Figure S112. ^{1}H NMR spectrum of **34**.

6. Mass spectra of tentative by-products of oximes 3a and 23a oxidation

6.1. Electron ionization spectra

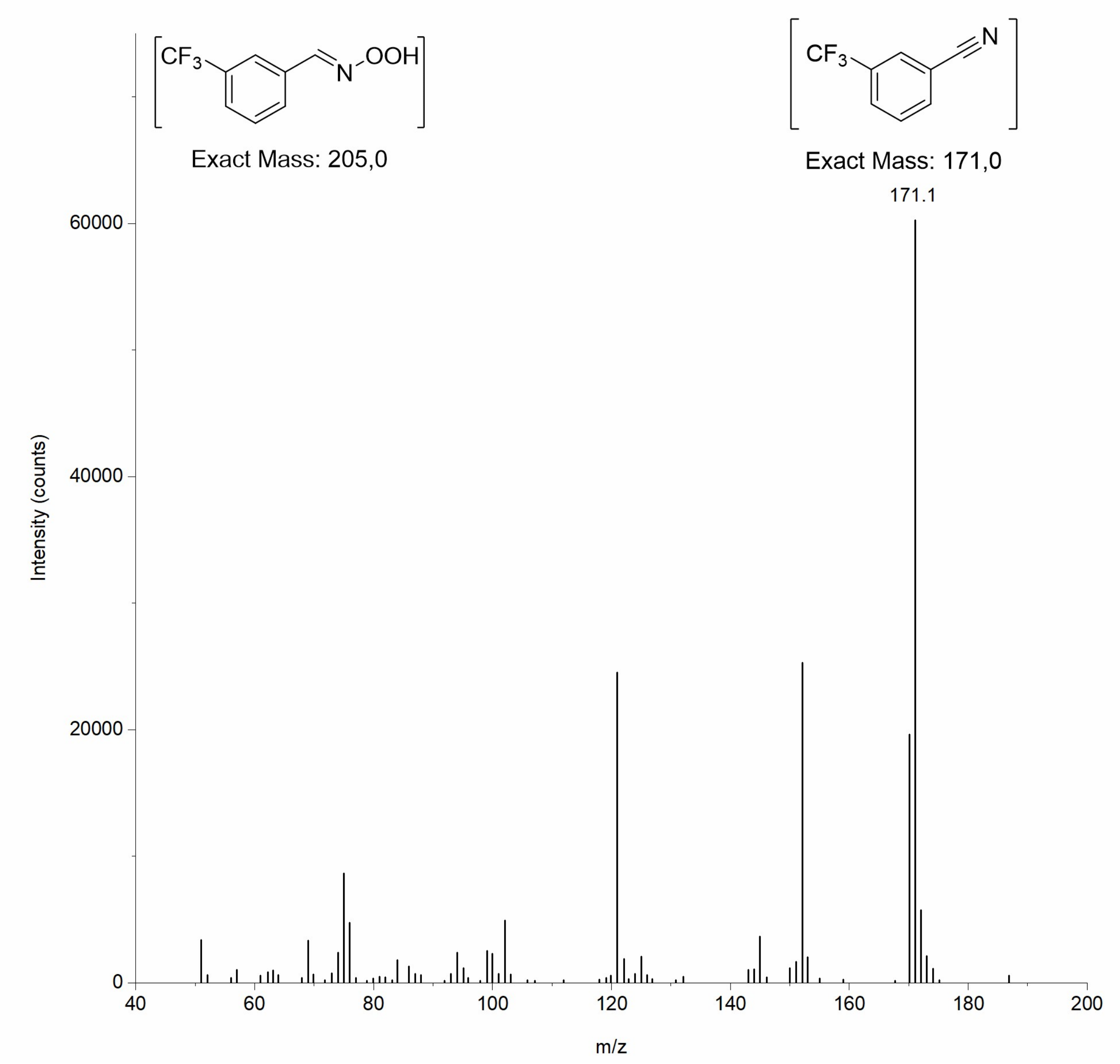

Figure S113. Mass spectrum of **32** with tentative structure. The molecular ion is not observed. The peak with m/z 171.1 might correspond to the nitrile formed from **32** upon loss of H_2O_2.

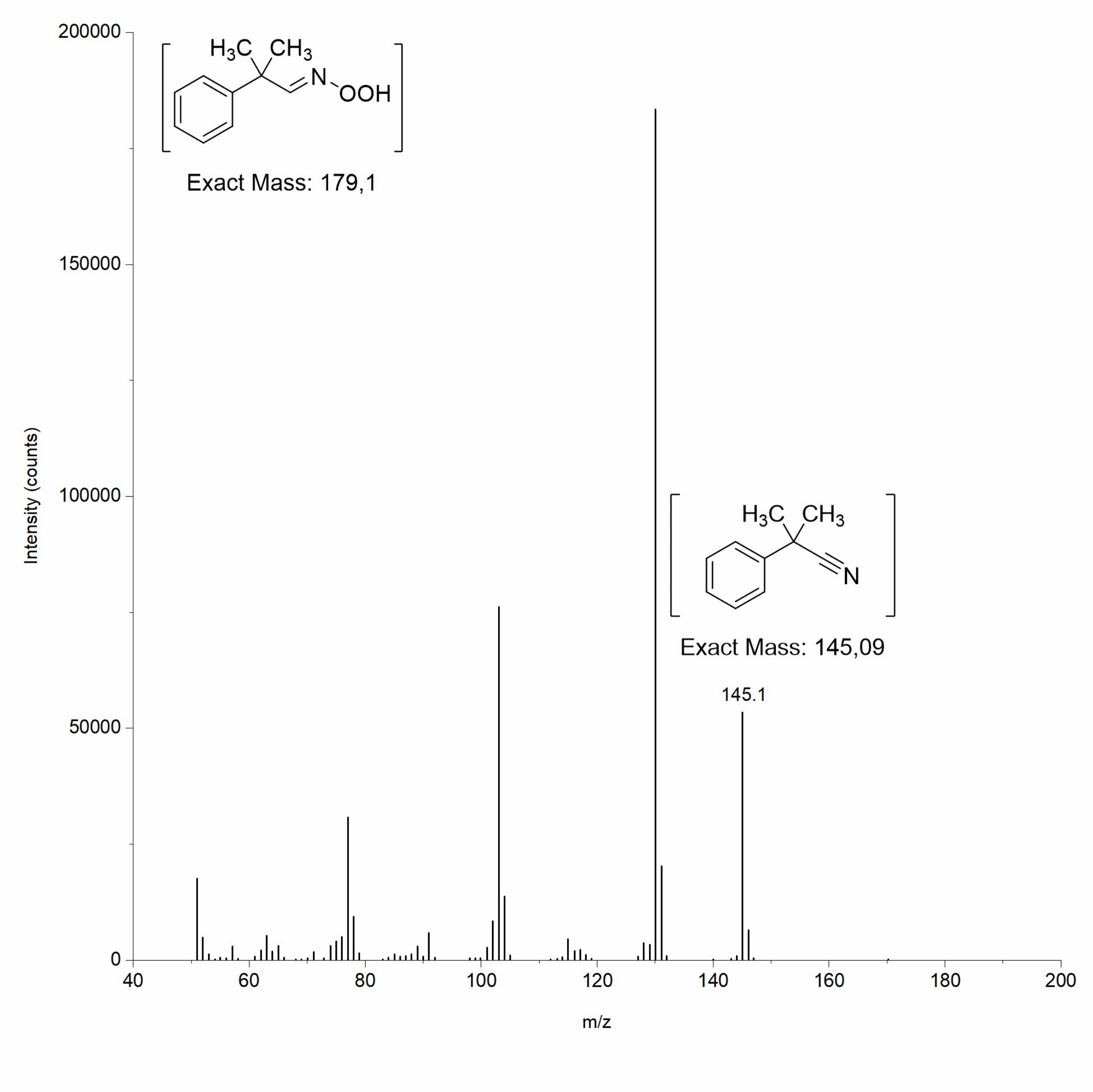

Figure S114. Mass spectrum of **33** with tentative structure. The molecular ion is not observed. The peak with m/z 145.1 might correspond to the nitrile formed from **33** upon loss of H_2O_2.

6.2. High-resolution mass spectra

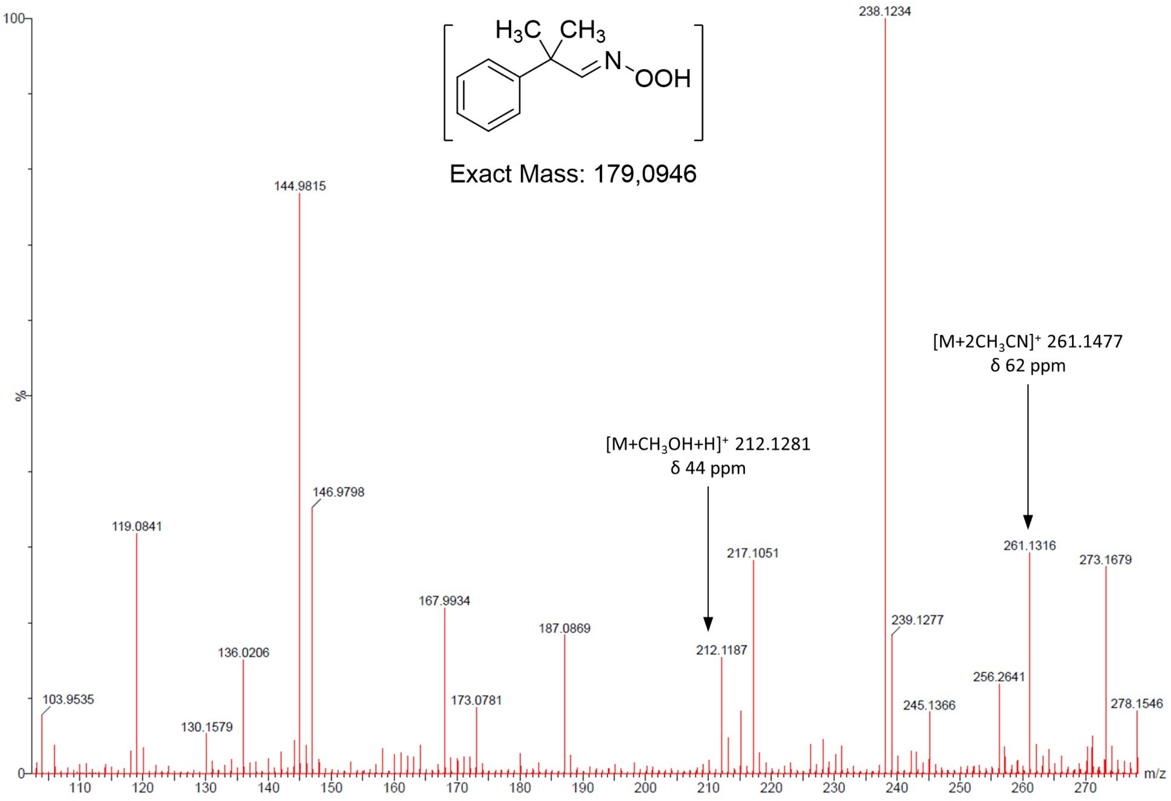

Figure S115. HR-MS spectrum of **33** (positive mode). The adducts of **33** with methanol and acetonitrile are labeled, errors between calculated and experimental masses are shown.

References

[1] B. Kurpil, K. Otte, A. Mishchenko, P. Lamagni, W. Lipiński, N. Lock, M. Antonietti, A. Savateev, *Nat. Commun.* **2019**, *10*, 945.

[2] X. Cai, A. Keshavarz, J. D. Omaque, B. J. Stokes, *Org. Lett.* **2017**, *19*, 2626-2629.

[3] S. Liu, L. S. Liebeskind, *Journal of the American Chemical Society* **2008**, *130*, 6918-6919.

[4] S. Alagaratnam, N. J. Meeuwenoord, J. A. Navarro, M. Hervás, M. A. De la Rosa, M. Hoffmann, O. Einsle, M. Ubbink, G. W. Canters, *The FEBS Journal* **2011**, *278*, 1506-1521.

[5] A. C. Larson, R. B. V. Dreele, *Los Alamos National Laboratory Report LAUR* **1994**, *86-748*.

[6] B. Toby, *J. Appl. Crystallogr.* **2001**, *34*, 210-213.

[7] H. Schlomberg, J. Kröger, G. Savasci, M. W. Terban, S. Bette, I. Moudrakovski, V. Duppel, F. Podjaski, R. Siegel, J. Senker, R. E. Dinnebier, C. Ochsenfeld, B. V. Lotsch, *Chem. Mater.* **2019**, *31*, 7478-7486.

[8] G. Kresse, J. Hafner, *Phys. Rev. B* **1993**, *47*, 558-561.

[9] G. Kresse, J. Hafner, *Phys. Rev. B* **1994**, *49*, 14251-14269.

[10] G. Kresse, J. Hafner, *Phys. Rev. B* **1996**, *54*, 11169-11186.

[11] J. P. Perdew, K. Burke, M. Ernzerhof, *Phys. Rev. Lett.* **1996**, *77*, 3865-3868.

[12] P. E. Blöchl, *Phys. Rev. B* **1994**, *50*, 17953-17979.

[13] S. Grimme, J. Antony, S. Ehrlich, H. Krieg, *The Journal of Chemical Physics* **2010**, *132*, 154104.

[14] H. J. Monkhorst, J. D. Pack, *Physical Review B* **1976**, *13*, 5188-5192.

[15] Y. Xu, S.-P. Gao, *International Journal of Hydrogen Energy* **2012**, *37*, 11072-11080.

[16] W. Wei, T. Jacob, *Physical Review B* **2013**, *87*, 085202.

[17] S. Mazzanti, B. Kurpil, B. Pieber, M. Antonietti, A. Savateev, *Nat. Commun.* **2020**, *11*, 1387.

[18] D. Dontsova, S. Pronkin, M. Wehle, Z. Chen, C. Fettkenhauer, G. Clavel, M. Antonietti, *Chem. Mater.* **2015**, *27*, 5170-5179.

[19] A. Kahn, *Materials Horizons* **2016**, *3*, 7-10.

[20] R. L. Donkers, M. S. Workentin, *Journal of the American Chemical Society* **2004**, *126*, 1688-1698.

[21] D. M. Cottrell, J. Capers, M. M. Salem, K. DeLuca-Fradley, S. L. Croft, K. A. Werbovetz, *Biorg. Med. Chem.* **2004**, *12*, 2815-2824.

[22] C. La Motta, S. Sartini, S. Salerno, F. Simorini, S. Taliani, A. M. Marini, F. Da Settimo, L. Marinelli, V. Limongelli, E. Novellino, *J. Med. Chem.* **2008**, *51*, 3182-3193.

[23] M. R. Mangione, A. Palumbo Piccionello, C. Marino, M. G. Ortore, P. Picone, S. Vilasi, M. Di Carlo, S. Buscemi, D. Bulone, P. L. San Biagio, *RSC Adv.* **2015**, *5*, 16540-16548.

[24] A. U. Meyer, V. W.-h. Lau, B. König, B. V. Lotsch, *Eur. J. Org. Chem.* **2017**, 2179-2185.

[25] Y. Zhao, M. Shalom, M. Antonietti, *Appl. Catal., B* **2017**, *207*, 311-315.

[26] J. D. Griffin, M. A. Zeller, D. A. Nicewicz, *Journal of the American Chemical Society* **2015**, *137*, 11340-11348.

[27] A. Savateev, D. Dontsova, B. Kurpil, M. Antonietti, *J. Catal.* **2017**, *350*, 203-211.
